# Metal-free site-selective functionalization with cyclic diaryl λ^3^-chloranes: suppression of benzyne formation for ligand-coupling reactions†

**DOI:** 10.1039/d4sc04108a

**Published:** 2024-09-13

**Authors:** Koushik Patra, Manas Pratim Dey, Mahiuddin Baidya

**Affiliations:** a Department of Chemistry, Indian Institute of Technology Madras Chennai 600 036 Tamil Nadu India mbaidya@iitm.ac.in

## Abstract

While hypervalent halogens are versatile reagents enabling diverse reactions in organic synthesis, the utility of hypervalent chlorine compounds, particularly cyclic λ^3^-chloranes, remains underdeveloped despite their unique electronic properties and innate enhanced reactivity. Herein, we illustrate the elusive ligand coupling reaction of cyclic λ^3^-chloranes that suppresses the more facile competing reaction modality involving benzyne intermediates. The methodology can be performed in three-component as well as two-component fashions, offering direct access to a wide range of unsymmetrically substituted biaryl molecules in very high yields and excellent *ortho*-regioselectivity. The reactions were scalable, and the versatility was demonstrated by constructing different types of C–S and C–N bonds under mild conditions. The reaction outcomes were also compared with those of corresponding λ^3^-iodanes and λ^3^-bromanes, demonstrating the superiority of cyclic λ^3^-chloranes in ligand-coupling reactions under metal-free conditions.

## Abstract 2

The elusive ligand coupling reaction of cyclic λ^3^-chloranes was developed in three-component and two-component fashion to access a range of unsymmetrically substituted biaryl molecules in very high yields and excellent *ortho*-regioselectivity.

## Graphical abstract

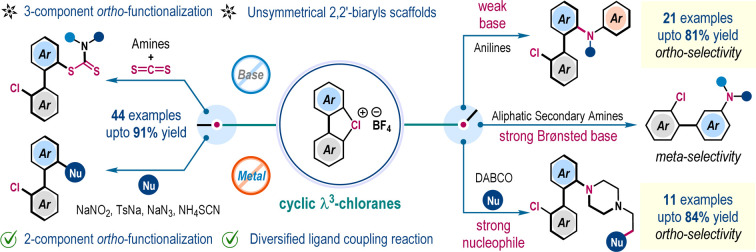

## Introduction

**Scheme 1:**
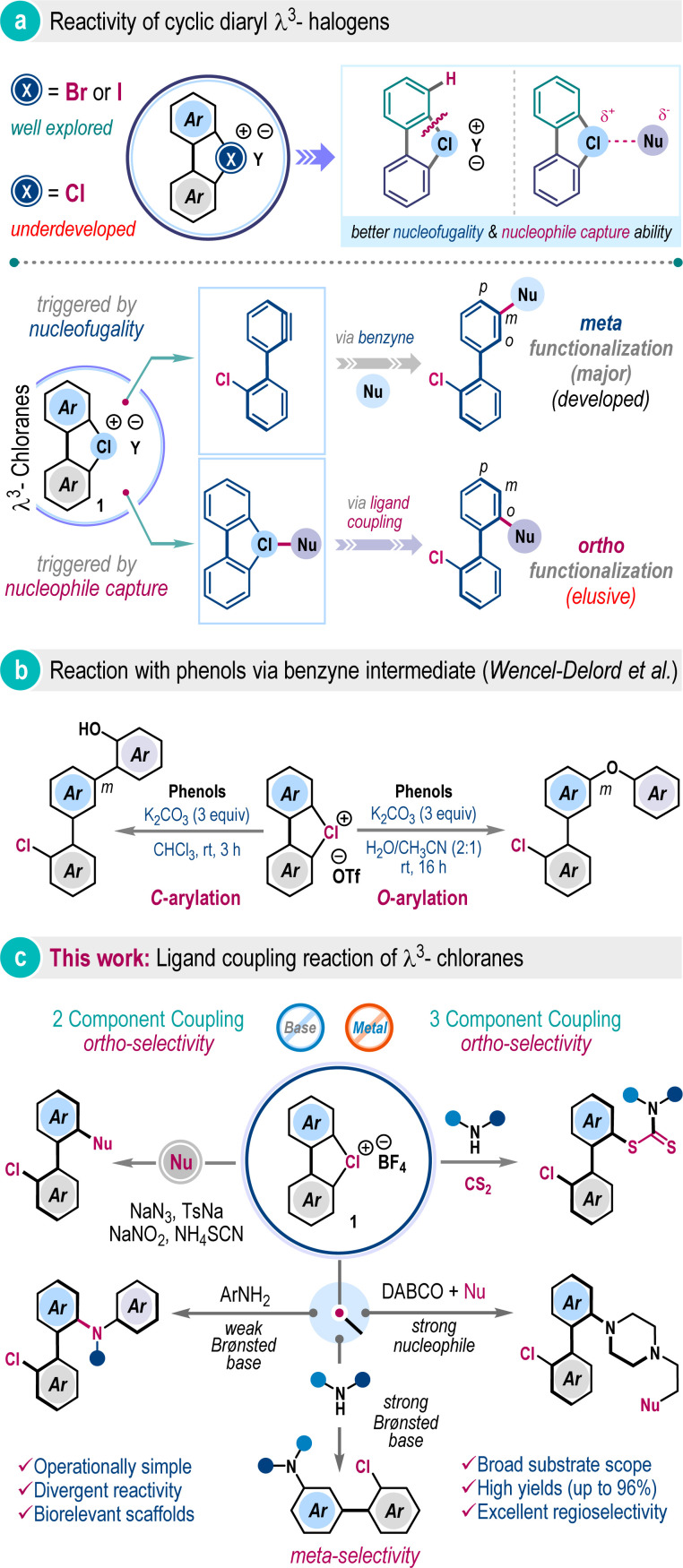
Ligand coupling strategies for the synthesis of unsymmetrical biaryls.

Hypervalent halogen compounds have emerged as versatile reagents in contemporary organic synthesis, enabling a wide array of transformations to harvest molecular complexity under mild conditions.^1^ Their unique electronic structures and properties, including low toxicity, tunable reactivity, compatibility with diverse organic functional groups, and facile synthesis have significantly enhanced their utility in chemical science.^1,2^ In this realm, λ^3^-iodanes and λ^3^-bromanes have been studied extensively; however, in sharp contrast, their congener λ^3^-chloranes have received little attention, although their elegant syntheses have been disclosed previously (Scheme 1a).^3^ Particularly, the cyclic diaryl λ^3^-chloranes (1) are intriguing, and due to the elevated electronegativity and ionization potential of the chlorine atom, these compounds are expected to exhibit increased nucleofugality and a higher tendency to capture nucleophiles (Scheme 1a).^3*b*,*e*^ The nucleofugality property can be manifested as a benzyne intermediate under basic conditions, which can then be trapped by a suitable nucleophile to facilitate steric-effect governed preferential *meta*-functionalization (Scheme 1a).^4,5^ Conversely, the reactivity of nucleophile capture can be translated into ligand coupling reaction, thus directing *ortho*-functionalization processes (Scheme 1a, below).^6^ Recently, the Wencel-Delord group elucidated selective C–O and C–C bond-forming reactions of diaryl λ^3^-chloranes with phenols under basic conditions (Scheme 1b).^5*a*^ They also showcased halogenation reaction with tetrabutylammonium halides.^5*b*^ To the best of our knowledge, these are the only few reported instances concerning cyclic diaryl λ^3^-chloranes, operating through a benzyne intermediate. Currently, a general method for ligand coupling reactions in cyclic diaryl λ^3^-chloranes, enabling *ortho*-functionalization, remains unexplored, despite the pyrolysis of cyclic diaryl λ^3^-chloranes, leading to 2,2-dihalogenobiphenyls being reported over fifty years ago.^7^

The *ortho*-selective functionalization of cyclic diaryl λ^3^-chloranes is increasingly challenging as the benzyne formation is highly facile with a very low energy barrier.^5*a*^ We envisioned that potent nucleophiles with very low Brønsted basicity would suppress the competitive benzyne formation, and such a scenario will constitute the direct interaction with the hypervalent halogen center significantly, which may materialize the desired ligand coupling reaction. If successful, this methodology will allow *ortho*-selective coupling of cyclic diaryl λ^3^-chloranes under metal-free conditions.

Herein, we demonstrated the first example of such a reaction through the development of a three-component coupling involving cyclic diaryl λ^3^-chloranes, carbon disulfide, and amines (Scheme 1c).^8^ The protocol is also operational in a two component fashion with a range of nucleophiles including aromatic amines, aryl sulfinate, nitrite, thiocyanate, and azide, offering biologically relevant and unsymmetrical biaryl frameworks in excellent yields. We also showcased the *meta*-selective functionalization of cyclic diaryl λ^3^-chloranes by employing amines with higher Brønsted basicity, routing to the benzyne mechanism and substantiating our hypothesis (Scheme 1c).

Further advancement in *ortho*-selective ligand coupling reaction has also been accomplished by engaging tertiary amines and subsequent trapping with different nucleophiles, dispensing uniquely functionalized N-heterocycles. These processes are operationally simple, scalable, and external additive-free, and display a wide substrate generality with excellent site selectivity. Fundamental innovation of this work relies on critically suppressing benzyne formation while selectively promoting the heretofore unknown ligand coupling reaction of cyclic diaryl λ^3^-chloranes.

## Results and discussion

Functionalized dithiocarbamate frameworks are prevalent in bioactive compounds.^9^ We envisaged that three-component coupling among cyclic diaryl λ^3^-chloranes 1, carbon disulfide (CS_2_), and amine would provide valuable chemical space encompassing biaryl frameworks. Accordingly, λ^3^-chlorane 1a (1.0 equiv.) was exposed to a mixture of carbon disulfide 2a (CS_2_, 2.5 equiv.) and pyrrolidine 3a (1.2 equiv.) in DCE at room temperature. Gratifyingly, the three-component coupling proceeded smoothly and the biaryl dithiocarbamate 4a was isolated in 72% yield (entry 1). Of note, this reaction is exclusively *ortho*-selective (with respect to chloro-phenyl substituent), and we did not detect any *meta*-functionalization product, indicating a preferential ligand coupling reaction over benzyne intermediate formation, which can be attributed to the poor Brønsted basicity and notable nucleophilicity of the *in situ* generated dithiocarbamate ion (R_2_N–CS_2_^−^). Screening of other solvents such as THF, CH_3_CN, DMF, and MeOH gave detrimental outcomes with reduced yields of 4a, while the reaction was unfruitful in HFIP medium (entries 1–3). However, the reaction yield significantly improved in DCM solvent, offering 4a in 88% isolated yield (entry 4). Examination of the counter-ion in cyclic diaryl λ^3^-chlorane revealed a comparable reactivity for the tosylate counter anion, but the production of 4a was marginally dropped to 70% for the triflate counter anion (entry 5). The increase of the reaction temperature to 50 °C also resulted in a decrease in yield to 66% (entry 6). To comprehend the reactivity of other cyclic diaryl λ^3^-halogens, we performed the reaction with λ^3^-bromane 1a′ and λ^3^-iodane 1a′′ (Table 1, below). Interestingly, λ^3^-bromane 1a′ exhibited moderate reactivity to dispense *ortho*-selective bromo-analog 5a in 69% yield. In sharp contrast, λ^3^-iodane 1a′′ did not furnish the desired iodo-analog 6a at room temperature and a poor conversion was noticed upon increasing the temperature up to 50 °C (Table 1, below). Of note, no *meta*-product was detected for these cases.

**Table 1:** Optimization of reaction conditions^a^

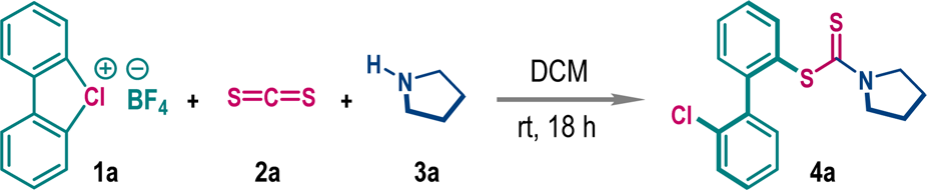		
Entry	Deviation from the standard conditions	Yield of 4a^b^ (%)
1	DCE/THF instead of DCM	72/55
2	CH_3_CN/DMF instead of DCM	48/37
3	MeOH/HFIP instead of DCM	65/NR
**4**	**None**	**88**
5	OTs anion/OTf anion instead of BF_4_ anion	81/70
6	50 °C instead of rt	66
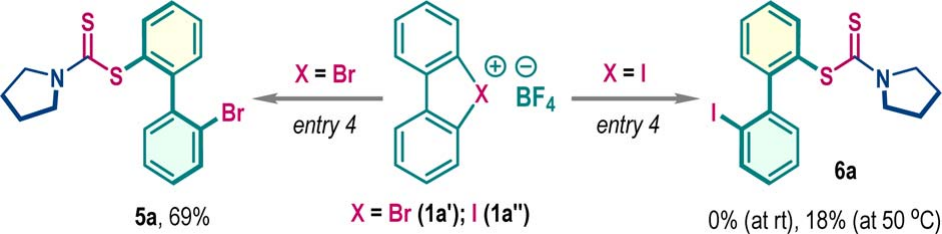		

a Reaction conditions: 1a (0.2 mmol), 2a (0.5 mmol), 3a (0.24 mmol), and solvent (1.5 mL), 18 h, under a N_2_ atmosphere.

b Isolated yields were provided.

**Scheme 2:**
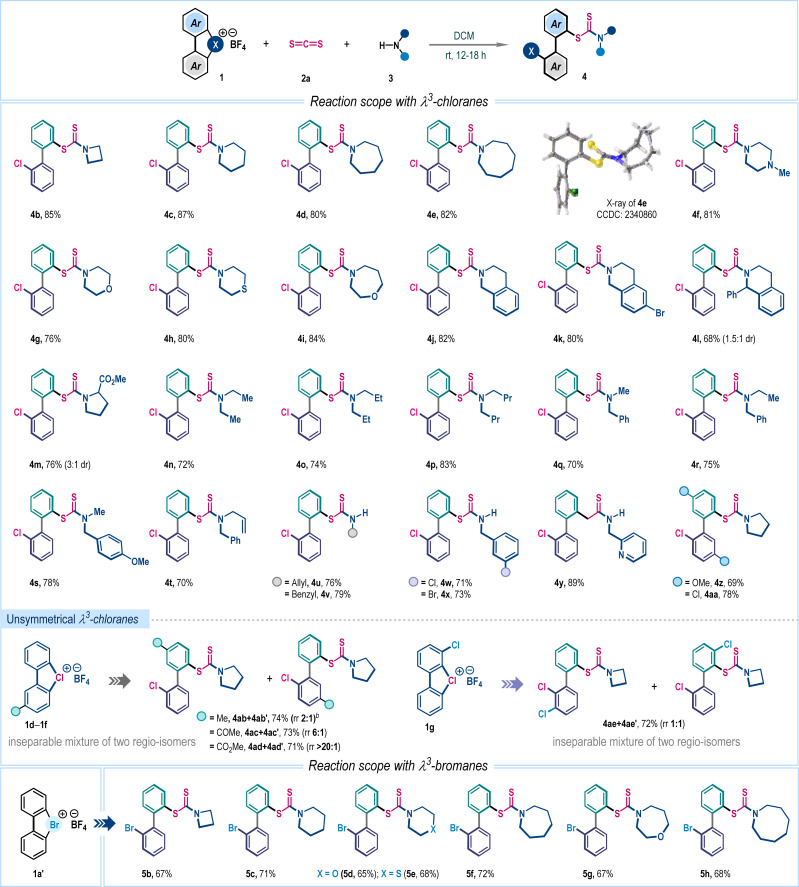
Exploration of the three-component reaction scope^*a*^. ^*a*^Reaction conditions: 1 (0.2 mmol), 2a (0.5 mmol), 3 (0.24 mmol), and DCM (1.5 mL), 18 h, under a N_2_ atmosphere. ^*b*^The rr value represents only the regioisomeric ratio and, at this juncture, which isomer was formed as the major is not apparent.

With the optimized reaction conditions in hand, we then explored the substrate generality of this three-component reaction with an array of structurally diverse amines 3 (Scheme 2). Cyclic aliphatic amines with different ring sizes such as azetidine (4b), piperidine (4c), azepane (4d), azocane (4e), *N*-methylpiperazine (4f), morpholine (4g), thiomorpholine (4h) and oxazepane (4i) smoothly participated in this reaction, furnishing corresponding biaryl dithiocarbamates in very high yields. Compound 4e was crystalized and the single crystal X-ray analysis unambiguously confirmed the product structure and regioselectivity. The methodology was also suitable for several tetrahydroisoquinoline derivatives to dispense 4j–4l in good to high yields. Interestingly, biologically relevant cyclic amino acid ester was also an effective substrate for this reaction, affording 4m in 76% yield. Later, several acyclic amines, symmetrical (4n–4p) and unsymmetrical (4q–4t), were examined and in all cases desired products were isolated in uniformly high yields (70–83%). Delightfully, dithiocarbamate ions generated with primary amines can be accommodated to access 4u–4y in high yields. Variation in λ^3^-chloranes (1) was also considered. The electron-donating methoxy and electron-withdrawing chloro-substituted λ^3^-chloranes delivered functionalized dithiocarbamates 4z and 4aa in 69% and 78% yields, respectively, with exclusive *ortho*-selectivity. When examining the unsymmetrical λ^3^-chlorane 1d–1g, we obtained a mixture of regioisomers (Scheme 2). This variation is likely due to ligand coupling occurring at different aryl rings of the unsymmetrical λ^3^-chloranes. We found that the electronic nature of substituents significantly affects the distribution of regioisomers, particularly for electron-withdrawing groups. For instance, the λ^3^-chlorane 1e, which has a keto group, produced a product with a regioisomeric ratio of 6 : 1. In contrast, the ester-substituted λ^3^-chlorane 1f resulted in an excellent regioisomeric ratio of over 20 : 1, with the ligand-coupling reaction preferentially occurring on the aryl ring bearing the ester functionality.^2*a*,6*b*^ The three-component coupling was also explored with λ^3^-bromane where the desired bromo-aryl substituted dithiocarbamates 5b–5h were isolated in moderate to good yields (65–72%, Scheme 2).

**Scheme 3:**
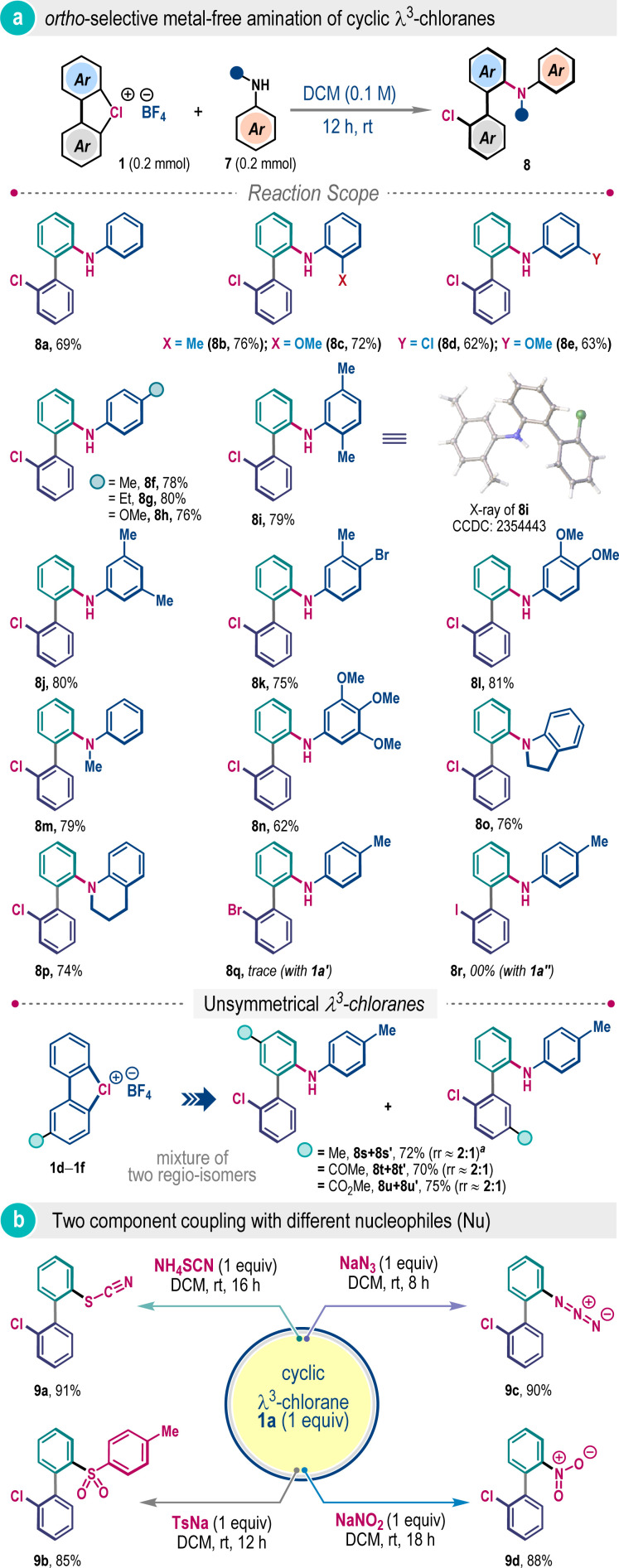
Two-component couplings for *ortho*-substituted biaryls^*a*^. ^*a*^Reaction conditions: 1a (0.2 mmol), amine/Nu (0.2 mmol), DCM (1.5 mL). The rr value represents only the regioisomeric ratio and, at this juncture, which isomer was formed as the major is not apparent.

Next, we wonder whether amines can be directly used for the ligand coupling reaction of λ^3^-chloranes in a two-component fashion. Given the poor Brønsted basicity of aromatic amines, we posited that aniline and derivatives thereof could be appropriate coupling partners. Pleasingly, when aniline was exposed to λ^3^-chlorane 1a in DCM at room temperature, the desired *ortho*-selective ligand coupling was successful to deliver 2′-chloro-[1,1]-biphenyl-2-amine 8a in 69% yield (Scheme 3a). The reaction is quite general for various *N*-free and *N*-substituted anilines with electronically and sterically diverse substitution patterns, allowing construction of a small library of valuable 2-aminobiphenyls (8b–8p). The *ortho*-selectivity was also validated through the single crystal X-ray analysis of compound 8i. It is important to mention that such ligand coupling did not take place when λ^3^-bromane 1a′ or λ^3^-iodane 1a′′ was examined, highlighting the unique reactivity of λ^3^-chlorane (Scheme 3a). The two-component reactions of unsymmetrical λ^3^-chlorane 1d–1f were also explored with *p*-toluidine under the standard reaction conditions (Scheme 3a). Unlike the three-component system discussed previously (Scheme 2), the influence of substituents on regioisomer distribution for these cases was marginal and the desired biaryl products were isolated with an approximately 2 : 1 regioisomeric ratio.

To extend the scaffold diversity further, we investigated two-component ligand coupling reactions with other sulfur and nitrogen nucleophiles (Scheme 3b). The coupling reaction was amenable with NH_4_SCN and sodium *p*-toluenesulfinate (TsNa), furnishing *ortho*-functionalized biaryls 9a and 9b in 91% and 85% yields, respectively. Reactions with nitrogen nucleophiles such as NaN_3_ and NaNO_2_ also successfully led to the production of useful azide and nitro-substituted unsymmetrical biaryls 9c and 9d in excellent yields with exclusive *ortho*-selectivity (Scheme 3b).

**Scheme 4:**
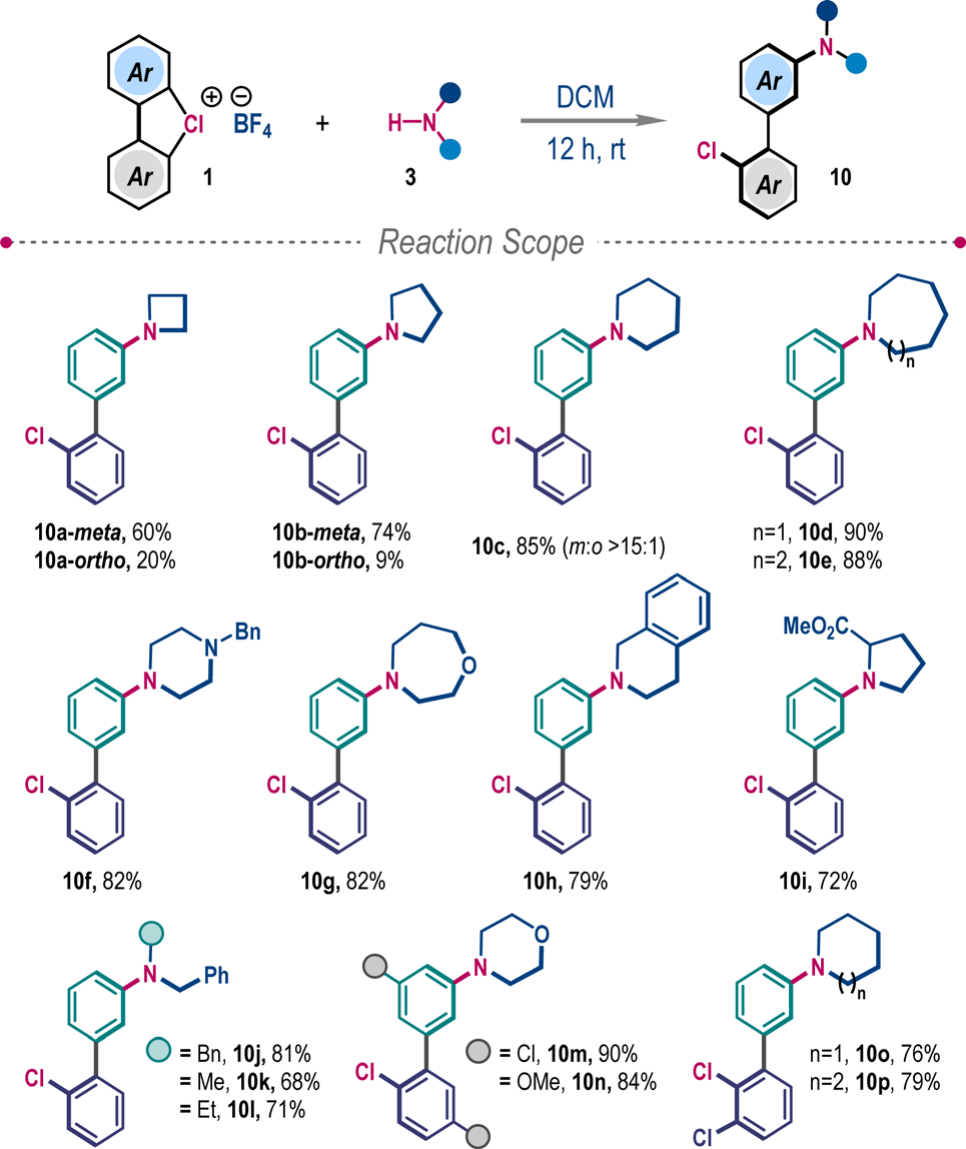
*Meta*-selective amination of cyclic diaryl λ^3^-chloranes^*a*^. ^*a*^Reaction conditions: 1 (0.2 mmol), 3 (1.2 equiv.), DCM (1.5 mL), 12 h.

When amines with higher Brønsted basicity were employed, in line with our hypothesis, they triggered the competitive functionalization through the benzyne intermediate (Scheme 4). Reactions of λ^3^-chlorane 1a with cyclic secondary amines, azetidine and pyrrolidine, furnished a mixture of *meta*- and *ortho*-functionalized products 10a–10b. However, the selectivity towards *meta*-functionalization gradually increased with the increasing ring size of cyclic amines, supplying 10c–10h in very high yields. Sterically bulky proline methyl ester and different *N*-substituted benzylamines also provided desired 3-aminobiphenyls 10i–10l. The protocol is also suitable for other substituted λ^3^-chlorane 1 to give 10m–10p in high yields and exclusive *meta*-selectivity (Scheme 4).

**Scheme 5:**
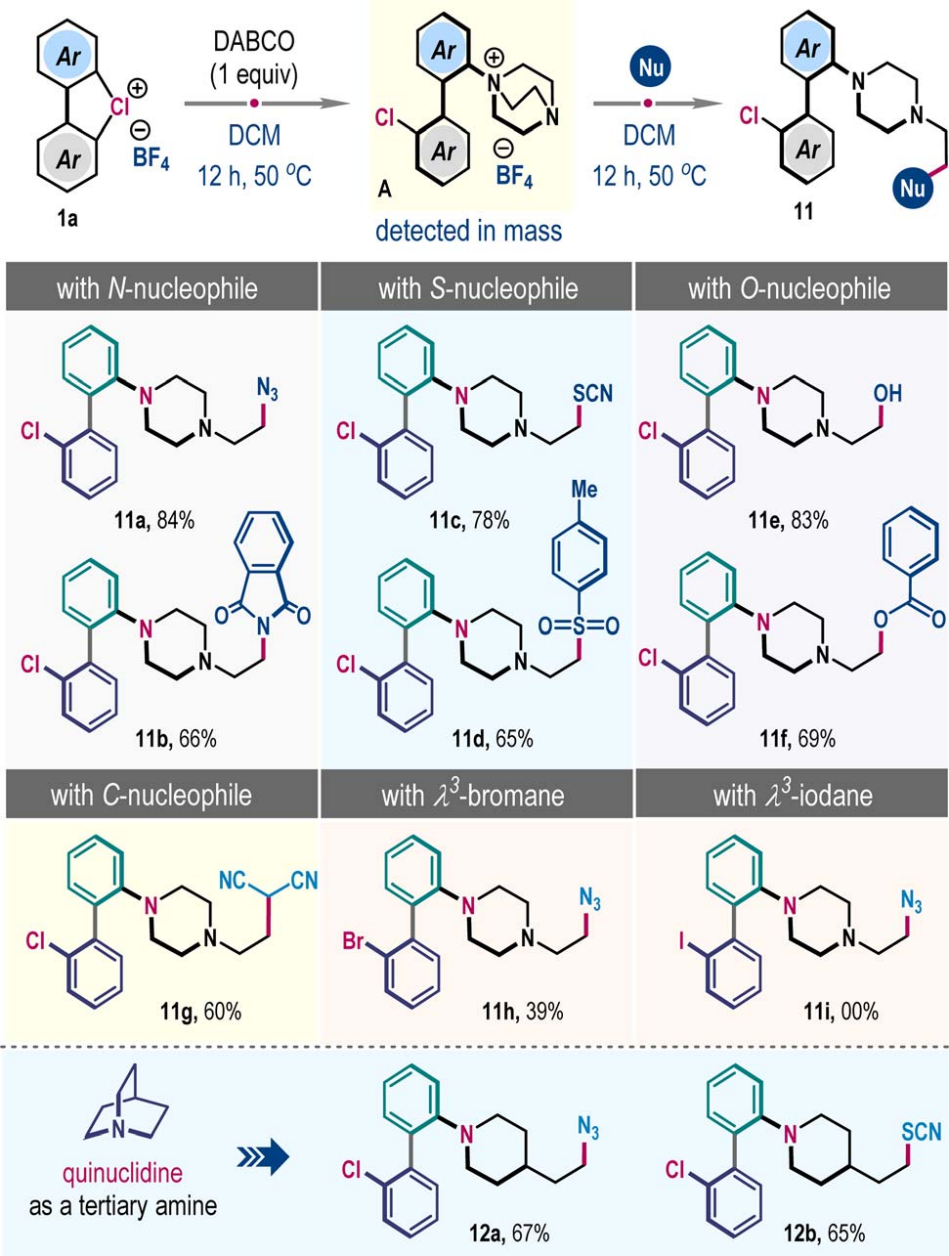
Incorporation of bicyclic amines in ligand coupling reaction.

Previously, Mayr *et al.* demonstrated that unhindered tertiary amine DABCO (1,4-diazabicyclo[2.2.2]octane) has superior nucleophilic reactivity.^10^ We anticipated that such a trait might favor ligand coupling reaction to generate ammonium salt A, which can be subsequently reacted with a second nucleophile to effect distinct *ortho*-functionalization of λ^3^-chloranes 1 (Scheme 5). Accordingly, a mixture of 1a and DABCO in DCM was heated for 12 h, and then NaN_3_ was introduced into the reaction mixture. Thrillingly, we obtained the formal three-component coupling product 11a in 84% yield, which involves the opening of a bicyclo[2.2.2]octane framework. The protocol is operational with other nitrogen (potassium phthalimide), sulfur (NH_4_SCN and TsNa), and oxygen (H_2_O and PhCOOK) nucleophiles, producing 11b–11f in high yields (Scheme 5). The reaction with a carbon nucleophile was also feasible; for example, the sodium salt of malononitrile furnished 11g in 60% yield. However, the λ^3^-bromane 1a′ exhibited moderate reactivity, giving 11h only in 39% yield, while such coupling with λ^3^-iodane 1a′′ was unsuccessful (Scheme 5). We have also examined this three-component reaction through the intermediacy of quinuclidine salt instead of DABCO salt. Satisfyingly, the coupling was successful, dispensing 12a and 12b in 67% and 65% yields, respectively (Scheme 5, below). Of note, this strategic blueprint not only complements the *meta*-functionalization outlined in Scheme 4 but also paves the way to formally achieve *ortho*-selective ligand coupling products of secondary amines with λ^3^-chloranes 1, a challenge hitherto formidable to surmount.

**Scheme 6:**
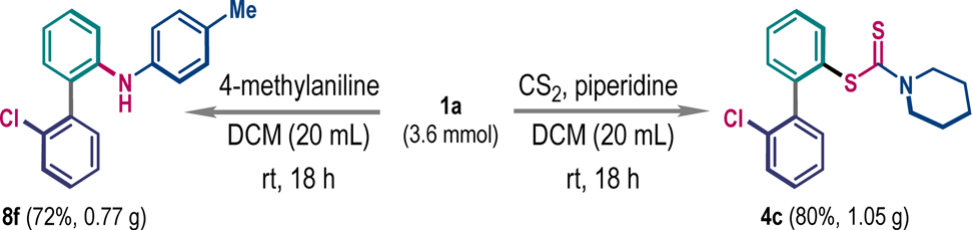
Gram scale synthesis.

To showcase the synthetic utility, we have performed the ligand coupling reaction in gram-scale, where the efficacy of the small-scale reaction was retained. A scaled-up three-component reaction produced product 4c in 80% yield (Scheme 6, right). Similarly, the compound 8f was prepared in 72% yield from a 3.6 mmol scaled two-component reaction (Scheme 6, left).

**Scheme 7:**
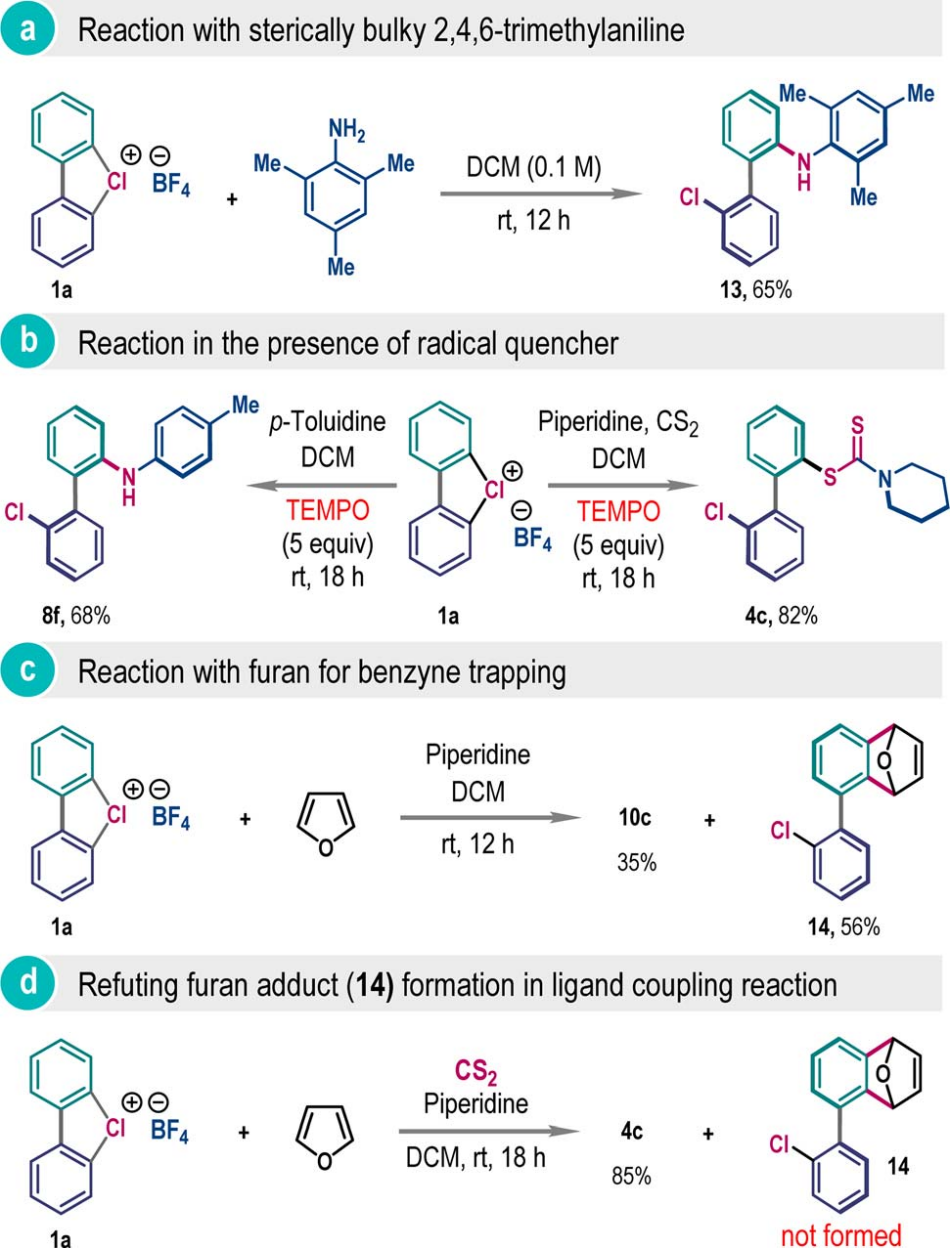
Mechanistic investigations.

Arguably, the formation of 2,2′-biaryl from cyclic λ^3^-chloranes 1 may take place through direct aromatic nucleophilic substitution reaction or *via* ligand association followed by ligand coupling reaction.^6*a*^ To distinguish between these two reaction modes, we have reacted cyclic λ^3^-chlorane 1a with 2,4,6-trimethylaniline, where *ortho*-coupling product 13 was isolated as a sole product in 65% yield (Scheme 7a). Being a sterically bulky weak nucleophile, here, the direct nucleophilic aromatic substitution reaction with 2,4,6-trimethylaniline is very unlikely, and also the absence of the *meta*-product does not support the contribution of the benzyne intermediate.^3*e*^ Further, we have isolated significant amounts of products 4c and 8f in the presence of a radical scavenger TEMPO, refuting the involvement of the radical mechanism for this reaction (Scheme 7b).

The generation of the benzyne intermediate in the presence of a highly basic amine was confirmed by trapping it with furan, dispensing compound 14 in 56% yield (Scheme 7c). However, this adduct formation was not observed when performing the ligand coupling reaction involving 1a, CS_2_, and piperidine in the presence of furan as a trapping reagent, albeit the desired ligand coupling product 4c was obtained in excellent yield (Scheme 7d). All these experiments collectively favor a ligand association followed by ligand coupling pathway for this unsymmetrical biaryl synthesis.

## Conclusions

In conclusion, we have developed an unprecedented ligand coupling reaction of cyclic λ^3^-chloranes under mild conditions. This protocol efficiently suppresses the more facile benzyne formation pathway and selectively promotes the challenging ligand coupling reaction under metal-free conditions, offering a spectrum of unsymmetrical 2,2′-biaryls in very high yields. The protocol operates effectively in both two-component and three-component fashions, is scalable, and smoothly assembles diverse C–S and C–N bonds with exclusive *ortho*-selectivity. Additionally, the use of bicyclic tertiary amines such as DABCO and quinuclidine was achieved by integrating the ligand coupling reaction and subsequent ring opening reaction of the bicyclo[2.2.2]octane framework with a range of nitrogen, sulfur, oxygen, and carbon nucleophiles. Comparative studies of cyclic λ^3^-chloranes with corresponding λ^3^-iodane and λ^3^-bromane compounds demonstrated the superior performance of cyclic λ^3^-chloranes in ligand-coupling reactions under metal-free conditions.

## Author contributions

The manuscript was written through contributions of all authors. All authors have given approval to the final version of the manuscript.

## Conflicts of interest

There are no conflicts to declare.

## Supplementary Material

SC-015-D4SC04108A-s001

SC-015-D4SC04108A-s002

## Data Availability

General information, experimental procedures, characterization data for all new compounds, and NMR spectra are in the ESI.† Data for the crystal structure reported in this paper have been deposited at the Cambridge Crystallographic Data Centre (CCDC) under the deposition number CCDC: 2340860 and 2354443.

## References

[cit1] (a) Olofsson B. , Marek I. and Rappoport Z., The Chemistry of Hypervalent Halogen Compounds, Wiley, 2019, p. 1072; for selected reviews, see

[cit2] (a) Zhdankin V. V. , Hypervalent Iodine Chemistry: Preparation, Structure and Synthetic Applications of Polyvalent Iodine Compounds, Wiley, 2013, p. 468; for selected reviews, see

[cit3] Sandin R. B., Hay A. S. (1952). J. Am. Chem. Soc..

[cit4] Lanzi M., Dherbassy Q., Wencel-Delord J. (2021). Angew. Chem., Int. Ed..

[cit5] Lanzi M., Rogge T., Truong T. S., Houk K. N., Wencel-Delord J. (2023). J. Am. Chem. Soc..

[cit6] Ozanne-Beaudenon A., Quideau S. (2005). Angew. Chem., Int. Ed..

[cit7] Heaney H., Lees P. (1968). Tetrahedron.

[cit8] Patra K. , Dey M. P. and Baidya M., ChemRxiv, 2024, preprint, 10.26434/chemrxiv-2024-sdx0k

[cit9] Kapanda C. N., Masquelier J., Labar G., Muccioli G. G., Poupaert J. H., Lambert D. M. (2012). J. Med. Chem..

[cit10] Baidya M., Kobayashi S., Brotzel F., Schmidhammer U., Riedle E., Mayr H. (2007). Angew. Chem., Int. Ed..

