# Supplementary material for: Metal-free site-selective functionalization with cyclic diaryl λ3-chloranes: suppression of benzyne formation for ligand-coupling reactions
Source: Chem Sci. 2024 Sep 13;15(40):16605–11. doi: 10.1039/d4sc04108a (PMC11414830; doi:10.1039/d4sc04108a)
Supplement: SC-015-D4SC04108A-s001 [file SC-015-D4SC04108A-s001.pdf]

Supplementary Information (SI) for Chemical Science.
This journal is © The Royal Society of Chemistry 2024

- Electronic Supporting Information -

Metal-Free Site-Selective Functionalization with Cyclic Diaryl λ^{3}-Chloranes: Suppression of Benzyne Formation for Ligand-Coupling reactions

Koushik Patra, Manas Pratim Dey, and Mahiuddin Baidya*

Department of Chemistry, Indian Institute of Technology Madras,
Chennai 600 036, Tamil Nadu, India
E-mail: mbaidya@iitm.ac.in

# – Table of Contents –

	Page
• General information	S3
• General procedure for the synthesis of unsymmetrical biaryls (**4/5/6a**)	S4
• Gram scale synthesis of compound **4c**	S4
• General procedure for the synthesis of compounds **8a-8p**	S13
• Gram scale synthesis of compound **8f**	S13
• General procedure for the synthesis of compounds **9a-9d**	S18
• General procedure for the synthesis of compounds **10a-10p**	S19
• General procedure for the synthesis of compounds **11/12**	S23
• Synthesis of compound **13**	S25
• Ligand coupling reaction in the presence of radical quencher	S26
• Aryne trapping experiment	S27
• Investigation of aryne formation during three-component ligand coupling reaction	S27
• Crystallographic experimental section	S28
• NMR spectra of synthesized compounds	S31

General information:

All non-aqueous reactions were carried out under an atmosphere of nitrogen in flame-dried glassware and were stirred using a magnetic stir plate. All reactions were carried out using commercial grade solvent unless otherwise noted. CH_3CN, DCE, and CH_2Cl_2 were dried over calcium hydride. Dry THF was prepared by distilling over sodium ketyl.

All reactions were monitored by thin layer chromatography (TLC) on WhatmanPartisil® K6F TLC plates (silica gel 60 Å, 0.25 mm thickness) and visualized using a UV lamp (366 or 254 nm) or by use of one of the following visualization reagents: PMA: 10 g phosphomolybdic acid/ 100 mL ethanol; $KMnO_4$: 0.75 g potassium permanganate, 5 g K_2CO_3, / 100mL water. Products were isolated by column chromatography (Merck silica gel 100-200μm). Yields refer to chromatographically and spectroscopically homogenous materials unless noted otherwise. ^{13}C and ^{1}H NMR spectra were recorded on a Bruker400 or Bruker 500 MHz spectrometers. Chemical shift values (δ) are reported in ppm and calibrated to the residual solvent peak $CDCl_3$ δ = 7.2600 ppm for ^{1}H, δ = 77.16 for ^{13}C, DMSO-d_6 δ = 2.500 ppm for ^{1}H, δ = 39.500 ppm for ^{13}C; or calibrated to tetramethylsilane (δ = 0.00 ppm). All NMR spectra were recorded at ambient temperature (290 K) unless otherwise noted. ^{1}H NMR spectra are reported as follows: chemical shift (multiplicity, coupling constant, integration). The following abbreviations are used to indicate multiplicities: s, singlet; d, doublet; t, triplet; q, quartet; quint, quintet; sext, sextet; sept, septet; m, multiplet; dd, doublet of doublet; dt, doublet of triplet; dq, doublet of quartet; td, triplet of doublet; tt, triplet of triplet; dq, doublet of quartet; br, broad; app, apparent.

Mass spectra were recorded by electrospray ionization (ESI) method on a Q-TOF Micro with lock spray source. The crystal data were collected and integrated using a BrukerAxs kappa apex2 CCD diffractometer, with graphite monochromated Mo-Kα radiation.

The λ^3-chloranes **1** were synthesized following literature procedures (*J. Am. Chem. Soc*. **2023**, *145*, 345).

General procedure for the synthesis of unsymmetrical biaryls (4/5/6a):

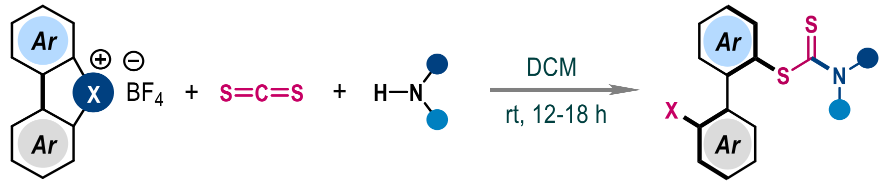

1 2a 3 4/5/6a

A 16x100 mm oven dried reaction tube equipped with a magnetic stir was charged with corresponding λ^3-chlorane/bromane/iodane **1** (0.2 mmol, 1.0 equiv), CS_2 (**2a**, 0.5 mmol, 2.5 equiv), and amine **3** (0.24 mmol, 1.2 equiv) under N_2 atmosphere. Then, dry DCM (1.5 mL) was added via syringe. The reaction mixture was allowed to stir at rt. After completion of the reaction (12–18 h, TLC monitored), volatiles were removed under reduced pressure and the crude product was purified by silica gel column chromatography to provide pure product **4/5.**

Compound **6a** was prepared similarly using λ^3-iodane (**1a"**), CS_2 (**2a**) and pyrrolidine (**3a**) at 50 °C.

To minimize potential contamination from metal impurities, we prepared the chloranes using distilled biaryl precursors (Kugelrohr distillation). Additionally, we conducted two rounds of crystallization on the synthesized λ^3-chloranes before employing them in the reaction.

Gram scale synthesis of compound 4c:

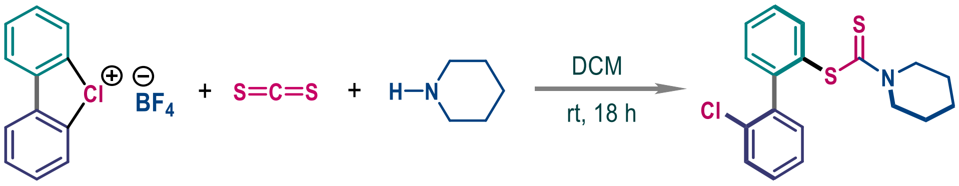

1a 2a 3c 4c

A 100 mL oven dried round bottom flask equipped with a magnetic stir was charged with λ^3-chlorane **1a** (3.64 mmol, 1.0 equiv), CS_2 (**2a**, 2.5 equiv), and piperidine **3c** (1.2 equiv) under N_2 atmosphere. Then, dry DCM (20 mL) was added via syringe. The reaction mixture was allowed to stir at rt for 18 h. After completion, volatiles were removed under reduced pressure and the crude product was purified by silica gel column chromatography to provide pure product **4c** (1.05 g, 80%).

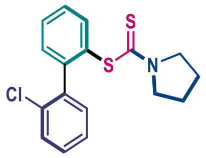

4a

Compound, **4a:** pale yellow sticky liquid; eluent (2% ethyl acetate in hexane). **Yield:** 88% (58 mg); **^{1}H NMR (400 MHz, Chloroform-*d*) δ:** 7.56 (d, *J* = 7.6 Hz, 1H), 7.45 (t, *J* = 7.4 Hz, 1H), 7.41 – 7.37 (m, 2H), 7.33 (d, *J* = 7.6 Hz, 1H), 7.25 (d, *J* = 7.5 Hz, 1H), 7.21 – 7.13 (m, 2H), 3.78 – 3.64 (m, 2H), 3.57 – 3.51 (m, 1H), 3.37 – 3.31 (m, 1H), 1.93 – 1.76 (m, 4H). **^{13}C NMR (101 MHz, Chloroform-*d*) δ:** 192.4, 144.7, 139.5, 138.5, 133.2, 131.3, 130.8, 130.7, 130.2, 128.8, 128.7, 128.6, 126.1, 55.0, 51.1, 26.1, 24.3. **HRMS (ESI/TOF-Q) m/z:** $[M+H]^+$ Calculated for $C_{17}H_{16}ClNS_2H^+$ 334.0485; Found 334.0477.

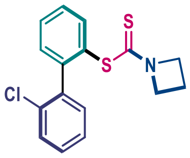

4b

Compound, **4b:** pale yellow sticky liquid; eluent (2% ethyl acetate in hexane). **Yield:** 85% (54 mg); **^{1}H NMR (400 MHz, Chloroform-*d*) δ:** 7.56 – 7.54 (m, 1H), 7.47 – 7.43 (m, 1H), 7.41 – 7.37 (m, 1H), 7.35 – 7.31 (m, 2H), 7.28 – 7.16 (m, 3H), 4.15 – 4.01 (m, 3H), 3.88 – 3.81 (m, 1H), 2.25 – 2.12 (m, 2H). **^{13}C NMR (101 MHz, Chloroform-*d*) δ:**193.7, 144.9, 139.5, 138.4, 133.3, 131.5, 130.8, 130.3, 130.0, 129.0, 128.9, 128.8, 126.3, 54.8, 54.0, 15.0. **HRMS (ESI/TOF-Q) m/z:** $[M+H]^+$ Calculated for $C_{16}H_{14}ClNS_2H^+$ 320.0329; Found 320.0324.

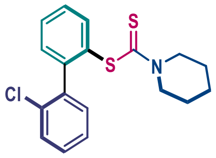

4c

Compound, **4c:** pale yellow sticky liquid; eluent (2% ethyl acetate in hexane). **Yield:** 87% (61 mg); **^{1}H NMR (400 MHz, Chloroform-*d*) δ:** 7.55 (d, *J* = 7.5 Hz, 1H), 7.46 (t, *J* = 7.4 Hz, 1H), 7.41 (d, *J* = 7.5 Hz, 1H), 7.37 – 7.32 (m, 2H), 7.25 (d, *J* = 7.4 Hz, 1H), 7.21 – 7.13 (m, 2H), 4.20 – 4.19 (m, 1H), 3.91 – 3.62 (m, 3H), 1.62 – 1.42 (m, 6H). **^{13}C NMR (101 MHz, Chloroform-*d*) δ:** 195.4, 144.8, 139.6, 138.7, 133.2, 131.4, 131.3, 130.7, 130.3, 128.79, 128.76, 128.7, 126.0, 53.1, 52.3, 25.8, 25.5, 24.2. **HRMS (ESI/TOF-Q) m/z:** $[M+H]^+$ Calculated for $C_{18}H_{18}ClNS_2H^+$ 348.0642; Found 348.0640.

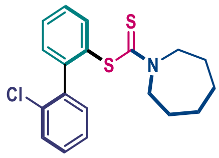

4d

Compound, **4d:** pale yellow sticky liquid; eluent (2% ethyl acetate in hexane). **Yield:** 80% (58 mg); **^{1}H NMR (400 MHz, Chloroform-*d*) δ:** 7.55 (d, *J* = 7.5 Hz, 1H), 7.48 (t, *J* = 7.4 Hz, 1H), 7.44 – 7.40 (m, 1H), 7.37 – 7.32 (m, 2H), 7.27 (d, *J* = 7.6 Hz, 1H), 7.21 – 7.12 (m, 2H), 4.25 – 4.19 (m, 1H), 3.83 – 3.67 (m, 2H), 3.54 – 3.48 (m, 1H), 1.78 – 1.17 (m, 8H). **^{13}C NMR (101 MHz, Chloroform-*d*) δ:** 196.1, 145.1, 139.7, 138.9, 133.3, 131.5, 131.2, 130.7, 130.4, 128.82, 128.78, 128.75, 126.1, 55.8, 53.4, 27.1, 26.4 (2×C), 26.2. **HRMS (ESI/TOF-Q) m/z:** $[M+H]^+$ Calculated for $C_{19}H_{20}ClNS_2H^+$ 362.0798; Found 362.0799.

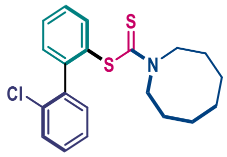

4e

Compound, **4e:** pale yellow solid; eluent (2% ethyl acetate in hexane). **Yield:** 82% (62 mg). MP: 112 ^{o}C; **^{1}H NMR (400 MHz, Chloroform-*d*) δ:** 7.55 (d, *J* = 1.5 Hz, 1H), 7.50 – 7.46 (m, 1H), 7.44 – 7.39 (m, 1H), 7.36 – 7.32 (m, 2H), 7.27 (d, *J* = 5.9 Hz, 1H), 7.20 – 7.11 (m, 2H), 4.21 – 4.14 (m, 1H), 3.82 – 3.76 (m, 1H), 3.67 – 3.61 (m, 1H), 3.49 – 3.43 (m, 1H), 1.85 – 1.78 (m, 1H), 1.72 – 1.51 (m, 3H), 1.37 – 1.22 (m, 6H). **^{13}C NMR (101 MHz, Chloroform-*d*) δ:** 196.2, 145.2, 139.7, 139.0, 133.3, 131.6, 131.2, 130.8, 130.5, 128.9, 128.79, 128.76, 126.2, 56.6, 54.3, 26.8, 26.3, 25.3 (2×C), 25.2. **HRMS (ESI/TOF-Q) m/z:** $[M+H]^+$ Calculated for $C_{20}H_{22}ClNS_2H^+$ 376.0955; Found 376.0952.

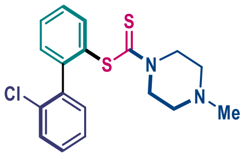

4f

Compound, **4f:** pale yellow sticky liquid; eluent (2% ethyl acetate in hexane). **Yield:** 81% (59 mg); **^{1}H NMR (400 MHz, Chloroform-*d*) δ:** 7.54 (dd, *J* = 7.6, 1.4 Hz, 1H), 7.49 – 7.45 (m, 1H), 7.42 – 7.38 (m, 1H), 7.34 – 7.31 (m, 2H), 7.25 (dd, *J* = 7.5, 1.6 Hz, 1H), 7.22 – 7.13 (m, 2H), 4.20 – 4.02 (m, 2H), 3.76 (bs, 2H), 2.35 – 2.22 (m, 4H), 2.18 (s, 3H). **^{13}C NMR (101 MHz, Chloroform-*d*) δ:** 196.9, 144.9, 139.6, 138.7, 133.4, 131.5, 131.1, 130.8, 130.5, 129.0, 128.90, 128.85, 126.2, 54.4 ((2×C), 51.4, 50.8, 45.6. **HRMS (ESI/TOF-Q) m/z:** $[M+H]^+$ Calculated for $C_{18}H_{19}ClN_2S_2H^+$ 363.0751; Found 363.0752.

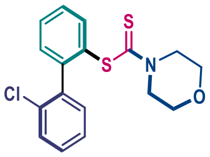

4g

Compound, **4g:** pale yellow sticky liquid; eluent (2% ethyl acetate in hexane). **Yield:** 76% (53 mg); **^{1}H NMR (400 MHz, Chloroform-*d*) δ:** 7.48 (d, *J* = 7.6 Hz, 1H), 7.41 (t, *J* = 7.4 Hz, 1H), 7.34 (t, *J* = 7.5 Hz, 1H), 7.26 (t, *J* = 7.4 Hz, 2H), 7.19 (d, *J* = 7.5 Hz, 1H), 7.20 – 7.07 (m, 2H), 3.98 – 3.36 (m, 8H). **^{13}C NMR (101 MHz, Chloroform-*d*) δ:** 197.3, 144.9, 139.5, 138.5, 133.3, 131.4, 130.8, 130.7, 130.5, 129.0, 128.88, 128.86, 126.1, 66.2, 51.5. **HRMS (ESI/TOF-Q) m/z:** $[M+H]^+$ Calculated for $C_{17}H_{16}ClNOS_2H^+$ 350.0435; Found 350.0436.

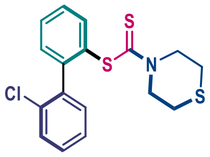

4h

Compound, **4h:** pale yellow sticky liquid; eluent (2% ethyl acetate in hexane). **Yield:** 80% (59 mg); **^{1}H NMR (400 MHz, Chloroform-*d*) δ:** 7.52 (d, *J* = 7.6 Hz, 1H), 7.47 (t, *J* = 7.1 Hz, 1H), 7.42 – 7.38 (m, 1H), 7.33 – 7.29 (m, 2H), 7.26 – 7.13 (m, 3H), 4.60 – 3.90 (m, 4H), 2.67 – 2.07 (m, 4H). **^{13}C NMR (101 MHz, Chloroform-*d*) δ:** 196.8, 145.0, 139.5, 138.6, 133.3, 131.4, 130.84, 130.79, 130.6, 129.0, 128.9 (2×C), 126.0, 54.5, 54.1, 27.0 (2×C). **HRMS (ESI/TOF-Q) m/z:** $[M+H]^+$ Calculated for $C_{17}H_{16}ClNS_3H^+$ 366.0206; Found 366.0208.

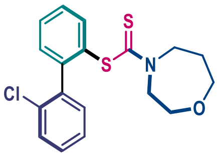

4i

Compound, **4i:** pale yellow sticky liquid; eluent (2% ethyl acetate in hexane). **Yield:** 84% (61 mg); **^{1}H NMR (400 MHz, Chloroform-*d*) δ:** 7.58 – 7.54 (m, 1H), 7.54 – 7.50 (m, 1H), 7.47 – 7.43 (m, 1H), 7.38 – 7.34 (m, 2H), 7.30 (d, *J* = 7.5 Hz, 1H), 7.26 – 7.16 (m, 2H), 4.49 – 4.30 (m, 1H), 4.01 – 3.83 (m, 2H), 3.75 – 3.24 (m, 4H), 3.23 – 3.02 (m, 1H), 2.03 – 1.67 (m, 2H). **^{13}C NMR (101 MHz, Chloroform-*d*) δ:** 197.5, 196.9, 145.12, 145.09, 139.7, 139.6, 138.79, 138.75, 133.4, 131.6, 131.5, 130.88, 130.85, 130.7, 129.04, 128.97, 128.90, 128.88, 128.8, 126.1, 70.1, 69.8, 69.5, 69.3, 58.6, 56.4, 53.7, 51.3, 27.8, 27.0. **HRMS (ESI/TOF-Q) m/z:** $[M+H]^+$ Calculated for $C_{18}H_{18}ClNOS_2H^+$ 364.0591; Found 364.0598.

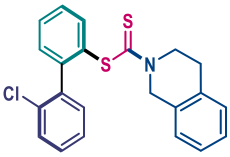

4j

Compound, **4j:** pale yellow sticky liquid; eluent (2% ethyl acetate in hexane). **Yield:** 82% (65 mg); **^{1}H NMR (400 MHz, Chloroform-*d*) δ:** 7.60 (d, *J* = 7.6 Hz, 1H), 7.51 (t, *J* = 7.5 Hz, 1H), 7.44 (t, *J* = 7.5 Hz, 1H), 7.37 – 7.29 (m, 3H), 7.24 – 7.01 (m, 6H), 5.25 – 4.76 (m, 2H), 4.26 – 3.90 (m, 2H), 2.97 – 2.73 (m, 2H). **^{13}C NMR (101 MHz, Chloroform-*d*) δ:** 196.5, 144.9, 139.5, 138.6, 135.3, 133.8, 133.2, 132.0, 131.3, 130.8, 130.4, 130.2, 129.6, 129.5, 129.0, 128.9, 128.8, 127.9, 127.4, 127.2, 126.8, 126.6, 126.1, 53.9, 52.2, 50.0, 48.7, 31.6, 29.1. **HRMS (ESI/TOF-Q) m/z:** $[M+H]^+$ Calculated for $C_{22}H_{18}ClNS_2H^+$ 396.0642; Found 396.0652.

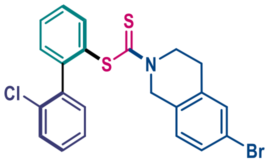

4k

Compound, **4k:** pale yellow sticky liquid; eluent (2% ethyl acetate in hexane). **Yield:** 80% (76 mg); **^{1}H NMR (400 MHz, Chloroform-*d*) δ:** 7.64 (d, *J* = 7.5 Hz, 1H), 7.59 – 7.48 (m, 2H), 7.42 – 7.31 (m, 5H), 7.26 – 6.94 (m, 3H), 5.14 – 4.81 (m, 2H), 4.35 – 3.94 (m, 2H), 2.99 – 2.75 (m, 2H). **^{13}C NMR (101 MHz, Chloroform-*d*) δ:** 196.8, 144.9, 139.4, 138.6, 138.5, 133.2, 131.3, 130.9, 130.5, 129.9, 129.8, 128.93, 128.85, 128.8, 128.3, 126.6, 126.1, 120.8, 53.3, 51.8, 49.4, 48.2, 31.6, 28.8. **HRMS (ESI/TOF-Q) m/z:** $[M+H]^+$ Calculated for $C_{22}H_{17}Br^{81}ClNS_2H^+$ 475.9747; Found 475.9742.

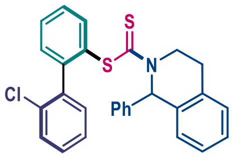

4l

Compound, **4l:** pale yellow sticky liquid; eluent (2% ethyl acetate in hexane). **Yield:** 68% (1.5:1 dr) (64 mg); **^{1}H NMR (400 MHz, Chloroform-*d*) δ:** 7.96 – 7.88 (m, 1.51H), 7.73 – 7.69 (m, 1.20H), 7.63 – 7.47 (m, 7.88H), 7.45 – 7.11 (m, 28.33H), 7.04 – 6.96 (m, 2.12H), 6.91 – 6.78 (m, 2H), 6.75 – 6.68 (m, 1.46H), 5.00 – 4.83 (m, 1H), 4.22 – 4.06 (m, 1.46H), 3.61 – 3.41 (m, 2.44H), 3.12 – 2.90 (m, 2.13H), 2.74 – 2.53 (m, 3.04H). **^{13}C NMR (101 MHz, Chloroform-*d*) δ:** 197.34, 197.29, 196.2, 195.9, 145.2, 144.9, 140.5, 139.9, 139.6, 138.9, 138.8, 138.7, 135.3, 134.6, 133.20, 133.15, 131.5, 131.4, 131.3, 130.9, 130.8, 130.7, 130.5, 129.0, 128.92, 128.87, 128.7, 128.5, 128.3, 128.2, 128.0, 127.8, 127.6, 127.53, 127.47, 126.6, 126.4, 126.0, 65.6, 64.5, 64.3, 64.2, 47.0, 45.8, 45.2, 28.5, 28.2, 27.7. **HRMS (ESI/TOF-Q) m/z:** $[M+H]^+$ Calculated for $C_{28}H_{22}ClNS_2H^+$ 472.0955; Found 472.0955.

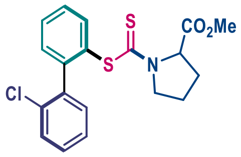

4m

Compound, **4m:** pale yellow sticky liquid; eluent (2% ethyl acetate in hexane). **Yield:** 76% (3:1 dr) (60 mg); **^{1}H NMR (400 MHz, Chloroform-*d*) δ:** 7.58 – 7.52 (m, 4.77H), 7.49 – 7.26 (m, 18.52H), 7.24 – 7.11 (m, 8.46H), 4.88 – 4.78 (m, 3.05H), 4.64 – 4.40 (m, 1H), 3.97 – 3.56 (m, 18.46H), 3.48 – 3.39 (m, 2.61H), 2.39 – 1.75 (m, 15.49H). **^{13}C NMR (101 MHz, Chloroform-*d*) δ:** 195.13, 195.10, 194.4, 171.1, 170.9, 170.8, 170.5, 144.8, 144.7, 139.6, 139.3, 138.7, 138.6, 138.5, 138.4, 133.4, 133.1, 133.0, 132.9, 131.6, 131.4, 131.0, 130.74, 130.69, 130.6, 130.4, 130.3, 128.99, 128.95, 128.9, 128.82, 128.76, 128.7, 126.4, 126.20, 126.18, 126.1, 66.4, 66.2, 63.3, 62.8, 55.4, 55.2, 52.9, 52.6, 52.4, 52.3, 51.5, 51.4, 31.8, 31.5, 29.32, 29.27, 24.8, 22.6, 22.5. **HRMS (ESI/TOF-Q) m/z:** $[M+H]^+$ Calculated for $C_{19}H_{18}ClNO_2S_2H^+$ 392.0540; Found 392.0546.

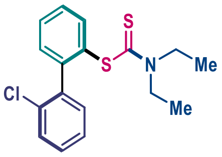

4n

Compound, **4n:** pale yellow sticky liquid; eluent (2% ethyl acetate in hexane). **Yield:** 72% (48 mg); **^{1}H NMR (400 MHz, Chloroform-*d*) δ:** 7.65 – 7.45 (m, 3H), 7.43 – 7.37 (m, 2H), 7.36 – 7.31 (m, 1H), 7.30 – 7.28 (m, 1H), 7.24 – 7.19 (m, 1H), 4.01 – 3.86 (m, 2H), 3.73 – 3.49 (m, 2H), 1.35 – 1.05 (m, 6H). **^{13}C NMR (101 MHz, Chloroform-*d*) δ:** 195.6, 145.0, 139.7, 138.9, 133.3, 131.4, 131.3, 130.8, 130.4, 128.9, 128.84, 128.80, 126.1, 49.8, 47.4, 12.6, 11.7. **HRMS (ESI/TOF-Q) m/z:** $[M+H]^+$ Calculated for $C_{17}H_{18}ClNS_2H^+$ 336.0642; Found 336.0647.

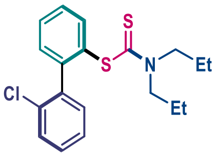

4o

Compound, **4o:** pale yellow sticky liquid; eluent (2% ethyl acetate in hexane). **Yield:** 74% (54 mg); **^{1}H NMR (400 MHz, Chloroform-*d*) δ:** 7.63 (d, *J* = 7.5 Hz, 1H), 7.56 (t, *J* = 7.6 Hz, 1H), 7.51 – 7.47 (m, 1H), 7.43 – 7.33 (m, 3H), 7.30 – 7.18 (m, 2H), 3.96 – 3.38 (m, 4H), 1.80 – 1.41 (m, 4H), 0.97 – 0.79 (m, 6H). **^{13}C NMR (101 MHz, Chloroform-*d*) δ:** 196.2, 145.0, 139.7, 138.9, 133.3, 131.40, 131.35, 130.8, 130.4, 128.82, 128.78 (2×C), 126.1, 56.9, 54.8, 20.9, 19.7, 11.3, 11.2. **HRMS (ESI/TOF-Q) m/z:** $[M+H]^+$ Calculated for $C_{19}H_{22}ClNS_2H^+$ 364.0955; Found 364.0957.

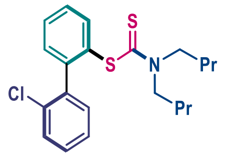

4p

Compound, **4p:** pale yellow sticky liquid; eluent (2% ethyl acetate in hexane). **Yield:** 83% (65 mg); **^{1}H NMR (400 MHz, Chloroform-*d*) δ:** 7.70 – 7.62 (m, 1H), 7.57 – 7.47 (m, 2H), 7.42 – 7.28 (m, 3H), 7.22 – 7.13 (m, 2H), 3.96 – 3.37 (m, 4H), 1.73 – 1.16 (m, 8H), 0.97 – 0.86 (m, 6H). **^{13}C NMR (101 MHz, Chloroform-*d*) δ:** 195.9, 145.1, 139.7, 138.9, 133.3, 131.4, 131.3, 130.7, 130.4, 128.81 (2×C), 128.79, 126.1, 55.1, 53.0, 29.5, 28.5, 20.1, 20.0, 14.0, 13.8. **HRMS (ESI/TOF-Q) m/z:** $[M+H]^+$ Calculated for $C_{21}H_{26}ClNS_2H^+$ 392.1268; Found 392.1266.

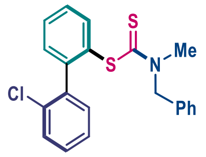

4q

Compound, **4q:** pale yellow sticky liquid; eluent (2% ethyl acetate in hexane). **Yield:** 70% (56 mg); **^{1}H NMR (400 MHz, Chloroform-*d*) δ:** 7.57 – 7.14 (m, 11H), 6.99 – 6.83 (m, 2H), 5.44 – 4.52 (m, 2H), 3.20 – 3.02 (m, 3H). **^{13}C NMR (101 MHz, Chloroform-*d*) δ:** 198.9, 197.5, 145.2, 145.1, 139.6, 138.8, 138.6, 135.6, 134.7, 133.4, 131.5, 131.4, 130.9, 130.6, 128.9, 128.8, 128.0, 127.7, 127.6, 127.3, 126.2, 126.1, 59.6, 58.2, 43.2, 39.4. **HRMS (ESI/TOF-Q) m/z:** $[M+Na]^+$ Calculated for $C_{21}H_{18}ClNS_2Na^+$ 406.0461; Found 406.0465.

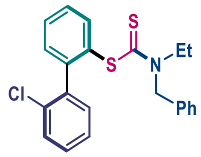

4r

Compound, **4r:** pale yellow sticky liquid; eluent (2% ethyl acetate in hexane). **Yield:** 75% (60 mg); **^{1}H NMR (400 MHz, Chloroform-*d*) δ:** 7.54 – 7.09 (m, 11H), 6.96 – 6.82 (m, 2H), 5.28 – 4.44 (m, 2H), 3.83 – 3.60 (m, 1H), 3.45 – 3.36 (m, 1H), 0.99 – 0.90 (m, 3H).**^{13}C NMR (101 MHz, Chloroform-*d*) δ:** 198.1, 196.9, 145.1, 139.7, 138.8, 135.9, 133.3, 131.5, 131.2, 130.8, 130.6, 128.91, 128.87, 128.8, 128.6, 127.6, 127.3, 126.1, 57.0, 55.4, 49.4, 46.8, 12.2, 11.2. **HRMS (ESI/TOF-Q) m/z:** $[M+H]^+$ Calculated for $C_{22}H_{20}ClNS_2H^+$ 398.0798; Found 398.0791.

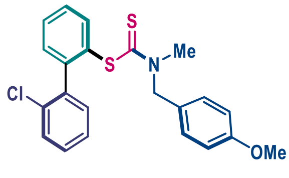

4s

Compound, **4s:** pale yellow sticky liquid; eluent (2% ethyl acetate in hexane). **Yield:** 78% (65 mg); **^{1}H NMR (400 MHz, Chloroform-*d*) δ:** 7.70 – 6.78 (m, 12H), 5.42 – 4.55 (m, 2H), 3.81 (s, 3H), 3.28 – 3.10 (m, 3H). **^{13}C NMR (101 MHz, Chloroform-*d*) δ:** 198.6, 197.2, 159.4, 159.3, 145.2, 145.1, 139.8, 139.7, 138.9, 138.7, 133.9, 133.4, 131.7, 131.5, 131.4, 130.9, 130.6, 130.08, 130.06, 129.7, 129.58, 129.55, 129.1, 129.0, 128.9, 128.8, 127.5, 127.2, 126.8, 126.7, 126.6, 126.3, 126.1, 114.3, 114.2, 59.1, 57.7, 55.4, 43.0, 39.2. **HRMS (ESI/TOF-Q) m/z:** $[M+H]^+$ Calculated for $C_{22}H_{20}ClNOS_2H^+$ 414.0748; Found 414.0754.

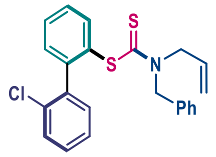

4t

Compound, **4t:** pale yellow sticky liquid; eluent (2% ethyl acetate in hexane). **Yield:** 70% (57 mg); **^{1}H NMR (400 MHz, Chloroform-*d*) δ:** 7.68 – 7.28 (m, 11H), 7.20 – 6.98 (m, 2H), 5.79 – 5.57 (m, 1H), 5.37 – 4.90 (m, 3H), 4.95 – 4.31 (m, 2H), 4.17 – 4.03 (m, 1H). **^{13}C NMR (101 MHz, Chloroform-*d*) δ:** 199.1, 198.3, 145.2, 139.7, 138.8, 135.7, 134.8, 133.4, 131.7, 131.5, 131.4, 131.2, 130.9, 130.7, 130.2, 130.1, 129.7, 129.6, 129.0, 128.94, 128.91, 128.7, 127.8, 127.7, 127.4, 126.7, 126.2, 126.1, 118.6, 118.4, 56.7, 56.2, 54.9, 53.8. **HRMS (ESI/TOF-Q) m/z:** $[M+H]^+$ Calculated for $C_{23}H_{20}ClNS_2H^+$ 410.0798; Found 410.0797.

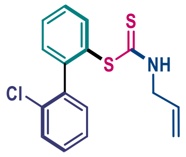

4u

Compound, **4u:** pale yellow sticky liquid; eluent (2% ethyl acetate in hexane). **Yield:** 76% (49 mg); **^{1}H NMR (400 MHz, Chloroform-*d*) δ**: 7.73 (d, *J* = 7.5 Hz, 1H), 7.62 – 7.58 (m, 1H), 7.55 – 7.51 (m, 1H), 7.47 – 7.43 (m, 2H), 7.35 – 7.28 (m, 2H), 7.20 (d, *J* = 7.1 Hz, 1H), 6.67 (s, 1H), 5.78 – 5.69 (m, 1H), 5.12 (d, *J* = 10.3 Hz, 1H), 5.05 (d, *J* = 17.2 Hz, 1H), 4.32 – 4.25 (m, 1H), 4.13 – 4.06 (m, 1H). **^{13}C NMR (101 MHz, Chloroform-*d*) δ**: 194.7, 144.8, 138.3, 137.1, 132.9, 132.4, 131.7, 131.4 (2×C), 129.9, 129.8, 129.7, 128.4, 126.8, 118.1, 48.6. **HRMS (ESI/TOF-Q) m/z:** $[M+H]^+$ Calculated for $C_{16}H_{14}ClNS_2H^+$ 320.0329; Found 320.0332.

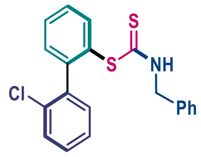

4v

Compound, **4v:** pale yellow sticky liquid; eluent (2% ethyl acetate in hexane). **Yield:** 79% (59 mg); **^{1}H NMR (400 MHz, Chloroform-*d*) δ:** 7.65 (d, *J* = 7.5 Hz, 1H), 7.50 – 7.40 (m, 2H), 7.37 – 7.32 (m, 2H), 7.30 – 7.18 (m, 5H), 7.07 – 7.01 (m, 3H), 6.79 (s, 1H), 4.86 (dd, *J* = 15.0, 6.1 Hz, 1H), 4.57 (dd, *J* = 14.9, 5.0 Hz, 1H). **^{13}C NMR (101 MHz, Chloroform-*d*) δ**: 194.9, 144.7, 138.3, 137.1, 136.0, 132.9, 132.3, 131.4, 131.3, 129.8, 129.69, 129.66, 128.9, 128.1, 127.8, 126.7, 125.6, 50.2. **HRMS (ESI/TOF-Q) m/z:** $[M+H]^+$ Calculated for $C_{20}H_{16}ClNS_2H^+$ 370.0485; Found 370.0485.

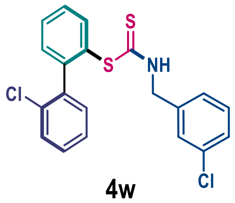

4w

Compound, **4w:** pale yellow sticky liquid; eluent (2% ethyl acetate in hexane). **Yield:** 71% (57 mg); **^{1}H NMR (400 MHz, Chloroform-*d*) δ:** 7.73 (d, *J* = 7.6 Hz, 1H), 7.60 – 7.55 (m, 1H), 7.54 – 7.49 (m, 1H), 7.43 – 7.41 (m, 2H), 7.36 – 7.28 (m, 2H), 7.25 – 7.20 (m, 2H), 7.15 – 7.13 (m, 1H), 7.09 (s, 1H), 7.01 (d, *J* = 7.0 Hz, 1H), 6.88 (s, 1H), 4.90 – 4.85 (m, 1H), 4.70 – 4.62 (m, 1H). **^{13}C NMR (101 MHz, Chloroform-*d*) δ**: 195.4, 144.7, 138.2, 138.1, 137.1, 134.7, 132.8, 132.4, 131.5, 131.3, 130.1, 130.0, 129.8, 129.7, 128.22, 128.19, 127.8, 126.8, 125.9, 49.3. **HRMS (ESI/TOF-Q) m/z:** $[M+H]^+$ Calculated for $C_{20}H_{15}Cl_2NS_2H^+$ 404.0096; Found 404.0095.

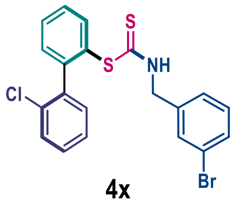

4x

Compound, **4x:** pale yellow sticky liquid; eluent (2% ethyl acetate in hexane). **Yield:** 73% (65 mg); **^{1}H NMR (400 MHz, Chloroform-*d*) δ:** 7.59 – 7.57 (m, 1H), 7.47 – 7.35 (m, 2H), 7.28 – 7.24 (m, 2H), 7.21 – 7.14 (m, 2H), 7.14 – 7.10 (m, 2H), 7.04 – 6.98 (m, 2H), 6.91 (d, *J* = 7.7 Hz, 1H), 6.75 – 6.72 (m, 1H), 4.74 – 4.69 (m, 1H), 4.54 – 4.46 (m, 1H). **^{13}C NMR (101 MHz, Chloroform-*d*) δ**: 195.4, 144.7, 138.4, 138.1, 137.1, 132.8, 132.4, 131.5, 131.3, 131.1, 130.6, 130.4, 130.0, 129.8, 129.7, 128.2, 126.8, 126.4, 122.8, 49.2. **HRMS (ESI/TOF-Q) m/z:** $[M+H]^+$ Calculated for $C_{20}H_{15}BrClNS_2H^+$ 447.9591; Found 447.9591.

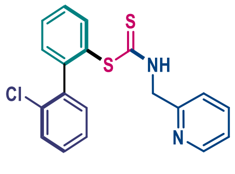

4y

Compound, **4y:** pale yellow sticky liquid; eluent (2% ethyl acetate in hexane). **Yield:** 89% (66 mg); **^{1}H NMR (400 MHz, Chloroform-*d*) δ:** 8.36 (s, 1H), 8.24 (d, *J* = 4.8 Hz, 1H), 7.73 (d, *J* = 7.5 Hz, 1H), 7.62 – 7.57 (m, 2H), 7.51 (t, *J* = 7.6 Hz, 1H), 7.41 (d, *J* = 7.7 Hz, 1H), 7.37 (d, *J* = 8.1 Hz, 1H), 7.24 – 7.22 (m, 1H), 7.18 – 7.11 (m, 4H), 4.83 – 4.77 (m, 1H), 4.72 – 4.67 (m, 1H). **^{13}C NMR (101 MHz, Chloroform-*d*) δ:** 194.7, 154.0, 148.7, 145.0, 138.8, 137.3, 137.0, 133.3, 131.9, 131.2, 130.9, 129.8, 129.6, 129.5, 128.6, 126.6, 122.7, 122.1, 50.2. **HRMS (ESI/TOF-Q) m/z:** $[M+H]^+$ Calculated for $C_{19}H_{15}ClN_2S_2H^+$ 371.0438; Found 371.0446.

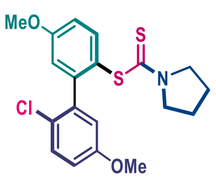

4z

Compound, **4z:** pale yellow sticky liquid; eluent (4% ethyl acetate in hexane). **Yield:** 69% (45 mg); **^{1}H NMR (400 MHz, Chloroform-*d*) δ:** 7.51 (d, J = 8.5 Hz, 1H), 7.29 – 7.27 (m, 1H), 7.09 (d, J = 3.0 Hz, 1H), 7.01 (dd, J = 8.6, 2.7 Hz, 1H), 6.88 (d, J = 2.8 Hz, 1H), 6.83 (dd, J = 8.7, 2.9 Hz, 1H), 3.86 (s, 3H), 3.82 – 3.76 (m, 2H), 3.73 (s, 3H), 3.68 – 3.61 (m, 1H), 3.50 – 3.44 (m, 1H), 2.07 – 1.97 (m, 2H), 1.97 – 1.89 (m, 2H). **^{13}C NMR (126 MHz, Chloroform-*d*) δ:** 194.2, 161.1, 157.6, 146.5, 140.4, 140.1, 129.6, 124.4, 121.6, 116.3, 116.2, 115.5, 114.8, 55.9, 55.5, 55.3, 51.2, 26.3, 24.5. **HRMS (ESI/TOF-Q) m/z:** [M+H]$^{+}$ Calculated for $C_{19}H_{20}ClNO_2S_2H^{+}$ 394.0697; Found 394.0696.

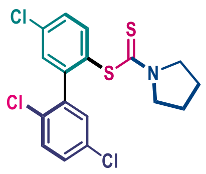

4aa

Compound, **4aa**: pale yellow sticky liquid; eluent (4% ethyl acetate in hexane). **Yield:** 78% (50 mg); **^{1}H NMR (400 MHz, Chloroform-*d*) δ:** 7.56 (d, J = 8.4 Hz, 1H), 7.48 – 7.41 (m, 2H), 7.38 – 7.28 (m, 3H), 3.82 (t, J = 7.2 Hz, 2H), 3.65 – 3.59 (m, 1H), 3.48 – 3.42 (m, 1H), 2.04 – 1.90 (m, 4H). **^{13}C NMR (101 MHz, Chloroform-*d*) δ:** 191.5, 145.0, 140.0, 139.9, 136.7, 132.2, 131.8, 131.2, 130.7, 130.1, 129.6, 129.5, 129.4, 55.3, 51.4, 26.3, 24.5. **HRMS** (ESI/TOF-Q) m/z: [M+H]$^{+}$ Calculated for $C_{17}H_{14}Cl_3NS_2H^{+}$ 401.9706; Found 401.9702.

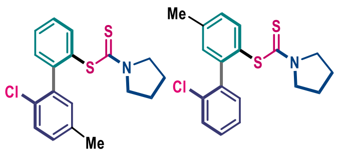

4ab+4ab'

Compound, **4ab+4ab′:** pale yellow sticky liquid; eluent (3% ethyl acetate in hexane). **Yield:** 74% (2:1 rr) (52 mg); **^{1}H NMR (400 MHz, Chloroform-*d*) δ:** 7.63 – 7.61 (m, 2H), 7.53 – 7.38 (m, 7H), 7.31 – 7.23 (m, 9H), 7.16 – 7.12 (m, 1H), 7.08 – 7.05 (m, 2H), 3.86 – 3.79 (m, 3H), 3.78 – 3.71 (m, 3H), 3.64 – 3.58 (m, 3H), 3.47 – 3.39 (m, 3H), 2.42 (s, 3H), 2.28 (s, 6H), 2.02 – 1.85 (m, 12H). **^{13}C NMR (101 MHz, Chloroform-*d*) δ:** 193.2, 192.7, 144.9, 144.7, 140.6, 139.8, 139.3, 138.5, 138.4, 135.9, 133.3, 132.1, 131.6, 131.5, 131.0, 130.7, 130.3, 130.2, 129.7, 129.6, 128.8, 128.6, 128.5, 127.5, 126.2, 55.1, 55.1, 51.3, 51.2, 26.2, 24.4, 24.4, 21.6, 20.9. **HRMS (ESI/TOF-Q) m/z:** [M+H]$^{+}$ Calculated for $C_{18}H_{18}ClNS_2H^{+}$ 348.0642; Found 348.0640.

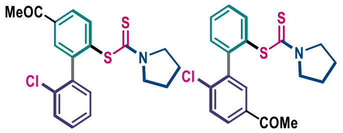

4ac+4ac'

Compound, **4ac+4ac′:** pale yellow sticky liquid; eluent (3% ethyl acetate in hexane). **Yield:** 73% (6:1 rr) (54 mg); **^{1}H NMR (400 MHz, Chloroform-*d*) δ:** 8.12 (d, *J* = 2.2 Hz, 1H), 8.03 (dd, *J* = 8.1, 2.0 Hz, 6H), 7.93 – 7.90 (m, 7H), 7.75 (d, *J* = 8.1 Hz, 6H), 7.65 (dd, *J* = 7.6, 1.5 Hz, 1H), 7.61 – 7.57 (m, 1H), 7.54 – 7.50 (m, 2H), 7.45 – 7.42 (m, 12H), 7.38 (dd, *J* = 7.5, 1.7 Hz, 1H), 7.43 – 7.25 (m, 12H), 3.86 – 3.70 (m, 14H), 3.67 – 3.60 (m, 7H), 3.49 – 3.39 (m, 7H), 2.64 (s, 18H), 2.53 (s, 3H), 2.09 – 1.87 (m, 28H). **^{13}C NMR (101 MHz, Chloroform-*d*) δ:** 197.4, 191.2, 145.0, 138.9, 138.9, 138.8, 138.1, 136.6, 133.4, 132.4, 131.5, 130.8, 130.6, 129.5, 129.4, 129.4, 129.1, 128.3, 128.2, 126.5, 55.2, 55.2, 51.5, 51.3, 26.9, 26.3, 24.5, 24.5. **HRMS (ESI/TOF-Q) m/z:** [M+H]$^{+}$ Calculated for $C_{19}H_{18}ClNOS_2H^{+}$ 376.0591; Found 376.0587.

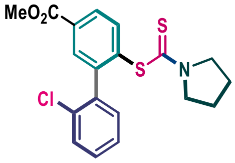

4ad

Compound, **4ad:** pale yellow sticky liquid; eluent (3% ethyl acetate in hexane). **Yield:** 71% (>20:1 rr) (55 mg); **^{1}H NMR (400 MHz, Chloroform-*d*) δ:** 8.12 (dd, *J* = 8.1, 1.9 Hz, 1H), 8.00 (d, *J* = 1.9 Hz, 1H), 7.72 (d, *J* = 8.1 Hz, 1H), 7.44 – 7.41 (m, 2H), 7.33 – 7.24 (m, 2H), 3.92 (s, 3H), 3.85 – 3.72 (m, 2H), 3.67 – 3.60 (m, 1H), 3.49 – 3.42 (m, 1H), 2.07 – 1.97 (m, 2H), 1.96 – 1.88 (m, 2H). **^{13}C NMR (101 MHz, Chloroform-*d*) δ:** 191.3, 166.5, 144.9, 138.9, 138.6, 136.4, 133.4, 131.9, 131.7, 131.5, 129.6, 129.3, 129.0, 126.4, 55.2, 52.5, 51.5, 26.3, 24.5. **HRMS (ESI/TOF-Q) m/z:** [M+H]$^{+}$ Calculated for $C_{19}H_{18}ClNO_2S_2H^{+}$ 392.0540; Found 392.0543.

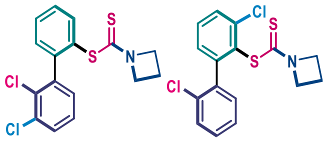

4ae+4ae'

Compound, **4ae+4ae′:** pale yellow sticky liquid; eluent (3% ethyl acetate in hexane). **Yield:** 72% (1:1) (45 mg); **^{1}H NMR (400 MHz, Chloroform-*d*) δ:**7.64 – 7.41 (m, 7H), 7.35 – 7.28 (m, 4H), 7.24 – 7.18 (m, 3H), 4.30 – 4.11 (m, 6H), 4.02 – 3.93 (m, 2H), 2.37 – 2.21 (m, 4H). **^{13}C NMR (101 MHz, Chloroform-*d*) δ:** 193.6, 191.4, 147.6, 144.8, 141.7, 141.6, 139.6, 138.6, 133.2, 132.7, 131.9, 131.3, 131.2, 130.6, 130.5, 130.1, 129.9, 129.81, 129.78, 129.7, 129.32, 129.28, 129.2, 129.0, 126.9, 126.4, 54.9 (2×C), 54.1, 54.0, 15.1, 15.0. **HRMS (ESI/TOF-Q) m/z:** [M+H]$^{+}$ Calculated for $C_{16}H_{13}Cl_2NS_2H^{+}$ 353.9939; Found 353.9947.

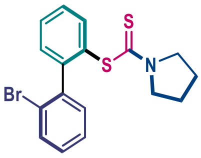

5a

Compound, **5a:** pale yellow sticky liquid; eluent (2% ethyl acetate in hexane). **Yield:** 69% (52 mg); **^{1}H NMR (400 MHz, Chloroform-*d*) δ:** 7.61 – 7.52 (m, 2H), 7.46 (t, J = 7.4 Hz, 1H), 7.41 (d, J = 7.4 Hz, 1H), 7.37 (d, J = 8.0, 1H), 7.24 – 7.16 (m, 2H), 7.14 – 7.07 (m, 1H), 3.79 – 3.65 (m, 2H), 3.58 – 3.52 (m, 1H), 3.39 – 3.32 (m, 1H), 1.95 – 1.80 (m, 4H). **^{13}C NMR (101 MHz, Chloroform-*d*) δ:** 192.6, 146.5, 141.7, 138.6, 132.0, 131.3, 130.74, 130.70, 130.3, 129.1, 128.8, 126.8, 123.8, 55.1, 51.3, 26.3, 24.4. **HRMS (ESI/TOF-Q) m/z:** [M+H]$^{+}$ Calculated for $C_{17}H_{16}Br^{81}NS_2H^{+}$ 379.9980; Found 379.9976.

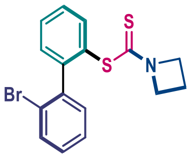

5b

Compound, **5b:** pale yellow sticky liquid; eluent (2% ethyl acetate in hexane). **Yield:** 67% (49 mg); **^{1}H NMR (400 MHz, Chloroform-*d*) δ:** 7.68 – 7.61 (m, 2H), 7.58 – 7.52 (m, 1H), 7.50 – 7.46 (m, 1H), 7.41 (dd, J = 7.6, 1.8 Hz, 1H), 7.37 – 7.28 (m, 2H), 7.24 – 7.20 (m, 1H), 4.24 – 4.07 (m, 3H), 3.97 – 3.90 (m, 1H), 2.43 – 2.18 (m, 2H). **^{13}C NMR (101 MHz, Chloroform-*d*) δ:** 193.7, 146.6, 141.5, 138.4, 132.1, 131.4, 130.7, 130.4, 129.9, 129.2, 128.9, 126.9, 123.8, 54.8, 54.0, 15.1. **HRMS (ESI/TOF-Q) m/z:** [M+H]$^{+}$ Calculated for $C_{16}H_{14}BrNS_2H^{+}$ 363.9824; Found 363.9829.

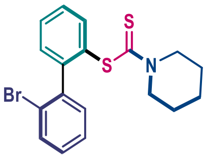

5c

Compound, **5c:** pale yellow sticky liquid; eluent (2% ethyl acetate in hexane). **Yield:** 71% (56 mg); **^{1}H NMR (400 MHz, Chloroform-*d*) δ:** 7.55 (t, J = 8.3 Hz, 2H), 7.51 – 7.47 (m, 1H), 7.45 – 7.30 (m, 2H), 7.28 – 7.20 (m, 2H), 7.16 – 7.09 (m, 1H), 4.21 – 3.64 (m, 4H), 1.65 – 1.44 (m, 6H). **^{13}C NMR (101 MHz, Chloroform-*d*) δ:** 195.5, 146.6, 141.7, 138.7, 132.0, 131.4, 131.2, 130.7, 130.3, 129.0, 128.8, 126.7, 123.8, 53.2, 52.4, 25.8, 25.6, 24.3. **HRMS (ESI/TOF-Q) m/z:** [M+H]$^{+}$ Calculated for $C_{18}H_{18}BrNS_2H^{+}$ 392.0137; Found 392.0140.

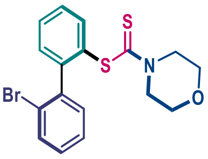

5d

Compound, **5d:** pale yellow sticky liquid; eluent (2% ethyl acetate in hexane). **Yield:** 65% (51 mg); **^{1}H NMR (400 MHz, Chloroform-*d*) δ:**7.65 – 7.53 (m, 2H), 7.51 – 7.47 (m, 1H), 7.45 – 7.39 (m, 1H), 7.34 – 7.30 (m, 1H), 7.26 – 7.19 (m, 2H), 7.16 – 7.09 (m, 1H), 3.81 – 3.31 (m, 8H). **^{13}C NMR (101 MHz, Chloroform-*d*) δ:** 197.4, 146.6, 141.5, 138.6, 132.0, 131.3, 130.8, 130.60, 130.57, 129.2, 128.9, 126.7, 123.8, 66.3, 51.5. **HRMS (ESI/TOF-Q) m/z:** [M+H]$^{+}$ Calculated for $C_{17}H_{16}BrNOS_2H^{+}$ 393.9929; Found 393.9932.

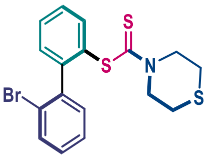

5e

Compound, **5e:** pale yellow sticky liquid; eluent (2% ethyl acetate in hexane). **Yield:** 68% (56 mg); **^{1}H NMR (400 MHz, Chloroform-*d*) δ:** 7.55 – 7.48 (m, 3H), 7.45 – 7.40 (m, 1H), 7.34 – 7.31 (m, 1H), 7.26 – 7.20 (m, 2H), 7.18 – 7.13 (m, 1H), 4.63 – 3.91 (m, 4H), 2.68 – 2.09 (m, 4H). **^{13}C NMR (101 MHz, Chloroform-*d*) δ:** 196.8, 146.6, 141.6, 138.7, 132.3, 131.3, 130.8, 130.73, 130.69, 129.2, 128.9, 126.6, 123.9, 54.7, 54.4, 27.1 (2×C). **HRMS (ESI/TOF-Q) m/z:** [M+H]$^{+}$ Calculated for $C_{17}H_{16}BrNS_3H^{+}$ 409.9701; Found 409.9705.

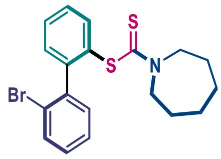

5f

Compound, **5f:** pale yellow sticky liquid; eluent (2% ethyl acetate in hexane). **Yield:** 72% (59 mg); **^{1}H NMR (400 MHz, Chloroform-*d*) δ:** 7.54 (t, *J* = 8.0 Hz, 2H), 7.48 (dd, *J* = 7.5, 1.4 Hz, 1H), 7.45 – 7.41 (m, 1H), 7.37 (dd, *J* = 7.5, 1.7 Hz, 1H), 7.25 (dd, *J* = 7.5, 1.5 Hz, 1H), 7.21 – 7.17 (m, 1H), 7.13 – 7.09 (m, 1H), 4.26 – 4.20 (m, 1H), 3.83 – 3.77 (m, 1H), 3.74 – 3.67 (m, 1H), 3.54 – 3.48 (m, 1H), 1.81 – 1.50 (m, 3H), 1.49 – 1.16 (m, 5H). **^{13}C NMR (101 MHz, Chloroform-*d*) δ:** 196.1, 146.7, 141.8, 138.9, 131.9, 131.4, 131.1, 130.7, 130.4, 129.0, 128.8, 126.7, 123.8, 55.8, 53.4, 27.2, 26.4 (2×C), 26.2. **HRMS (ESI/TOF-Q) m/z:** $[M+H]^+$ Calculated for $C_{19}H_{20}BrNS_2H^+$ 406.0293; Found 406.0295.

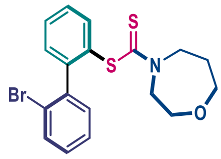

5g

Compound, **5g:** pale yellow sticky liquid; eluent (2% ethyl acetate in hexane). **Yield:** 67% (55 mg); **^{1}H NMR (400 MHz, Chloroform-*d*) δ:** 7.63 – 7.48 (m, 4H), 7.43 – 7.40 (m, 1H), 7.34 – 7.26 (m, 2H), 7.23 – 7.17 (m, 1H), 4.55 – 4.36 (m, 1H), 4.07 – 3.87 (m, 2H), 3.81 – 3.06 (m, 5H), 2.07 – 1.74 (m, 2H). **^{13}C NMR (101 MHz, Chloroform-*d*) δ:** 197.4, 196.8, 146.74, 146.72, 141.7, 141.6, 138.79, 138.76, 132.0, 131.9, 131.5, 131.4, 130.8, 130.71, 130.68, 129.2, 129.1, 128.9, 126.7, 123.9, 70.1, 69.8, 69.6, 69.3, 58.6, 56.4, 53.7, 51.3, 27.8, 27.0. **HRMS (ESI/TOF-Q) m/z:** $[M+H]^+$ Calculated for $C_{19}H_{18}BrNOS_2H^+$ 408.0086; Found 408.0088.

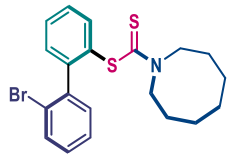

5h

Compound, **5h:** pale yellow sticky liquid; eluent (2% ethyl acetate in hexane). **Yield:** 68% (57 mg); **^{1}H NMR (400 MHz, Chloroform-*d*) δ:** 7.62 – 7.58 (m, 2H), 7.55 (dd, *J* = 7.4, 1.4 Hz, 1H), 7.52 – 7.47 (m, 1H), 7.44 (dd, *J* = 7.6, 1.7 Hz, 1H), 7.33 (dd, *J* = 7.6, 1.5 Hz, 1H), 7.28 – 7.24 (m, 1H), 7.19 – 7.15 (m, 1H), 4.29 – 4.23 (m, 1H), 3.90 – 3.84 (m, 1H), 3.74 – 3.68 (m, 1H), 3.56 – 3.50 (m, 1H), 1.95 – 1.52 (m, 5H), 1.45 – 1.21 (m, 5H). **^{13}C NMR (101 MHz, Chloroform-*d*) δ:** 196.1, 146.8, 141.7, 139.0, 131.9, 131.4, 131.0, 130.7, 130.4, 129.0, 128.8, 126.7, 123.8, 56.6, 54.3, 26.8, 26.3, 25.34, 25.31, 25.2. **HRMS (ESI/TOF-Q) m/z:** $[M+H]^+$ Calculated for $C_{20}H_{22}Br^{81}NS_2H^+$ 422.0450; Found 422.0451.

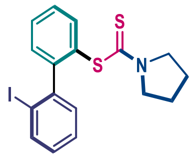

6a

Compound, **6a**: pale yellow sticky liquid; eluent (2% ethyl acetate in hexane). **Yield:** 18% (15 mg); **^{1}H NMR (400 MHz, Chloroform-*d*) δ:** 7.76 (d, *J* = 8.0 Hz, 1H), 7.51 (d, *J* = 7.6 Hz, 1H), 7.44 – 7.30 (m, 2H), 7.32 (dd, *J* = 7.7, 1.5 Hz, 1H), 7.19 (t, *J* = 7.5 Hz, 1H), 7.14 (d, *J* = 6.9 Hz, 1H), 6.92 – 6.88 (m, 1H), 3.74 – 3.60 (m, 2H), 3.53 – 3.47 (m, 1H), 3.33 – 3.27 (m, 1H), 1.94 – 1.74 (m, 4H). **^{13}C NMR (101 MHz, Chloroform-*d*) δ:** 192.5, 149.6, 145.8, 138.7, 138.3, 130.7, 130.6, 130.40, 130.35, 129.1, 128.9, 127.6, 100.4, 55.1, 51.3, 26.3, 24.5. **HRMS (ESI/TOF-Q) m/z:** $[M+H]^+$ Calculated for $C_{17}H_{16}INS_2H^+$ 425.9842; Found 425.9844.

General procedure for the synthesis of compounds 8a-8p:

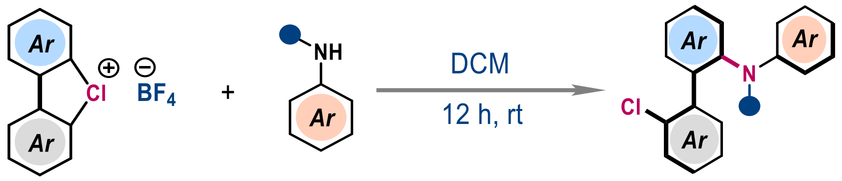

1a 7 8

A 16x100 mm oven dried reaction tube equipped with a magnetic stir was charged with λ^3-chlorane **1a** (0.2 mmol, 1.0 equiv) and corresponding aniline **7** (0.2 mmol, 1.0 equiv) under N_2 atmosphere. Then, dry DCM (2 mL) was added via syringe. The reaction mixture was allowed to stir at rt for 12 h. After completion of the reaction (TLC monitored), volatiles were removed under reduced pressure and the crude product was purified by silica gel column chromatography to provide pure product **8**.

Gram scale synthesis of compound 8f:

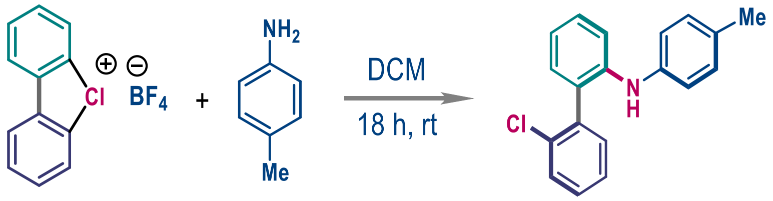

1a 8f

A 100 mL oven dried round bottom flask equipped with a magnetic stir was charged with λ^3-chlorane **1a** (3.64 mmol, 1.0 equiv) and 4-methylaniline (1.0 equiv) under N_2 atmosphere. Then, dry DCM (20 mL) was added via syringe. The reaction mixture was allowed to stir at rt for 18 h. After completion of the reaction (TLC monitored), volatiles were removed under reduced pressure and the crude product was purified by silica gel column chromatography to provide pure product **8f** (0.77 g, 72%).

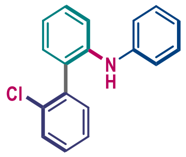

8a

Compound, **8a:** yellow sticky liquid; eluent (1% ethyl acetate in hexane). **Yield:** 69% (39 mg); **^{1}H NMR (400 MHz, Chloroform-*d*) δ:** 7.43 – 7.41 (m, 1H), 7.30 – 7.22 (m, 4H), 7.20 – 7.09 (m, 4H), 6.96 – 6.90 (m, 3H), 6.84 (t, *J* = 7.4 Hz, 1H), 5.24 (s, 1H). **^{13}C NMR (101 MHz, Chloroform-*d*) δ:** 143.2, 141.2, 137.9, 134.3, 132.2, 131.0, 130.1, 129.4, 129.3, 129.04, 128.99, 127.4, 121.5, 120.7, 119.0, 117.0. **HRMS (ESI/TOF-Q) m/z:** $[M+H]^+$ Calculated for $C_{18}H_{14}ClNH^+$ 280.0888; Found 280.0886.

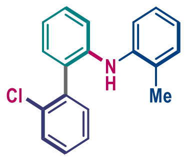

8b

Compound, **8b:** yellow sticky liquid; eluent (1% ethyl acetate in hexane). **Yield:** 76% (45 mg); **^{1}H NMR (400 MHz, Chloroform-*d*) δ**: 7.54 – 7.50 (m, 1H), 7.40 – 7.29 (m, 3H), 7.27 – 7.23 (m, 2H), 7.19 – 7.17 (m, 1H), 7.15 – 7.07 (m, 3H), 6.98 – 6.90 (m, 2H), 5.11 (s, 1H), 2.08 (s, 3H). **^{13}C NMR (101 MHz, Chloroform-*d*) δ:** 141.9, 141.1, 138.0, 134.4, 132.1, 131.0, 130.7, 130.0, 129.5, 129.4, 129.0, 128.3, 127.4, 126.8, 122.5, 120.1, 119.9, 116.2, 17.8. **HRMS (ESI/TOF-Q) m/z:** $[M+H]^+$ Calculated for $C_{19}H_{16}ClNH^+$ 294.1044; Found 294.1039.

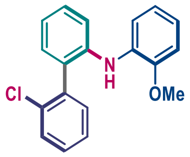

8c

Compound, **8c:** yellow sticky liquid; eluent (1% ethyl acetate in hexane). **Yield:** 72% (45 mg); **^{1}H NMR (400 MHz, Chloroform-*d*) δ:** 7.53 – 7.47 (m, 2H), 7.37 – 7.27 (m, 5H), 7.23 – 7.21 (m, 1H), 7.03 (t, *J* = 7.3 Hz, 1H), 6.89 – 6.80 (m, 3H), 5.81 (s, 1H), 3.73 (s, 3H). **^{13}C NMR (101 MHz, Chloroform-*d*) δ:** 149.0, 140.8, 138.0, 134.3, 133.2, 132.1, 131.0, 130.1, 129.9, 129.1, 128.9, 127.1, 120.9 (2×C), 120.3, 118.0, 115.7, 111.0, 55.8. **HRMS (ESI/TOF-Q) m/z:** $[M+H]^+$ Calculated for $C_{19}H_{16}ClNOH^+$ 310.0993; Found 310.0995.

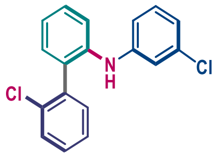

8d

Compound, **8d:** yellow sticky liquid; eluent (1% ethyl acetate in hexane). **Yield:** 62% (39 mg); **^{1}H NMR (400 MHz, Chloroform-*d*) δ**: 7.51 – 7.48 (m, 1H), 7.40 – 7.28 (m, 5H), 7.21 (d, *J* = 7.5 Hz, 1H), 7.13 – 7.06 (m, 1H), 6.98 (s, 1H), 6.85 – 6.83 (m, 2H), 5.35 (s, 1H). **^{13}C NMR (101 MHz, Chloroform-*d*) δ:** 145.0, 140.0, 137.7, 135.0, 134.1, 132.0, 131.2, 130.33, 130.30, 130.1, 129.4, 129.1, 127.4, 122.0, 120.9, 118.6, 117.6, 116.0. **HRMS (ESI/TOF-Q) m/z:** $[M+H]^+$ Calculated for $C_{18}H_{13}Cl_2NH^+$ 314.0498; Found 314.0508.

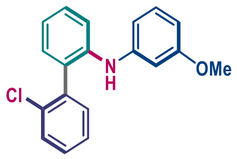

8e

Compound, **8e:** yellow sticky liquid; eluent (1% ethyl acetate in hexane). **Yield:** 63% (39 mg); **^{1}H NMR (400 MHz, Chloroform-*d*) δ**: 7.51 – 7.49 (m, 1H), 7.42 (d, *J* = 8.2 Hz, 1H), 7.34 – 7.29 (m, 4H), 7.19 (d, *J* = 7.2 Hz, 1H), 7.13 (t, *J* = 8.0 Hz, 1H), 7.02 (t, *J* = 7.4 Hz, 1H), 6.59 (s, 2H), 6.48 (d, *J* = 8.5 Hz, 1H), 5.32 (s, 1H), 3.76 (s, 3H). **^{13}C NMR (101 MHz, Chloroform-*d*) δ:** 160.8, 144.7, 140.9, 137.8, 134.3, 132.1, 131.0, 130.09, 130.07, 129.4, 129.3, 129.0, 127.4, 121.0, 117.8, 111.3, 106.7, 104.4, 55.4. **HRMS (ESI/TOF-Q) m/z:** $[M+H]^+$ Calculated for $C_{19}H_{16}ClNOH^+$ 310.0993; Found 310.0995.

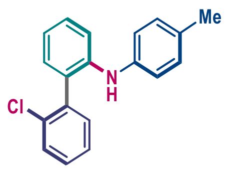

8f

Compound, **8f:** yellow sticky liquid; eluent (1% ethyl acetate in hexane). **Yield:** 78% (46 mg); **^{1}H NMR (400 MHz, Chloroform-*d*) δ**: 7.50 – 7.47 (m, 1H), 7.37 – 7.28 (m, 3H), 7.26 – 7.22 (m, 2H), 7.14 (d, *J* = 7.3 Hz, 1H), 7.04 (d, *J* = 8.1 Hz, 2H), 6.96 – 6.91 (m, 3H), 5.21 (s, 1H), 2.28 (s, 3H). **^{13}C NMR (101 MHz, Chloroform-*d*) δ:** 142.0, 140.4, 137.9, 134.3, 132.2, 131.5, 130.9, 130.1, 129.9, 129.3, 129.0, 128.1, 127.4, 120.1, 119.9, 116.0, 20.8. **HRMS (ESI/TOF-Q) m/z:** $[M+H]^+$ Calculated for $C_{19}H_{16}ClNH^+$ 294.1044; Found 294.1039.

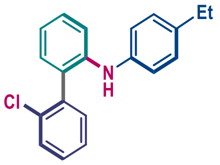
8g

Compound, **8g:** yellow sticky liquid; eluent (1% ethyl acetate in hexane). **Yield:** 80% (49 mg); **^{1}H NMR (400 MHz, Chloroform-*d*) δ**: 7.53 – 7.51 (m, 1H), 7.37 – 7.28 (m, 5H), 7.17 (d, *J* = 7.3 Hz, 1H), 7.10 (d, *J* = 8.1 Hz, 2H), 7.02 – 6.95 (m, 3H), 5.26 (s, 1H), 2.61 (q, *J* = 7.6 Hz, 2H), 1.24 (t, *J* = 7.6 Hz, 3H).**^{13}C NMR (101 MHz, Chloroform-*d*) δ:** 141.92, 140.57, 138.02, 137.91, 134.35, 132.22, 130.89, 130.08, 129.28, 128.98, 128.71, 128.17, 127.39, 120.06, 119.91, 116.02, 28.31, 15.86. **HRMS (ESI/TOF-Q) m/z:** $[M+H]^+$ Calculated for $C_{20}H_{18}ClNH^+$ 308.1201; Found 308.1204.

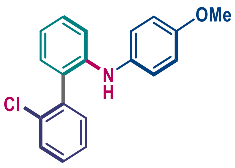
8h

Compound, **8h:** yellow sticky liquid; eluent (1% ethyl acetate in hexane). **Yield:** 76% (47 mg); **^{1}H NMR (400 MHz, Chloroform-*d*) δ**: 7.53 – 7.51 (m, 1H), 7.41 – 7.31 (m, 3H), 7.23 (d, *J* = 7.4 Hz, 1H), 7.15 – 7.05 (m, 4H), 6.91 (t, *J* = 7.4 Hz, 1H), 6.85 (d, *J* = 8.7 Hz, 2H), 5.14 (s, 1H), 3.79 (s, 3H). **^{13}C NMR (101 MHz, Chloroform-*d*) δ:** 155.81, 143.19, 137.94, 135.76, 134.35, 132.29, 130.75, 130.12, 129.30, 129.05, 127.47, 127.07, 123.59, 119.11, 114.75, 114.56, 55.69. **HRMS (ESI/TOF-Q) m/z:** $[M+H]^+$ Calculated for $C_{19}H_{16}ClNOH^+$ 310.0993; Found 310.0995.

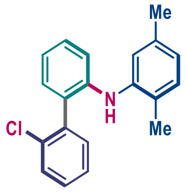
8i

Compound, **8i:** white solid; eluent (1% ethyl acetate in hexane). **Yield:** 79% (49 mg); MP: 118 ^oC; **^{1}H NMR (400 MHz, Chloroform-*d*) δ:** 7.54 – 7.50 (m, 1H), 7.40 – 7.31 (m, 3H), 7.30 – 7.28 (m, 1H), 7.19 – 7.17 (m, 1H), 7.08 – 7.02 (m, 3H), 6.96 (t, *J* = 7.4 Hz, 1H), 6.75 (d, *J* = 7.5 Hz, 1H), 5.08 (s, 1H), 2.27 (s, 3H), 2.04 (s, 3H). **^{13}C NMR (101 MHz, Chloroform-*d*) δ:** 142.0, 140.7, 138.0, 136.5, 134.4, 132.1, 130.8, 130.7, 130.0, 129.3, 129.0, 128.1, 127.4, 126.5, 123.4, 120.9, 119.7, 116.1, 21.2, 17.4. **HRMS (ESI/TOF-Q) m/z:** $[M+H]^+$ Calculated for $C_{20}H_{18}ClNH^+$ 308.1201; Found 308.1204.

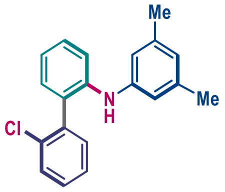
8j

Compound, **8j:** yellow sticky liquid; eluent (1% ethyl acetate in hexane). **Yield:** 80% (49 mg); **^{1}H NMR (400 MHz, Chloroform-*d*) δ:** 7.52 – 7.50 (m, 1H), 7.39 – 7.28 (m, 5H), 7.18 (dd, *J* = 7.6, 1.6 Hz, 1H), 6.99 (t, *J* = 7.3 Hz, 1H), 6.68 (s, 2H), 6.59 (s, 1H), 5.25 (s, 1H), 2.25 (s, 6H). **^{13}C NMR (101 MHz, Chloroform-*d*) δ:** 143.0, 141.3, 139.1, 137.9, 134.3, 132.2, 131.0, 130.1, 129.3, 129.0, 128.8, 127.4, 123.4, 120.3, 117.0, 116.8, 21.5. **HRMS (ESI/TOF-Q) m/z:** $[M+H]^+$ Calculated for $C_{20}H_{18}ClNH^+$ 308.1201; Found 308.1204.

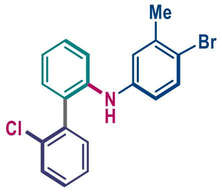
8k

Compound, **8k:** yellow sticky liquid; eluent (1% ethyl acetate in hexane). **Yield:** 75% (56 mg); **^{1}H NMR (400 MHz, Chloroform-*d*) δ:** 7.51 – 7.48 (m, 1H), 7.34 – 7.29 (m, 6H), 7.20 – 7.18 (m, 1H), 7.05 – 7.01 (m, 1H), 6.88 (d, *J* = 2.8 Hz, 1H), 6.73 (dd, *J* = 8.6, 2.7 Hz, 1H), 5.24 (s, 1H), 2.31 (s, 3H). **^{13}C NMR (101 MHz, Chloroform-*d*) δ:** 142.7, 140.7, 138.7, 137.7, 134.2, 132.9, 132.1, 131.1, 130.1, 129.5, 129.4, 129.1, 127.4, 121.2, 120.8, 117.7, 117.6, 115.9, 23.1. **HRMS (ESI/TOF-Q) m/z:** $[M+H]^+$ Calculated for $C_{19}H_{15}BrClNH^+$ 372.0149; Found 372.0153.

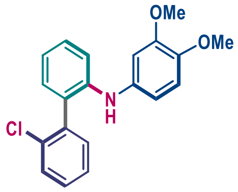

8l

Compound, **8l:** yellow sticky liquid; eluent (1% ethyl acetate in hexane). **Yield:** 81% (59 mg); **^{1}H NMR (400 MHz, Chloroform-*d*) δ:** 7.53 – 7.51 (m, 1H), 7.40 – 7.33 (m, 3H), 7.28 – 7.23 (m, 1H), 7.16 – 7.13 (m, 2H), 6.94 – 6.90 (m, 1H), 6.79 (d, J = 8.3 Hz, 1H), 6.68 – 6.65 (m, 2H), 5.16 (s, 1H), 3.86 (s, 3H), 3.82 (s, 3H). **^{13}C NMR (101 MHz, Chloroform-*d*) δ:** 149.7, 145.2, 142.9, 137.9, 136.3, 134.3, 132.3, 130.8, 130.1, 129.3, 129.1, 127.5, 127.3, 119.4, 115.0, 113.7, 112.2, 106.7, 56.4, 56.0. **HRMS (ESI/TOF-Q) m/z:** [M+Na]$^+$ Calculated for $C_{20}H_{18}ClNO_2Na^+$ 362.0918; Found 362.0929.

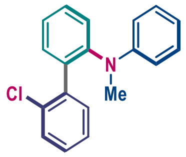

8m

Compound, **8m:** yellow sticky liquid; eluent (1% ethyl acetate in hexane). **Yield:** 79% (46 mg); **^{1}H NMR (400 MHz, Chloroform-*d*) δ:** 7.43 – 7.39 (m, 3H), 7.34 – 7.28 (m, 2H), 7.20 – 7.10 (m, 5H), 6.72 – 6.68 (m, 1H), 6.65 (d, J = 8.1 Hz, 2H), 2.92 (s, 3H). **^{13}C NMR (101 MHz, Chloroform-*d*) δ:** 149.4, 147.0, 138.7, 137.6, 133.2, 132.5, 131.5, 129.7, 129.5, 128.8, 128.7, 128.3, 126.5, 125.4, 117.6, 114.3, 39.4. **HRMS (ESI/TOF-Q) m/z:** [M+H]$^+$ Calculated for $C_{19}H_{16}ClNH^+$ 294.1044; Found 294.1039.

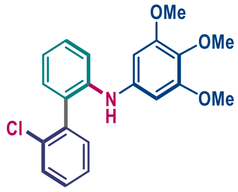

8n

Compound, **8n:** yellow sticky liquid; eluent (1% ethyl acetate in hexane). **Yield:** 62% (49 mg); **^{1}H NMR (400 MHz, Chloroform-*d*) δ:** 7.53 – 7.50 (m, 1H), 7.37 – 7.27 (m, 5H), 7.18 – 7.16 (m, 1H), 7.00 – 6.95 (m, 1H), 6.29 (s, 2H), 5.20 (s, 1H), 3.80 (s, 3H), 3.79 (s, 6H). **^{13}C NMR (101 MHz, Chloroform-*d*) δ:** 153.9, 141.7, 139.2, 137.8, 134.3, 133.4, 132.2, 131.1, 130.1, 129.4, 129.1, 128.5, 127.5, 120.3, 116.6, 97.7, 61.2, 56.2. **HRMS (ESI/TOF-Q) m/z:** [M+Na]$^+$ Calculated for $C_{21}H_{20}ClNO_3Na^+$ 392.1024; Found 392.1031.

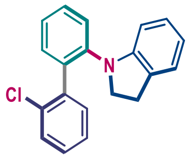

8o

Compound, **8o:** yellow sticky liquid; eluent (1% ethyl acetate in hexane). **Yield:** 76% (47 mg); **^{1}H NMR (400 MHz, Chloroform-*d*) δ:** 7.56 – 7.54 (m, 1H), 7.45 – 7.32 (m, 4H), 7.27 – 7.23 (m, 3H), 7.07 (d, J = 7.1 Hz, 1H), 7.00 (t, J = 8.2 Hz, 1H), 6.69 – 6.65 (m, 2H), 3.40 – 3.32 (m, 2H), 2.94 – 2.87 (m, 2H). **^{13}C NMR (101 MHz, Chloroform-*d*) δ:** 149.7, 144.1, 139.4, 135.7, 133.6, 132.0, 131.8, 130.5, 129.7, 129.2, 128.6, 127.0, 126.7, 124.8, 124.5, 123.4, 118.3, 109.0, 54.1, 28.9. **HRMS (ESI/TOF-Q) m/z:** [M+H]$^+$ Calculated for $C_{20}H_{16}ClNH^+$ 306.1044; Found 306.1040.

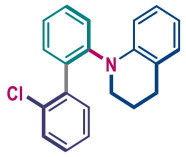

8p

Compound, **8p:** yellow sticky liquid; eluent (1% ethyl acetate in hexane). **Yield:** 74% (47 mg); **^{1}H NMR (400 MHz, Chloroform-*d*) δ:** 7.44 – 7.36 (m, 4H), 7.32 – 7.28 (m, 1H), 7.25 – 7.14 (m, 3H), 6.94 – 6.89 (m, 2H), 6.61 (t, J = 7.3 Hz, 1H), 6.55 (d, J = 8.2 Hz, 1H), 3.15 (d, J = 50.3 Hz, 2H), 2.72 – 2.66 (m, 2H), 1.77 – 1.60 (m, 2H). **^{13}C NMR (101 MHz, Chloroform-*d*) δ:** 146.4, 145.3, 138.7, 137.8, 132.5, 131.6, 129.7, 129.4, 129.3, 128.7, 128.5, 126.7, 126.51, 126.47, 125.4, 122.8, 117.2, 114.8, 50.6, 27.9, 21.9. **HRMS (ESI/TOF-Q) m/z:** [M+H]$^+$ Calculated for $C_{21}H_{18}ClNH^+$ 320.1201; Found 320.1205.

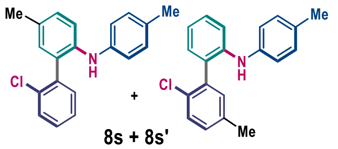

8s + 8s'

Compound, **8s+8s′:** yellow sticky liquid; eluent (1% ethyl acetate in hexane). **Yield:** 72% (2.4:1 rr) (44 mg); **^{1}H NMR (400 MHz, Chloroform-*d*) δ:** 7.43 – 7.38 (m, 1H), 7.30 – 7.28 (m, 2.52H), 7.26 – 7.19 (m, 3.96H), 7.18 – 7.12 (m, 2.92H), 7.09 – 6.93 (m, 16.96H), 6.91 – 6.80 (m, 10.22H), 5.17 (s, 2.41H), 5.05 (s, 1H), 2.27 (s, 7.19H), 2.25 (s, 3H), 2.21 (s, 7.21H), 2.19 (s, 3H). **^{13}C NMR (101 MHz, Chloroform-*d*) δ:** 142.0, 141.2, 140.4, 139.2, 138.1, 137.6, 137.3, 132.8, 132.2, 131.5, 131.4, 131.2, 130.8, 130.1, 130.0, 129.90, 129.85, 129.7, 129.6, 129.2, 128.9, 128.2, 127.3, 120.3, 119.8, 119.0, 117.3, 115.7, 21.0, 20.8, 20.8, 20.7. **HRMS (ESI/TOF-Q) m/z:** $[M+H]^+$ Calculated for $C_{20}H_{18}ClNH^+$ 308.1201; Found 308.1194.

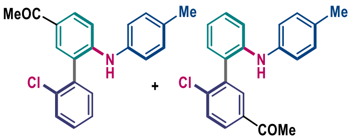

8t + 8t'

Compound, **8t+8t′:** yellow sticky liquid; eluent (1% ethyl acetate in hexane). **Yield:** 70% (2.3:1 rr) (47 mg); **^{1}H NMR (500 MHz, Chloroform-*d*) δ:** 7.94 – 7.90 (m, 2.02H), 7.87 – 7.84 (m, 2.07H), 7.76 (t, *J* = 1.9 Hz, 2.03H), 7.60 – 7.53 (m, 3.00H), 7.41 – 7.36 (m, 6.54H), 7.31 – 7.26 (m, 2.42H), 7.16 – 7.12 (m, 7.80H), 7.06 – 7.04 (m, 6.38H), 7.00 – 6.93 (m, 3.82H), 5.59 (s, 2.34H), 5.12 (s, 1.00H), 2.57 (s, 3.02H), 2.53 (s, 6.90H), 2.33 (s, 6.92H), 2.28 (s, 3.09H). **^{13}C NMR (101 MHz, Chloroform-*d*) δ:** 196.9, 196.4, 147.2, 142.0, 140.3, 139.7, 138.5, 137.9, 136.7, 136.3, 134.4, 134.2, 132.4, 132.3, 131.9, 131.7, 130.8, 130.4, 130.3, 130.3, 130.2, 130.0, 129.9, 129.5, 128.9, 128.2, 127.8, 125.8, 123.0, 120.3, 120.1, 116.6, 112.4, 26.7, 26.3, 21.0, 20.8. **HRMS (ESI/TOF-Q) m/z:** $[M+H]^+$ Calculated for $C_{21}H_{18}ClNOH^+$ 336.1150; Found 336.1151.

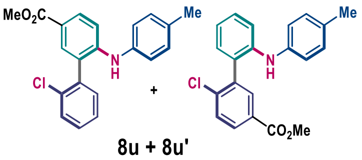

8u + 8u'

Compound, **8u+8u′:** yellow sticky liquid; eluent (1% ethyl acetate in hexane). **Yield:** 75% (2:1) (51 mg); **for major isomer**; **^{1}H NMR (500 MHz, Chloroform-*d*) δ:** 7.91 – 7.89 (m, 1H), 7.82 – 7.81 (m, 1H), 7.53 – 7.52 (m, 1H), 7.40 – 7.36 (m, 3H), 7.14 – 7.12 (m, 3H), 7.05 – 7.03 (m, 2H), 5.53 (s, 1H), 3.86 (s, 3H), 2.32 (s, 3H). **^{13}C NMR (126 MHz, Chloroform-*d*) δ:** 167.1, 146.9, 138.1, 136.7, 134.4, 133.9, 132.7, 132.4, 131.2, 130.3, 130.2, 129.8, 127.7, 125.9, 122.7, 120.2, 112.6, 51.9, 21.0. **HRMS (ESI/TOF-Q) m/z:** $[M+H]^+$ Calculated for $C_{21}H_{19}ClNO_2H^+$ 352.1099; Found 352.1101. **for minor isomer**; **^{1}H NMR (400 MHz, Chloroform-*d*) δ**: 8.05 (d, *J* = 2.1 Hz, 1H), 7.98 (dd, *J* = 8.4, 2.2 Hz, 1H), 7.57 (d, *J* = 8.4 Hz, 1H), 7.28 – 7.25 (m, 2H), 7.13 (dd, *J* = 7.3, 1.2 Hz, 1H), 7.06 – 7.04 (m, 2H), 6.98 – 6.93 (m, 3H), 5.11 (s, 1H), 3.91 (s, 3H), 2.28 (s, 3H). **^{13}C NMR (101 MHz, Chloroform-*d*) δ**: 166.3, 142.0, 140.1, 139.5, 138.4, 133.4, 131.7, 130.8, 130.3, 130.2, 129.9, 129.5, 129.4, 127.3, 120.2, 120.1, 116.18, 52.5, 20.9. **HRMS (ESI/TOF-Q) m/z:** $[M+H]^+$ Calculated for $C_{21}H_{19}ClNO_2H^+$ 352.1099; Found 352.1101.

General procedure for the synthesis of compounds 9a-9d:

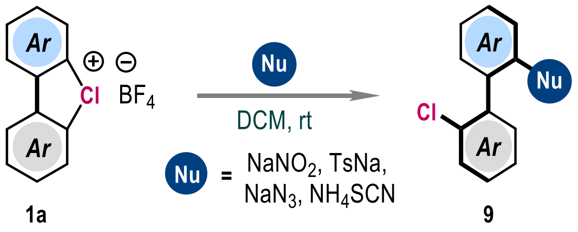

A 16x100 mm oven dried reaction tube equipped with a magnetic stir was charged with λ^3-chlorane **1a** (0.2 mmol, 1.0 equiv) and corresponding nucleophile (0.2 mmol, 1.0 equiv) under N_2 atmosphere. Then, dry DCM (1.5 mL) was added via syringe. The reaction mixture was allowed to stir at rt. After completion of the reaction (8–18 h, TLC monitored), volatiles were removed under reduced pressure and the crude product was purified by silica gel column chromatography to provide pure product **9.**

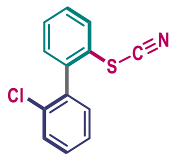

9a

Compound, **9a:** yellow liquid; eluent (1% ethyl acetate in hexane). **Yield:** 91% (45 mg); **^{1}H NMR (400 MHz, Chloroform-*d*) δ**: 7.71 (d, *J* = 7.2 Hz, 1H), 7.42 – 7.36 (m, 3H), 7.33 – 7.26 (m, 2H), 7.22 (d, *J* = 6.9 Hz, 1H), 7.19 – 7.16 (m, 1H). **^{13}C NMR (101 MHz, Chloroform-*d*) δ:** 140.3, 137.3, 133.6, 131.3 (2×C), 130.43, 130.36, 130.0, 129.9, 129.5, 127.2, 125.1, 110.8. **HRMS (ESI/TOF-Q) m/z:** $[M+H]^+$ Calculated for $C_{13}H_8ClNSH^+$ 246.0139; Found 246.0140.

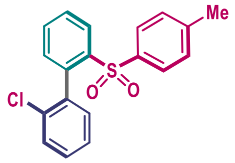

9b

Compound, **9b:** yellow liquid; eluent (3% ethyl acetate in hexane). **Yield:** 85% (58 mg); **^{1}H NMR (400 MHz, Chloroform-*d*) δ**: 8.45 – 8.40 (m, 1H), 7.63 – 7.58 (m, 2H), 7.31 – 7.25 (m, 3H), 7.22 – 7.16 (m, 4H), 7.04 (d, *J* = 8.0 Hz, 2H), 2.35 (s, 3H). **^{13}C NMR (101 MHz, Chloroform-*d*) δ:** 143.9, 140.0, 138.8, 137.5, 136.5, 134.0, 133.1, 132.91, 132.87, 129.6, 129.3, 128.91, 128.86, 128.5, 128.2, 125.7, 21.7. **HRMS (ESI/TOF-Q) m/z:** $[M+H]^+$ Calculated for $C_{19}H_{15}ClO_2SH^+$ 343.0554; Found 343.0559.

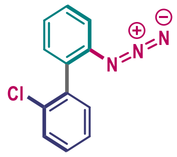

9c

Compound, **9c:** colourless liquid; eluent (1% ethyl acetate in hexane). **Yield:** 90% (41 mg); **^{1}H NMR (400 MHz, Chloroform-*d*) δ**: 7.43 – 7.36 (m, 2H), 7.28 – 7.23 (m, 2H), 7.20 – 7.12 (m, 4H). **^{13}C NMR (101 MHz, Chloroform-*d*) δ:** 138.3, 137.3, 133.8, 131.6, 131.5, 131.4, 129.6, 129.5, 129.3, 126.6, 124.7, 118.6. **HRMS (ESI/TOF-Q) m/z:** $[M+H]^+$ Calculated for $C_{12}H_8ClN_3H^+$ 230.0480; Found 230.0482.

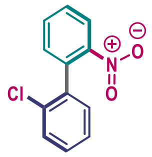

9d

Compound, **9d:** yellow liquid; eluent (2% ethyl acetate in hexane). **Yield:** 88% (41 mg); **^{1}H NMR (400 MHz, Chloroform-*d*) δ**: 7.98 (d, *J* = 8.1 Hz, 1H), 7.56 (t, *J* = 7.6 Hz, 1H), 7.45 (t, *J* = 7.8 Hz, 1H), 7.35 – 7.33 (m, 1H), 7.24 – 7.21 (m, 2H), 7.16 – 7.14 (m, 2H). **^{13}C NMR (101 MHz, Chloroform-*d*) δ:** 148.8, 137.3, 134.5, 133.1, 132.9, 132.5, 130.0, 129.6 (2×C), 129.1, 127.1, 124.6. **HRMS (ESI/TOF-Q) m/z:** $[M+H]^+$ Calculated for $C_{12}H_8ClNO_2H^+$ 234.0316; Found 234.0322.

General procedure for the synthesis of compounds 10a-10p:

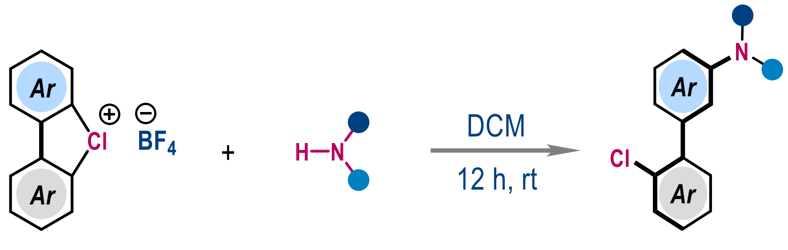

A 16x100 mm oven dried reaction tube equipped with a magnetic stir was charged with λ^3-chlorane **1** (0.2 mmol, 1.0 equiv) and corresponding secondary amine **3** (0.3 mmol, 1.5 equiv) under N_2 atmosphere. Then, dry DCM (1.5 mL) was added via syringe. The reaction mixture was allowed to stir at rt for 12 h. After completion of the reaction (TLC monitored), volatiles were removed under reduced pressure and the crude product was purified by silica gel chromatography to provide pure product **10.**

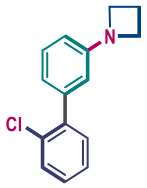

10a-*meta*

Compound, **10a-*meta*:** colourless sticky liquid; eluent (1% ethyl acetate in hexane). **Yield:** 60% (29 mg); **^{1}H NMR (400 MHz, Chloroform-*d*) δ:** 7.45 (dd, *J* = 7.2, 1.9 Hz, 1H), 7.35 (dd, *J* = 7.3, 2.2 Hz, 1H), 7.31 – 7.25 (m, 3H), 6.79 (d, *J* = 7.5 Hz, 1H), 6.50 – 6.46 (m, 2H), 3.91 (t, *J* = 7.2 Hz, 4H), 2.37 (p, *J* = 7.2 Hz, 2H). **^{13}C NMR (101 MHz, Chloroform-*d*) δ:** 152.0, 141.3, 140.2, 132.7, 131.5, 130.0, 128.7, 128.4, 126.8, 118.7, 112.6, 110.7, 52.6, 17.2. **HRMS (ESI/TOF-Q) m/z:** $[M+H]^+$ Calculated for $C_{15}H_{14}ClNH^+$ 244.0888; Found 244.0886.

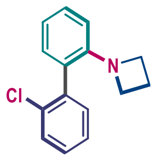

10a-*ortho*

Compound, **10a-*ortho*:** colourless sticky liquid; eluent (1% ethyl acetate in hexane). **Yield:** 20% (9 mg); **^{1}H NMR (400 MHz, Chloroform-*d*) δ:** 7.46 – 7.41 (m, 1H), 7.36 – 7.33 (m, 1H), 7.29 – 7.23 (m, 3H), 7.04 (dd, *J* = 7.5, 1.6 Hz, 1H), 6.82 (td, *J* = 7.4, 1.2 Hz, 1H), 6.55 (dd, *J* = 8.2, 1.2 Hz, 1H), 3.51 (q, *J* = 7.2 Hz, 2H), 3.41 (q, *J* = 7.2 Hz, 2H), 2.08 (p, *J* = 7.2 Hz, 2H). **^{13}C NMR (101 MHz, Chloroform-*d*) δ:** 145.0, 140.0, 134.3, 132.6, 131.5, 129.4, 128.6, 128.5, 126.3, 125.0, 117.7, 112.8, 53.6, 17.1. **HRMS (ESI/TOF-Q) m/z:** $[M+H]^+$ Calculated for $C_{15}H_{14}ClNH^+$ 244.0888; Found 244.0886.

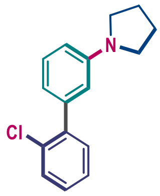

10b-*meta*

Compound, **10b-*meta*:** colourless sticky liquid; eluent (1% ethyl acetate in hexane). **Yield:** 74% (38 mg); **^{1}H NMR (400 MHz, Chloroform-*d*) δ**: 7.46 (d, *J* = 7.7 Hz, 1H), 7.39 – 7.37 (m, 1H), 7.30 – 7.26 (m, 3H), 6.71 (d, *J* = 7.6 Hz, 1H), 6.61 – 6.59 (m, 2H), 3.34 – 3.30 (m, 4H), 2.03 – 1.99 (m, 4H). **^{13}C NMR (101 MHz, Chloroform-*d*) δ:** 147.7, 141.7, 140.4, 132.7, 131.5, 129.9, 128.9, 128.3, 126.7, 116.7, 112.8, 111.0, 47.8, 25.6. **HRMS (ESI/TOF-Q) m/z:** $[M+H]^+$ Calculated for $C_{16}H_{16}ClNH^+$ 258.1044; Found 258.1045.

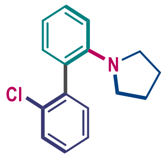

10b-*ortho*

Compound, **10b-*ortho*:** colourless sticky liquid; eluent (1% ethyl acetate in hexane). **Yield:** 9% (5 mg); **^{1}H NMR (400 MHz, Chloroform-*d*) δ**: 7.42 – 7.38 (m, 1H), 7.34 – 7.31 (m, 1H), 7.24 – 7.18 (m, 3H), 7.06 (d, *J* = 7.5 Hz, 1H), 6.82 – 6.76 (m, 2H), 2.94 – 2.83 (m, 4H), 1.79 – 1.70 (m, 4H).**^{13}C NMR (101 MHz, Chloroform-*d*) δ**: 147.8, 142.2, 134.0, 132.5, 132.3, 129.3, 128.6, 128.0, 126.3 (2×C), 117.0, 114.2, 50.3, 25.8. **HRMS (ESI/TOF-Q) m/z:** [M+H]$^{+}$ Calculated for $C_{16}H_{16}ClNH^{+}$ 258.1044; Found 258.1045.

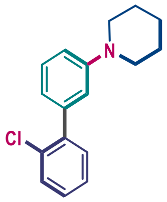

10c

Compound, **10c:** colourless sticky liquid; eluent (1% ethyl acetate in hexane). **Yield:** 85% (46 mg); **^{1}H NMR (400 MHz, Chloroform-*d*) δ:** 7.46 (d, *J* = 7.5 Hz, 1H), 7.36 (d, *J* = 7.4 Hz, 1H), 7.33 – 7.26 (m, 3H), 7.00 (s, 1H), 6.96 (d, *J* = 8.5 Hz, 1H), 6.89 (d, *J* = 7.6 Hz, 1H), 3.22 – 3.19 (m, 4H), 1.74 – 1.70 (m, 4H), 1.60 – 1.57 (m, 2H). **^{13}C NMR (101 MHz, Chloroform-*d*) δ:** 152.0, 141.3, 140.3, 132.6, 131.5, 130.0, 128.8, 128.4, 126.8, 120.4, 117.9, 115.7, 50.7, 26.0, 24.4. **HRMS (ESI/TOF-Q) m/z:** [M+H]$^{+}$ Calculated for $C_{17}H_{18}ClNH^{+}$ 272.1201; Found 272.1209.

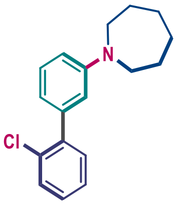

10d

Compound, **10d:** colourless sticky liquid; eluent (1% ethyl acetate in hexane). **Yield:** 90% (52 mg); **^{1}H NMR (400 MHz, Chloroform-*d*) δ:** 7.48 – 7.46 (m, 1H), 7.39 (d, *J* = 7.4 Hz, 1H), 7.33 – 7.26 (m, 3H), 6.75 – 6.68 (m, 3H), 3.50 – 3.47 (m, 4H), 1.81 – 1.80 (m, 4H), 1.58 (brs, 4H). **^{13}C NMR (101 MHz, Chloroform-*d*) δ:** 148.6, 141.8, 140.5, 132.7, 131.5, 130.0, 129.0, 128.3, 126.8, 116.4, 112.6, 110.6, 49.3, 28.0, 27.3. **HRMS (ESI/TOF-Q) m/z:** [M+H]$^{+}$ Calculated for $C_{18}H_{20}ClNH^{+}$ 286.1357; Found 286.1363.

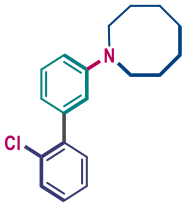

10e

Compound, **10e:** colourless sticky liquid; eluent (1% ethyl acetate in hexane). **Yield:** 88% (53 mg); **^{1}H NMR (400 MHz, Chloroform-*d*) δ**: 7.44 (d, *J* = 7.6 Hz, 1H), 7.37 (d, *J* = 7.3 Hz, 1H), 7.30 – 7.23 (m, 3H), 6.70 – 6.67 (m, 3H), 3.47 – 3.44 (m, 4H), 1.76 – 1.74 (m, 4H), 1.58 – 1.55 (m, 6H). **^{13}C NMR (101 MHz, Chloroform-*d*) δ**: 148.0, 141.8, 140.4, 132.7, 131.5, 130.0, 128.9, 128.3, 126.8, 116.3, 112.6, 110.5, 50.9, 27.4, 27.3, 27.1. **HRMS (ESI/TOF-Q) m/z:** [M+H]$^{+}$ Calculated for $C_{19}H_{22}ClNH^{+}$ 300.1514; Found 300.1514.

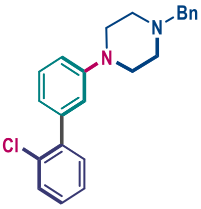

10f

Compound, **10f:** colourless sticky liquid; eluent (3% ethyl acetate in hexane). **Yield:** 82% (59 mg); **^{1}H NMR (400 MHz, Chloroform-*d*) δ:** 7.37 – 7.35 (m, 1H), 7.28 – 7.14 (m, 9H), 6.89 (s, 1H), 6.85 – 6.81 (m, 2H), 3.49 (s, 2H), 3.17 – 3.15 (m, 4H), 2.55 – 2.52 (m, 4H). **^{13}C NMR (101 MHz, Chloroform-*d*) δ:** 151.1, 141.1, 140.4, 138.0, 132.6, 131.4, 130.0, 129.4, 128.9, 128.5, 128.4, 127.3, 126.8, 120.8, 117.5, 115.3, 63.2, 53.2, 49.2. **HRMS (ESI/TOF-Q) m/z:** [M+H]$^{+}$ Calculated for $C_{23}H_{23}ClN_2H^{+}$ 363.1623; Found 363.1625.

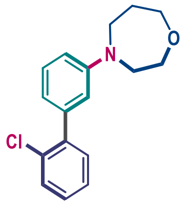

10g

Compound, **10g:** colourless sticky liquid; eluent (1% ethyl acetate in hexane). **Yield:** 82% (47 mg); **^{1}H NMR (400 MHz, Chloroform-*d*) δ:** 7.46 (d, *J* = 7.6 Hz, 1H), 7.37 (d, *J* = 7.3 Hz, 1H), 7.32 – 7.26 (m, 3H), 6.76 – 6.73 (m, 3H), 3.86 – 3.84 (m, 2H), 3.73 – 3.70 (m, 2H), 3.67 – 3.63 (m, 4H), 2.08 – 2.04 (m, 2H). **^{13}C NMR (101 MHz, Chloroform-*d*) δ:** 148.0, 141.4, 140.7, 132.6, 131.4, 130.0, 129.3, 128.5, 126.8, 117.5, 113.3, 111.2, 70.22, 70.16, 52.3, 47.7, 29.3. **HRMS (ESI/TOF-Q) m/z:** $[M+H]^+$ Calculated for $C_{17}H_{18}ClNOH^+$ 288.1150; Found 288.1155.

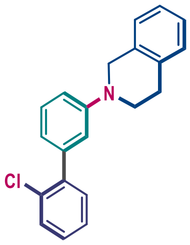

10h

Compound, **10h:** colourless sticky liquid; eluent (1% ethyl acetate in hexane). **Yield:** 79% (51 mg); **^{1}H NMR (400 MHz, Chloroform-*d*) δ:** 7.48 – 7.36 (m, 4H), 7.26 – 7.19 (m, 3H), 7.13 – 7.09 (m, 3H), 7.05 – 7.01 (m, 2H), 4.22 – 4.13 (m, 2H), 3.12 – 2.96 (m, 2H), 2.47 – 2.44 (m, 2H). **^{13}C NMR (101 MHz, Chloroform-*d*) δ:** 150.8, 140.0, 135.2, 135.0, 133.4, 133.3, 132.1, 132.0, 129.9, 129.0, 128.9, 128.3, 126.6, 126.5, 126.1, 125.7, 122.1, 118.8, 52.7, 50.5, 29.2. **HRMS (ESI/TOF-Q) m/z:** $[M+H]^+$ Calculated for $C_{21}H_{18}ClNH^+$ 320.1201; Found 320.1203.

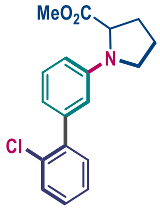

10i

Compound, **10i:** colourless sticky liquid; eluent (1% ethyl acetate in hexane). **Yield:** 72% (45 mg); **^{1}H NMR (400 MHz, Chloroform-*d*) δ:** 7.45 (dd, *J* = 7.4, 1.9 Hz, 1H), 7.35 (dd, *J* = 7.1, 2.2 Hz, 1H), 7.31 – 7.24 (m, 3H), 6.77 (d, *J* = 7.5 Hz, 1H), 6.61 (s, 1H), 6.57 (dd, *J* = 8.2, 2.6 Hz, 1H), 4.29 (dd, *J* = 8.5, 2.1 Hz, 1H), 3.73 (s, 3H), 3.64 – 3.59 (m, 1H), 3.43 – 3.37 (m, 1H), 2.35 – 2.23 (m, 1H), 2.21 – 2.12 (m, 2H), 2.10 – 2.00 (m, 1H).**^{13}C NMR (101 MHz, Chloroform-*d*) δ:** 175.0, 146.5, 141.4, 140.5, 132.7, 131.5, 130.0, 129.1, 128.4, 126.8, 118.1, 113.3, 111.4, 61.0, 52.3, 48.5, 31.1, 24.0. **HRMS (ESI/TOF-Q) m/z:** $[M+H]^+$ Calculated for $C_{18}H_{18}ClNO_2H^+$ 316.1099; Found 316.1099.

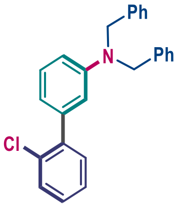

10j

Compound, **10j:** colourless sticky liquid; eluent (1% ethyl acetate in hexane). **Yield:** 81% (62 mg); **^{1}H NMR (400 MHz, Chloroform-*d*) δ:** 7.40 – 7.29 (m, 8H), 7.25 – 7.07 (m, 7H), 6.81 (s, 1H), 6.75 (d, *J* = 6.9 Hz, 2H), 4.65 (s, 4H). **^{13}C NMR (101 MHz, Chloroform-*d*) δ:** 149.1, 141.2, 140.4, 138.6, 132.6, 131.5, 130.0, 129.0, 128.8, 128.4, 127.0, 126.9, 126.8, 118.1, 114.0, 111.9, 54.3. **HRMS (ESI/TOF-Q) m/z:** $[M+H]^+$ Calculated for $C_{26}H_{22}ClNH^+$ 384.1514; Found 384.1517.

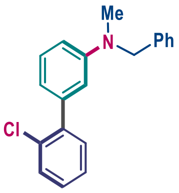

10k

Compound, **10k:** colourless sticky liquid; eluent (1% ethyl acetate in hexane). **Yield:** 68% (42 mg); **^{1}H NMR (400 MHz, Chloroform-*d*) δ:** 7.43 (dd, *J* = 7.2, 2.0 Hz, 1H), 7.34 – 7.28 (m, 4H), 7.27 – 7.21 (m, 5H), 6.82 (s, 1H), 6.78 – 6.75 (m, 2H), 4.55 (s, 2H), 3.03 (s, 3H). **^{13}C NMR (101 MHz, Chloroform-*d*) δ:** 149.6, 141.5, 140.4, 139.1, 132.7, 131.5, 130.0, 129.0, 128.7, 128.4, 127.03, 126.96, 126.8, 117.9, 113.8, 111.8, 56.9, 38.7. **HRMS (ESI/TOF-Q) m/z:** $[M+H]^+$ Calculated for $C_{20}H_{18}ClNH^+$ 308.1201; Found 308.1209.

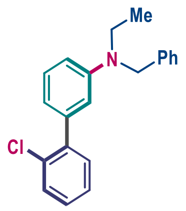

10l

Compound, **10l:** colourless sticky liquid; eluent (1% ethyl acetate in hexane). **Yield:** 71% (46 mg); **^{1}H NMR (400 MHz, Chloroform-*d*) δ:** 7.40 (d, *J* = 7.7 Hz, 1H), 7.30 – 7.21 (m, 9H), 6.78 (s, 1H), 6.73 (d, *J* = 7.8 Hz, 2H), 4.53 (s, 2H), 3.49 – 3.47 (m, 2H), 1.23 – 1.20 (m, 3H). **^{13}C NMR (126 MHz, Chloroform-*d*) δ:** 141.3, 140.4, 132.6, 131.5, 130.0, 129.0, 128.7 (2×C), 128.4, 127.0, 126.8, 122.3, 120.2, 117.6, 113.8, 111.8, 54.4, 45.5, 12.3. **HRMS (ESI/TOF-Q) m/z:** $[M+H]^+$ Calculated for $C_{21}H_{20}ClNH^+$ 322.1357; Found 322.1369.

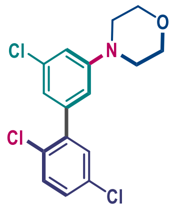

10m

Compound, **10m:** colourless sticky liquid; eluent (1% ethyl acetate in hexane). **Yield:** 90% (62 mg); **^{1}H NMR (400 MHz, Chloroform-*d*) δ:** 7.42 (d, *J* = 8.6 Hz, 1H), 7.30 – 7.26 (m, 2H), 7.23 (d, *J* = 2.6 Hz, 1H), 7.21 – 7.17 (m, 2H), 3.67 (t, *J* = 5.0 Hz, 4H), 3.54 (brs, 4H). **^{13}C NMR (101 MHz, Chloroform-*d*) δ:** 146.2, 139.7, 134.9, 132.2, 132.0, 131.6, 131.4, 130.18, 130.16, 129.3, 128.7, 118.5, 66.41, 66.39. **HRMS (ESI/TOF-Q) m/z:** $[M+H]^+$ Calculated for $C_{16}H_{14}Cl_3NOH^+$ 342.0214; Found 342.0218.

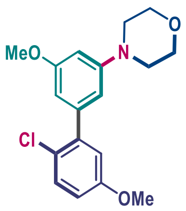

10n

Compound, **10n:** colourless sticky liquid; eluent (1% ethyl acetate in hexane). **Yield:** 84% (56 mg); **^{1}H NMR (400 MHz, Chloroform-*d*) δ:** 7.45 (d, *J* = 8.9 Hz, 1H), 7.23 (d, *J* = 7.1 Hz, 1H), 6.90 (dd, *J* = 8.9, 2.9 Hz, 1H), 6.82 (d, *J* = 3.1 Hz, 1H), 6.79 – 6.76 (m, 2H), 3.80 (s, 3H), 3.75 (s, 3H), 3.69 (t, *J* = 5.0 Hz, 4H), 3.50 (brs, 4H). **^{13}C NMR (101 MHz, Chloroform-*d*) δ:** 158.1, 157.8, 141.4, 140.2, 136.3, 129.6, 125.4, 118.2, 116.9, 115.2, 114.8, 114.4, 66.5, 55.7 (2×C), 48.1. **HRMS (ESI/TOF-Q) m/z:** $[M+H]^+$ Calculated for $C_{18}H_{20}ClNO_3H^+$ 334.1204; Found 334.1205.

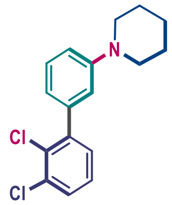

10o

Compound, **10o:** colourless sticky liquid; eluent (1% ethyl acetate in hexane). **Yield:** 76% (46 mg); **^{1}H NMR (400 MHz, Chloroform-*d*) δ:** 7.44 (dd, *J* = 7.2, 2.4 Hz, 1H), 7.30 (t, *J* = 7.8 Hz, 1H), 7.26 – 7.20 (m, 2H), 6.98 – 6.94 (m, 2H), 6.84 – 6.82 (m, 1H), 3.20 (t, *J* = 5.4 Hz, 4H), 1.75 – 1.69 (m, 4H), 1.62 – 1.57 (m, 2H). **^{13}C NMR (101 MHz, Chloroform-*d*) δ:** 152.0, 143.7, 140.2, 133.6, 131.3, 129.6, 129.3, 128.9, 127.1, 120.0, 117.6, 115.9, 50.6, 26.0, 24.5. **HRMS (ESI/TOF-Q) m/z:** $[M+H]^+$ Calculated for $C_{17}H_{17}Cl_2NH^+$ 306.0811; Found 306.0810.

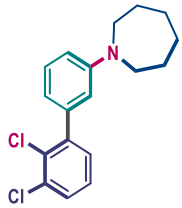

10p

Compound, **10p:** colourless sticky liquid; eluent (1% ethyl acetate in hexane). **Yield:** 79% (50 mg); **^{1}H NMR (400 MHz, Chloroform-*d*) δ:** 7.44 (dd, *J* = 7.7, 1.9 Hz, 1H), 7.28 – 7.20 (m, 3H), 6.73 (dd, *J* = 8.3, 2.7 Hz, 1H), 6.69 (s, 1H), 6.63 (d, *J* = 7.6 Hz, 1H), 3.48 (t, *J* = 6.0 Hz, 4H), 1.81 – 1.79 (m, 4H), 1.59 – 1.56 (m, 4H). **^{13}C NMR (101 MHz, Chloroform-*d*) δ:** 148.7, 144.2, 140.4, 133.5, 131.3, 129.6, 129.2, 129.1, 127.1, 116.2, 112.4, 110.9, 49.3, 27.9, 27.3. **HRMS (ESI/TOF-Q) m/z:** $[M+H]^+$ Calculated for $C_{18}H_{19}Cl_2NH^+$ 320.0967; Found 320.0976.

General procedure for the synthesis of compounds 11/12:

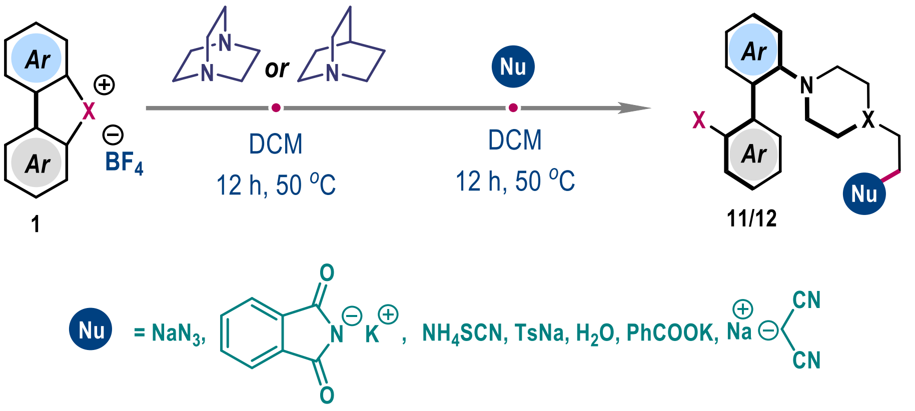

A 16x100 mm oven dried reaction tube equipped with a magnetic stir was charged with λ^3-chlorane **1a** (0.2 mmol, 1.0 equiv) and tertiary amine DABCO or quinuclidine (0.2 mmol, 1.0 equiv) under N_2 atmosphere. Then, dry DCM (1.5 mL) was added via syringe. The reaction mixture was allowed to stir at 50 °C for 12 h. Then, the corresponding nucleophile (1.2 equiv; for water, 6 equiv) was added in the reaction mixture and allowed to stir further for 12 h at 50 °C. After completion of the reaction, volatiles were removed under reduced pressure and the crude product was purified by silica gel column chromatography to provide pure product **11/12.**

Compound **11h** was prepared similarly using λ^3-bromane (**1a**), DABCO, and NaN_3.

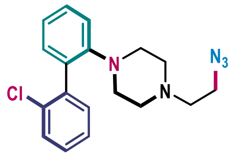

11a

Compound, **11a:** yellow sticky liquid; eluent (4% ethyl acetate in hexane). **Yield:** 84% (57 mg); **^{1}H NMR (400 MHz, Chloroform-*d*) δ**: 7.48 – 7.45 (m, 1H), 7.39 – 7.33 (m, 2H), 7.31 – 7.24 (m, 2H), 7.20 (dd, *J* = 7.7, 1.8 Hz, 1H), 7.11 – 7.08 (m, 2H), 3.30 (t, *J* = 6.1 Hz, 2H), 2.86 (t, *J* = 4.9 Hz, 4H), 2.52 (t, *J* = 6.1 Hz, 2H), 2.33 – 2.31 (m, 4H). **^{13}C NMR (101 MHz, Chloroform-*d*) δ:** 150.9, 139.9, 134.0, 133.6, 132.0, 131.5, 129.8, 129.1, 128.2, 126.5, 122.7, 119.2, 57.2, 53.5, 51.2, 48.3. **HRMS (ESI/TOF-Q) m/z:** $[M+H]^+$ Calculated for $C_{18}H_{20}ClN_5H^+$ 342.1480; Found 342.1482.

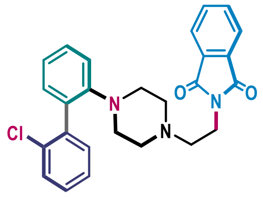

11b

Compound, **11b:** yellow sticky liquid; eluent (5% ethyl acetate in hexane). **Yield:** 66% (59 mg); **^{1}H NMR (400 MHz, Chloroform-*d*) δ**: 7.87 – 7.78 (m, 2H), 7.77 – 7.67 (m, 2H), 7.47 – 7.43 (m, 1H), 7.38 – 7.35 (m, 1H), 7.33 – 7.27 (m, 2H), 7.25 – 7.21 (m, 1H), 7.19 – 7.16 (m, 1H), 7.08 – 7.03 (m, 2H), 3.77 (t, *J* = 6.6 Hz, 2H), 2.78 (t, *J* = 4.9 Hz, 4H), 2.56 (t, *J* = 6.7 Hz, 2H), 2.34 (brs, 4H). **^{13}C NMR (101 MHz, Chloroform-*d*) δ:** 168.5, 151.0, 139.9, 134.0, 133.9, 133.5, 132.3, 132.0, 131.5, 129.8, 129.0, 128.3, 126.5, 123.3, 122.5, 119.1, 55.8, 53.3, 51.2, 35.3. **HRMS (ESI/TOF-Q) m/z:** $[M+H]^+$ Calculated for $C_{26}H_{24}ClN_3O_2H^+$ 446.1630; Found 446.1638.

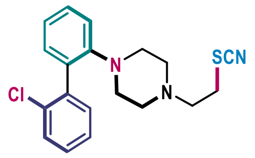

11c

Compound, **11c:** yellow sticky liquid; eluent (3% ethyl acetate in hexane). **Yield:** 78% (56 mg); **^{1}H NMR (400 MHz, Chloroform-*d*) δ**: 7.46 (dd, *J* = 7.2, 2.1 Hz, 1H), 7.38 – 7.33 (m, 2H), 7.31 – 7.24 (m, 2H), 7.19 (dd, *J* = 7.5, 1.8 Hz, 1H), 7.12 – 7.08 (m, 2H), 3.13 (t, *J* = 6.7 Hz, 2H), 2.84 (t, *J* = 4.9 Hz, 4H), 2.66 (t, *J* = 6.7 Hz, 2H), 2.33 – 2.27 (m, 4H). **^{13}C NMR (101 MHz, Chloroform-*d*) δ:** 150.8, 139.8, 134.0, 133.6, 132.0, 131.5, 129.8, 129.1, 128.3, 126.5, 122.8, 119.2, 113.2, 56.3, 53.0, 51.1, 32.6. **HRMS (ESI/TOF-Q) m/z:** $[M+H]^+$ Calculated for $C_{19}H_{20}ClN_3SH^+$ 358.1139; Found 358.1152.

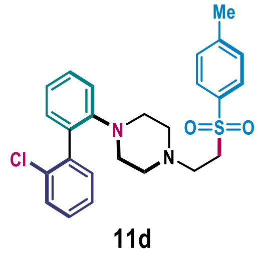

11d

Compound, **11d:** yellow sticky liquid; eluent (4% ethyl acetate in hexane). **Yield:** 65% (59 mg); **^{1}H NMR (400 MHz, Chloroform-*d*) δ**: 7.77 – 7.73 (m, 2H), 7.45 – 7.42 (m, 1H), 7.35 – 7.30 (m, 4H), 7.29 – 7.22 (m, 2H), 7.17 (dd, *J* = 7.6, 1.7 Hz, 1H), 7.10 – 7.06 (m, 1H), 7.02 (d, *J* = 7.7 Hz, 1H), 3.23 – 3.20 (m, 2H), 2.72 – 2.66 (m, 6H), 2.44 (s, 3H), 2.20 – 2.16 (m, 4H). **^{13}C NMR (101 MHz, Chloroform-*d*) δ:** 150.7, 144.8, 139.8, 136.8, 134.0, 133.5, 132.0, 131.6, 130.0, 129.8, 129.1, 128.3, 128.2, 126.5, 122.8, 119.1, 53.7, 53.1, 51.4, 51.0, 21.8. **HRMS (ESI/TOF-Q) m/z:** $[M+H]^+$ Calculated for $C_{25}H_{27}ClN_2O_2SH^+$ 455.1555; Found 455.1556.

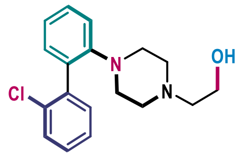

11e

Compound, **11e:** yellow sticky liquid; eluent (5% ethyl acetate in hexane). **Yield:** 83% (53 mg); **^{1}H NMR (400 MHz, Chloroform-*d*) δ**: 7.46 (d, *J* = 7.4 Hz, 1H), 7.38 – 7.33 (m, 2H), 7.31 – 7.24 (m, 2H), 7.19 (d, *J* = 7.5 Hz, 1H), 7.11 – 7.08 (m, 2H), 3.52 (t, *J* = 7.2 Hz, 2H), 2.87 – 2.84 (m, 4H), 2.67 – 2.63 (m, 2H), 2.33 – 2.31 (m, 4H). **^{13}C NMR (101 MHz, Chloroform-*d*) δ:** 150.9, 139.9, 134.0, 133.6, 132.1, 131.5, 129.8, 129.1, 128.3, 126.5, 122.7, 119.2, 60.0, 53.5, 51.2, 41.0. **HRMS (ESI/TOF-Q) m/z:** $[M+H]^+$ Calculated for $C_{18}H_{21}ClN_2OH^+$ 317.1415; Found 317.1410.

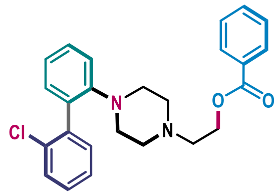

11f

Compound, **11f:** yellow sticky liquid; eluent (5% ethyl acetate in hexane). **Yield:** 58% (76 mg); **^{1}H NMR (400 MHz, Chloroform-*d*) δ**: 7.91 (d, *J* = 7.7 Hz, 2H), 7.46 (t, *J* = 7.4 Hz, 1H), 7.35 – 7.31 (m, 3H), 7.28 – 7.22 (m, 2H), 7.19 – 7.08 (m, 3H), 7.01 – 6.97 (m, 2H), 4.32 (t, *J* = 5.9 Hz, 2H), 2.77 (t, *J* = 4.9 Hz, 4H), 2.64 (t, *J* = 6.0 Hz, 2H), 2.37 – 2.29 (m, 4H). **^{13}C NMR (101 MHz, Chloroform-*d*) δ:** 166.6, 150.9, 139.9, 133.9, 133.5, 133.1, 132.0, 131.5, 130.3, 129.8, 129.7, 129.1, 128.5, 128.3, 126.5, 122.7, 119.2, 62.5, 56.7, 53.6, 51.2. **HRMS** (ESI/TOF-Q) m/z: $[M+H]^+$ Calculated for $C_{25}H_{25}ClN_2O_2H^+$ 421.1677; Found 421.1672.

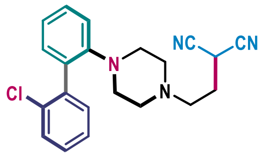

11g

Compound, **11g:** yellow sticky liquid; eluent (3% ethyl acetate in hexane). **Yield:** 60% (44 mg); **^{1}H NMR (400 MHz, Chloroform-*d*) δ**: 7.46 (d, *J* = 7.4 Hz, 1H), 7.38 – 7.33 (m, 2H), 7.31 – 7.26 (m, 2H), 7.19 (d, *J* = 7.4 Hz, 1H), 7.12 – 7.07 (m, 2H), 4.10 (t, *J* = 7.2 Hz, 1H), 2.83 (t, *J* = 4.9 Hz, 4H), 2.52 (t, *J* = 6.1 Hz, 2H), 2.31 – 2.25 (m, 4H), 2.12 (q, *J* = 6.5 Hz, 2H). **^{13}C NMR (101 MHz, Chloroform-*d*) δ:** 150.7, 139.9, 134.0, 133.6, 132.0, 129.8, 129.2, 128.3, 126.6, 122.9, 119.2, 113.0, 53.3 (2×C), 51.2, 28.5, 19.9. **HRMS (ESI/TOF-Q) m/z:** $[M+H]^+$ Calculated for $C_{21}H_{21}ClN_4H^+$ 365.1528; Found 365.1529.

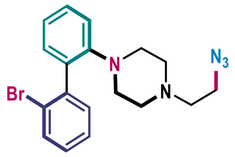

11h

Compound, **11h:** yellow sticky liquid; eluent (4% ethyl acetate in hexane). **Yield:** 39% (30 mg); **^{1}H NMR (400 MHz, Chloroform-*d*) δ**: 7.65 (d, *J* = 8.0 Hz, 1H), 7.38 – 7.31 (m, 3H), 7.19 – 7.15 (m, 2H), 7.11 – 7.07 (m, 2H), 3.29 (t, *J* = 6.1 Hz, 2H), 2.86 (t, *J* = 4.8 Hz, 4H), 2.51 (t, *J* = 6.1 Hz, 2H), 2.35 – 2.30 (m, 4H). **^{13}C NMR (101 MHz, Chloroform-*d*) δ:** 150.7, 141.9, 135.7, 133.0, 132.1, 131.5, 129.1, 128.4, 127.1, 124.1, 122.7, 119.2, 57.2, 53.5, 51.2, 48.4. **HRMS (ESI/TOF-Q) m/z:** $[M+H]^+$ Calculated for $C_{18}H_{20}BrN_5H^+$ 386.0975; Found 386.0980.

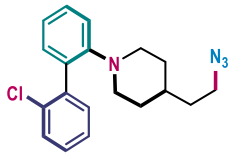

12a

Compound, **12a:** yellow sticky liquid; eluent (4% ethyl acetate in hexane). **Yield:** 67% (46 mg); **^{1}H NMR (400 MHz, Chloroform-*d*) δ:** 7.46 (dd, *J* = 7.4, 1.8 Hz, 1H), 7.39 – 7.31 (m, 2H), 7.31 – 7.27 (m, 1H), 7.25 – 7.23 (m, 1H), 7.18 (dd, *J* = 7.9, 1.7 Hz, 1H), 7.08 – 7.05 (m, 2H), 3.24 (t, *J* = 7.1 Hz, 2H), 3.04 – 3.00 (m, 2H), 2.60 – 2.52 (m, 2H), 1.51 – 1.44 (m, 4H), 1.40 – 1.30 (m, 1H), 1.02 – 0.87 (m, 2H). **^{13}C NMR (101 MHz, Chloroform-*d*) δ:** 151.9, 140.1, 134.0, 133.6, 132.1, 131.5, 129.7, 129.0, 128.1, 126.4, 122.3, 119.3, 52.1, 51.9, 49.1, 35.4, 33.2, 32.5, 32.3. **HRMS (ESI/TOF-Q) m/z:** $[M+H]^+$ Calculated for $C_{19}H_{21}ClN_4H^+$ 341.1528; Found 341.1525.

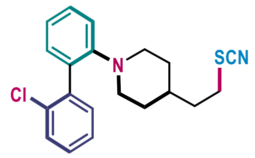

12b

Compound, **12b:** yellow sticky liquid; eluent (1% ethyl acetate in hexane). **Yield:** 65% (46 mg); **^{1}H NMR (400 MHz, Chloroform-*d*) δ**: 7.46 (dd, *J* = 7.4, 1.8 Hz, 1H), 7.38 – 7.35 (m, 1H), 7.33 – 7.27 (m, 2H), 7.26 – 7.22 (m, 1H), 7.21 – 7.17 (m, 1H), 7.09 – 7.06 (m, 2H), 3.05 – 3.02 (m, 2H), 2.90 (t, *J* = 7.6 Hz, 2H), 2.61 – 2.52 (m, 2H), 1.69 (q, *J* = 7.2 Hz, 2H), 1.53 – 1.49 (m, 2H), 1.46 – 1.34 (m, 1H), 1.05 – 0.88 (m, 2H). **^{13}C NMR (101 MHz, Chloroform-*d*) δ:** 151.7, 140.0, 134.1, 133.6, 132.0, 131.5, 129.7, 129.0, 128.2, 126.5, 122.4, 119.3, 112.3, 52.1, 51.8, 36.6, 34.5, 32.3, 32.1, 31.7. **HRMS (ESI/TOF-Q) m/z:** $[M+H]^+$ Calculated for $C_{20}H_{21}ClN_2SH^+$ 357.1187; Found 357.1188.

Synthesis of compound 13:

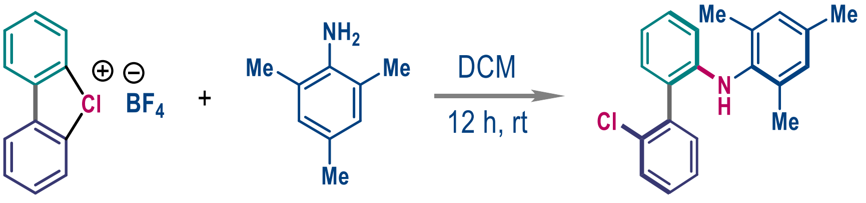

1a **13**

A 16x100 mm oven dried reaction tube equipped with a magnetic stir was charged with λ^3-chlorane **1a** (0.2 mmol, 1.0 equiv) and 2,4,6-trimethylaniline (0.2 mmol, 1.0 equiv) under N_2 atmosphere. Then, dry DCM (2 mL) was added via syringe. The reaction mixture was allowed to stir at rt for 12 h. After completion, volatiles were removed under reduced pressure and the crude product was purified by silica gel column chromatography to provide pure product **13.**

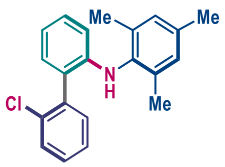

13

Compound, **13:** yellow sticky liquid; eluent (1% ethyl acetate in hexane). **Yield:** 65% (42 mg); **^{1}H NMR (400 MHz, Chloroform-*d*) δ**: 7.56 – 7.54 (m, 1H), 7.45 – 7.33 (m, 3H), 7.15 – 7.08 (m, 2H), 6.90 (s, 2H), 6.79 (t, *J* = 7.4 Hz, 1H), 6.22 (d, *J* = 8.1 Hz, 1H), 4.74 (s, 1H), 2.28 (s, 3H), 2.12 (s, 6H). **^{13}C NMR (101 MHz, Chloroform-*d*) δ**: 143.8, 138.2, 136.3, 135.6, 135.3, 134.7, 132.1, 130.17, 130.16, 129.32, 129.26, 129.2, 127.4, 125.2, 117.3, 111.5, 21.1, 18.3. **HRMS (ESI/TOF-Q) m/z:** $[M+H]^+$ Calculated for $C_{21}H_{20}ClNH^+$ 322.1357; Found 322.1348.

Ligand coupling reaction in the presence of radical quencher:

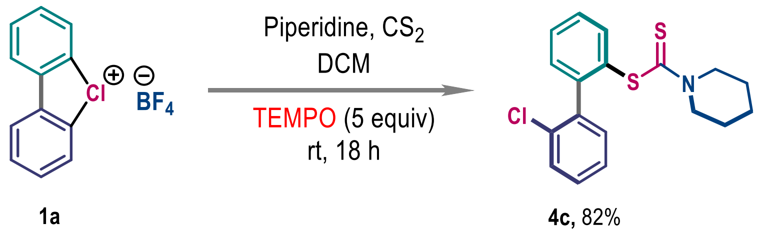

A 16x100 mm oven dried reaction tube equipped with a magnetic stir was charged with λ^3-chlorane **1a** (0.2 mmol, 1.0 equiv), CS_2 (**2a**, 2.5 equiv), piperidine (**3c**, 1.2 equiv), and TEMPO (5.0 equiv) under N_2 atmosphere. Then, dry DCM (2 mL) was added via syringe. The reaction mixture was allowed to stir at rt for 18 h. After completion, volatiles were removed under reduced pressure and the crude product was purified by silica gel column chromatography to provide pure product **4c** (57 mg, 82% yield).

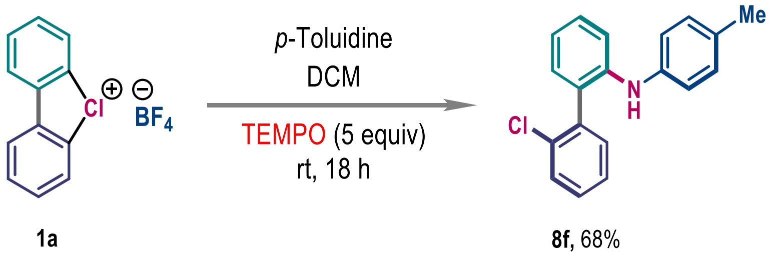

A 16x100 mm oven dried reaction tube equipped with a magnetic stir was charged with λ^3-chlorane **1a** (0.2 mmol, 1.0 equiv), *p*-toluidine (1.0 equiv), and TEMPO (5.0 equiv) under N_2 atmosphere. Then, dry DCM (2 mL) was added via syringe. The reaction mixture was allowed to stir at rt for 18 h. After completion, volatiles were removed under reduced pressure and the crude product was purified by silica gel column chromatography to provide pure product **8f** (40 mg, 68% yield).

Aryne trapping experiment:

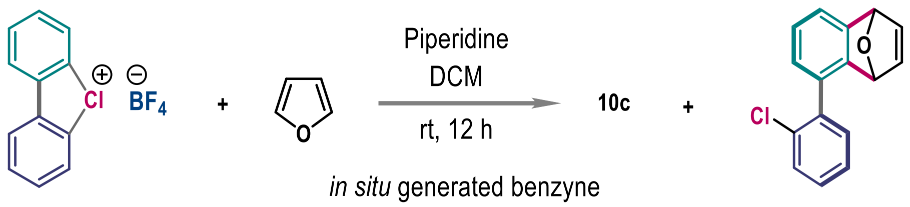

1a 14

A 16x100 mm oven dried reaction tube equipped with a magnetic stir was charged with λ^3-chlorane **1a** (0.2 mmol, 1.0 equiv) and furan **(**10.0 equiv) under N_2 atmosphere. Then, dry DCM (1.5 mL) was added via syringe. After that, piperidine (0.3 mmol, 1.5 equiv) was added slowly. The reaction mixture was allowed to stir at rt for 12 h. After completion of the reaction (TLC monitored), volatiles were removed under reduced pressure and the crude product was purified by silica gel chromatography to provide pure product **14.**

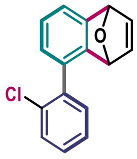

14

Compound, **14:** sticky liquid; eluent (1% ethyl acetate in hexane). **Yield:** 56% (29 mg); **^{1}H NMR (400 MHz, Chloroform-*d*) δ**: 7.52 – 7.48 (m, 1H), 7.36 – 7.30 (m, 2H), 7.29 – 7.23 (m, 2H), 7.16 – 7.13 (m, 1H), 7.10 – 7.04 (m, 2H), 6.95 (d, *J* = 7.8 Hz, 1H), 5.77 – 5.77 (m, 1H), 5.53 (s, 1H). **^{13}C NMR (101 MHz, Chloroform-*d*) δ**: 149.0, 148.5, 143.0, 138.6, 132.9, 132.5, 131.6, 129.9, 129.2, 127.0, 126.4, 125.2, 119.7, 82.7, 81.8. **HRMS (ESI/TOF-Q) m/z:** $[M+H]^+$ Calculated for $C_{16}H_{11}ClOH^+$ 255.0571; Found 255.0567.

Investigation of aryne formation during three-component ligand coupling reaction:

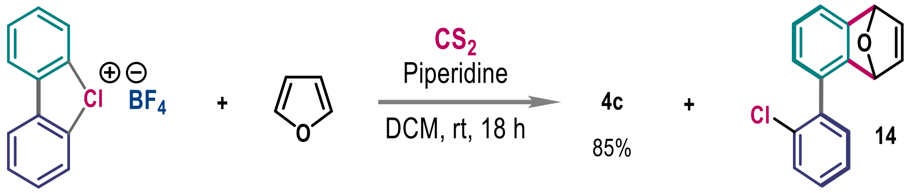

1a

A 16x100 mm oven dried reaction tube equipped with a magnetic stir was charged with λ^3-chlorane **1a** (0.2 mmol, 1.0 equiv), CS_2 (**2a**, 2.5 equiv), piperidine (**3c**, 1.2 equiv), and furan (10.0 equiv) under N_2 atmosphere. Then, dry DCM (2 mL) was added via syringe. The reaction mixture was allowed to stir at rt for 18 h. After completion of the reaction (TLC monitored), volatiles were removed under reduced pressure and the crude product was purified by silica gel chromatography to provide pure product **4c.** We did not observe the formation of compound **14**.

Crystallographic experimental section

Crystallization: Crystals of compound **4e** were obtained through slow evaporation technique at room temperature from a solution in hexane/DCM solvent combinations.

Crystal structure of compound **4e (CCDC number: 2340860,** Ellipsoid Probability 50%):

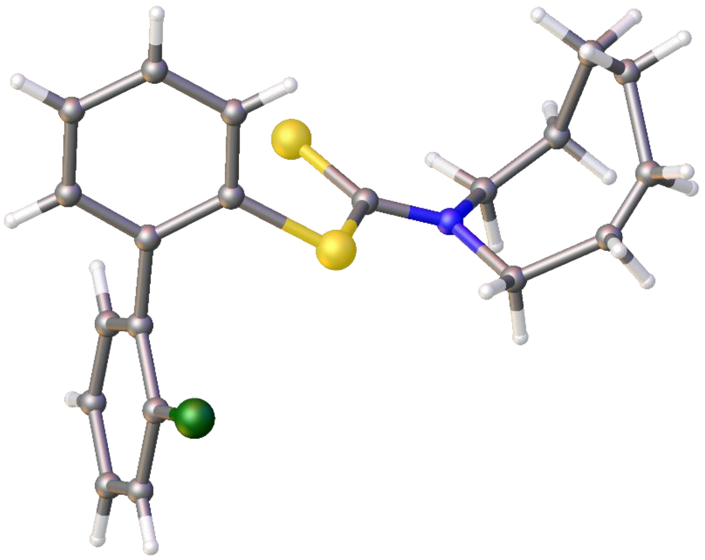

Identification code	**4e**
Empirical formula	$C_{20} H_{22} Cl N S_2$
Formula weight	375.95
Temperature	300(2) K
Wavelength	0.71073 A
Crystal system, space group	Monoclinic, P 21/c
Unit cell dimensions	a = 9.7904(14) A alpha = 90 deg.<br>b = 13.791(2) A beta = 99.606(5) deg.<br>c = 14.347(2) A gamma = 90 deg.
Volume	1909.9(5) A^3

Z, Calculated density	4, 1.307 Mg/m^3
Absorption coefficient	0.420 mm^-1
F(000)	792
Crystal size	0.241 x 0.147 x 0.092 mm
Theta range for data collection	3.118 to 26.344 deg.
Limiting indices	-12<=h<=12, -17<=k<=17, -17<=l<=17
Reflections collected / unique	55860 / 3879 [R(int) = 0.0831]
Completeness to theta = 25.242	99.7 %
Absorption correction	Semi-empirical from equivalents
Max. and min. transmission	0.7454 and 0.5420
Refinement method	Full-matrix least-squares on F^2
Data / restraints / parameters	3879 / 0 / 263
Goodness-of-fit on F^2	1.082
Final R indices [I>2sigma(I)]	R1 = 0.0620, wR2 = 0.1506
R indices (all data)	R1 = 0.0810, wR2 = 0.1619
Extinction coefficient	n/a
Largest diff. peak and hole	0.393 and -0.348 e.A^-3

Crystallization: Crystals of compound **8i** obtained through slow evaporation technique at room temperature from a solution in hexane/acetonitrile solvent combinations.

Crystal structure of compound **8i (CCDC number: 2354443,** Ellipsoid Probability 50%):

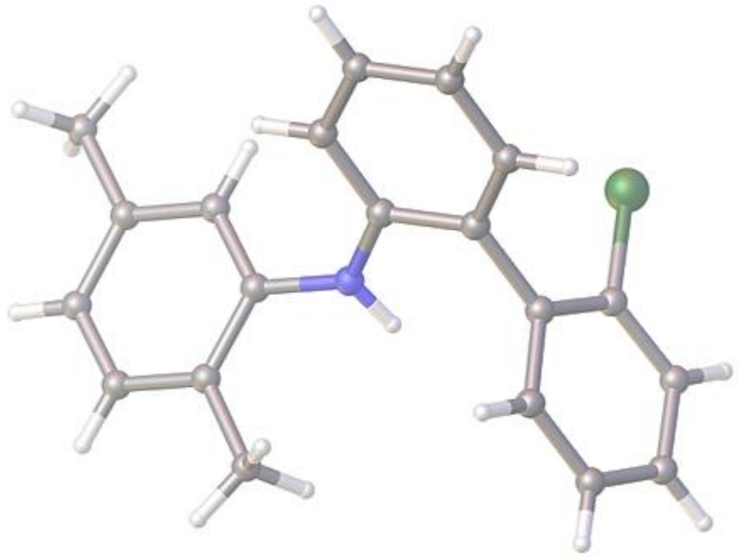

Identification code	**8i**
Empirical formula	$C_{20} H_{18} Cl N$
Formula weight	307.80
Temperature	296(2) K
Wavelength	0.71073 A
Crystal system, space group	Monoclinic, C2/c
Unit cell dimensions	a = 17.9856(9) A alpha = 90 deg.<br>b = 7.6368(3) A beta = 92.914(2) deg.<br>c = 23.5135(13) A gamma = 90 deg.
Volume	3225.5(3) A^3
Z, Calculated density	8, 1.268 Mg/m^3
Absorption coefficient	0.233 mm^-1

F(000)	1296
Crystal size	0.320 x 0.260 x 0.120 mm
Theta range for data collection	1.734 to 24.999 deg.
Limiting indices	-20<=h<=21, -9<=k<=7, -27<=l<=27
Reflections collected / unique	9434 / 2841 [R(int) = 0.0230]
Completeness to theta = 24.999	100.0 %
Absorption correction	None
Refinement method	Full-matrix least-squares on F^2
Data / restraints / parameters	2841 / 0 / 202
Goodness-of-fit on F^2	1.049
Final R indices [I>2sigma(I)]	R1 = 0.0449, wR2 = 0.1142
R indices (all data)	R1 = 0.0579, wR2 = 0.1235
Extinction coefficient	0.0024(3)
Largest diff. peak and hole	0.435 and -0.333 e.A^-3

NMR spectra of synthesized compounds

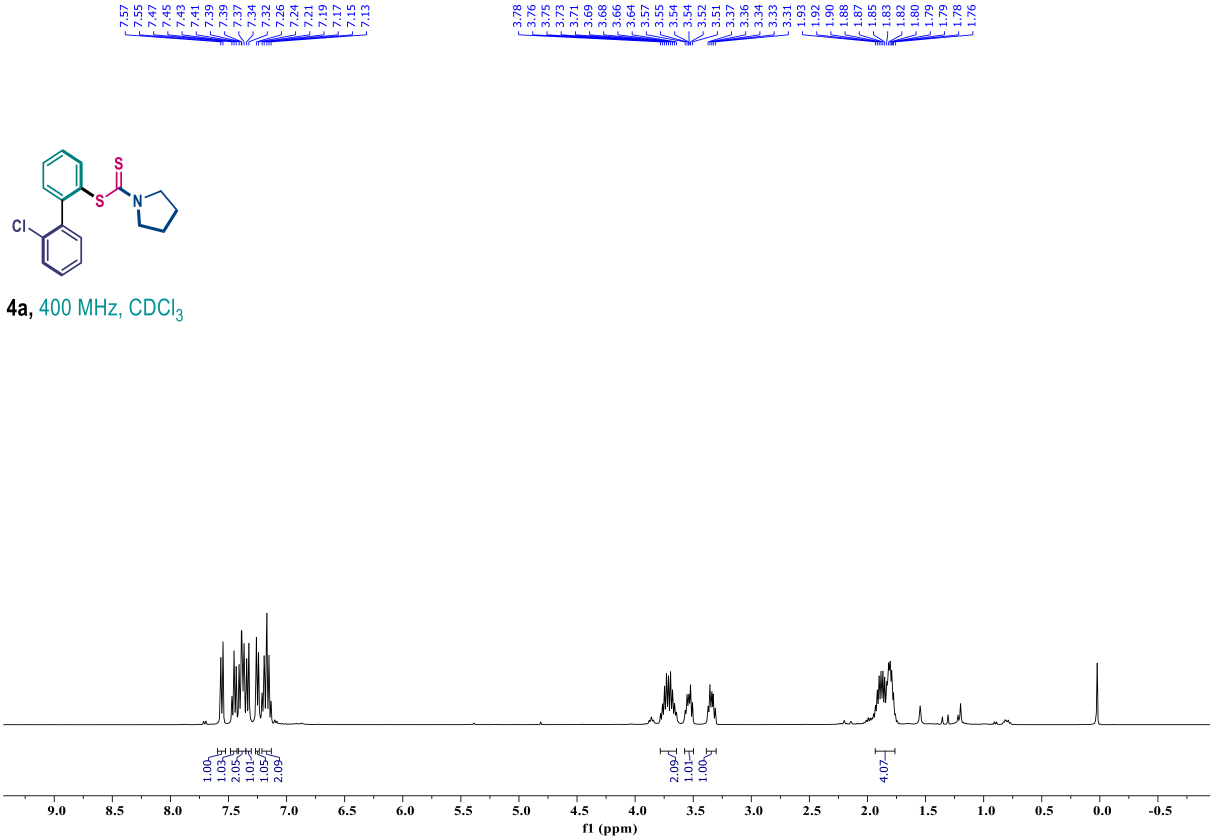

4a, 400 MHz, $CDCl_3$

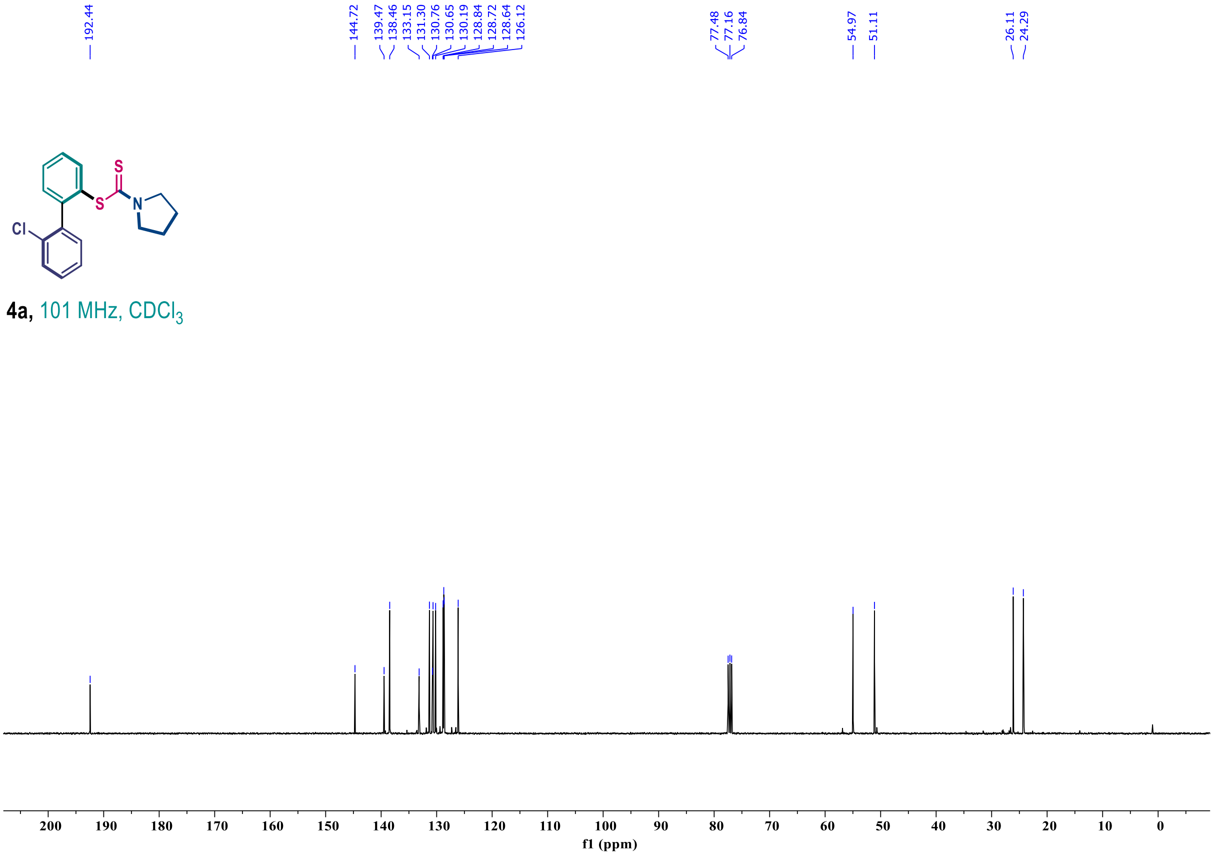

4a, 101 MHz, $CDCl_3$

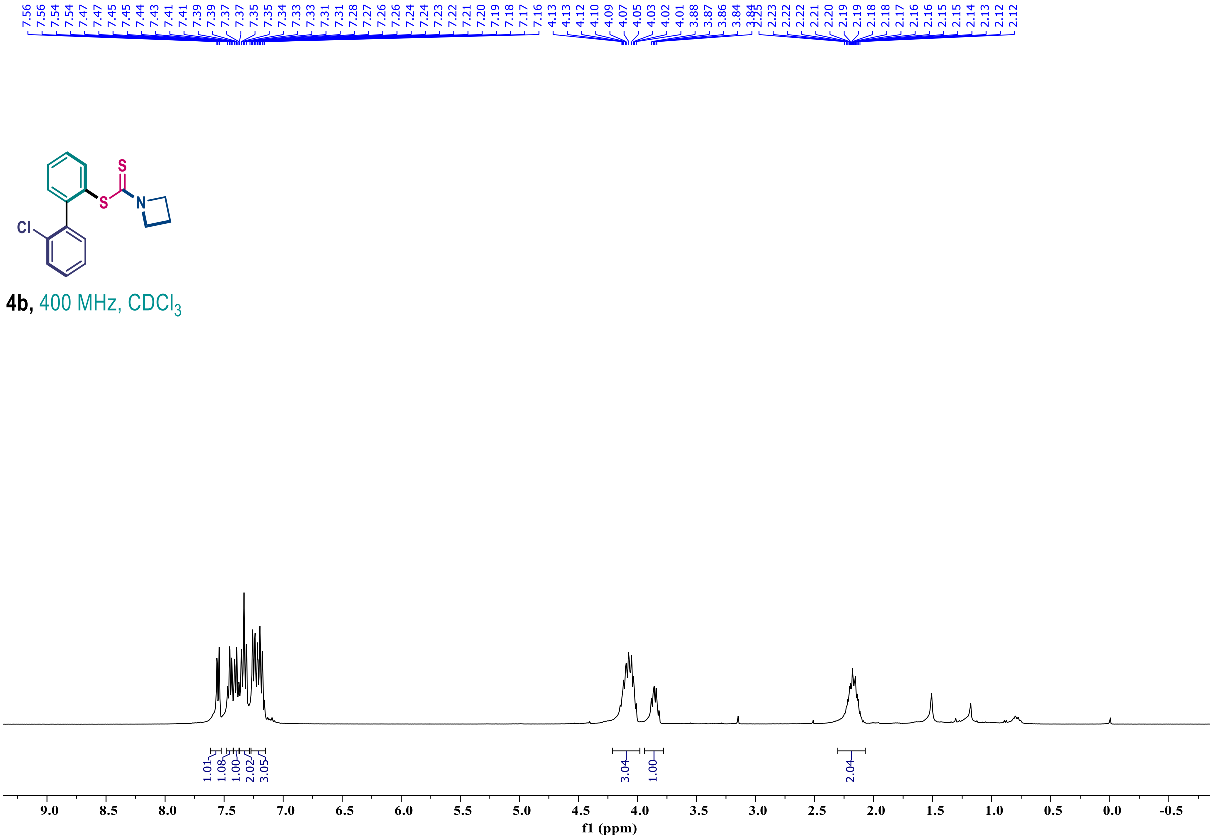

4b, 400 MHz, $CDCl_3$

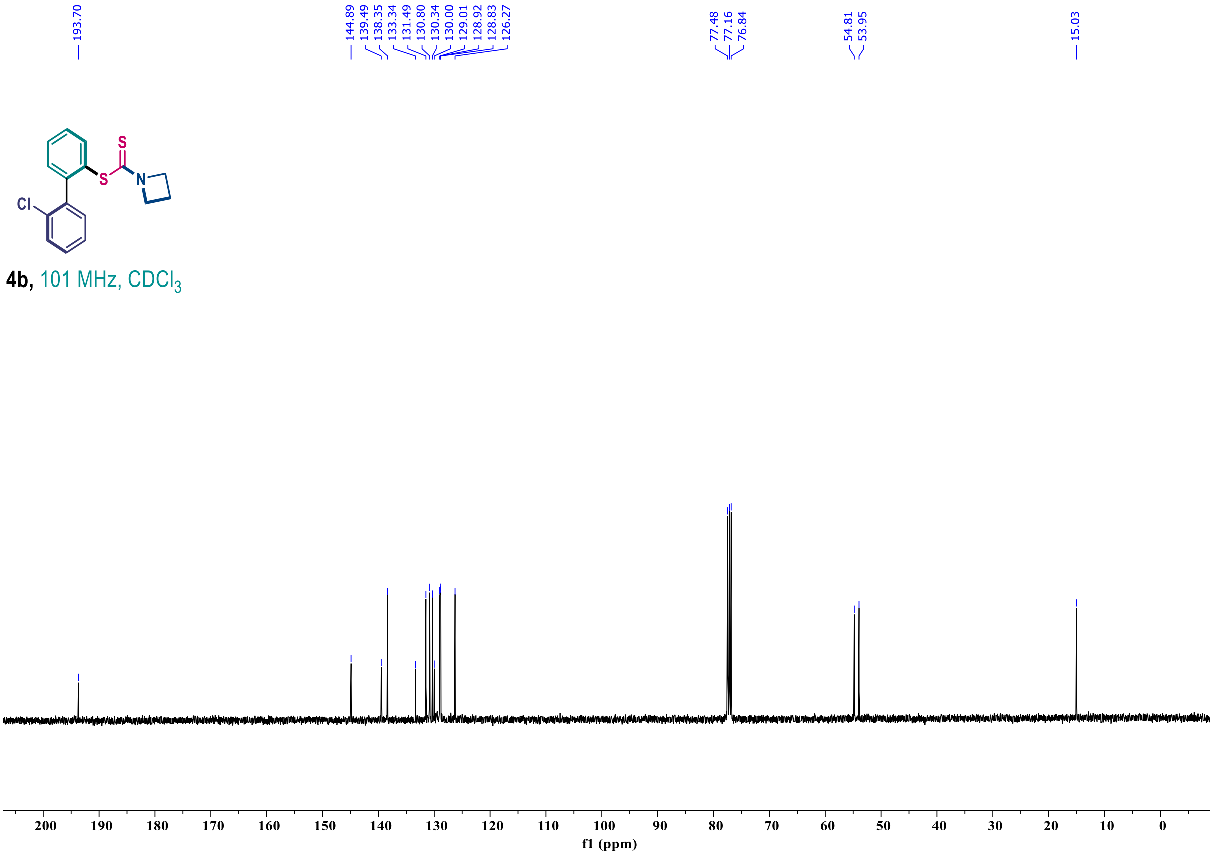

4b, 101 MHz, $CDCl_3$

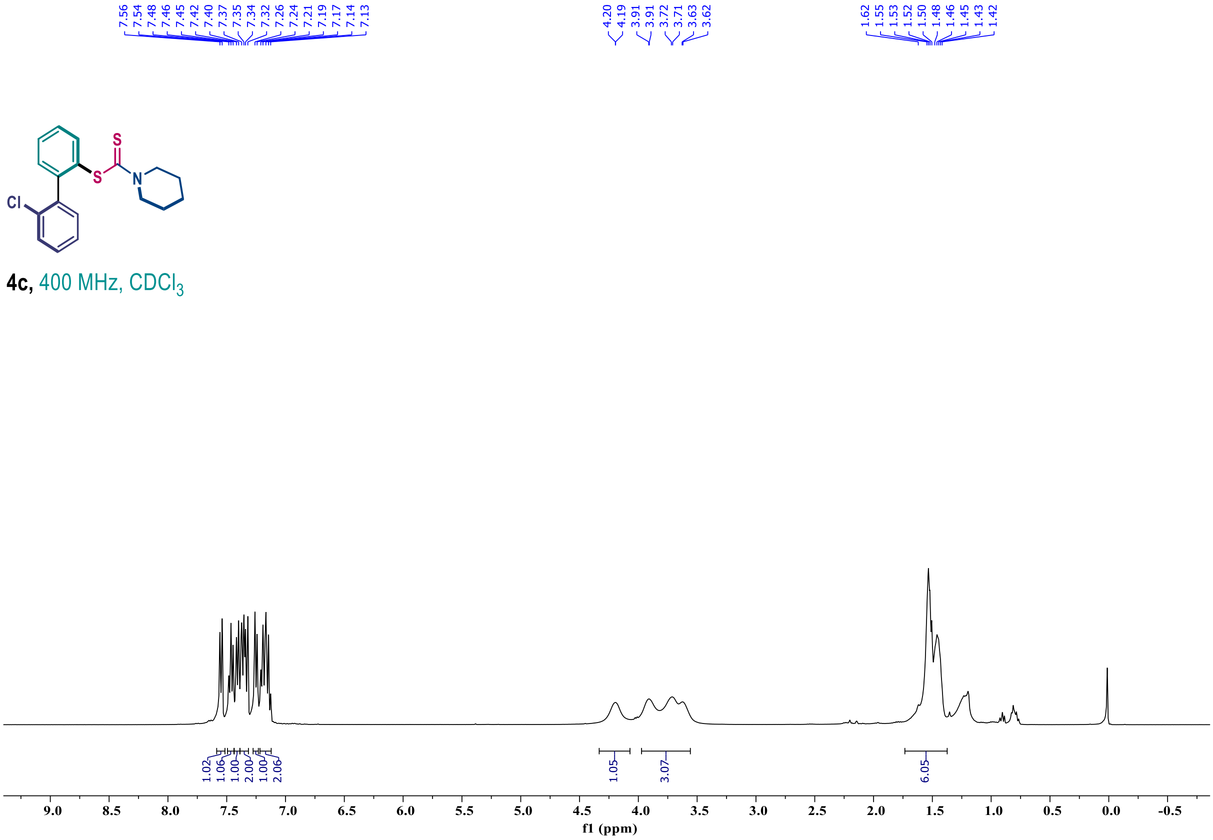
7.56 7.54 7.48 7.46 7.45 7.42 7.40 7.37 7.35 7.34 7.32 7.26 7.24 7.21 7.19 7.17 7.14 7.13
4.20 4.19 3.91 3.91 3.72 3.71 3.63 3.62
1.62 1.55 1.53 1.52 1.50 1.48 1.46 1.45 1.43 1.42
S
S
N
Cl
4c, 400 MHz, CDCl3
1.02 1.06 1.00 2.00 1.00 2.06
1.05
3.07
6.05
f1 (ppm)

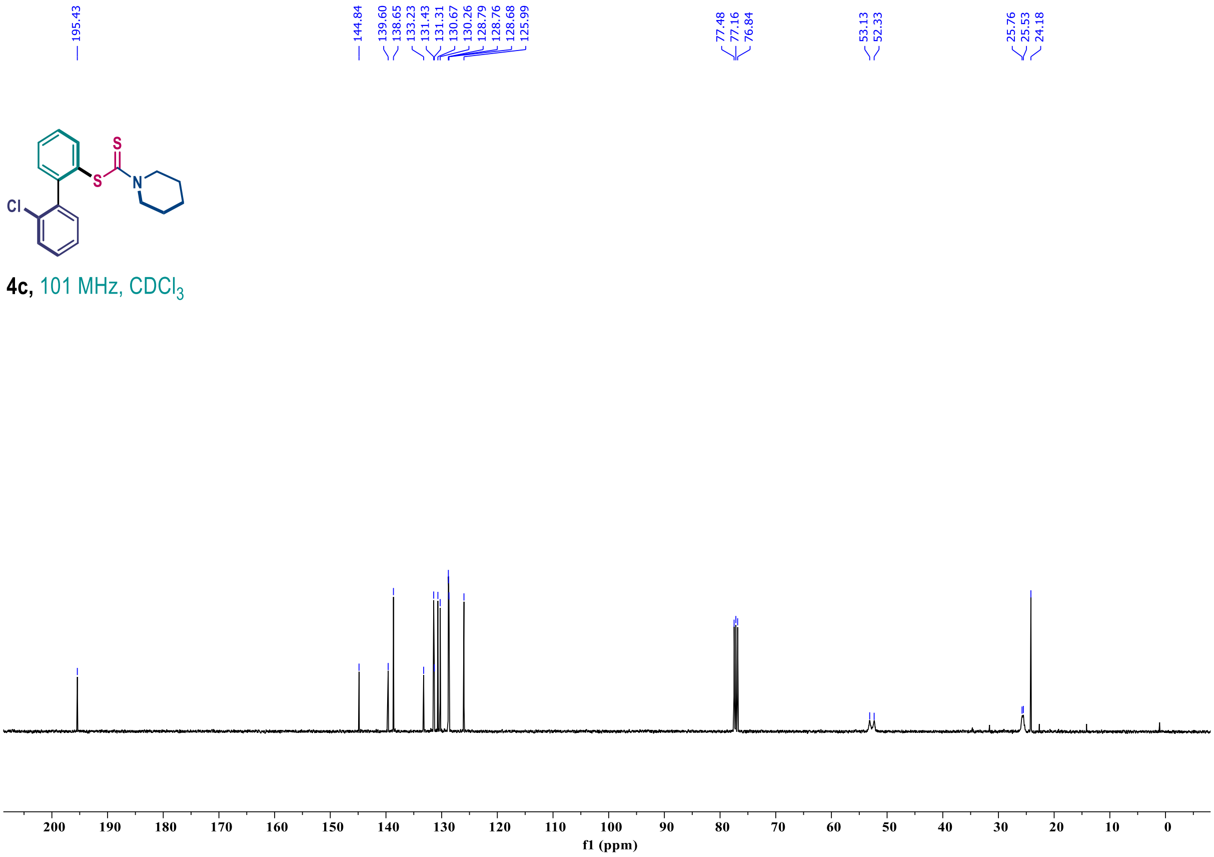
195.43
144.84 139.60 138.65 133.23 131.43 131.31 130.67 130.26 128.79 128.76 128.68 125.99
77.48 77.16 76.84
53.13 52.33
25.76 25.53 24.18
S
S
N
Cl
4c, 101 MHz, CDCl3
f1 (ppm)

4d, 400 MHz, $CDCl_3$

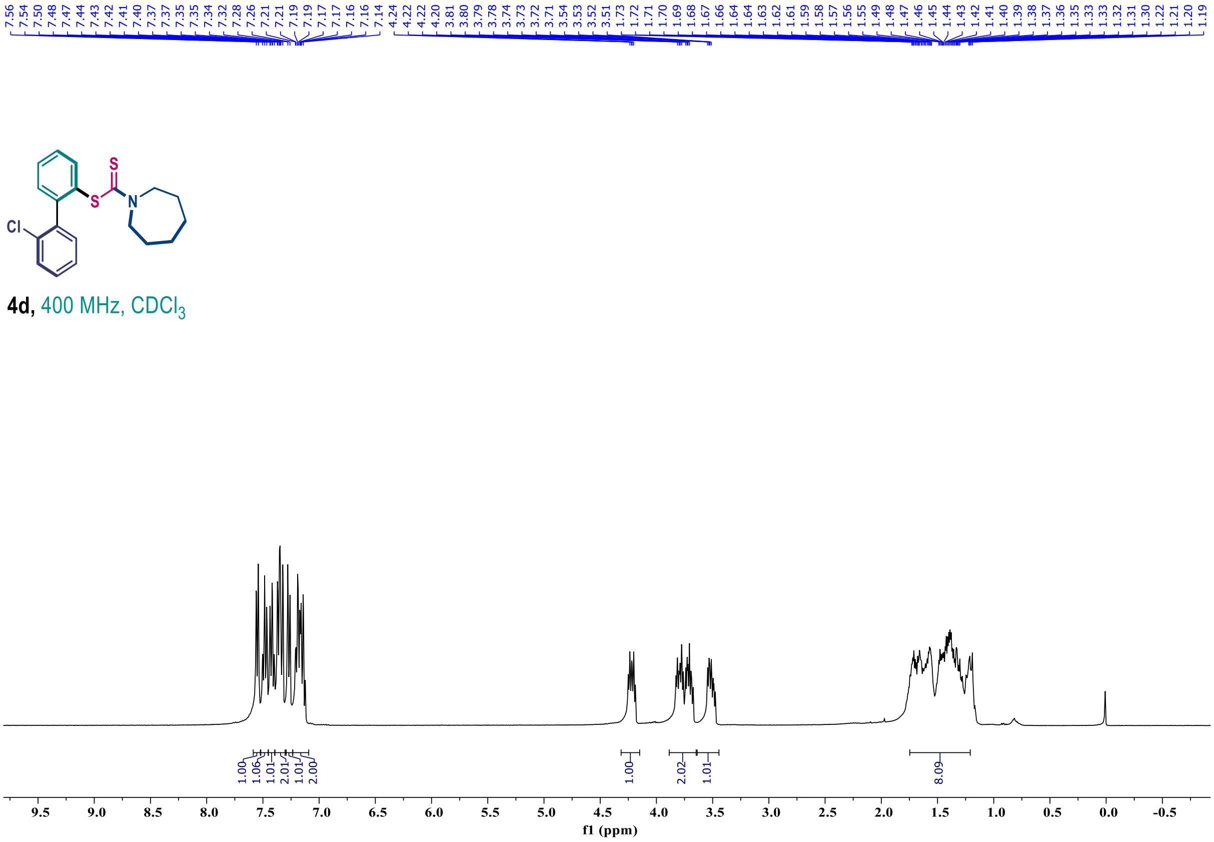

4d, 101 MHz, $CDCl_3$

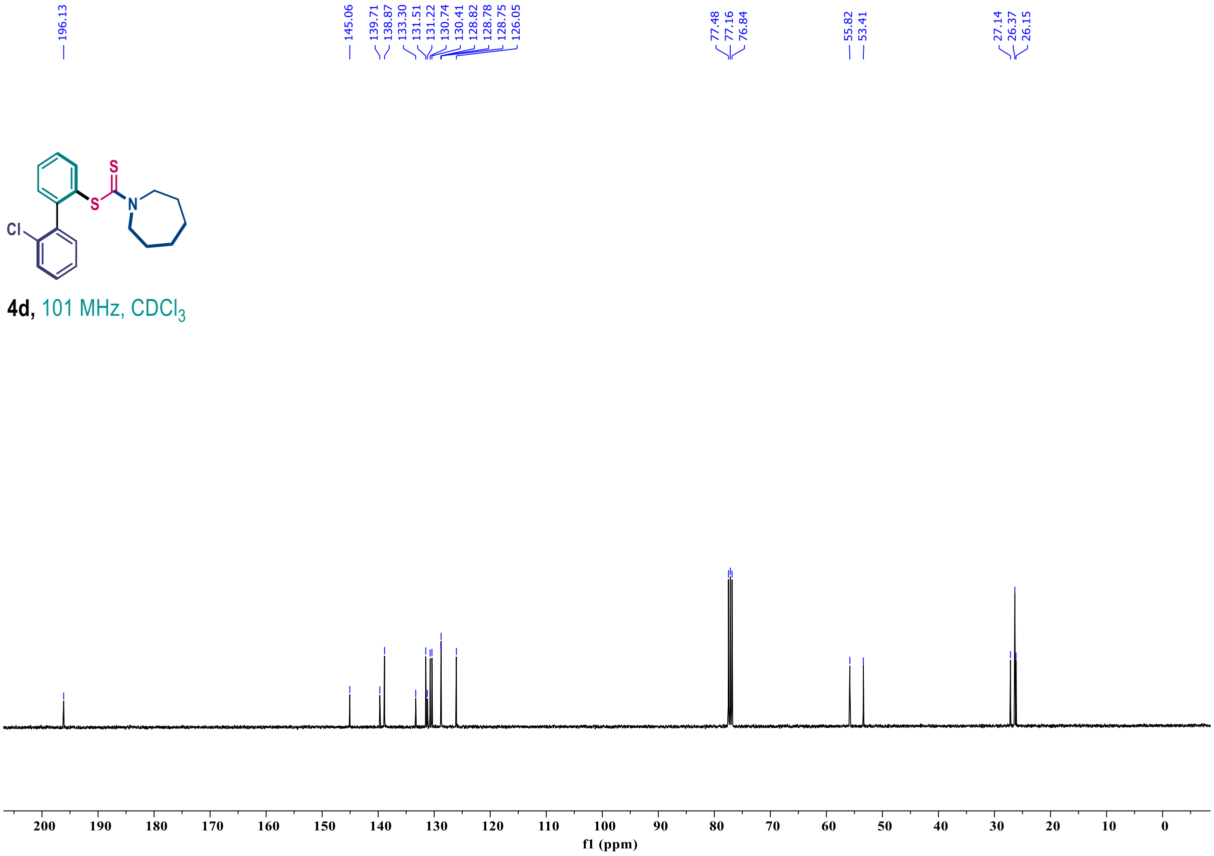

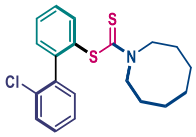

4e, 400 MHz, $CDCl_3$

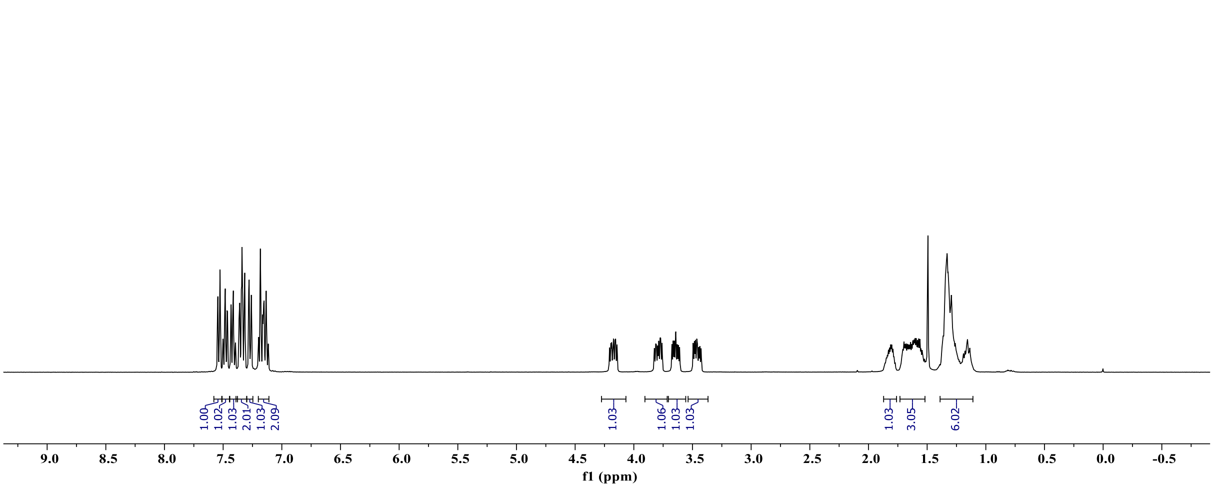

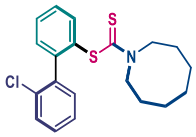

4e, 101 MHz, $CDCl_3$

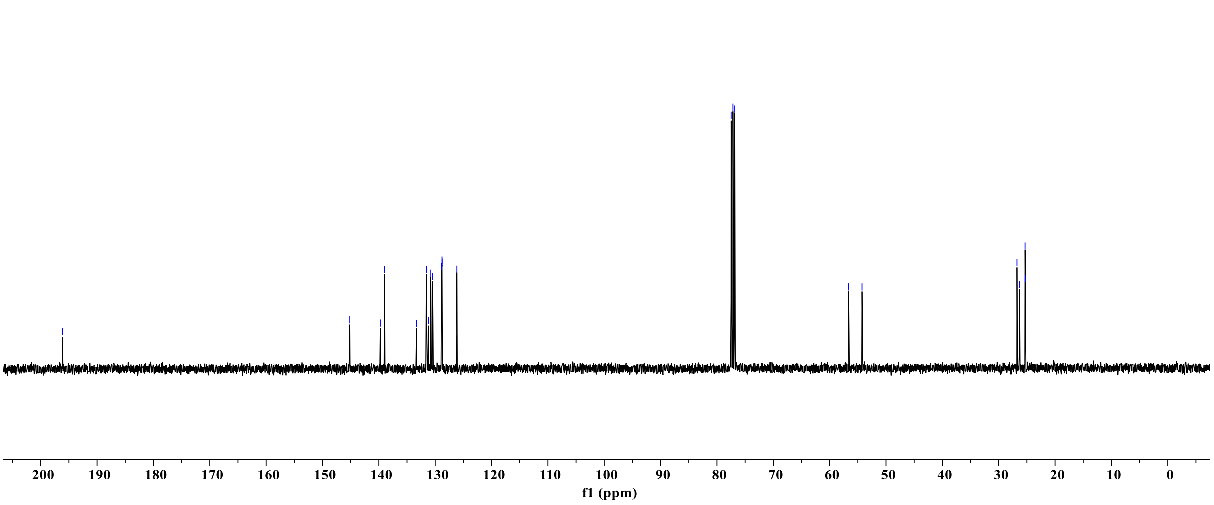

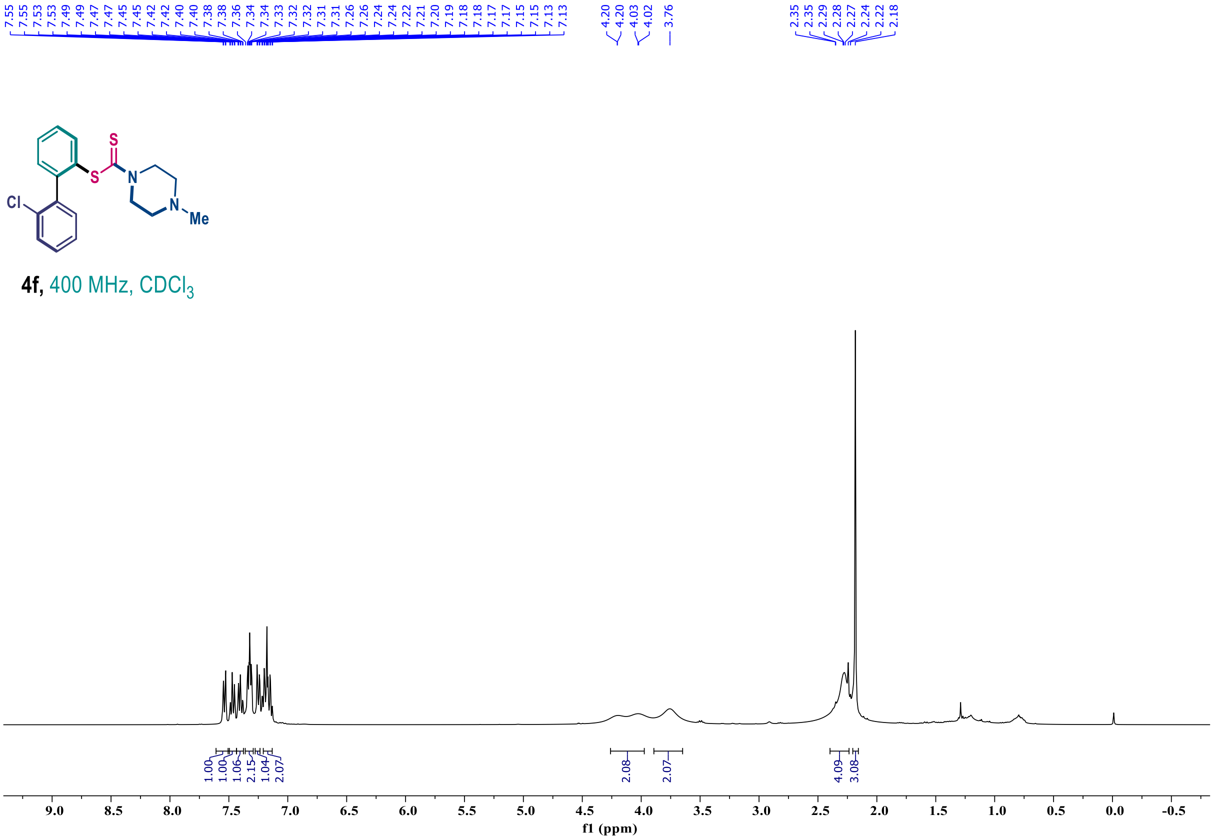
4f, 400 MHz, CDCl3
f1 (ppm)

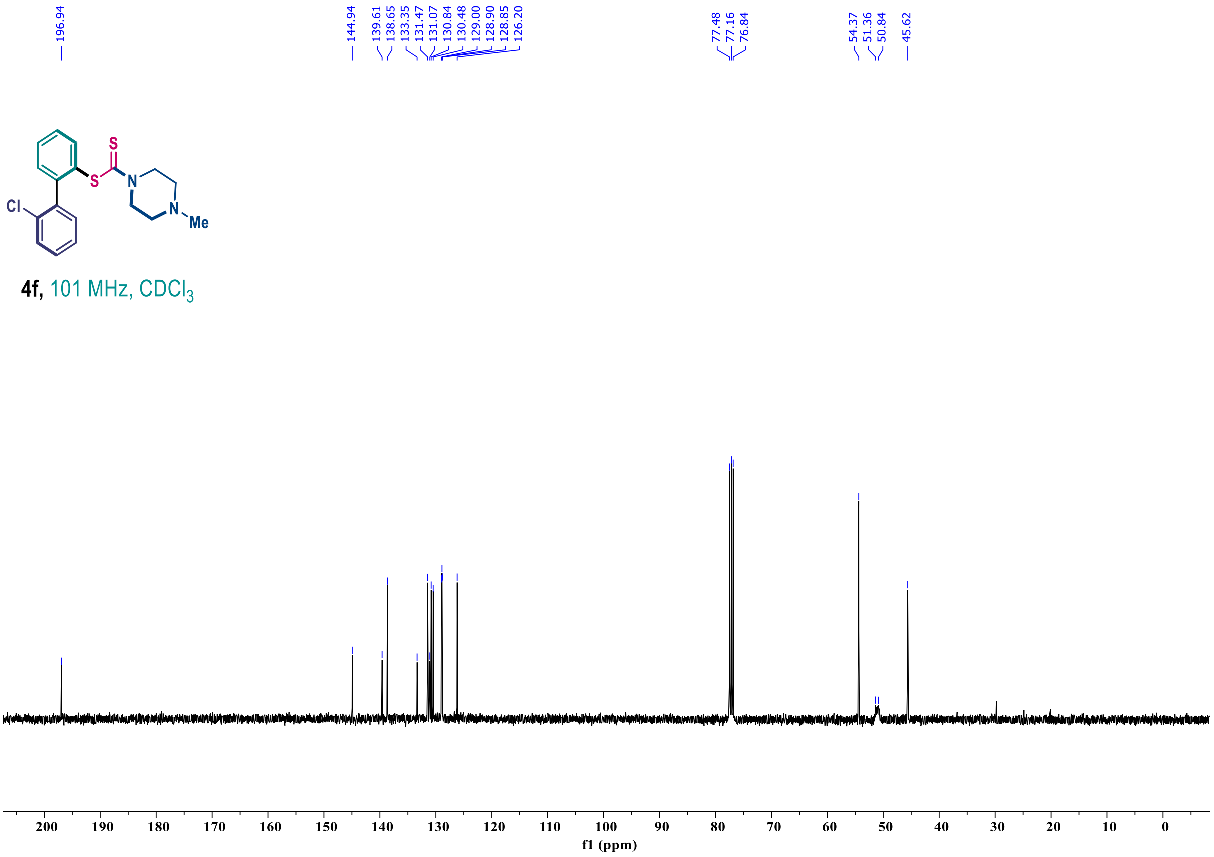
4f, 101 MHz, CDCl3
f1 (ppm)

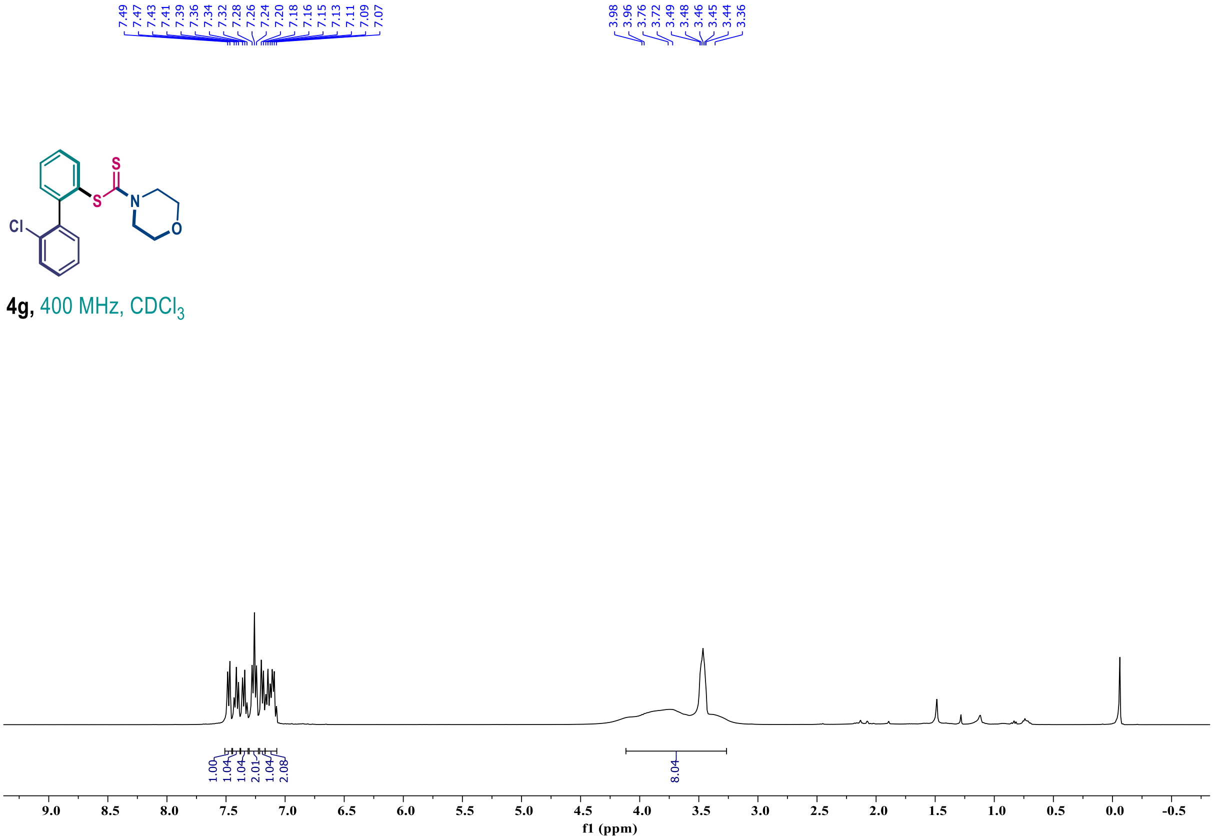

4g, 400 MHz, $CDCl_3$

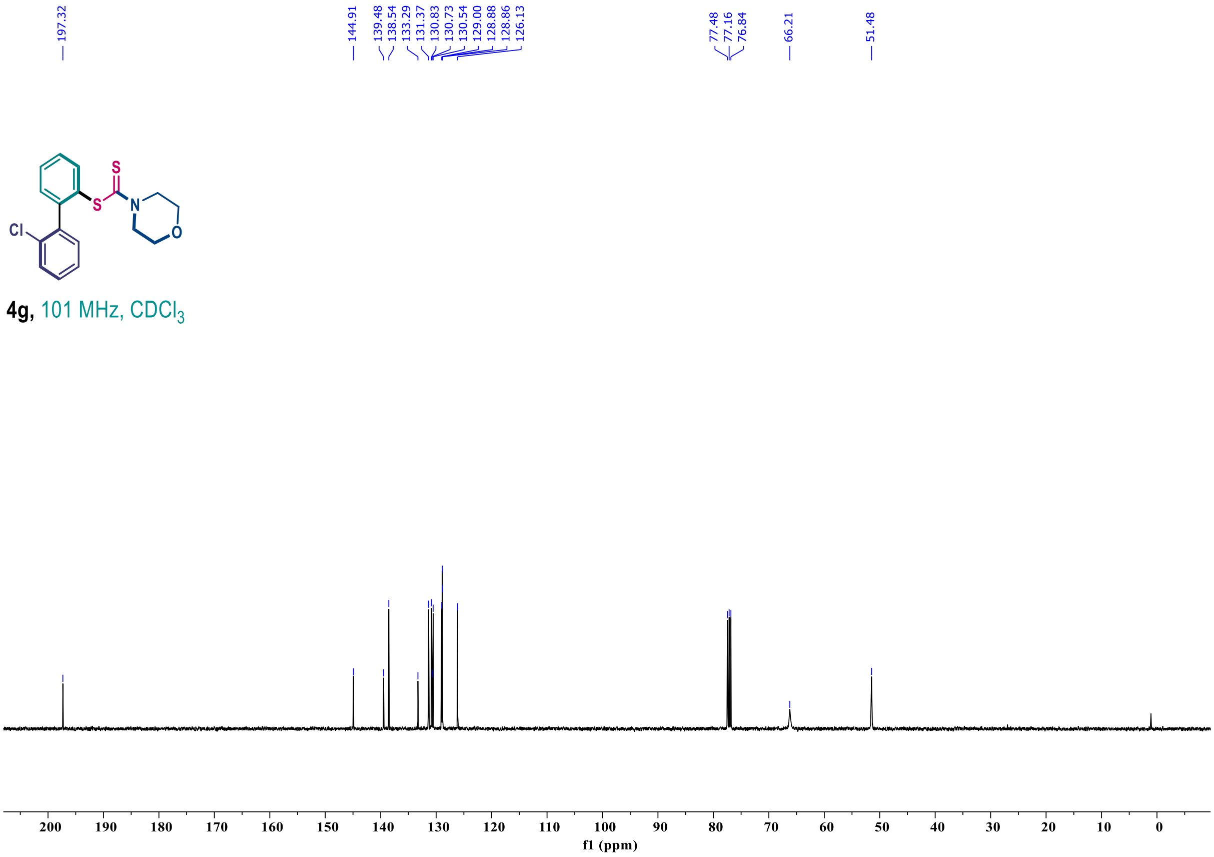

4g, 101 MHz, $CDCl_3$

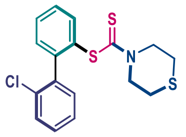

4h, 400 MHz, $CDCl_3$

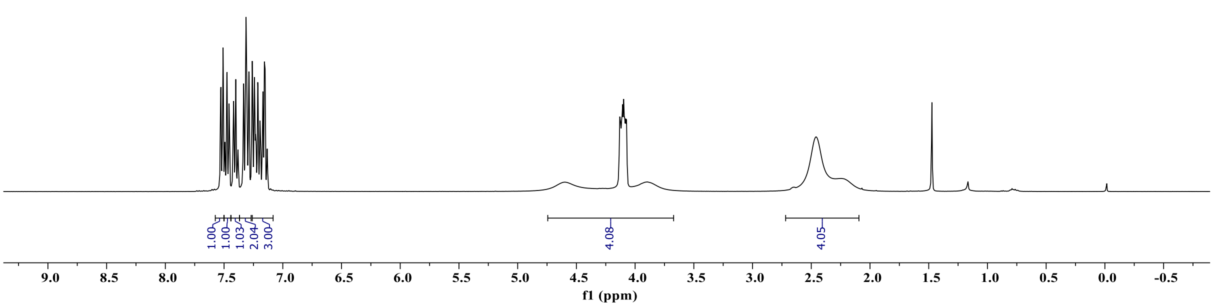

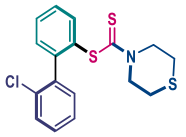

4h, 101 MHz, $CDCl_3$

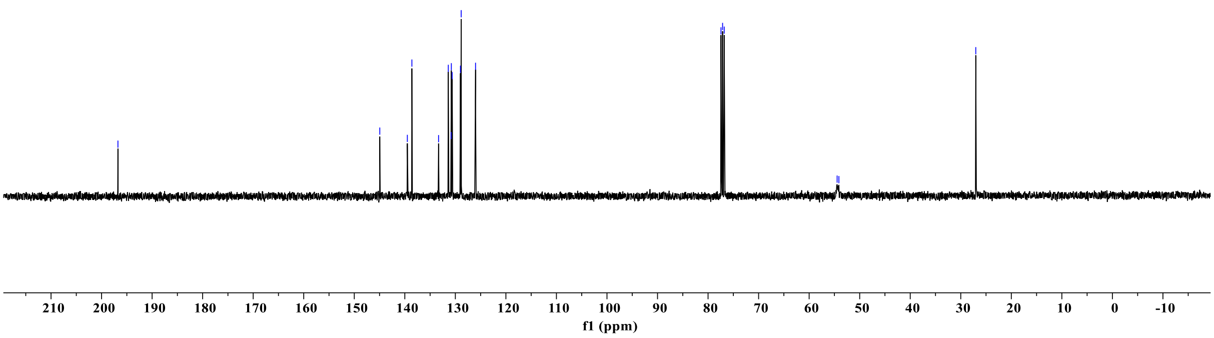

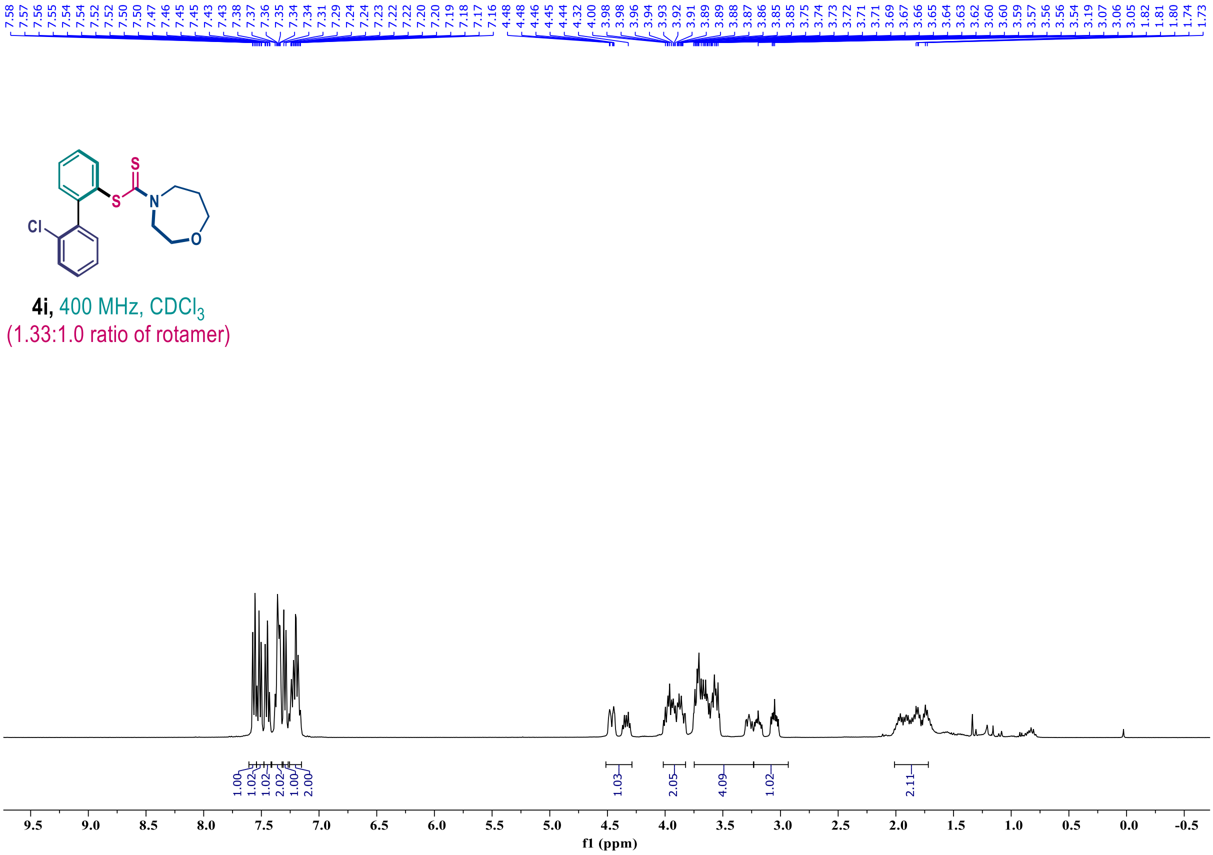

4i, 400 MHz, $CDCl_3$
(1.33:1.0 ratio of rotamer)

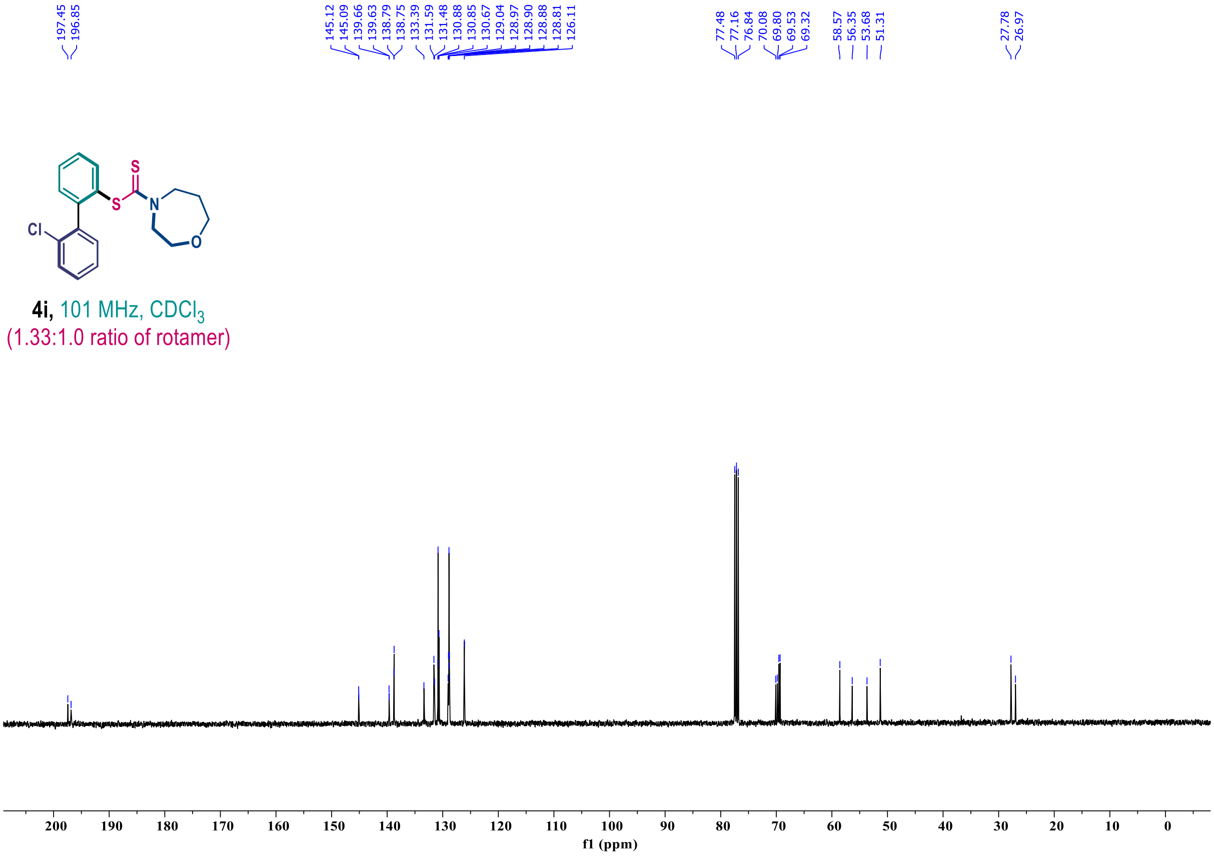

4i, 101 MHz, $CDCl_3$
(1.33:1.0 ratio of rotamer)

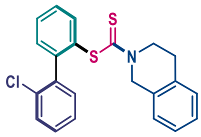

4j, 400 MHz, $CDCl_3$
(1.5:1.0 ratio of rotamer)

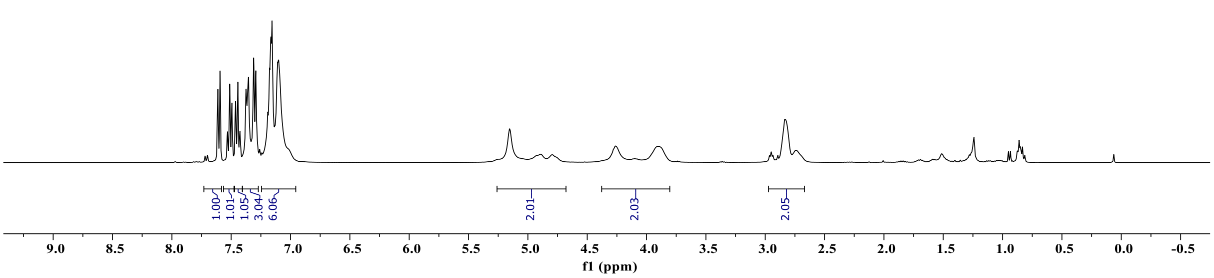

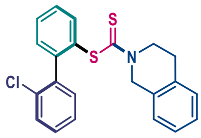

4j, 101 MHz, $CDCl_3$
(1.5:1.0 ratio of rotamer)

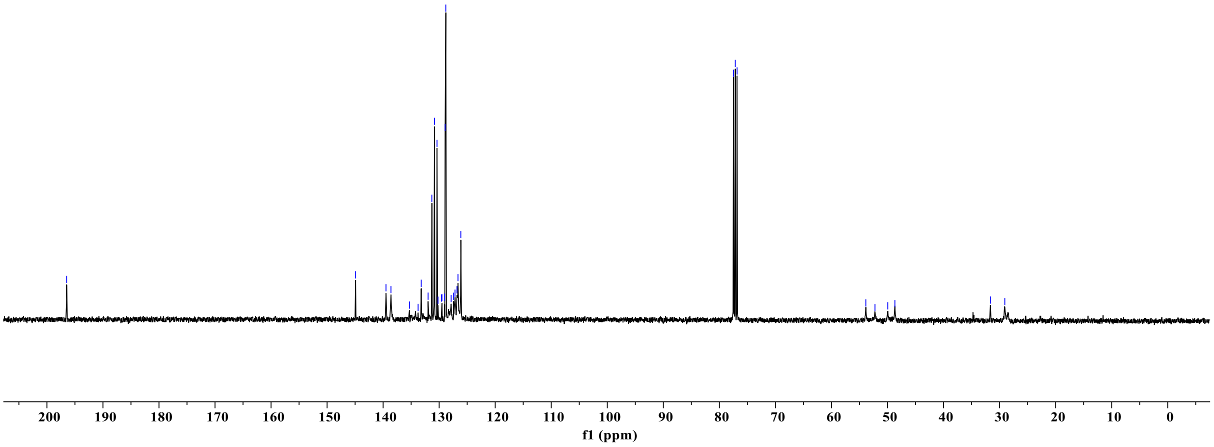

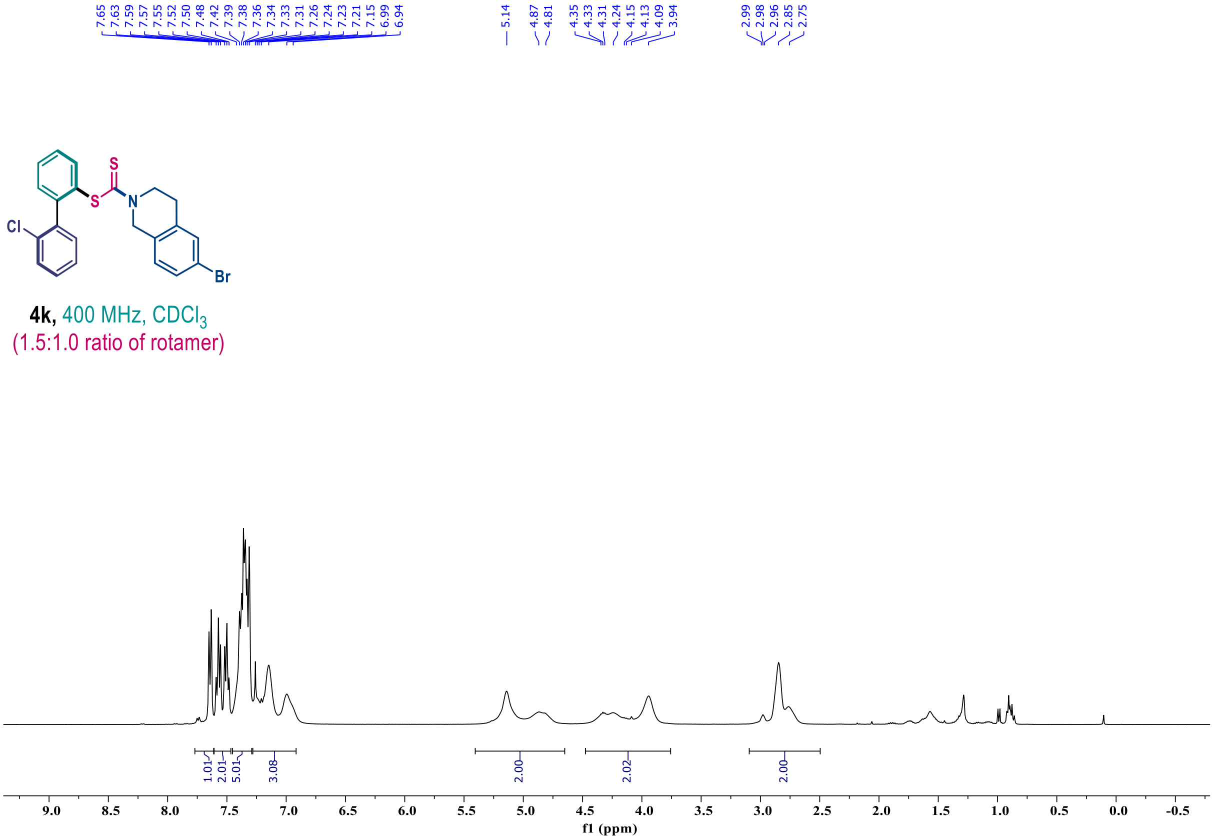
7.65 7.63 7.59 7.57 7.55 7.52 7.50 7.48 7.42 7.39 7.38 7.36 7.34 7.33 7.31 7.26 7.24 7.23 7.21 7.15 6.99 6.94
5.14 4.87 4.81 4.35 4.33 4.31 4.24 4.15 4.13 4.09 3.94
2.99 2.98 2.96 2.85 2.75
S
Cl
S
N
Br
4k, 400 MHz, CDCl3
(1.5:1.0 ratio of rotamer)
1.01 2.01 5.01 3.08 2.00 2.02 2.00
9.0 8.5 8.0 7.5 7.0 6.5 6.0 5.5 5.0 4.5 4.0 3.5 3.0 2.5 2.0 1.5 1.0 0.5 0.0 -0.5
f1 (ppm)

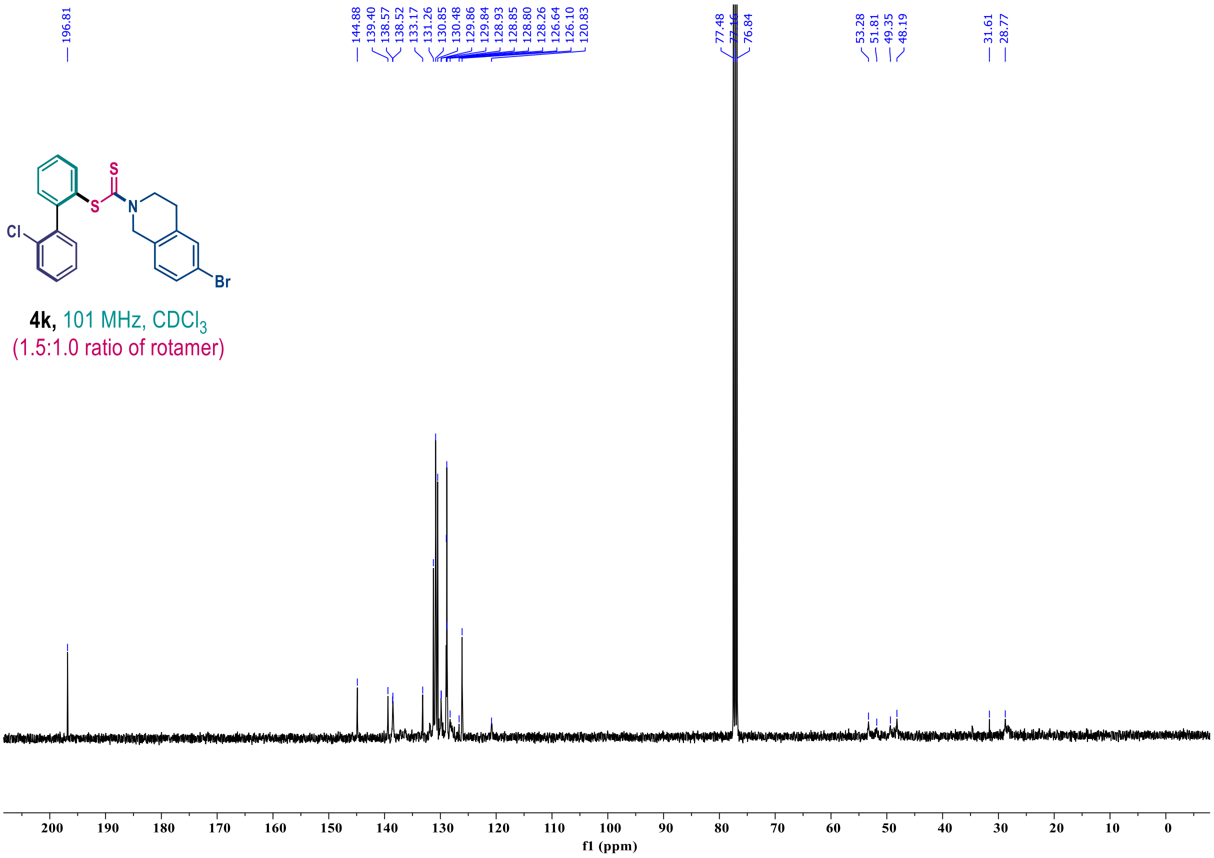
196.81
144.88 139.40 138.57 138.52 133.17 131.26 130.85 130.48 129.86 129.84 128.93 128.85 128.80 128.26 126.64 126.10 120.83
77.48 77.16 76.84
53.28 51.81 49.35 48.19
31.61 28.77
S
Cl
S
N
Br
4k, 101 MHz, CDCl3
(1.5:1.0 ratio of rotamer)
200 190 180 170 160 150 140 130 120 110 100 90 80 70 60 50 40 30 20 10 0
f1 (ppm)

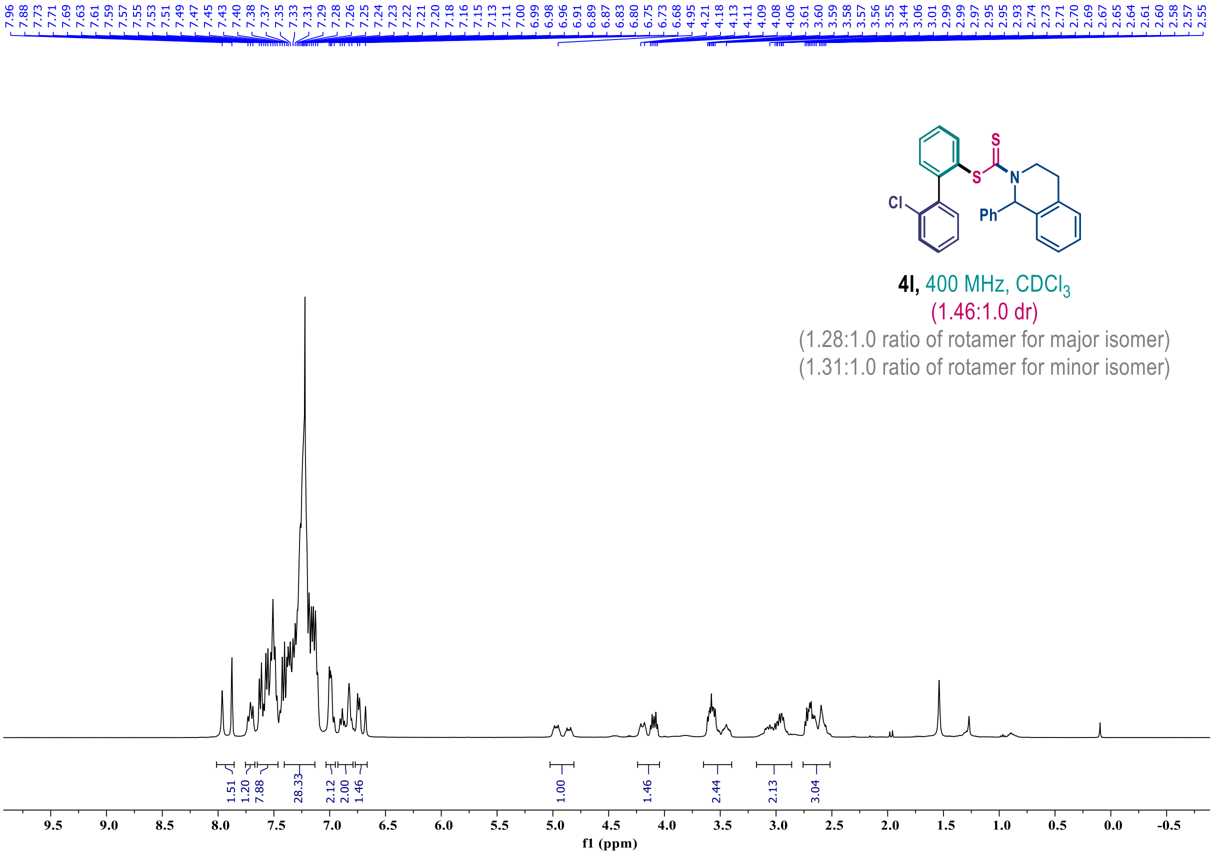
7.96 7.88 7.73 7.71 7.69 7.63 7.61 7.59 7.57 7.55 7.53 7.51 7.49 7.47 7.45 7.43 7.40 7.38 7.37 7.35 7.33 7.31 7.29 7.28 7.26 7.25 7.24 7.23 7.22 7.21 7.20 7.18 7.16 7.15 7.13 7.11 7.00 6.99 6.98 6.96 6.91 6.89 6.87 6.83 6.80 6.75 6.73 6.68 4.95 4.21 4.18 4.13 4.11 4.09 4.08 4.06 3.61 3.60 3.59 3.58 3.57 3.56 3.55 3.44 3.06 3.01 2.99 2.99 2.97 2.95 2.95 2.93 2.74 2.73 2.71 2.70 2.69 2.67 2.65 2.64 2.61 2.60 2.58 2.57 2.55
S
N
S
Cl
Ph
4l, 400 MHz, CDCl3
(1.46:1.0 dr)
(1.28:1.0 ratio of rotamer for major isomer)
(1.31:1.0 ratio of rotamer for minor isomer)
1.51 1.20 7.88 28.33 2.12 2.00 1.46 1.00 1.46 2.44 2.13 3.04
9.5 9.0 8.5 8.0 7.5 7.0 6.5 6.0 5.5 5.0 4.5 4.0 3.5 3.0 2.5 2.0 1.5 1.0 0.5 0.0 -0.5
f1 (ppm)

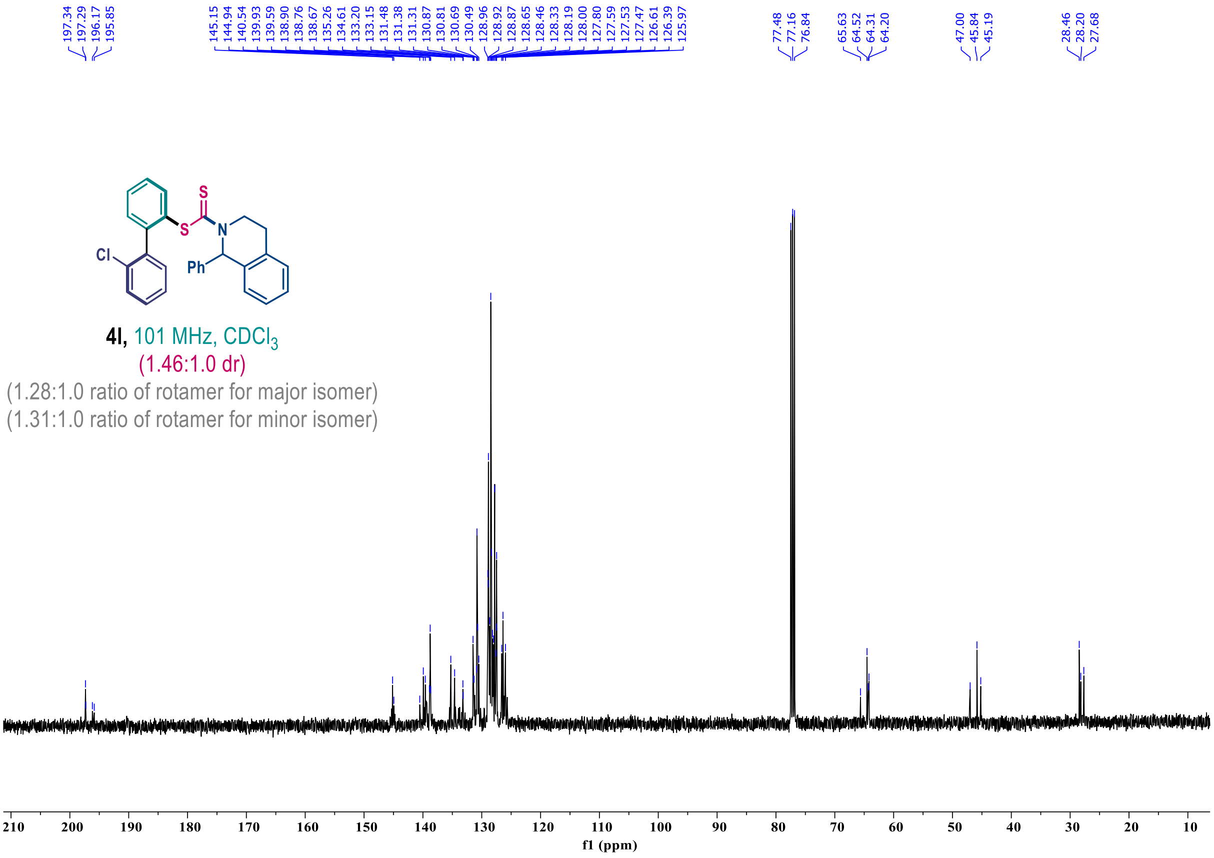
197.34 197.29 196.17 195.85
145.15 144.94 140.54 139.93 139.59 138.90 138.76 138.67 135.26 134.61 133.20 133.15 131.48 131.38 131.31 130.87 130.81 130.69 130.49 128.96 128.92 128.87 128.65 128.46 128.33 128.19 128.00 127.80 127.59 127.53 127.47 126.61 126.39 125.97
77.48 77.16 76.84
65.63 64.52 64.31 64.20
47.00 45.84 45.19
28.46 28.20 27.68
S
N
S
Cl
Ph
4l, 101 MHz, CDCl3
(1.46:1.0 dr)
(1.28:1.0 ratio of rotamer for major isomer)
(1.31:1.0 ratio of rotamer for minor isomer)
210 200 190 180 170 160 150 140 130 120 110 100 90 80 70 60 50 40 30 20 10
f1 (ppm)

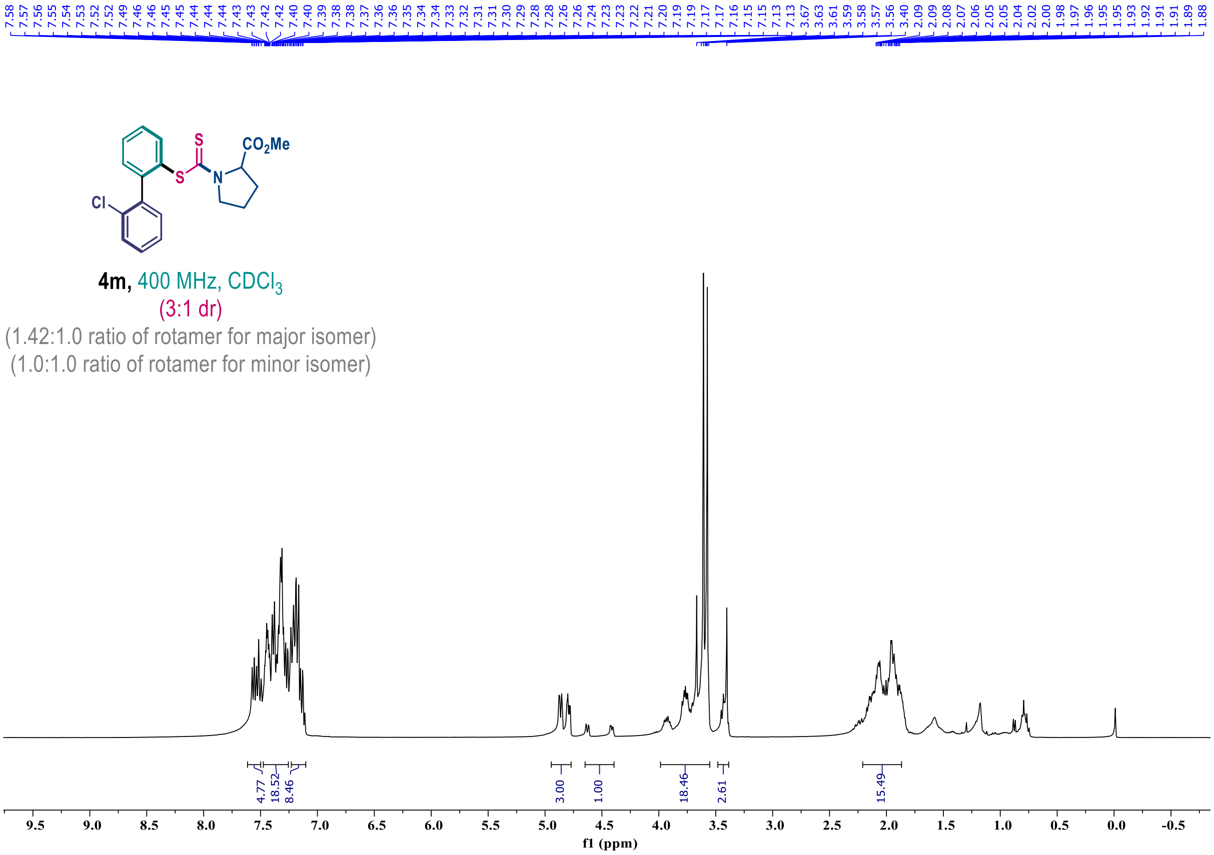
7.58 7.57 7.56 7.55 7.54 7.53 7.52 7.52 7.49 7.46 7.46 7.45 7.45 7.44 7.44 7.44 7.43 7.43 7.42 7.42 7.40 7.39 7.38 7.38 7.37 7.36 7.36 7.35 7.34 7.34 7.33 7.32 7.31 7.31 7.30 7.29 7.28 7.28 7.26 7.26 7.24 7.23 7.23 7.22 7.21 7.20 7.19 7.19 7.17 7.17 7.16 7.15 7.15 7.13 7.13 3.67 3.63 3.61 3.59 3.58 3.57 3.56 3.40 2.09 2.09 2.08 2.07 2.06 2.05 2.05 2.04 2.02 2.00 1.98 1.97 1.96 1.95 1.95 1.93 1.92 1.91 1.91 1.89 1.88
S
CO$_2$Me
S
N
Cl
4m, 400 MHz, CDCl$_3$
(3:1 dr)
(1.42:1.0 ratio of rotamer for major isomer)
(1.0:1.0 ratio of rotamer for minor isomer)
4.77 18.52 8.46 3.00 1.00 18.46 2.61 15.49
9.5 9.0 8.5 8.0 7.5 7.0 6.5 6.0 5.5 5.0 4.5 4.0 3.5 3.0 2.5 2.0 1.5 1.0 0.5 0.0 -0.5
f1 (ppm)

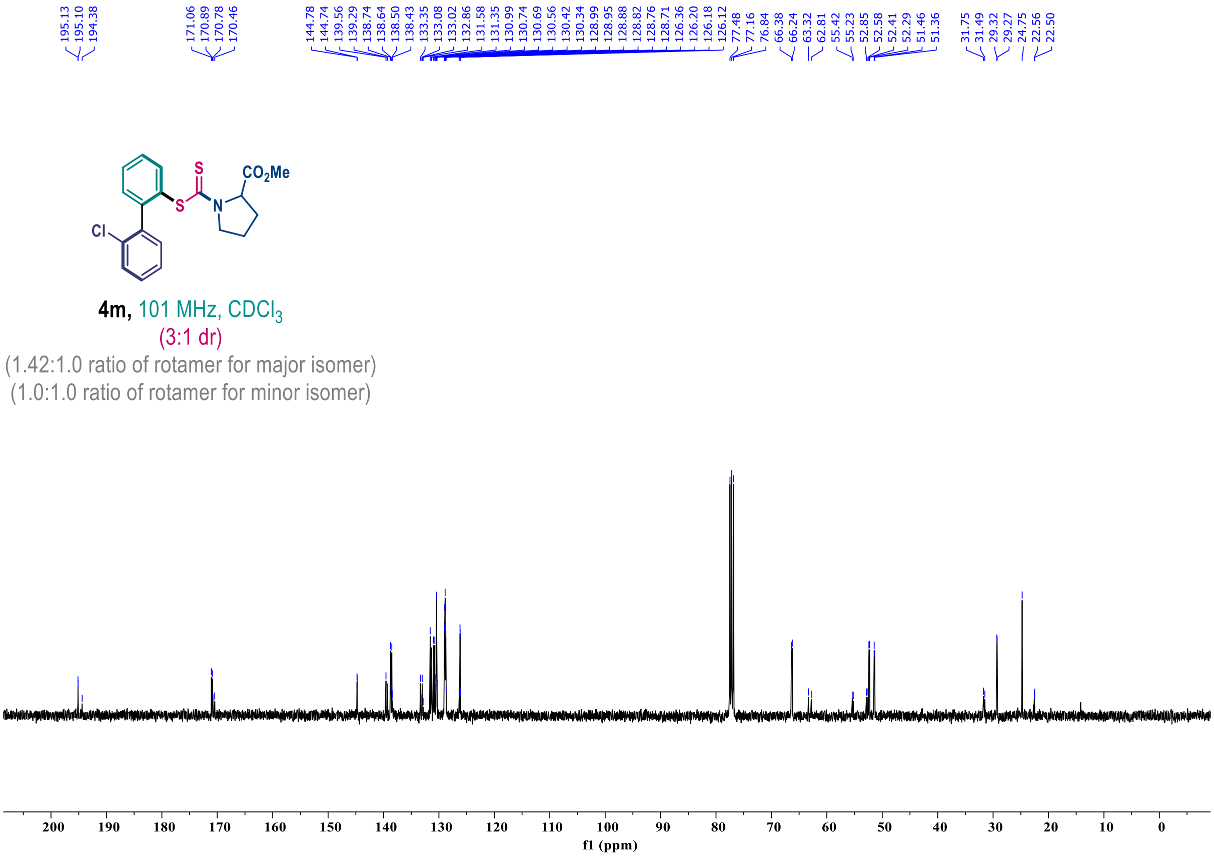
195.13 195.10 194.38 171.06 170.89 170.78 170.46 144.78 144.74 139.56 139.29 138.74 138.64 138.50 138.43 133.35 133.08 133.02 132.86 131.58 131.35 130.99 130.74 130.69 130.56 130.42 130.34 128.99 128.95 128.88 128.82 128.76 128.71 126.36 126.20 126.18 126.12 77.48 77.16 76.84 66.38 66.24 63.32 62.81 55.42 55.23 52.85 52.58 52.41 52.29 51.46 51.36 31.75 31.49 29.32 29.27 24.75 22.56 22.50
S
CO$_2$Me
S
N
Cl
4m, 101 MHz, CDCl$_3$
(3:1 dr)
(1.42:1.0 ratio of rotamer for major isomer)
(1.0:1.0 ratio of rotamer for minor isomer)
200 190 180 170 160 150 140 130 120 110 100 90 80 70 60 50 40 30 20 10 0
f1 (ppm)

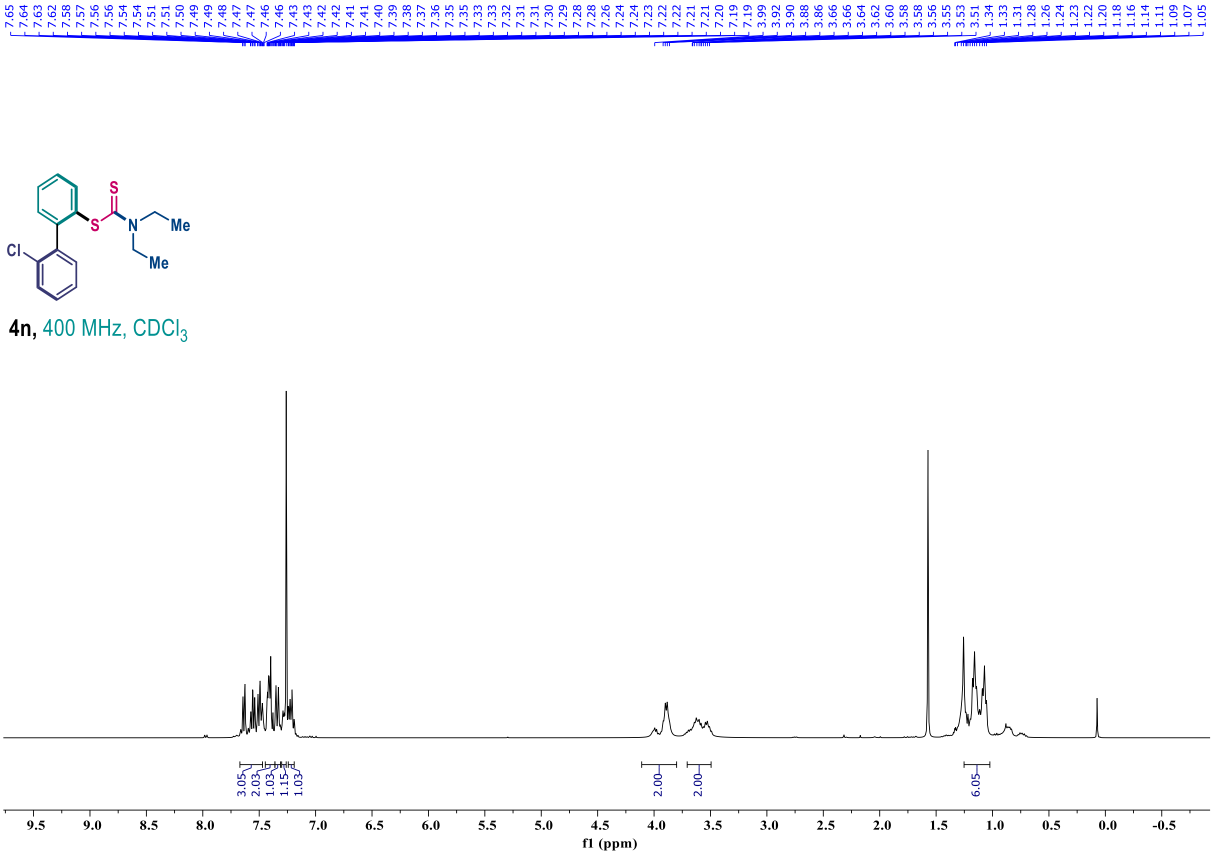

4n, 400 MHz, $CDCl_3$

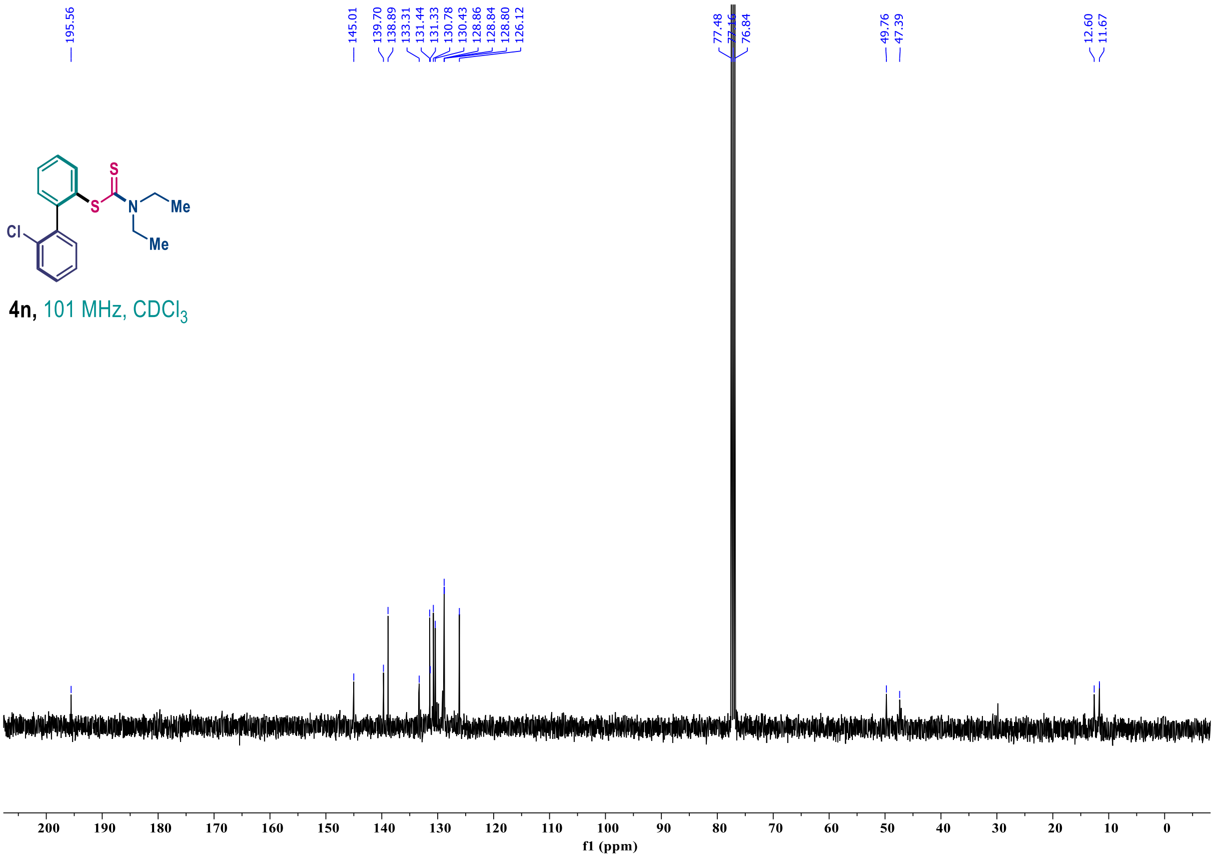

4n, 101 MHz, $CDCl_3$

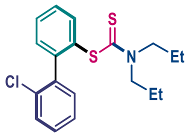

4o, 400 MHz, $CDCl_3$

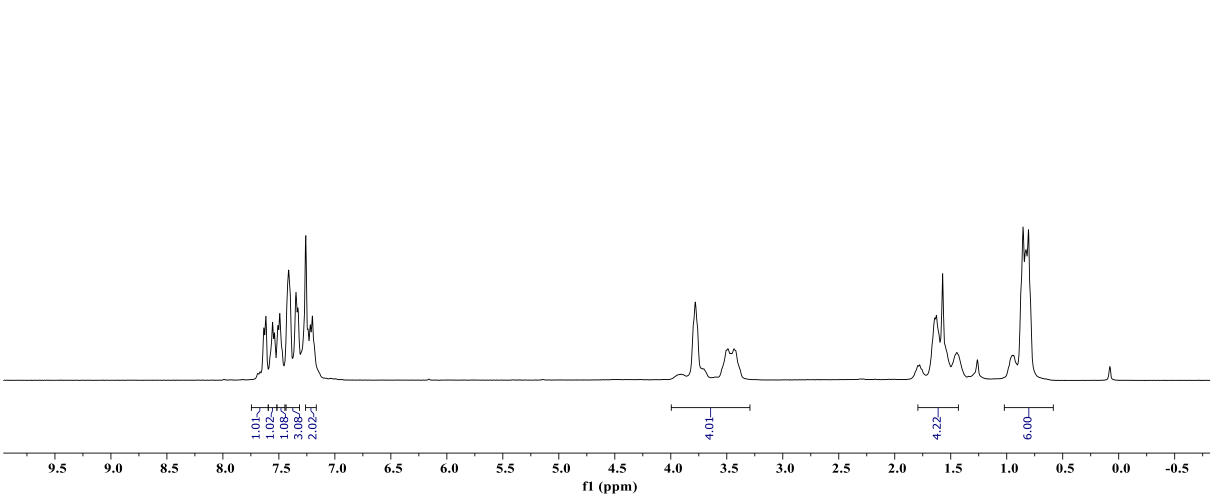

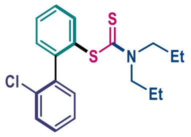

4o, 101 MHz, $CDCl_3$

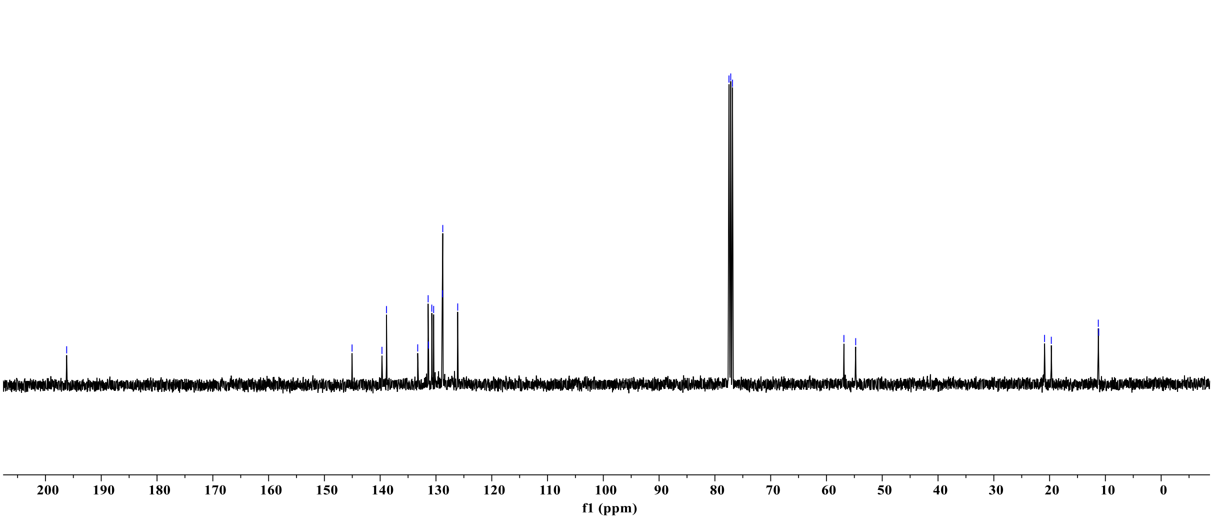

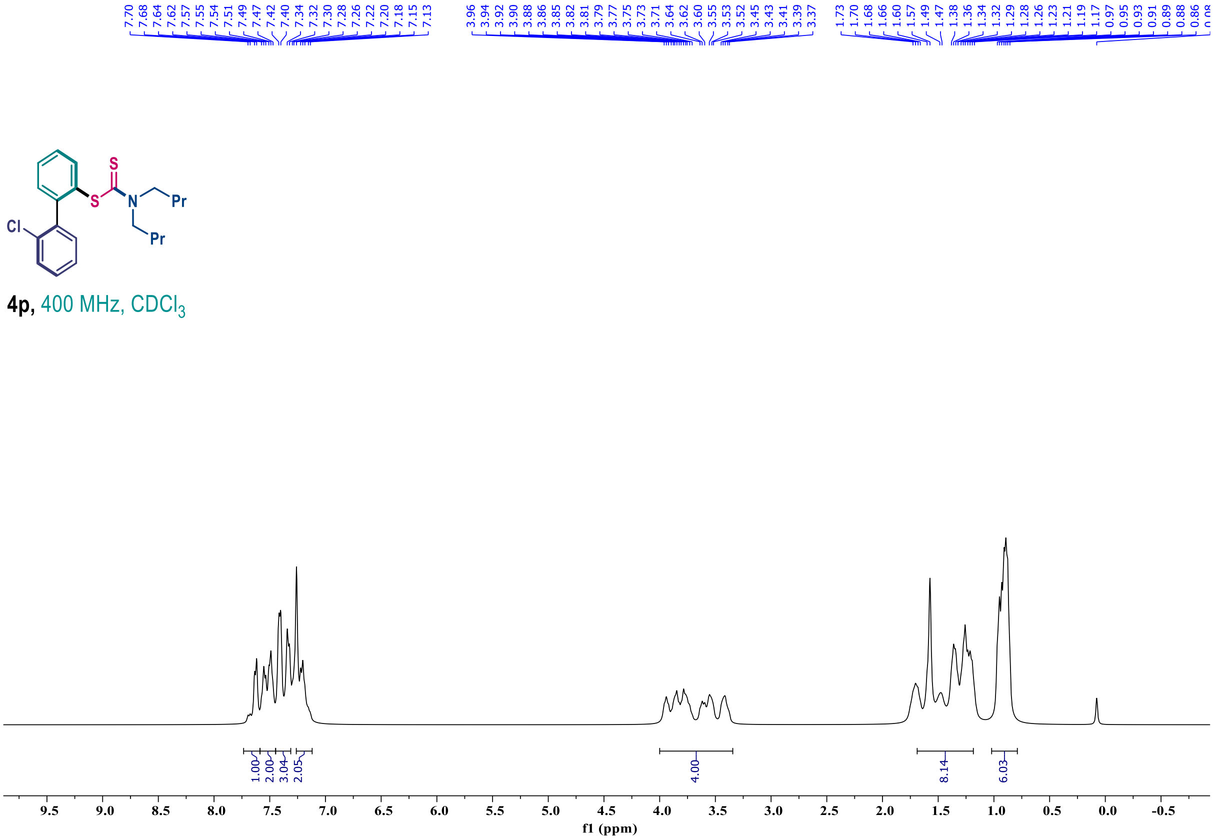

4p, 400 MHz, $CDCl_3$

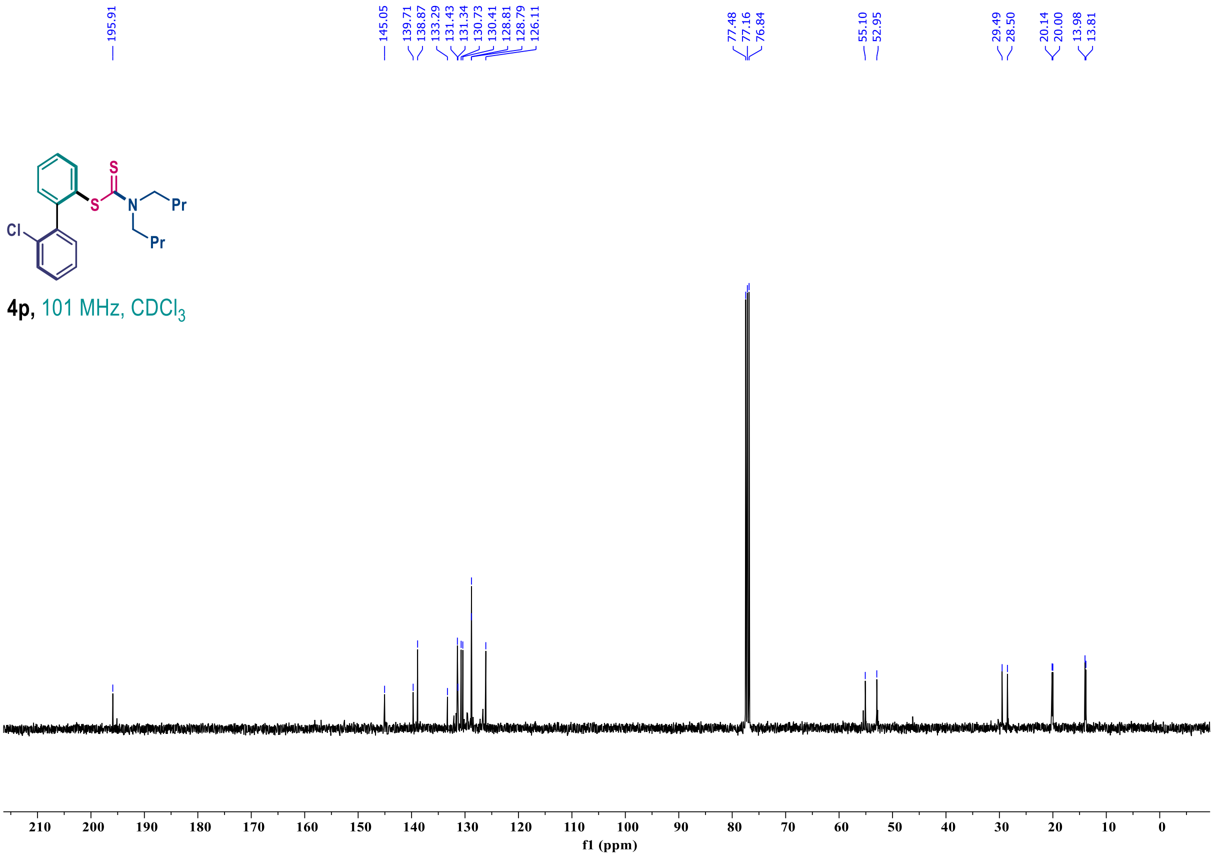

4p, 101 MHz, $CDCl_3$

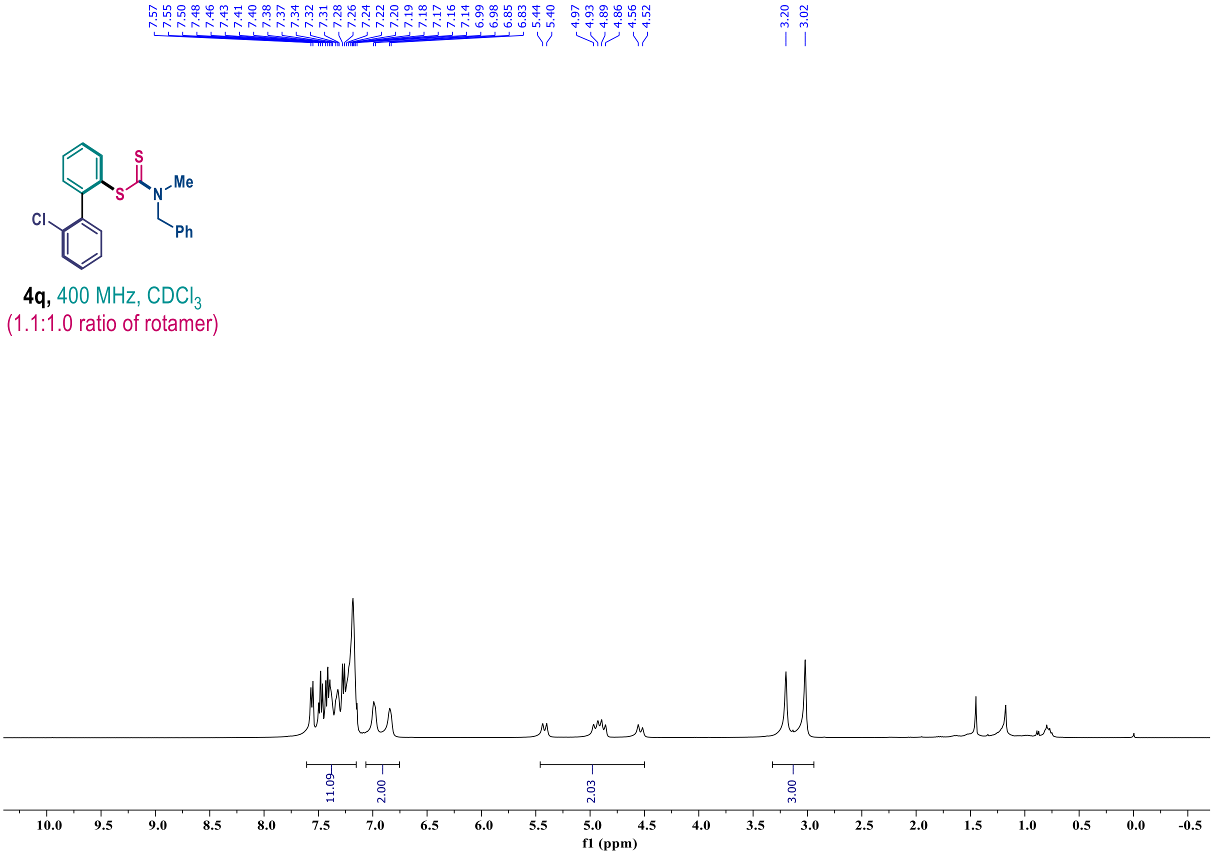
4q, 400 MHz, CDCl3
(1.1:1.0 ratio of rotamer)
f1 (ppm)

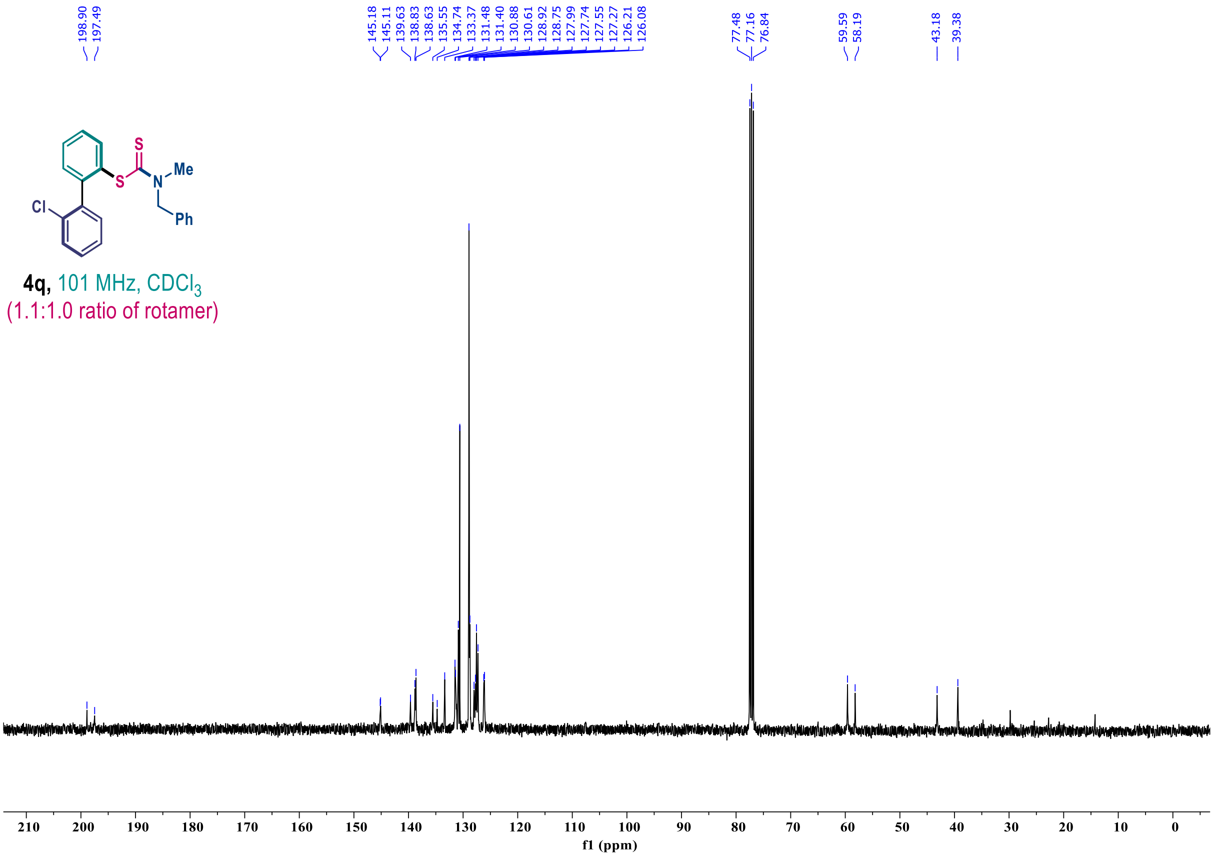
4q, 101 MHz, CDCl3
(1.1:1.0 ratio of rotamer)
f1 (ppm)

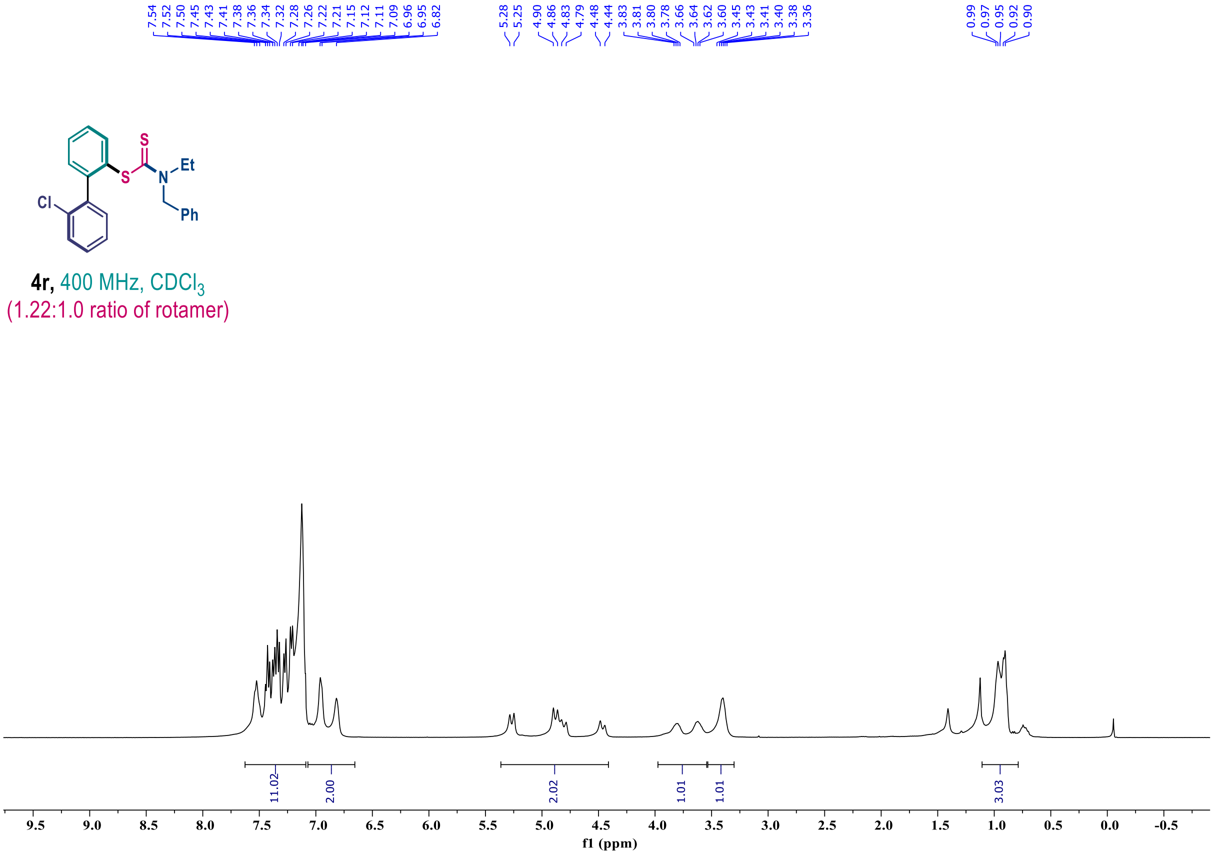

4r, 400 MHz, $CDCl_3$
(1.22:1.0 ratio of rotamer)

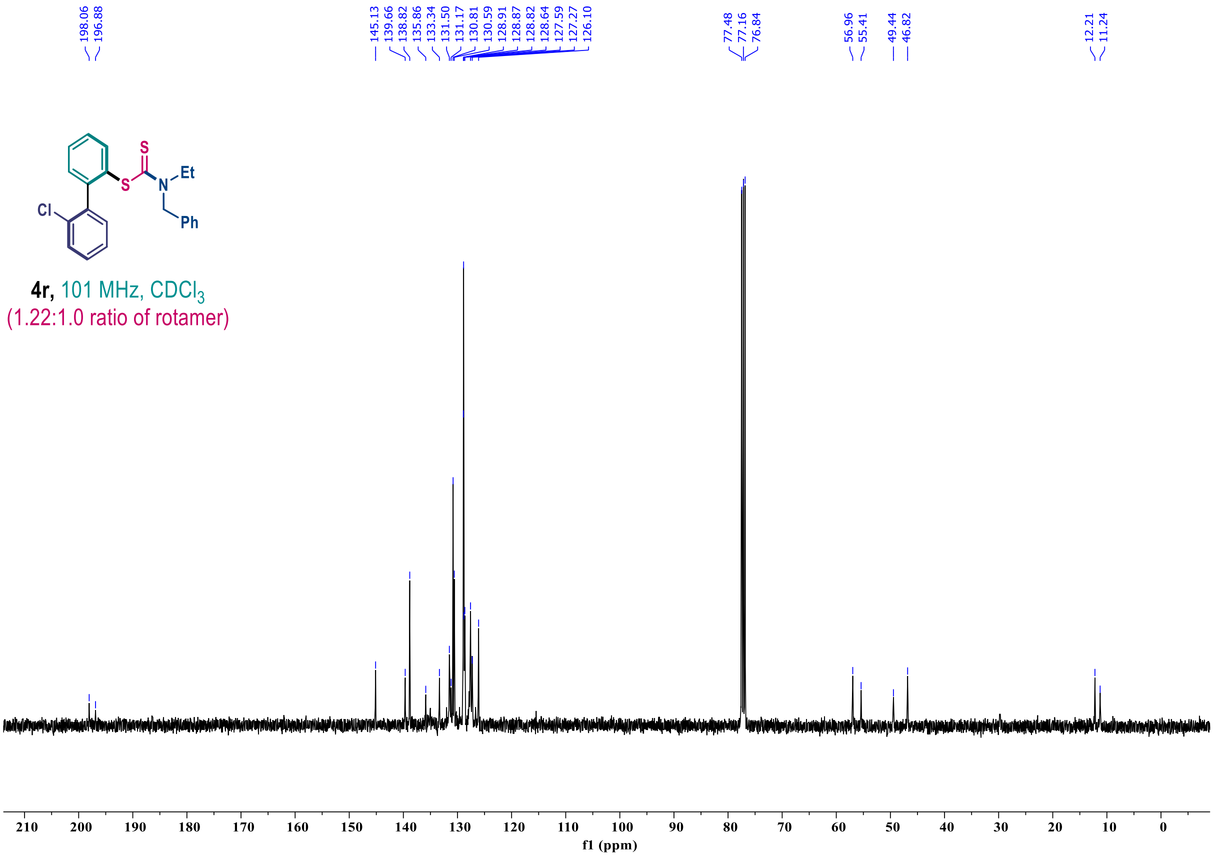

4r, 101 MHz, $CDCl_3$
(1.22:1.0 ratio of rotamer)

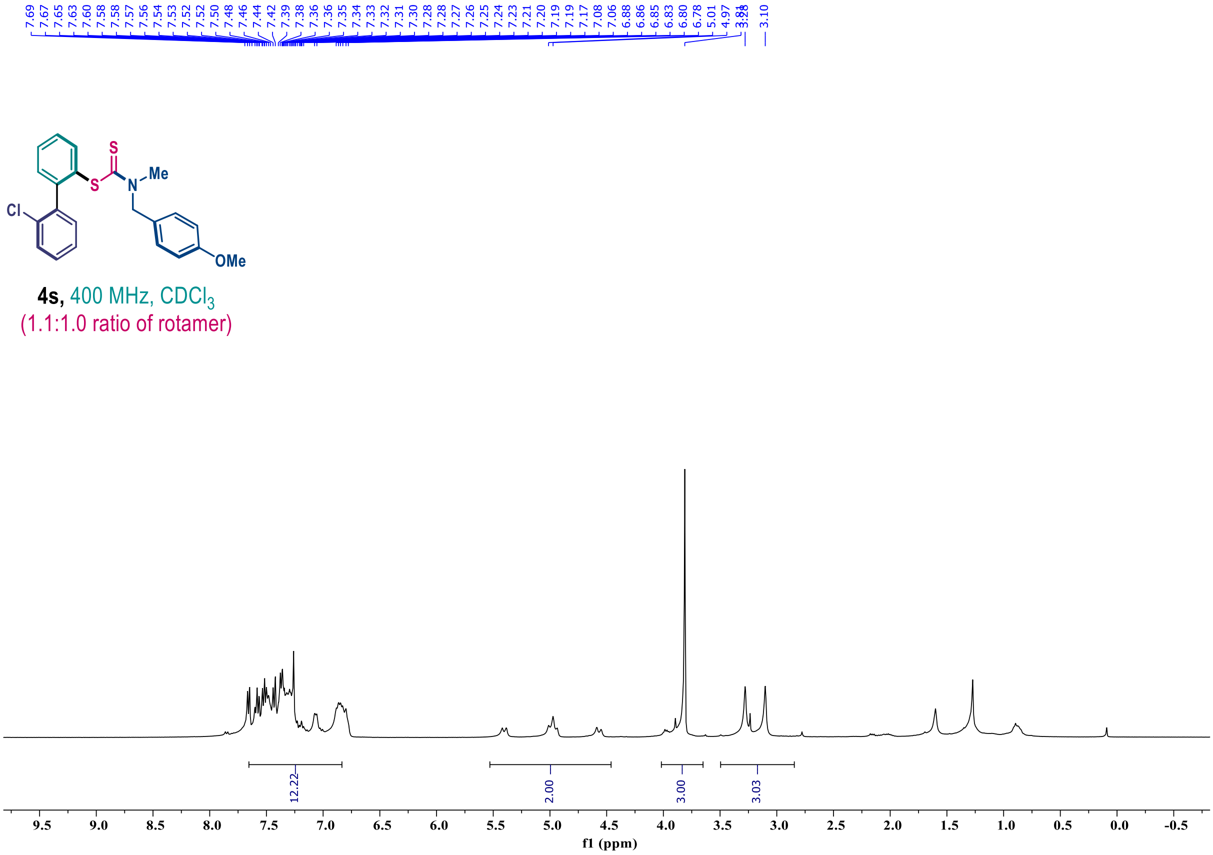
7.69 7.67 7.65 7.63 7.60 7.58 7.58 7.57 7.56 7.54 7.53 7.52 7.52 7.50 7.48 7.46 7.44 7.42 7.39 7.38 7.36 7.36 7.35 7.34 7.33 7.32 7.31 7.30 7.28 7.28 7.27 7.26 7.25 7.24 7.23 7.21 7.20 7.19 7.19 7.17 7.08 7.06 6.88 6.86 6.85 6.83 6.80 6.78 5.01 4.97 3.81 3.28 3.10
S
N
Me
S
Cl
OMe
4s, 400 MHz, CDCl3
(1.1:1.0 ratio of rotamer)
12.22
2.00
3.00
3.03
f1 (ppm)

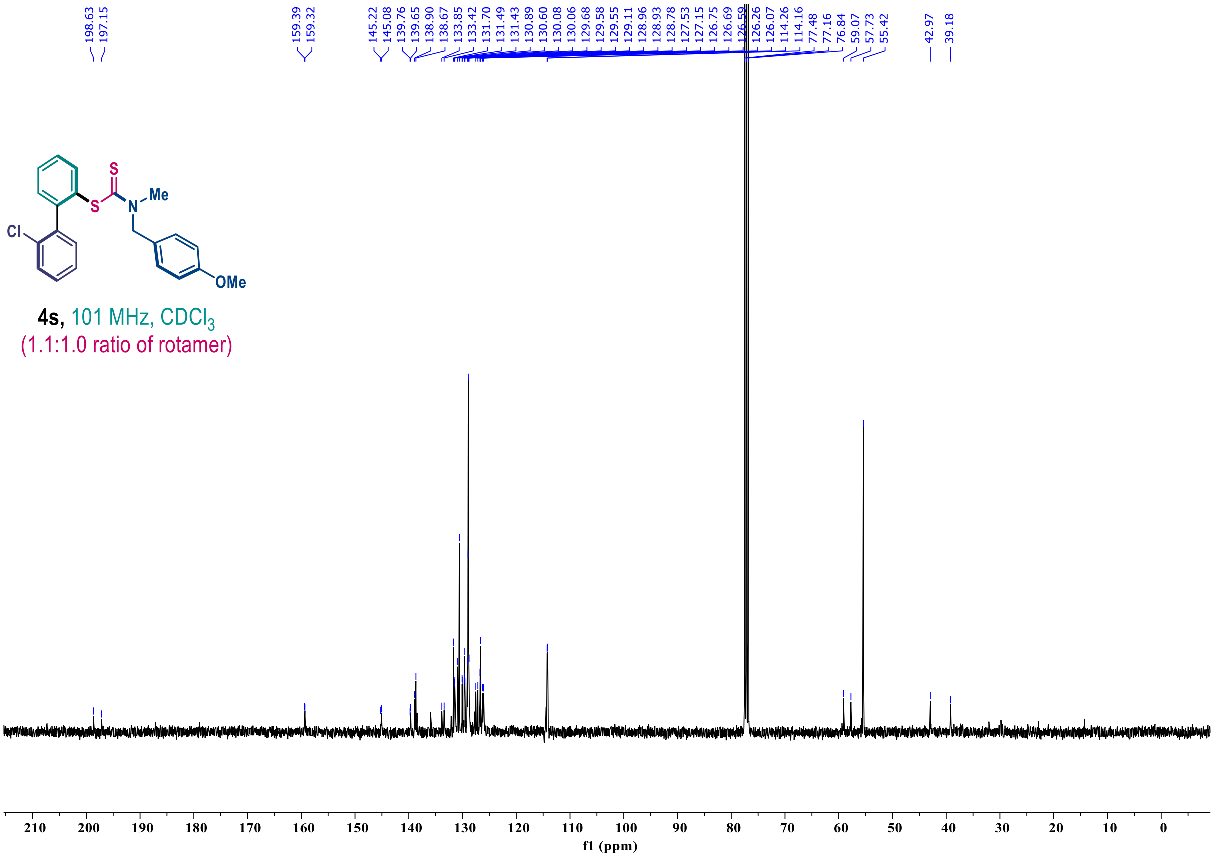
198.63 197.15 159.39 159.32 145.22 145.08 139.76 139.65 138.90 138.67 133.85 133.42 131.70 131.49 131.43 130.89 130.60 130.08 130.06 129.68 129.58 129.55 129.11 128.96 128.93 128.78 127.53 127.15 126.75 126.69 126.59 126.26 126.07 114.26 114.16 77.48 77.16 76.84 59.07 57.73 55.42 42.97 39.18
S
N
Me
S
Cl
OMe
4s, 101 MHz, CDCl3
(1.1:1.0 ratio of rotamer)
f1 (ppm)

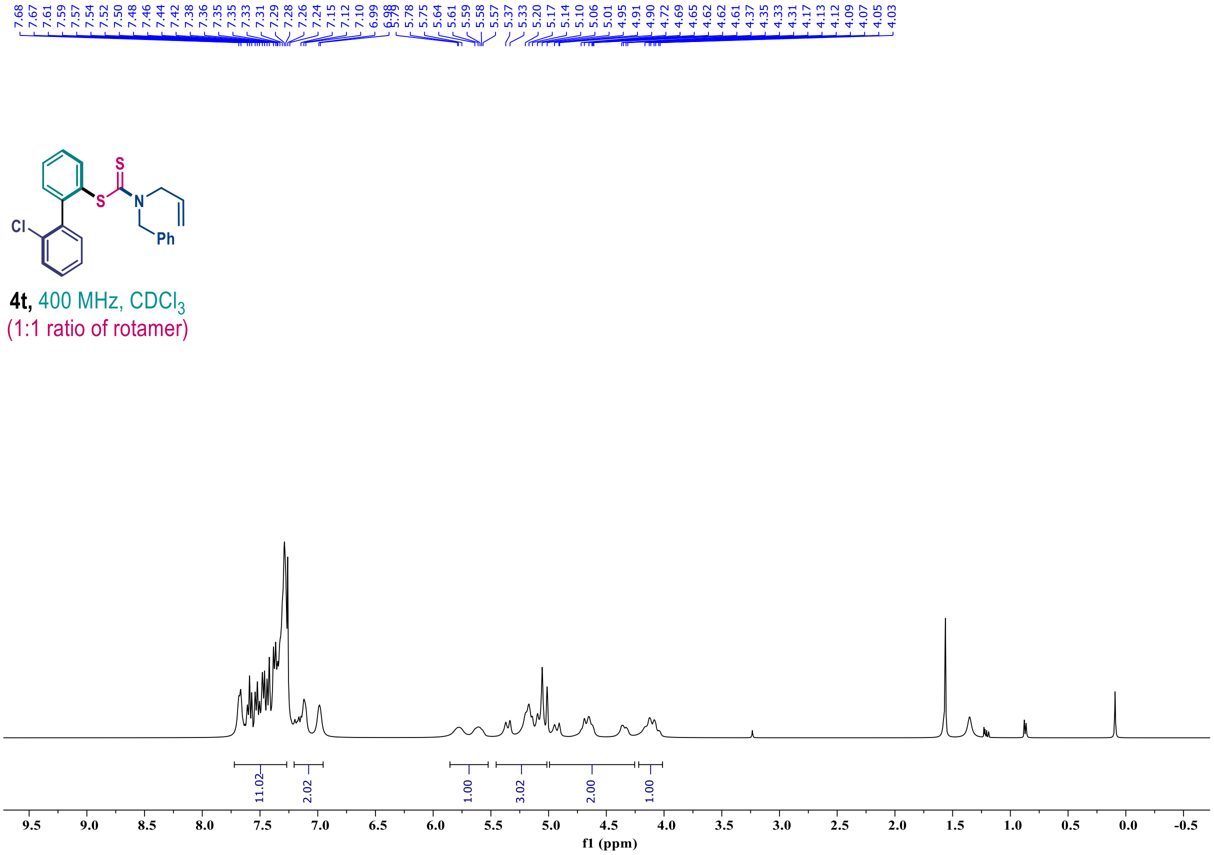
S
S
N
Cl
Ph
4t, 400 MHz, CDCl3
(1:1 ratio of rotamer)
11.02
2.02
1.00
3.02
2.00
1.00
f1 (ppm)

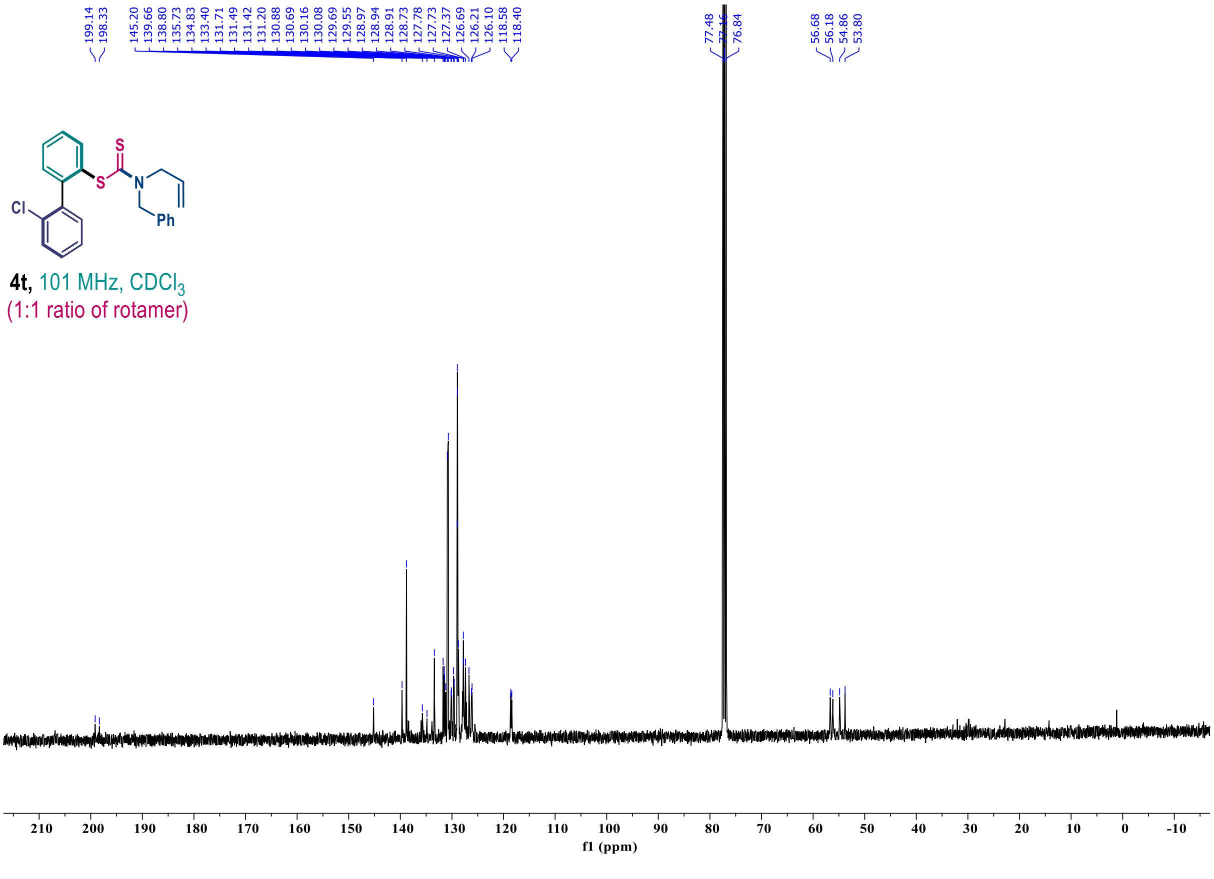
S
S
N
Cl
Ph
4t, 101 MHz, CDCl3
(1:1 ratio of rotamer)
f1 (ppm)

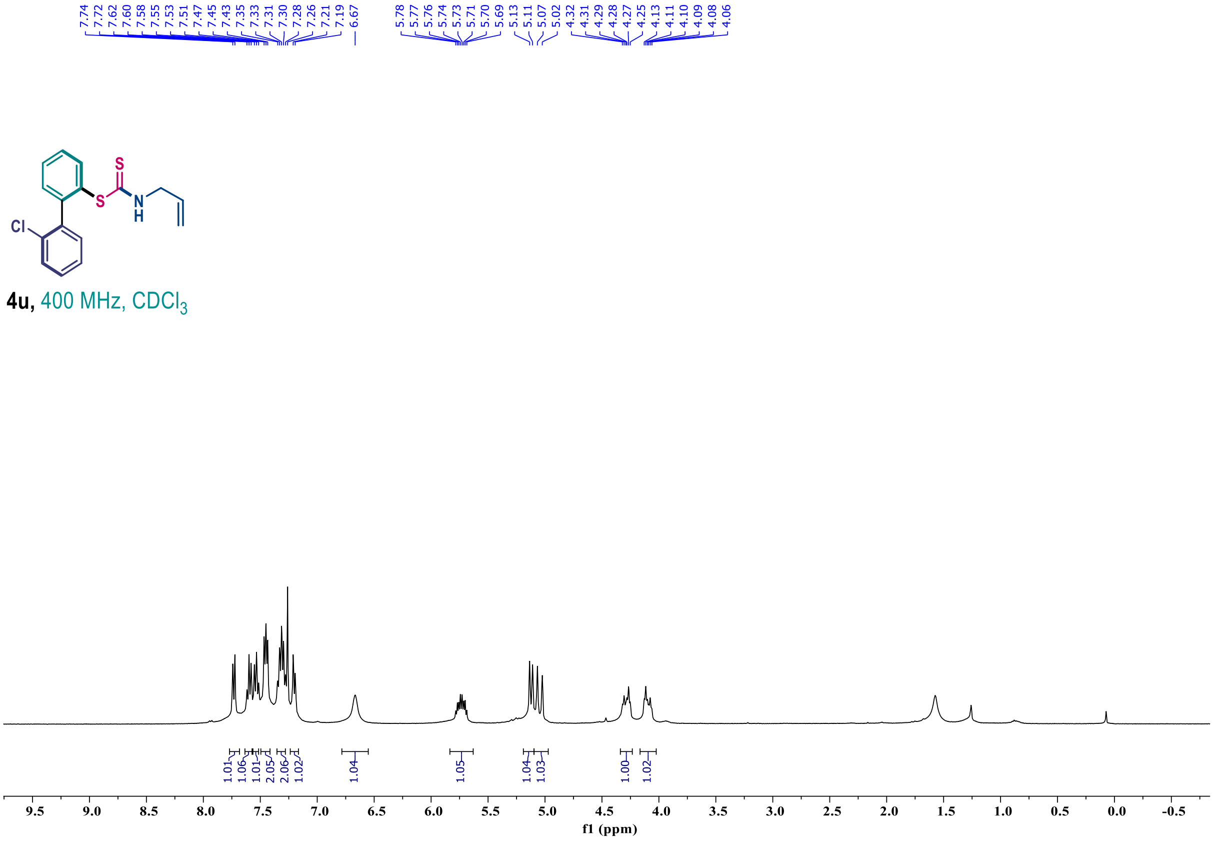
7.74
7.72
7.62
7.60
7.58
7.55
7.53
7.51
7.47
7.45
7.43
7.35
7.33
7.31
7.30
7.28
7.26
7.21
7.19
6.67
5.78
5.77
5.76
5.74
5.73
5.71
5.70
5.69
5.13
5.11
5.07
5.02
4.32
4.31
4.29
4.28
4.27
4.25
4.13
4.11
4.10
4.09
4.08
4.06
S
S
N
H
Cl
4u, 400 MHz, CDCl3
1.01
1.06
1.01
2.05
2.06
1.02
1.04
1.05
1.04
1.03
1.00
1.02
9.5
9.0
8.5
8.0
7.5
7.0
6.5
6.0
5.5
5.0
4.5
4.0
3.5
3.0
2.5
2.0
1.5
1.0
0.5
0.0
-0.5
f1 (ppm)

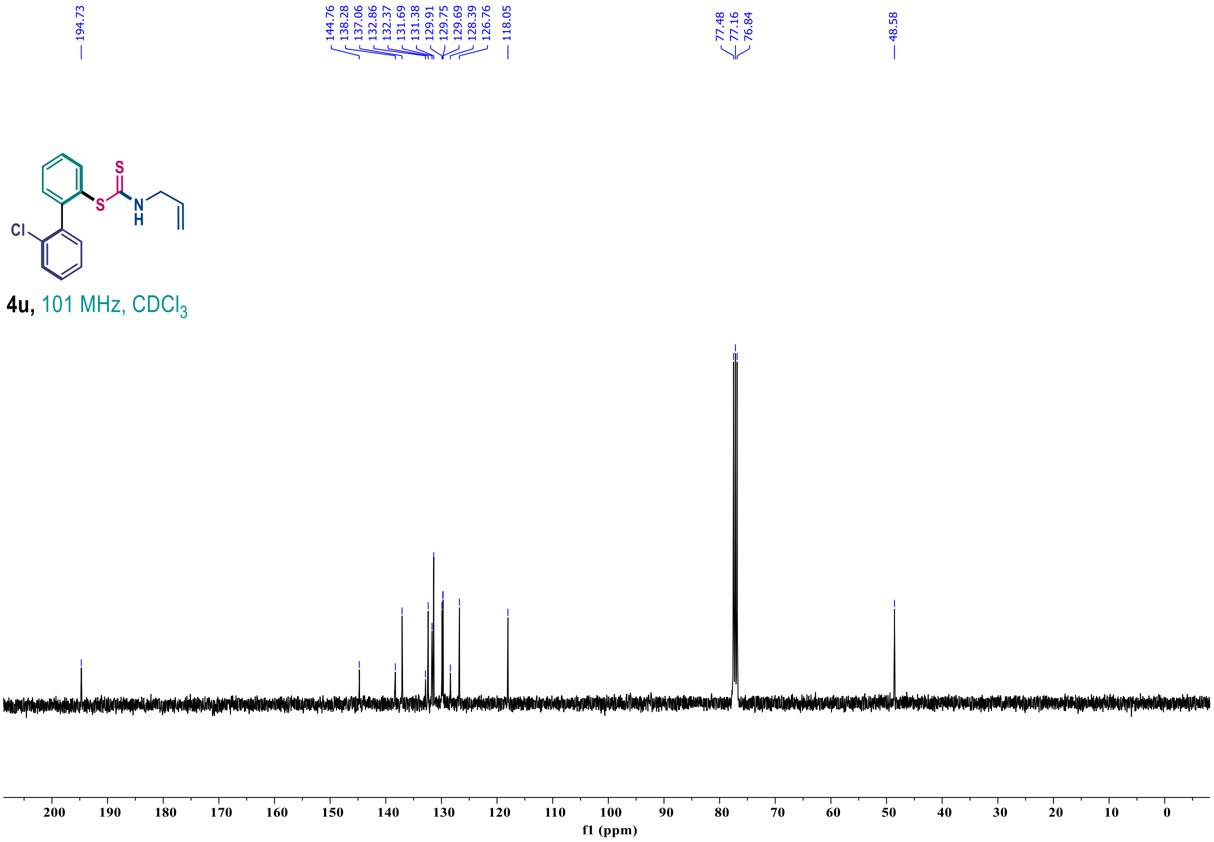
194.73
144.76
138.28
137.06
132.86
132.37
131.69
131.38
129.91
129.75
129.69
128.39
126.76
118.05
77.48
77.16
76.84
48.58
S
S
N
H
Cl
4u, 101 MHz, CDCl3
200
190
180
170
160
150
140
130
120
110
100
90
80
70
60
50
40
30
20
10
0
f1 (ppm)

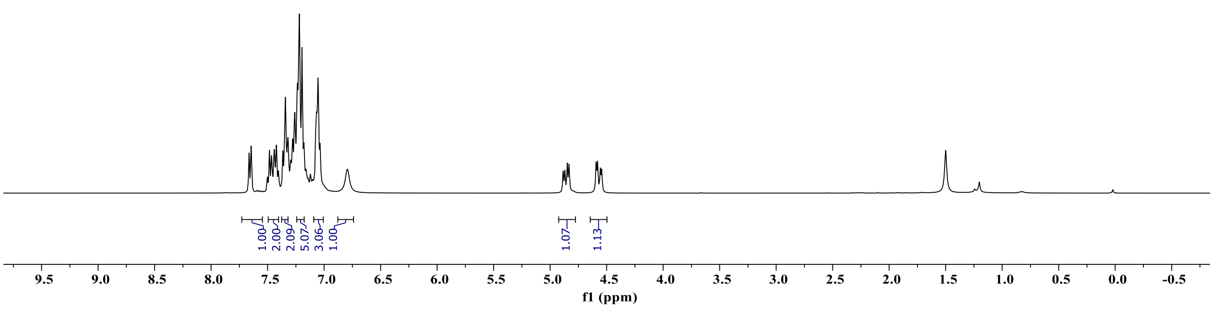

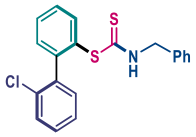

4v, 400 MHz, $CDCl_3$
(>10:1 ratio of rotamer)

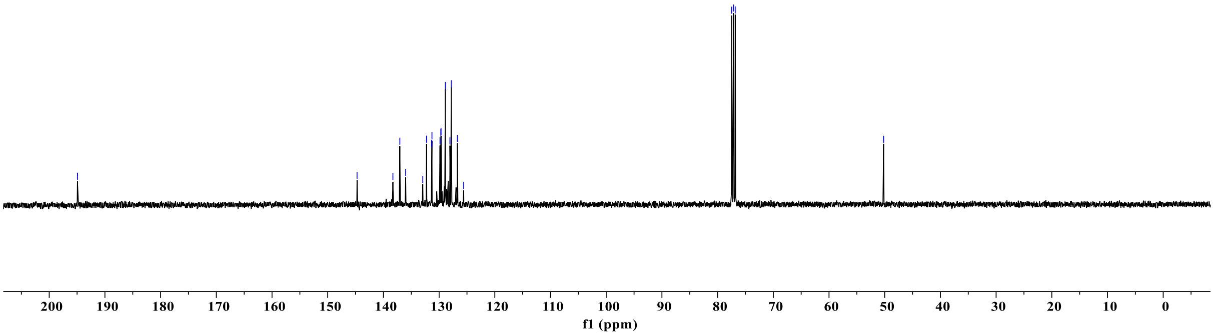

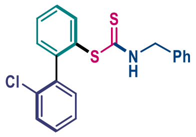

4v, 101 MHz, $CDCl_3$
(>10:1 ratio of rotamer)

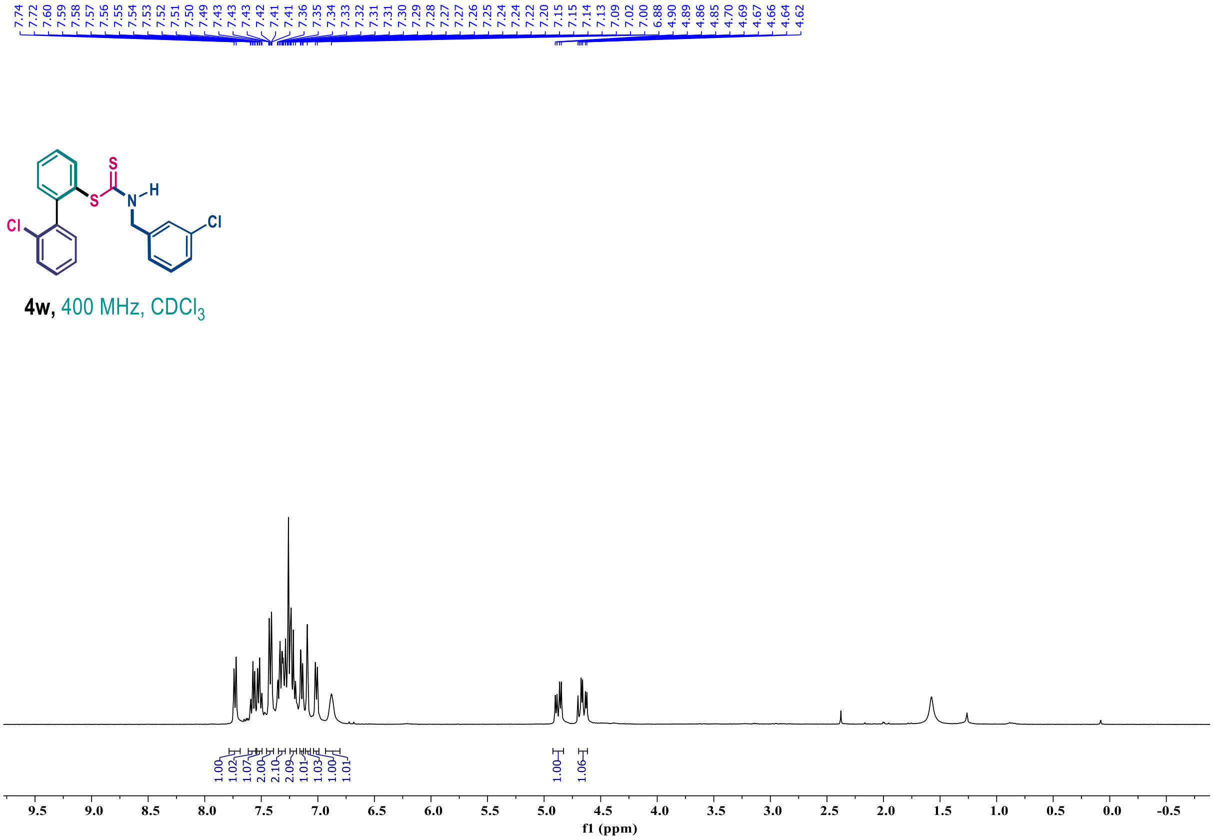

4w, 400 MHz, $CDCl_3$

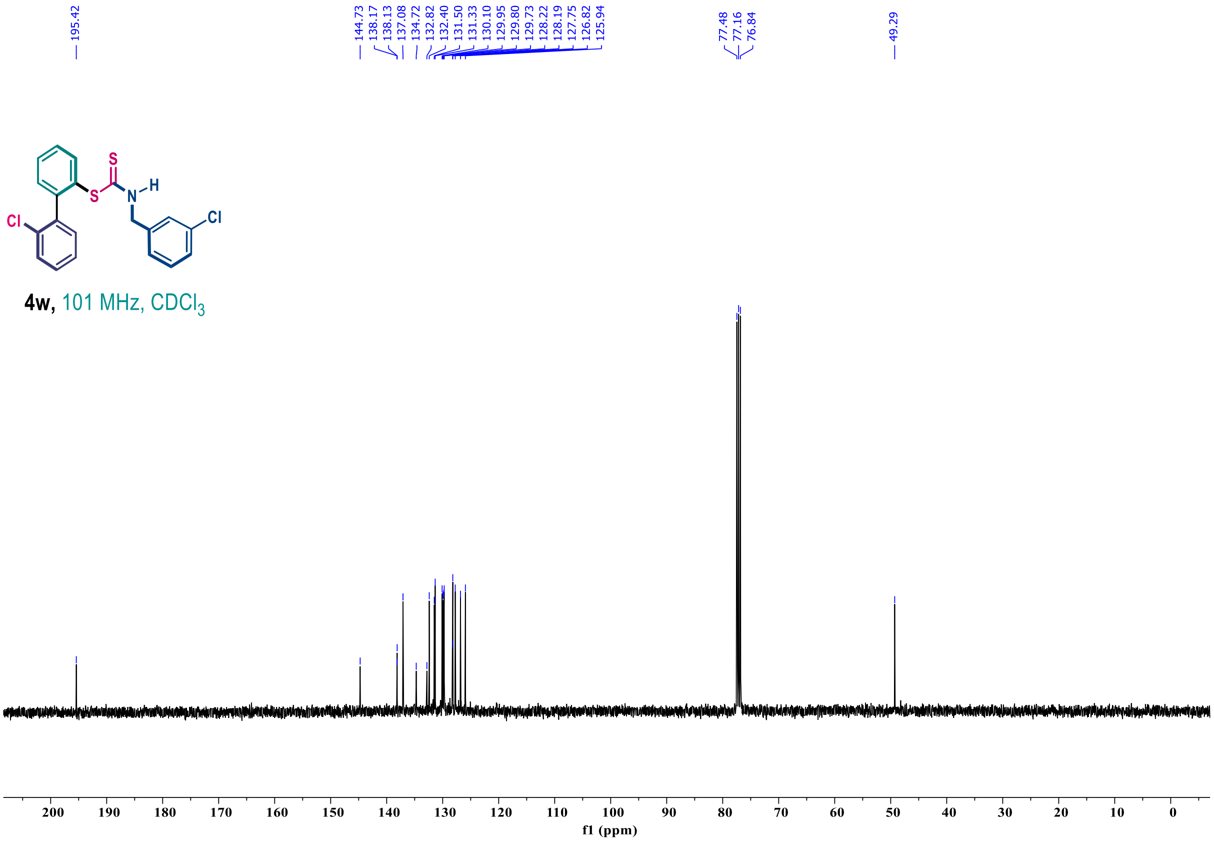

4w, 101 MHz, $CDCl_3$

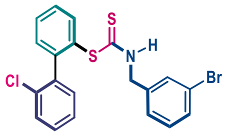

4x, 400 MHz, CDCl$_3$

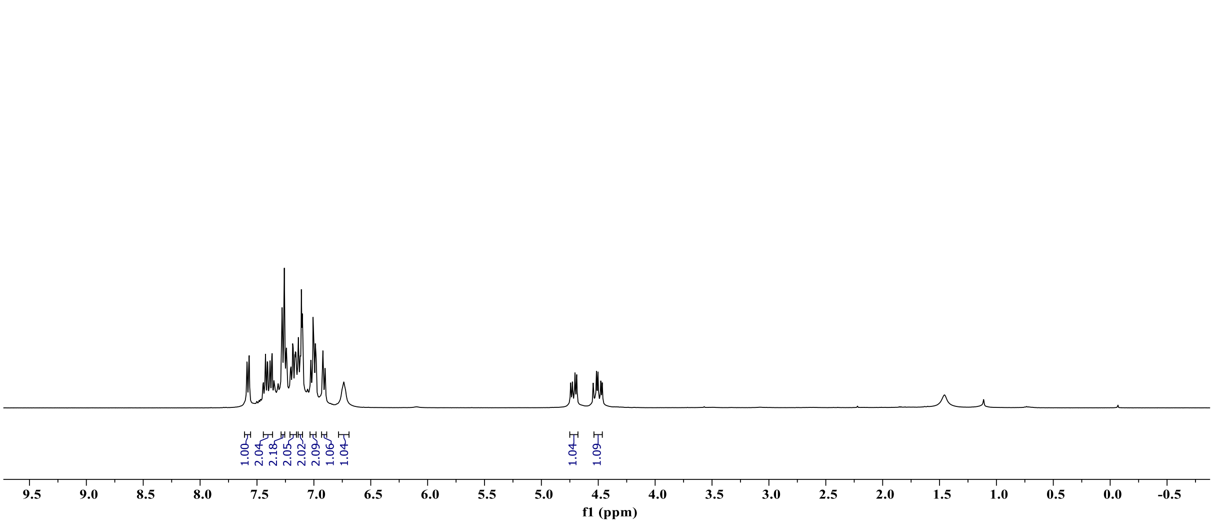

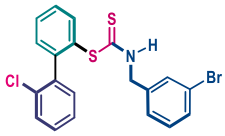

4x, 101 MHz, CDCl$_3$

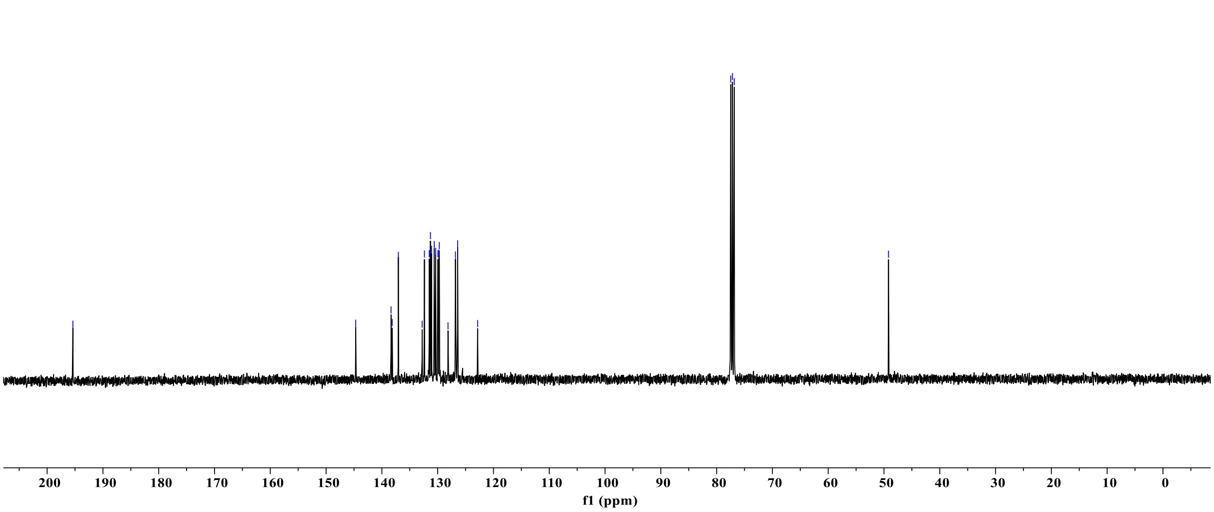

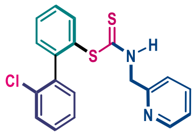

4y, 400 MHz, $CDCl_3$

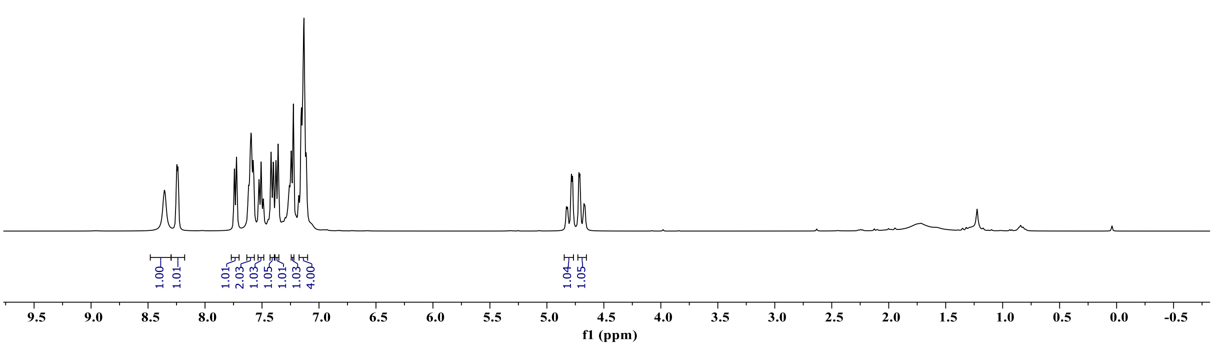

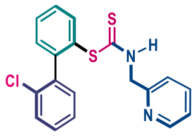

4y, 101 MHz, $CDCl_3$

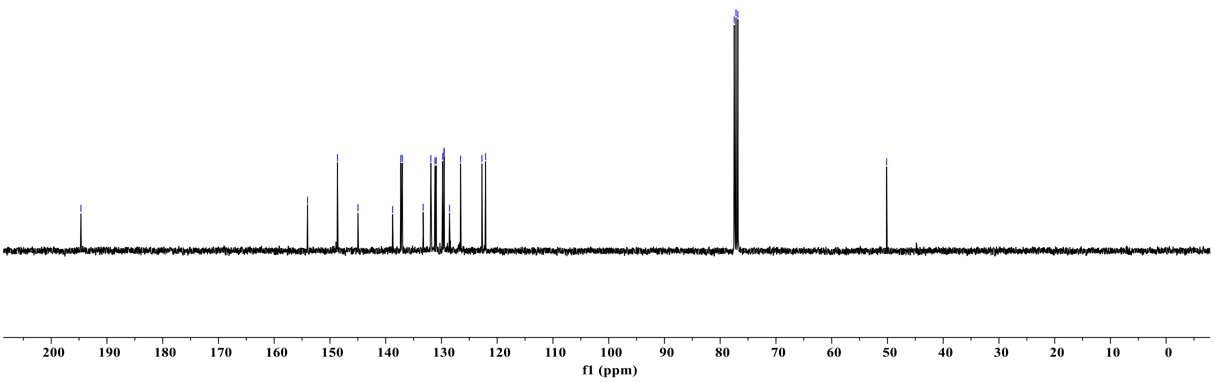

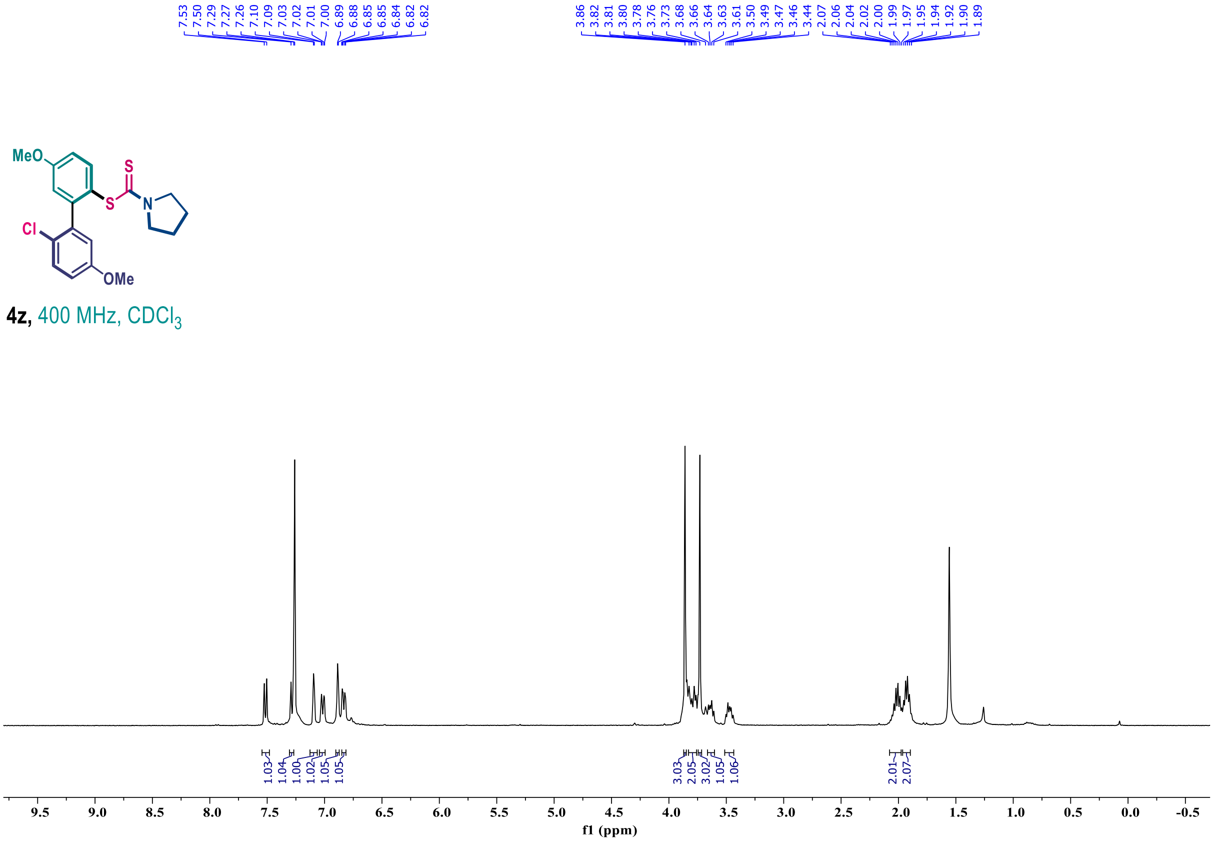
7.53 7.50 7.29 7.27 7.26 7.10 7.09 7.03 7.02 7.01 7.00 6.89 6.88 6.85 6.85 6.84 6.82 6.82
3.86 3.82 3.81 3.80 3.78 3.76 3.73 3.68 3.66 3.64 3.63 3.61 3.50 3.49 3.47 3.46 3.44 2.07 2.06 2.04 2.02 2.00 1.99 1.97 1.95 1.94 1.92 1.90 1.89
MeO
S
S
N
Cl
OMe
4z, 400 MHz, CDCl$_3$
1.03 1.04 1.00 1.02 1.05 1.05
3.03 2.05 3.02 1.05 1.06
2.01 2.07
f1 (ppm)

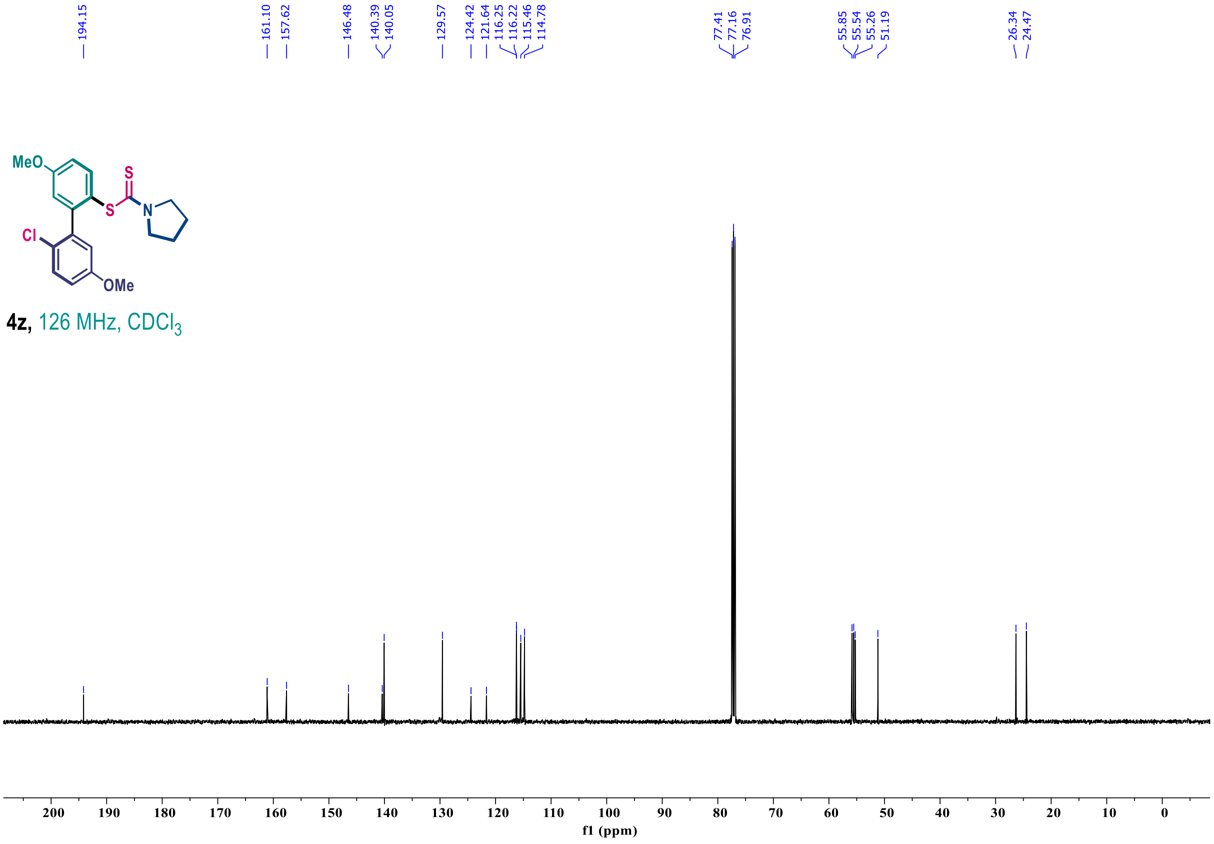
194.15
161.10 157.62
146.48
140.39 140.05
129.57
124.42 121.64 116.25 116.22 115.46 114.78
77.41 77.16 76.91
55.85 55.54 55.26 51.19
26.34 24.47
MeO
S
S
N
Cl
OMe
4z, 126 MHz, CDCl$_3$
f1 (ppm)

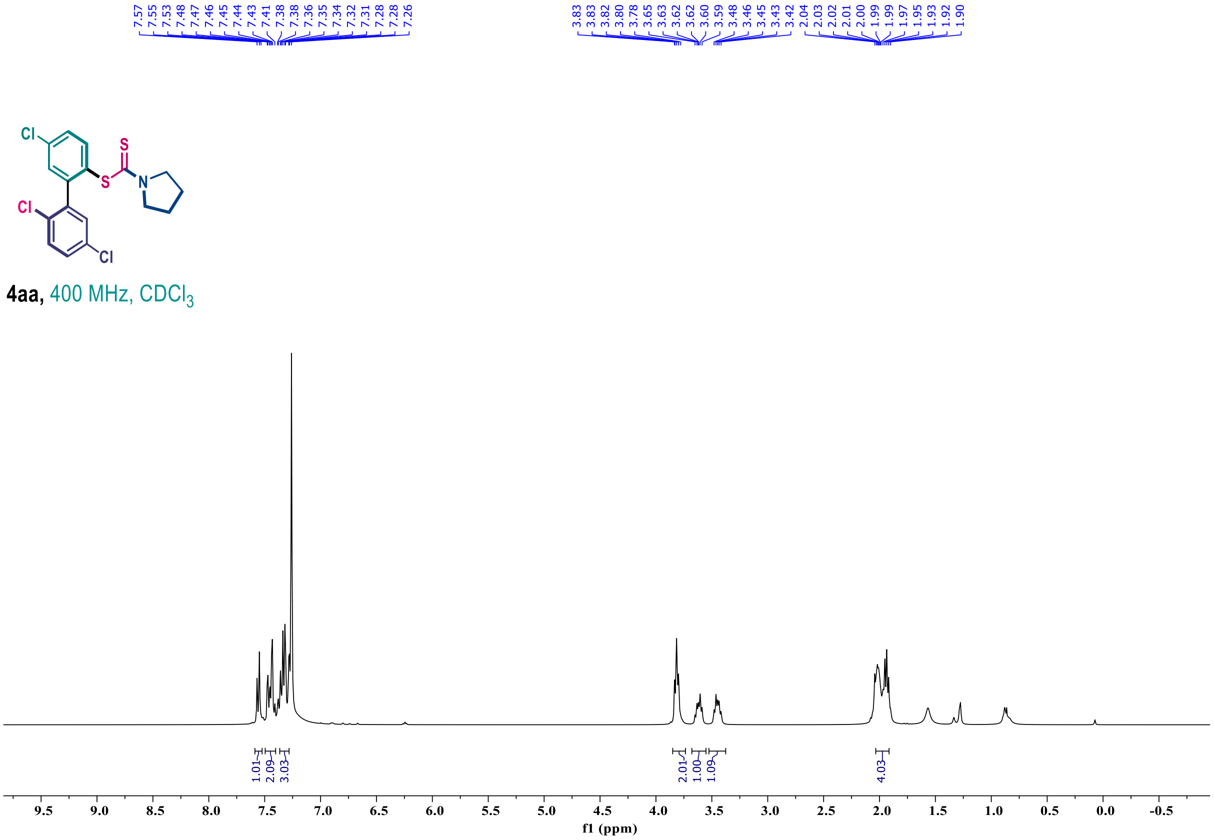
4aa, 400 MHz, CDCl3
f1 (ppm)

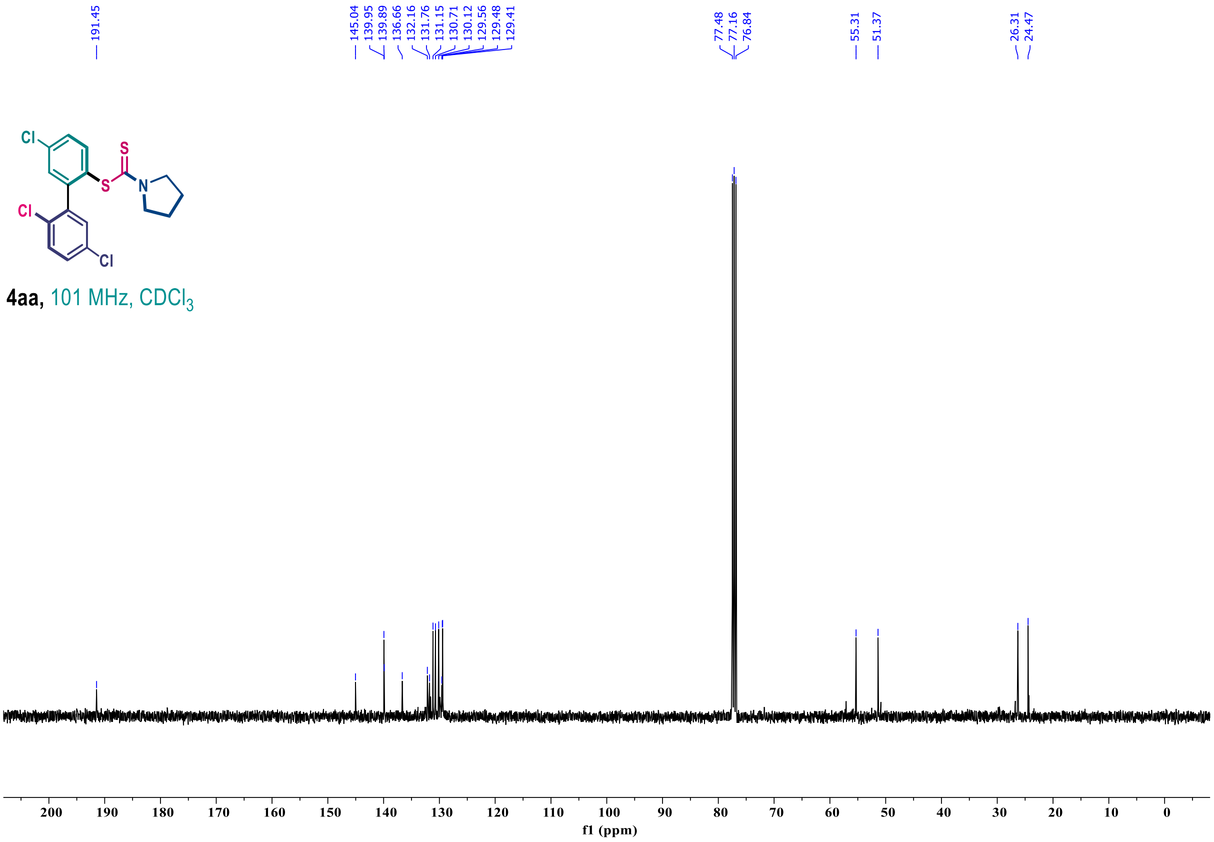
4aa, 101 MHz, CDCl3
f1 (ppm)

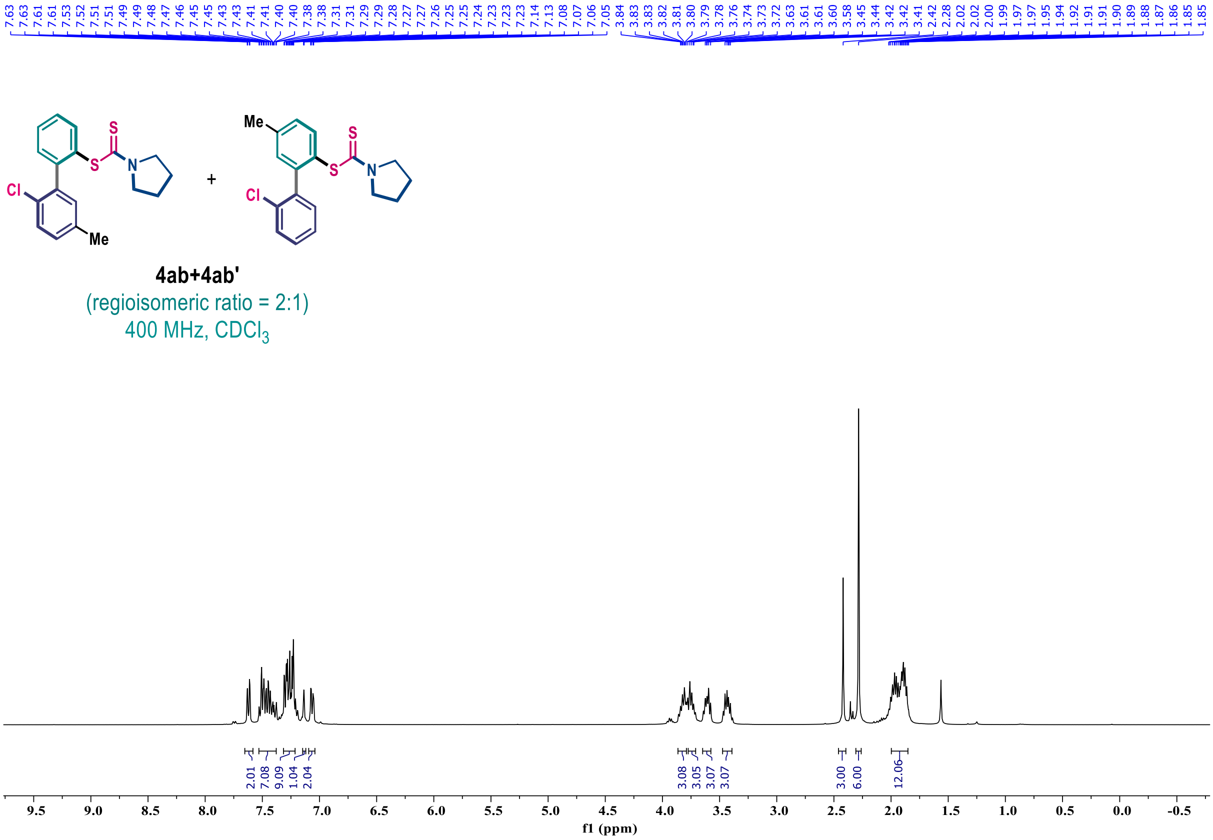
7.63 7.63 7.61 7.61 7.53 7.52 7.51 7.51 7.49 7.49 7.48 7.47 7.46 7.45 7.45 7.43 7.43 7.41 7.41 7.40 7.40 7.38 7.38 7.31 7.31 7.29 7.29 7.28 7.27 7.27 7.26 7.25 7.25 7.24 7.23 7.23 7.23 7.14 7.13 7.08 7.07 7.06 7.05 3.84 3.83 3.83 3.82 3.81 3.80 3.79 3.78 3.76 3.74 3.73 3.72 3.63 3.61 3.61 3.60 3.58 3.45 3.44 3.42 3.42 3.41 2.42 2.28 2.02 2.02 2.00 1.99 1.97 1.97 1.95 1.94 1.92 1.91 1.91 1.90 1.89 1.88 1.87 1.86 1.85 1.85
Me
S
Cl
N
+
4ab+4ab'
(regioisomeric ratio = 2:1)
400 MHz, CDCl3
2.01 7.08 9.09 1.04 2.04
3.08 3.05 3.07 3.07
3.00 6.00
12.06
9.5 9.0 8.5 8.0 7.5 7.0 6.5 6.0 5.5 5.0 4.5 4.0 3.5 3.0 2.5 2.0 1.5 1.0 0.5 0.0 -0.5
f1 (ppm)

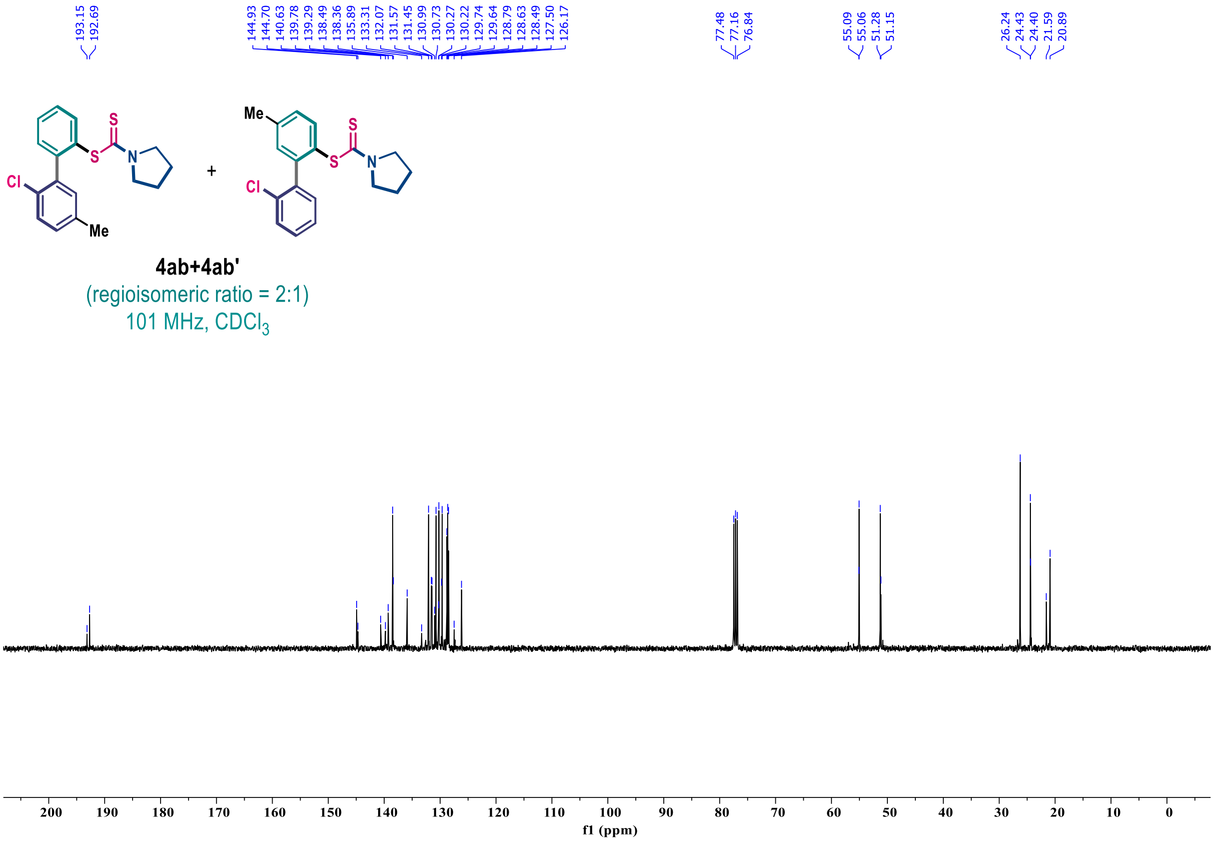
193.15 192.69
144.93 144.70 140.63 139.78 139.29 138.49 138.36 135.89 133.31 132.07 131.57 131.45 130.99 130.73 130.27 130.22 129.74 129.64 128.79 128.63 128.49 127.50 126.17
77.48 77.16 76.84
55.09 55.06 51.28 51.15
26.24 24.43 24.40 21.59 20.89
Me
S
Cl
N
+
4ab+4ab'
(regioisomeric ratio = 2:1)
101 MHz, CDCl3
200 190 180 170 160 150 140 130 120 110 100 90 80 70 60 50 40 30 20 10 0
f1 (ppm)

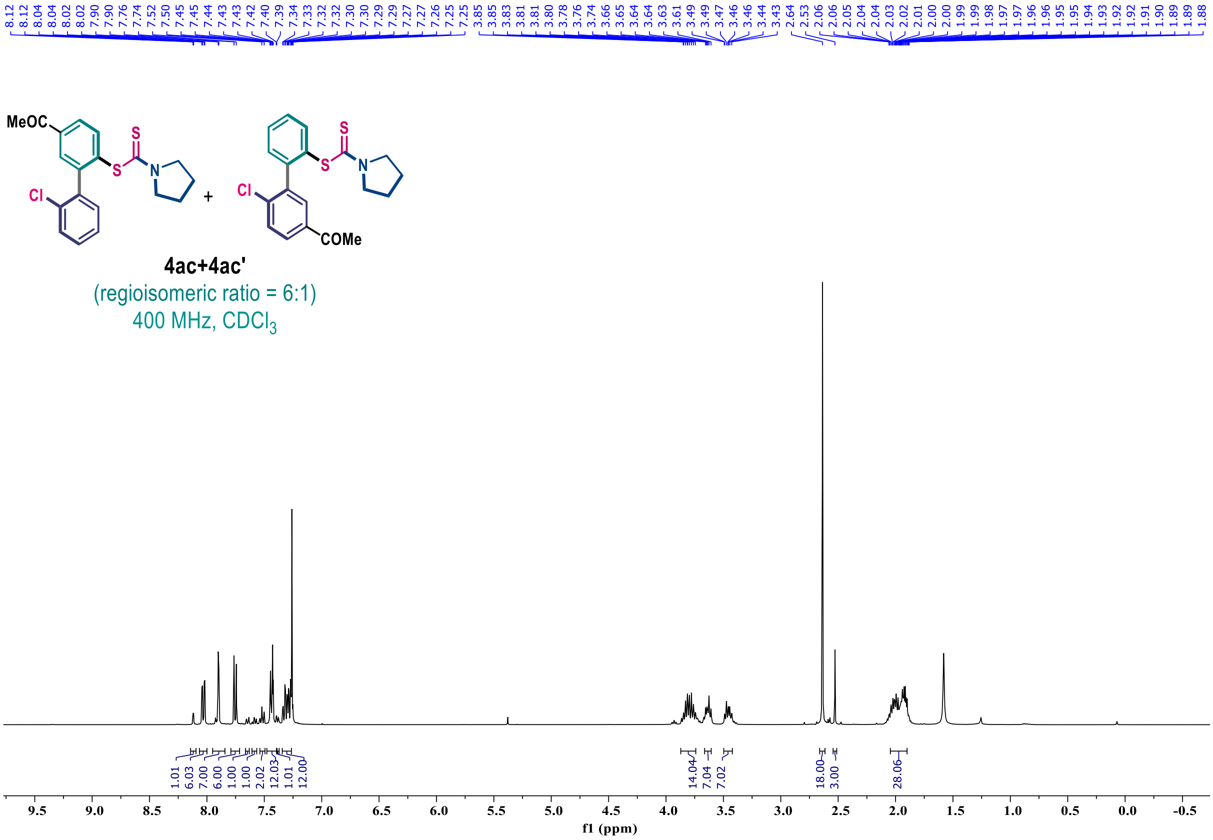

MeOC
Cl
S
S
N
+
COMe
4ac+4ac'
(regioisomeric ratio = 6:1)
400 MHz, CDCl3
f1 (ppm)

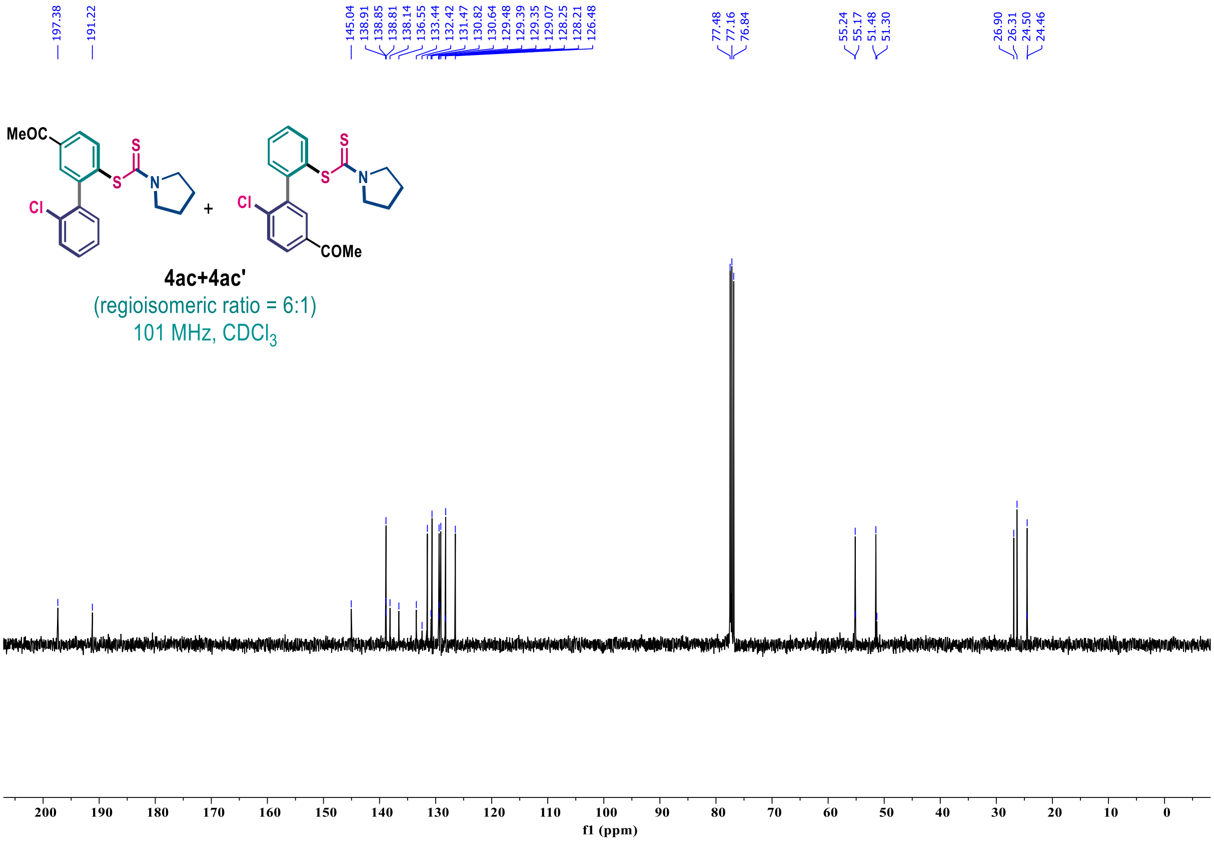

MeOC
Cl
S
S
N
+
COMe
4ac+4ac'
(regioisomeric ratio = 6:1)
101 MHz, CDCl3
f1 (ppm)

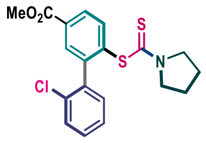

4ad
400 MHz, $CDCl_3$

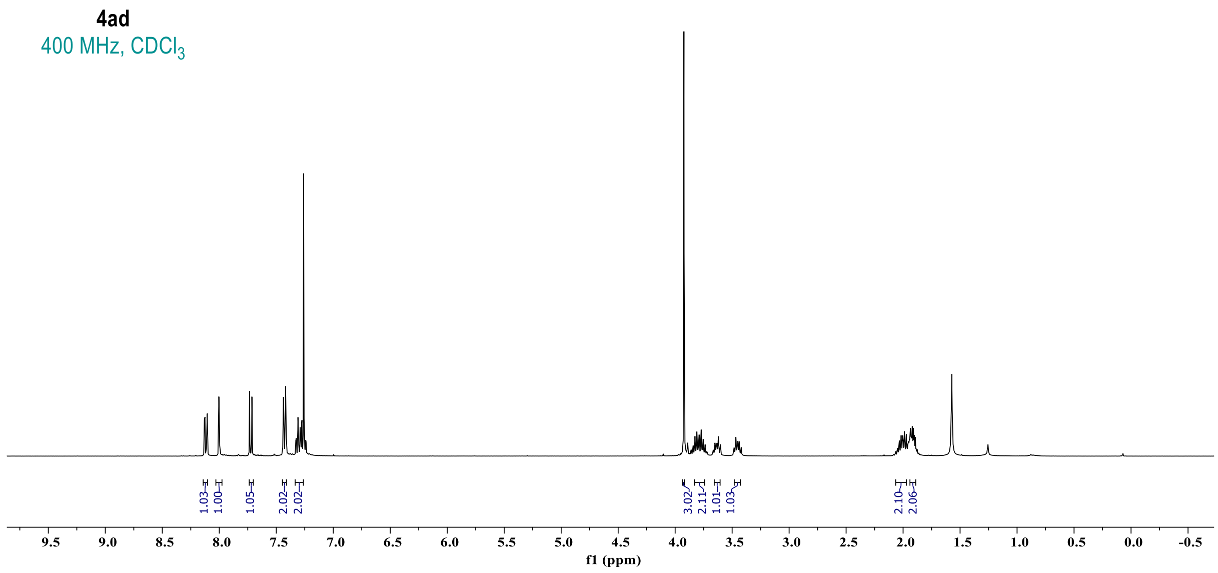

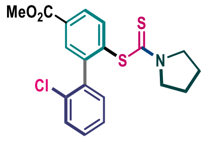

4ad
101 MHz, $CDCl_3$

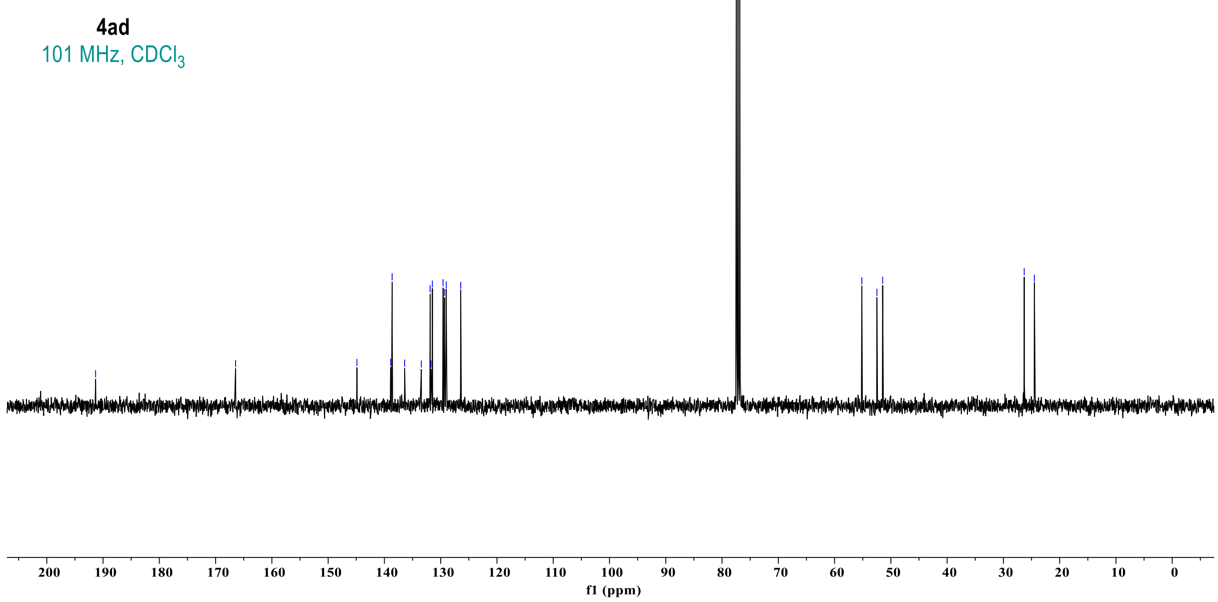

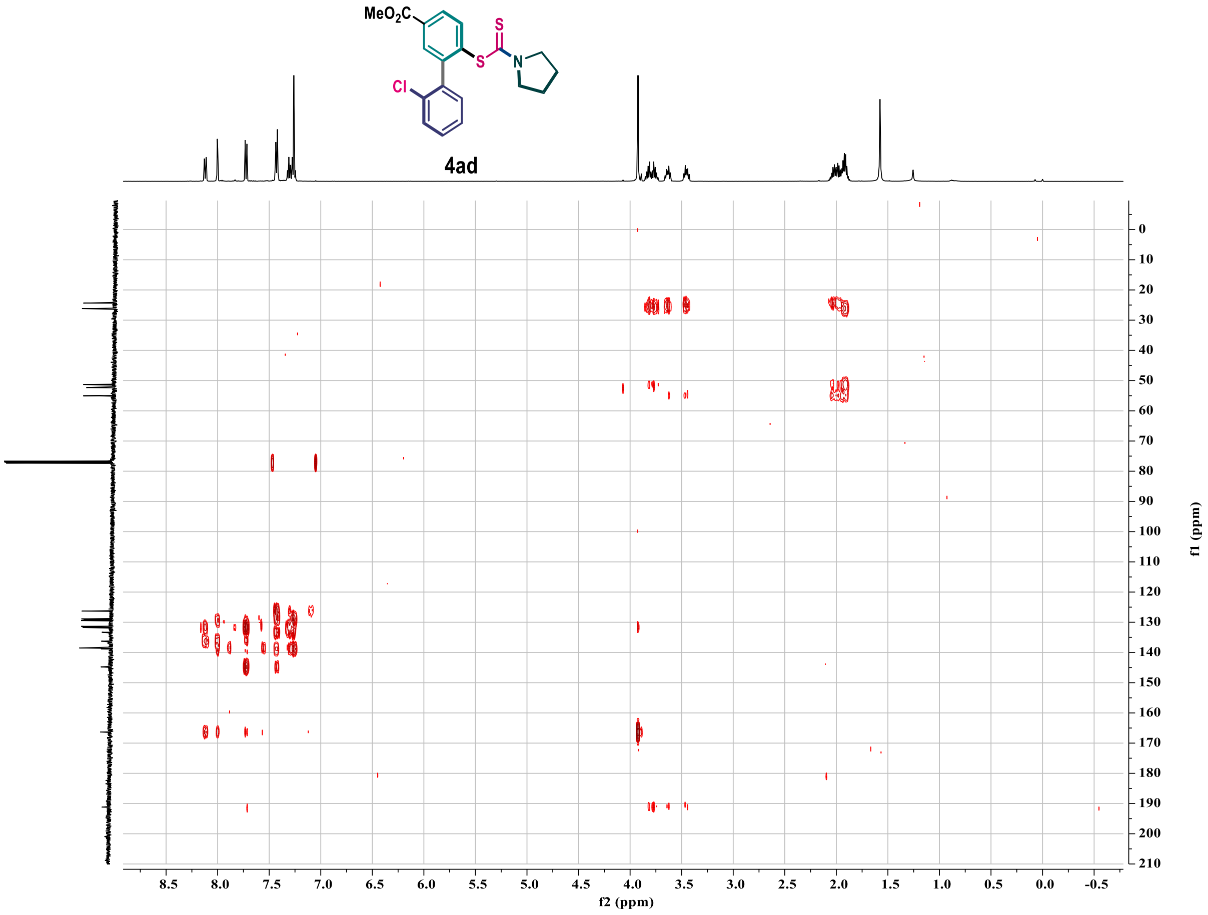
MeO2C
S
S
N
Cl
4ad
f2 (ppm)
f1 (ppm)

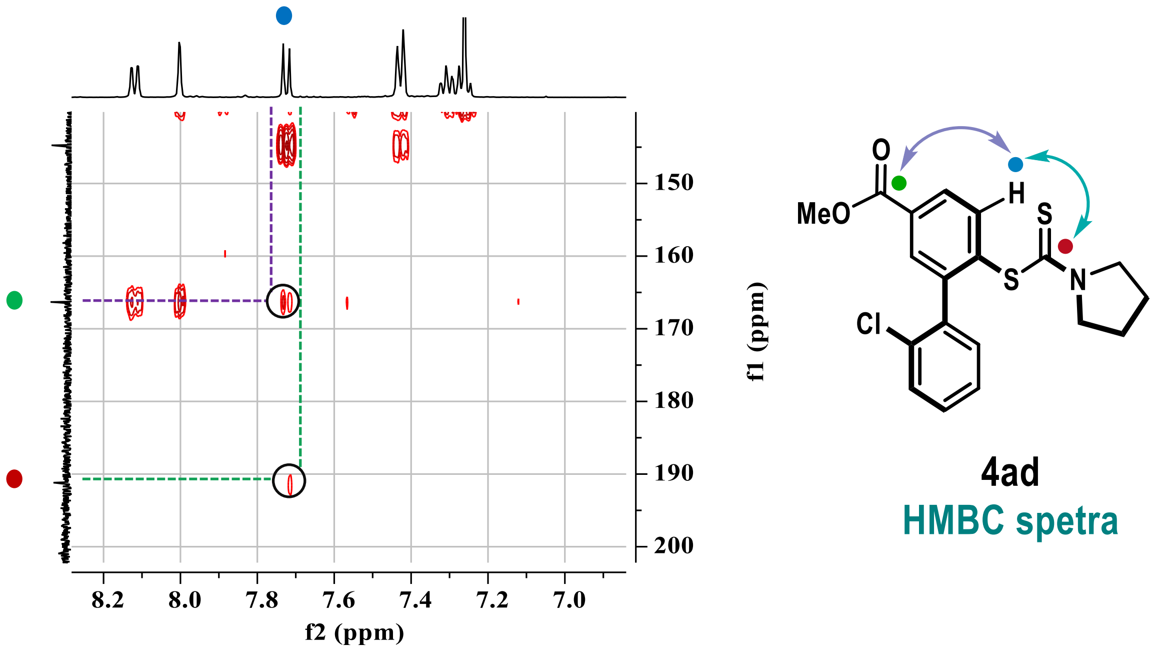
f2 (ppm)
f1 (ppm)
O
MeO
H
S
S
N
Cl
4ad
HMBC spetra

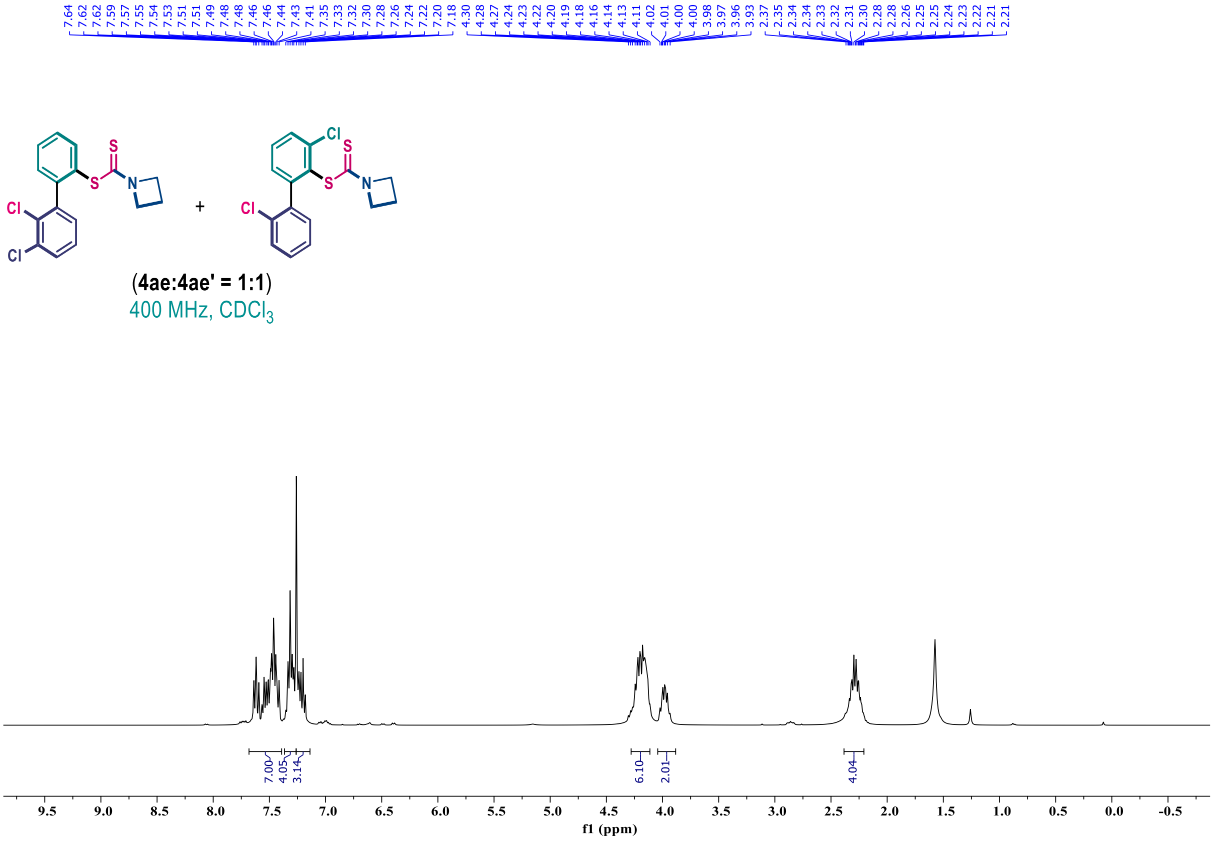
7.64 7.62 7.62 7.59 7.57 7.55 7.54 7.53 7.51 7.51 7.49 7.48 7.48 7.46 7.44 7.43 7.41 7.35 7.33 7.32 7.30 7.28 7.26 7.24 7.22 7.20 7.18 4.30 4.28 4.27 4.24 4.23 4.22 4.20 4.19 4.18 4.16 4.14 4.13 4.11 4.02 4.01 4.00 3.98 3.97 3.96 3.93 2.37 2.35 2.34 2.34 2.33 2.32 2.31 2.30 2.28 2.28 2.26 2.25 2.25 2.24 2.23 2.22 2.21 2.21
Cl
Cl
S
S
N
+
Cl
Cl
S
S
N
(4ae:4ae' = 1:1)
400 MHz, CDCl3
7.00
4.05
3.14
6.10
2.01
4.04
f1 (ppm)

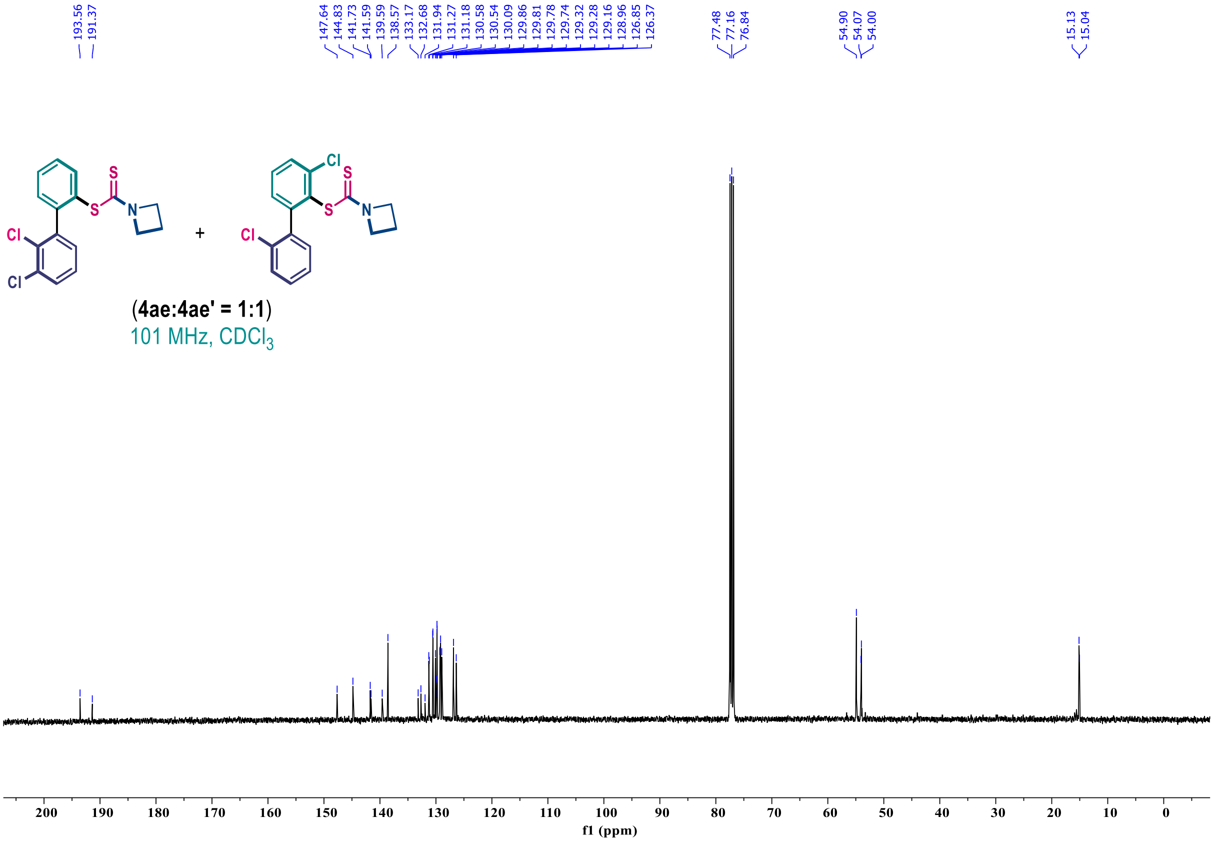
193.56 191.37
147.64 144.83 141.73 141.59 139.59 138.57 133.17 132.68 131.94 131.27 131.18 130.58 130.54 130.09 129.86 129.81 129.78 129.74 129.32 129.28 129.16 128.96 126.85 126.37
77.48 77.16 76.84
54.90 54.07 54.00
15.13 15.04
Cl
Cl
S
S
N
+
Cl
Cl
S
S
N
(4ae:4ae' = 1:1)
101 MHz, CDCl3
f1 (ppm)

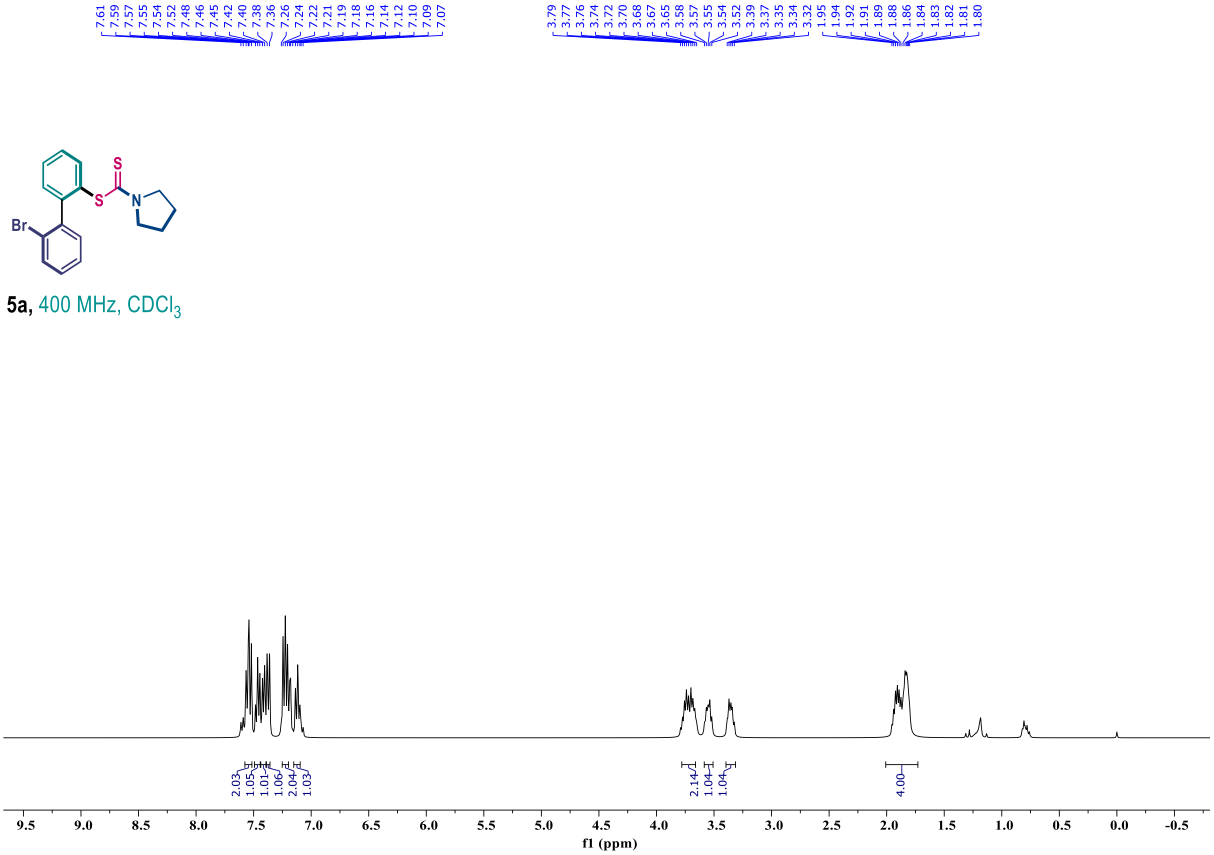
7.61 7.59 7.57 7.55 7.54 7.52 7.48 7.46 7.45 7.42 7.40 7.38 7.36 7.26 7.24 7.22 7.21 7.19 7.18 7.16 7.14 7.12 7.10 7.09 7.07
3.79 3.77 3.76 3.74 3.72 3.70 3.68 3.67 3.65 3.58 3.57 3.55 3.54 3.52 3.39 3.37 3.35 3.34 3.32 1.95 1.94 1.92 1.91 1.89 1.88 1.86 1.84 1.83 1.82 1.81 1.80
Br
S
S
N
5a, 400 MHz, CDCl3
2.03 1.05 1.01 1.06 2.04 1.03
2.14 1.04 1.04
4.00
f1 (ppm)

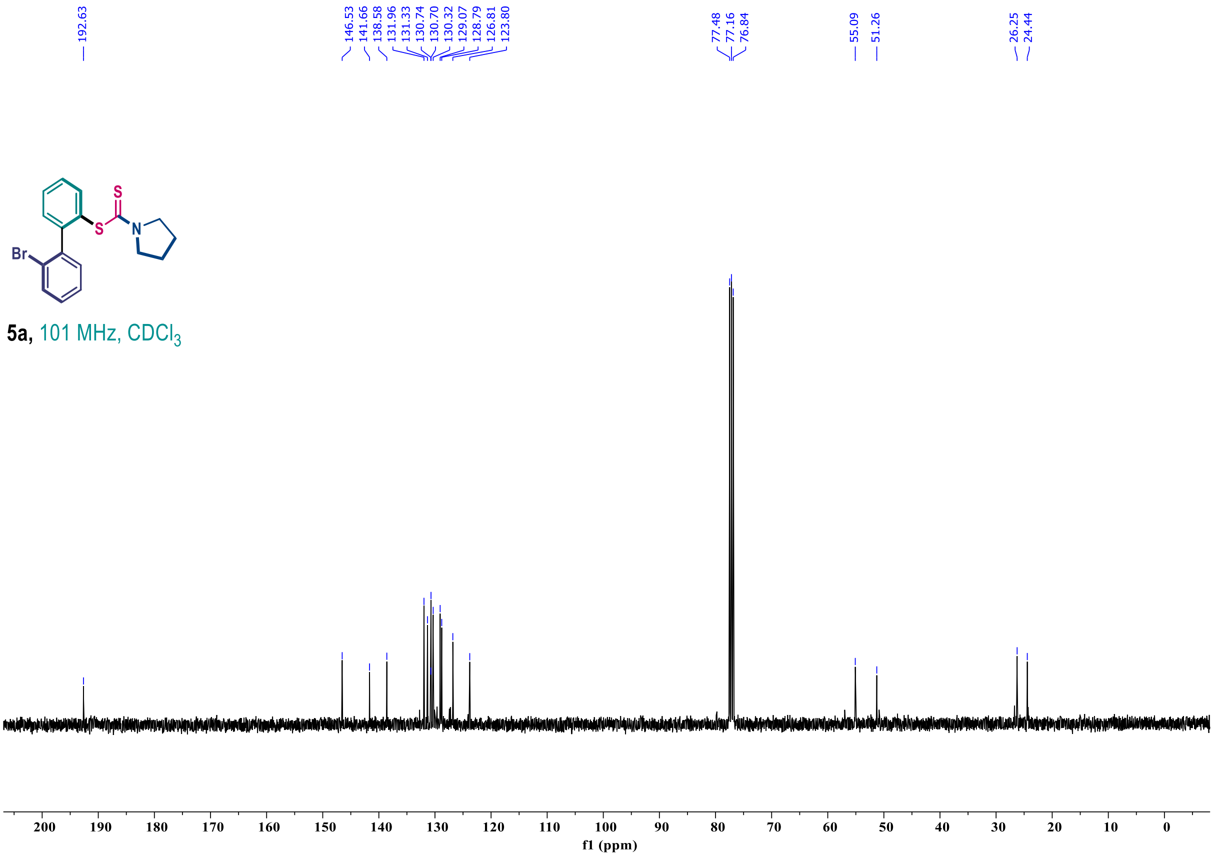
192.63
146.53 141.66 138.58 131.96 131.33 130.74 130.70 130.32 129.07 128.79 126.81 123.80
77.48 77.16 76.84
55.09 51.26
26.25 24.44
Br
S
S
N
5a, 101 MHz, CDCl3
f1 (ppm)

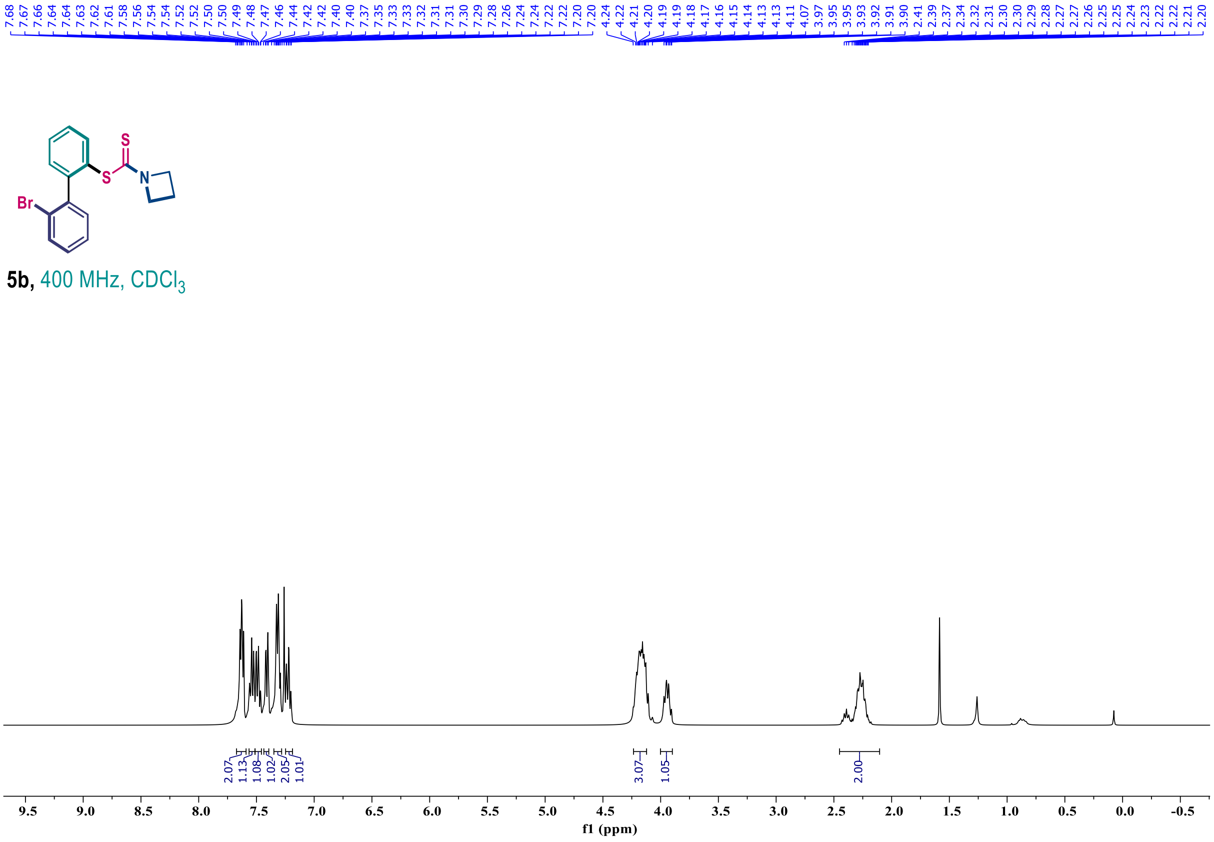

5b, 400 MHz, $CDCl_3$

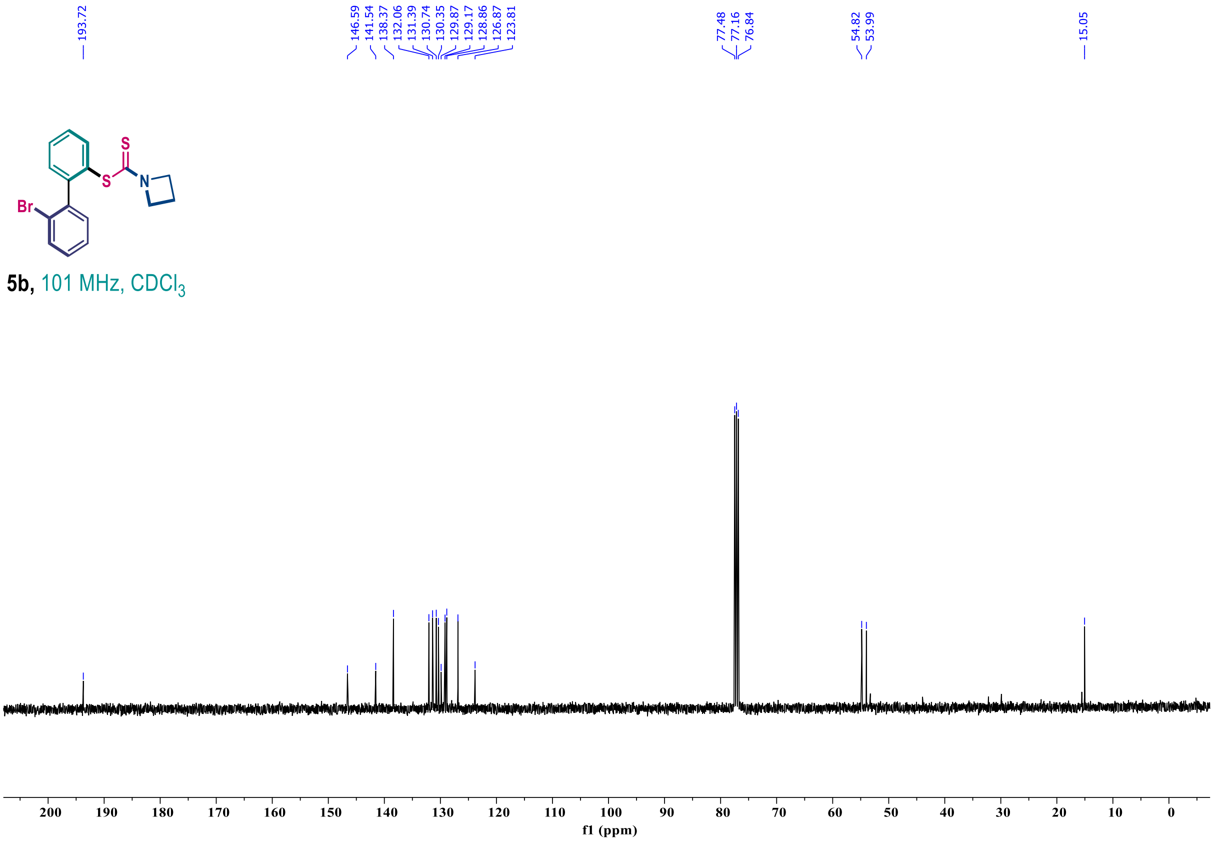

5b, 101 MHz, $CDCl_3$

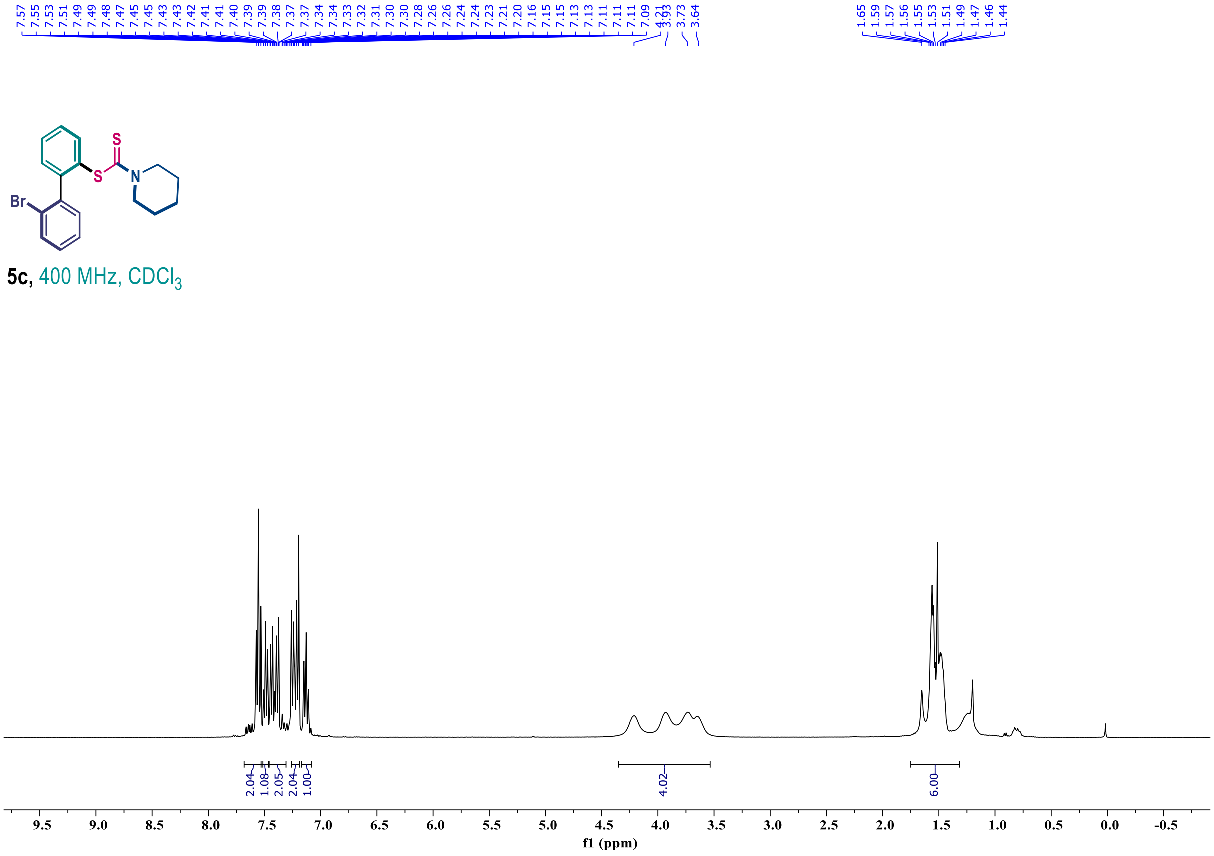
7.57 7.55 7.53 7.51 7.49 7.49 7.48 7.47 7.45 7.45 7.43 7.43 7.42 7.41 7.41 7.40 7.39 7.39 7.38 7.37 7.37 7.34 7.34 7.33 7.32 7.31 7.30 7.30 7.28 7.26 7.26 7.24 7.24 7.23 7.21 7.20 7.16 7.15 7.15 7.13 7.13 7.11 7.11 7.09 4.21 3.93 3.73 3.64
1.65 1.59 1.57 1.56 1.55 1.53 1.51 1.49 1.47 1.46 1.44
Br
S
S
N
5c, 400 MHz, CDCl3
2.04 1.08 2.05 2.04 1.00
4.02
6.00
9.5 9.0 8.5 8.0 7.5 7.0 6.5 6.0 5.5 5.0 4.5 4.0 3.5 3.0 2.5 2.0 1.5 1.0 0.5 0.0 -0.5
f1 (ppm)

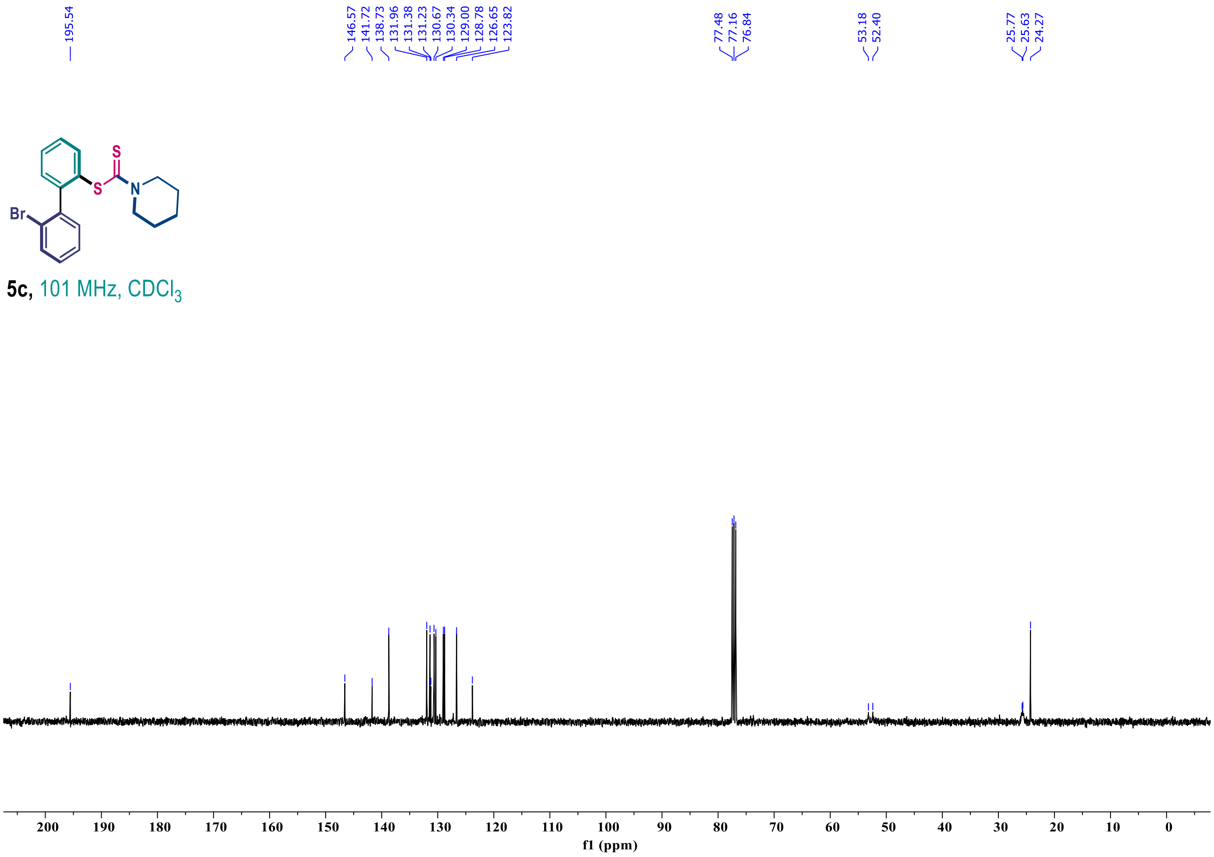
195.54
146.57 141.72 138.73 131.96 131.38 131.23 130.67 130.34 129.00 128.78 126.65 123.82
77.48 77.16 76.84
53.18 52.40
25.77 25.63 24.27
Br
S
S
N
5c, 101 MHz, CDCl3
200 190 180 170 160 150 140 130 120 110 100 90 80 70 60 50 40 30 20 10 0
f1 (ppm)

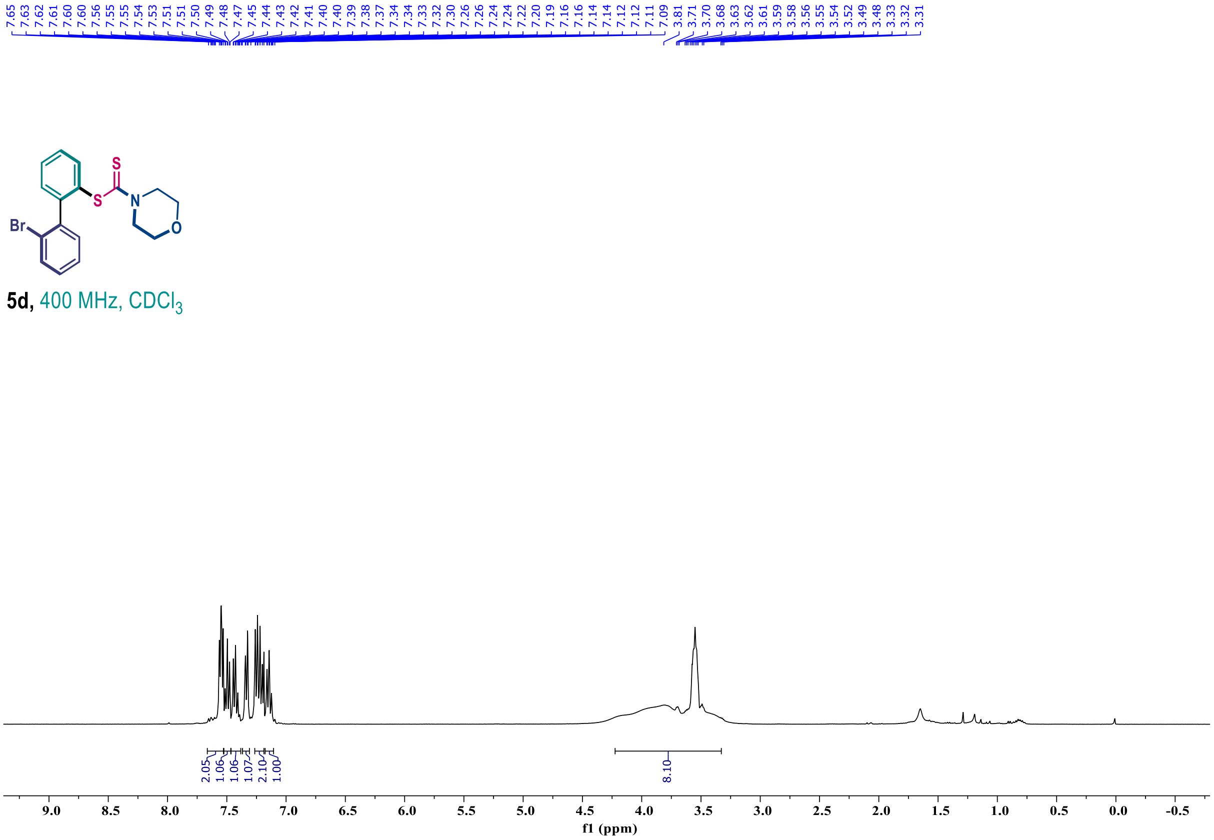
7.65 7.63 7.61 7.60 7.56 7.55 7.55 7.54 7.53 7.51 7.51 7.50 7.49 7.48 7.47 7.45 7.44 7.43 7.42 7.41 7.40 7.40 7.39 7.38 7.37 7.34 7.34 7.33 7.32 7.30 7.26 7.26 7.24 7.24 7.22 7.20 7.19 7.16 7.16 7.14 7.14 7.12 7.12 7.11 7.09 3.81 3.71 3.70 3.68 3.63 3.62 3.61 3.59 3.58 3.56 3.55 3.54 3.52 3.49 3.48 3.33 3.32 3.31
Br
S
S
N
O
5d, 400 MHz, $CDCl_3$
2.05 1.06 1.06 1.07 2.10 1.00
8.10
f1 (ppm)

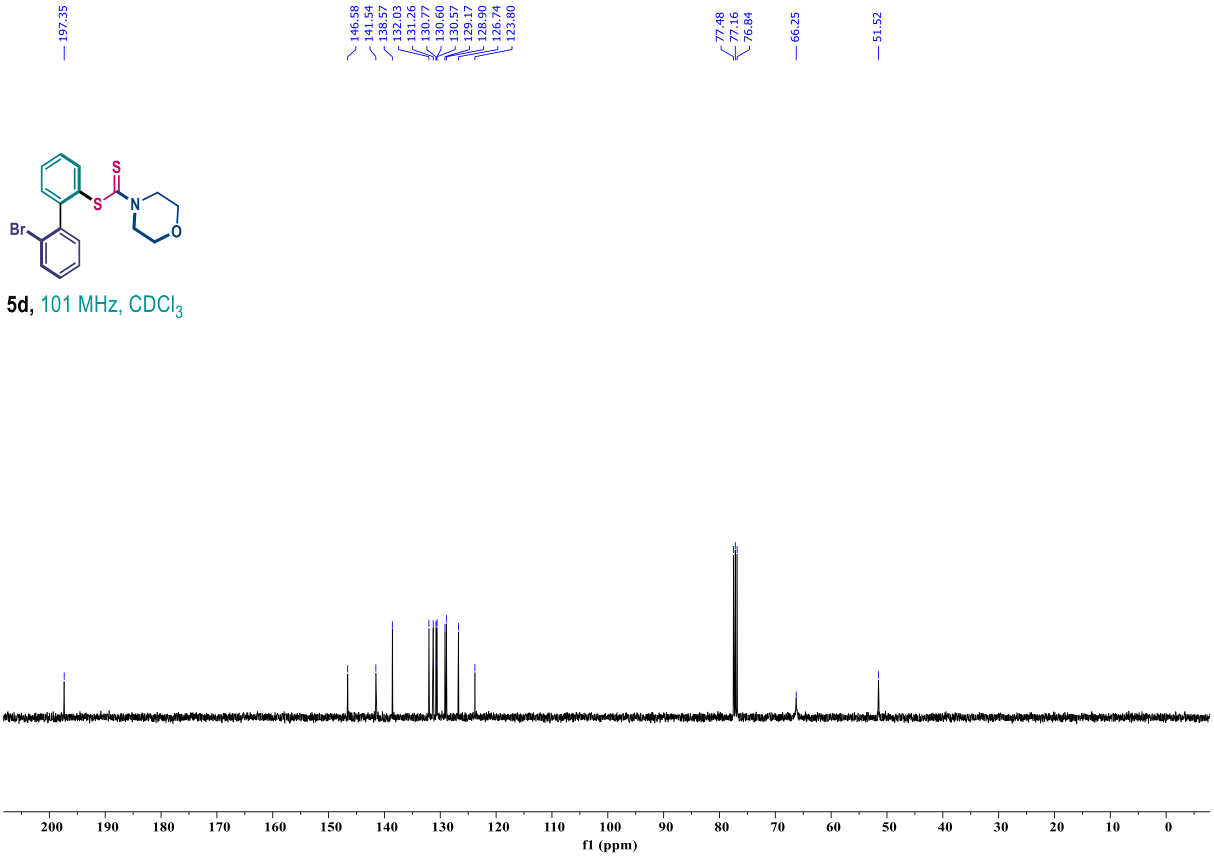
197.35
146.58 141.54 138.57 132.03 131.26 130.77 130.60 130.57 129.17 128.90 126.74 123.80
77.48 77.16 76.84
66.25
51.52
Br
S
S
N
O
5d, 101 MHz, $CDCl_3$
f1 (ppm)

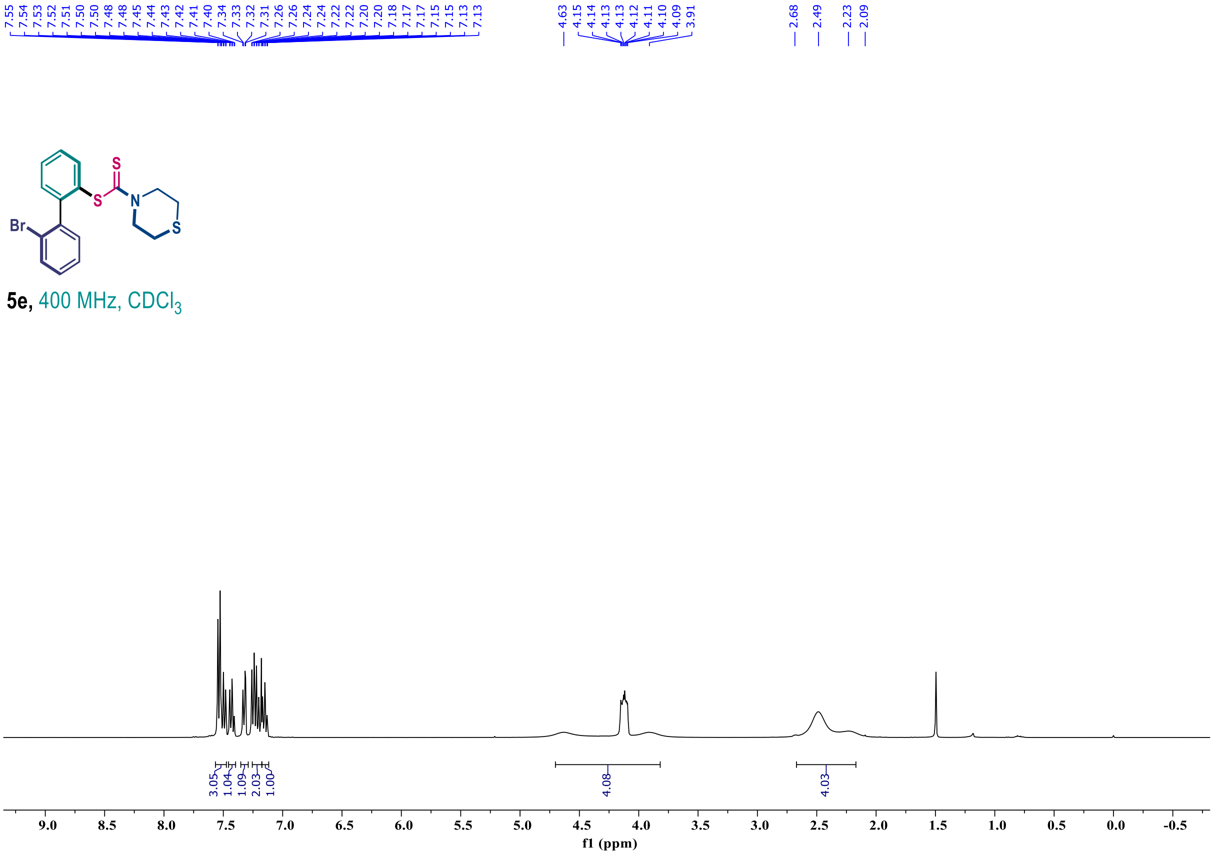

5e, 400 MHz, $CDCl_3$

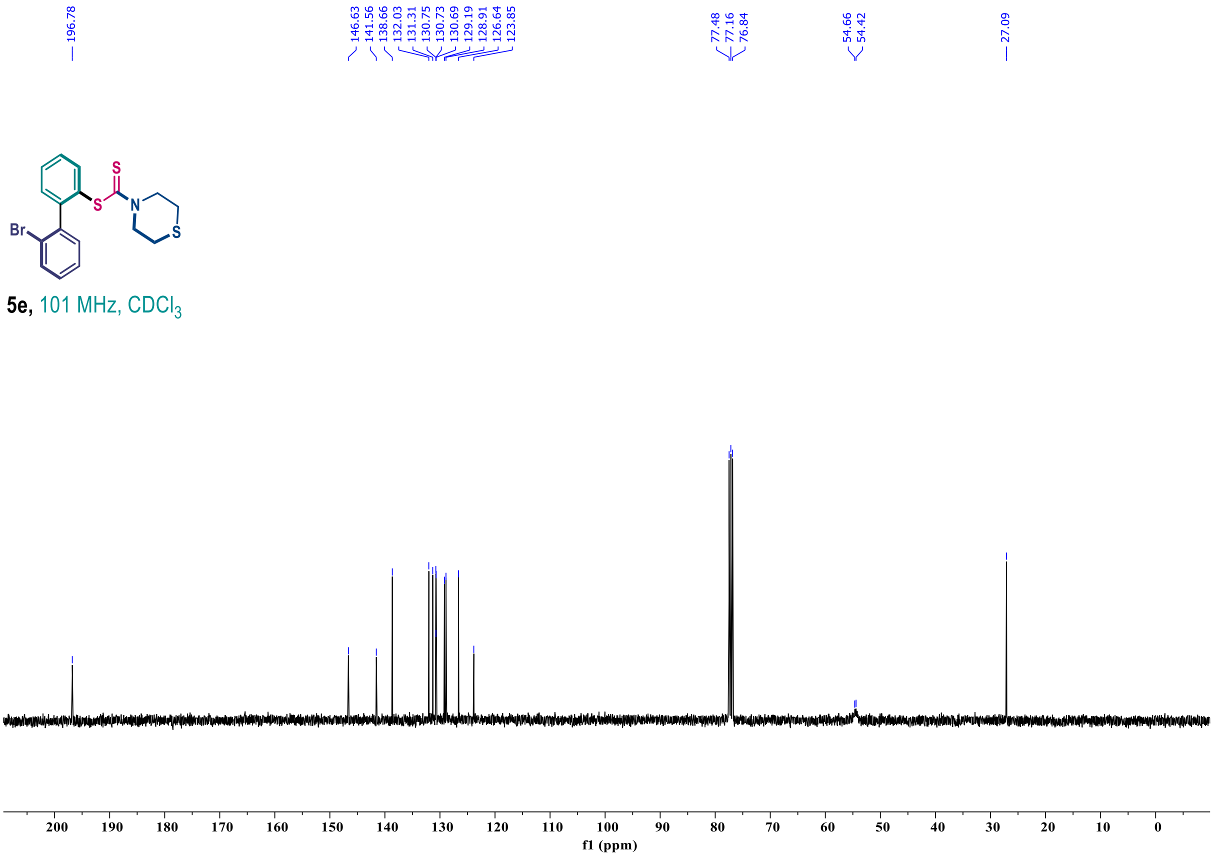

5e, 101 MHz, $CDCl_3$

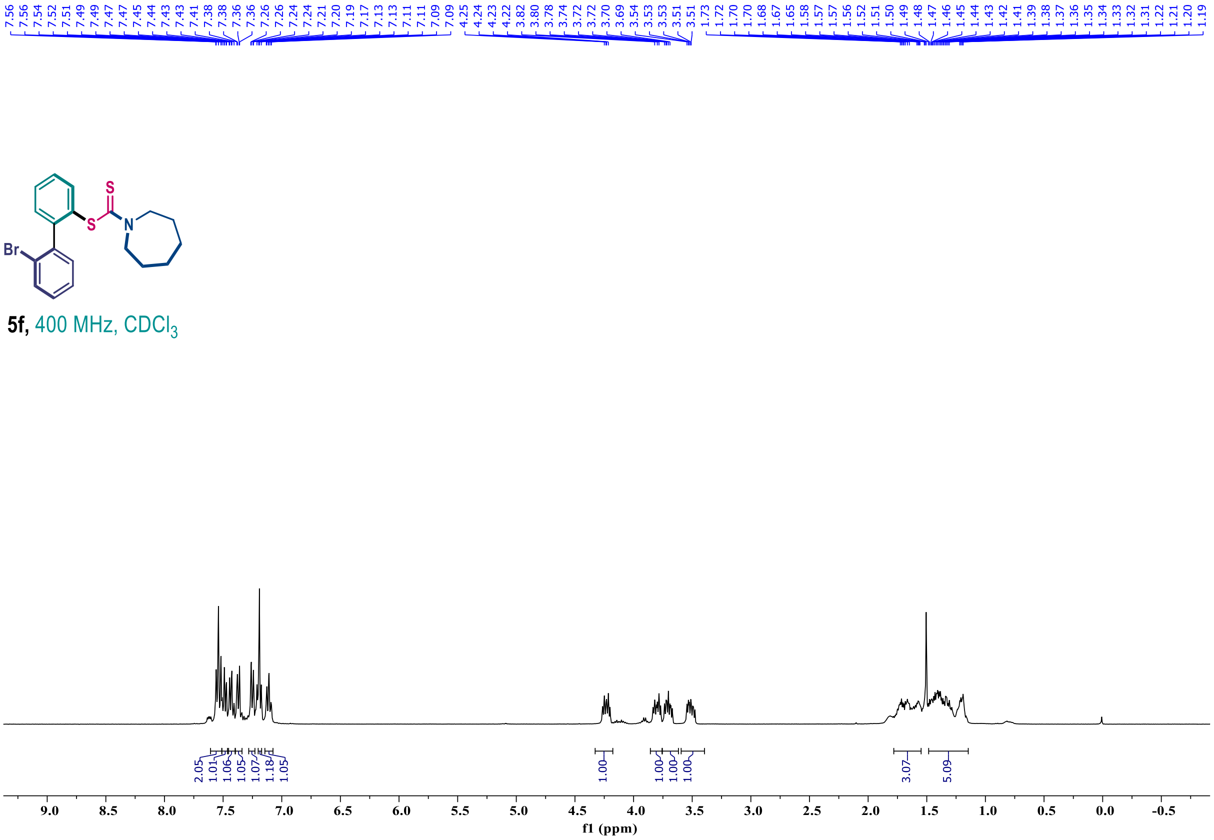
7.56 7.56 7.54 7.52 7.51 7.49 7.49 7.47 7.47 7.45 7.44 7.43 7.43 7.41 7.38 7.38 7.36 7.36 7.26 7.26 7.24 7.24 7.21 7.20 7.19 7.17 7.13 7.13 7.11 7.09 7.09 4.25 4.24 4.23 4.22 3.82 3.80 3.78 3.74 3.72 3.72 3.70 3.69 3.54 3.53 3.53 3.51 3.51 1.73 1.72 1.70 1.70 1.68 1.67 1.65 1.58 1.57 1.57 1.56 1.52 1.51 1.50 1.49 1.48 1.47 1.46 1.45 1.44 1.43 1.42 1.41 1.39 1.38 1.37 1.36 1.35 1.34 1.33 1.32 1.31 1.22 1.21 1.20 1.19
S
S
N
Br
5f, 400 MHz, CDCl3
2.05 1.01 1.06 1.05 1.07 1.18 1.05 1.00 1.00 1.00 1.00 3.07 5.09
f1 (ppm)

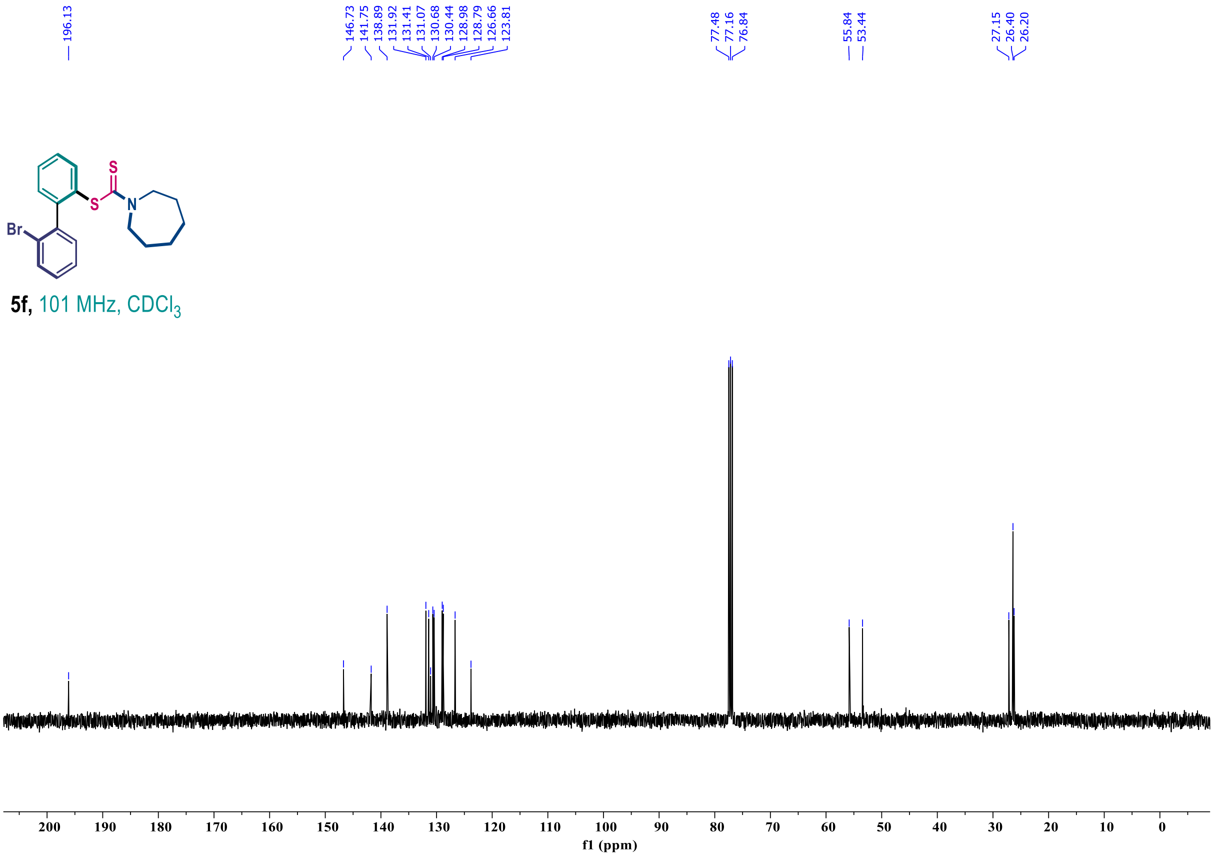
196.13
146.73 141.75 138.89 131.92 131.41 131.07 130.68 130.44 128.98 128.79 126.66 123.81
77.48 77.16 76.84
55.84 53.44
27.15 26.40 26.20
S
S
N
Br
5f, 101 MHz, CDCl3
f1 (ppm)

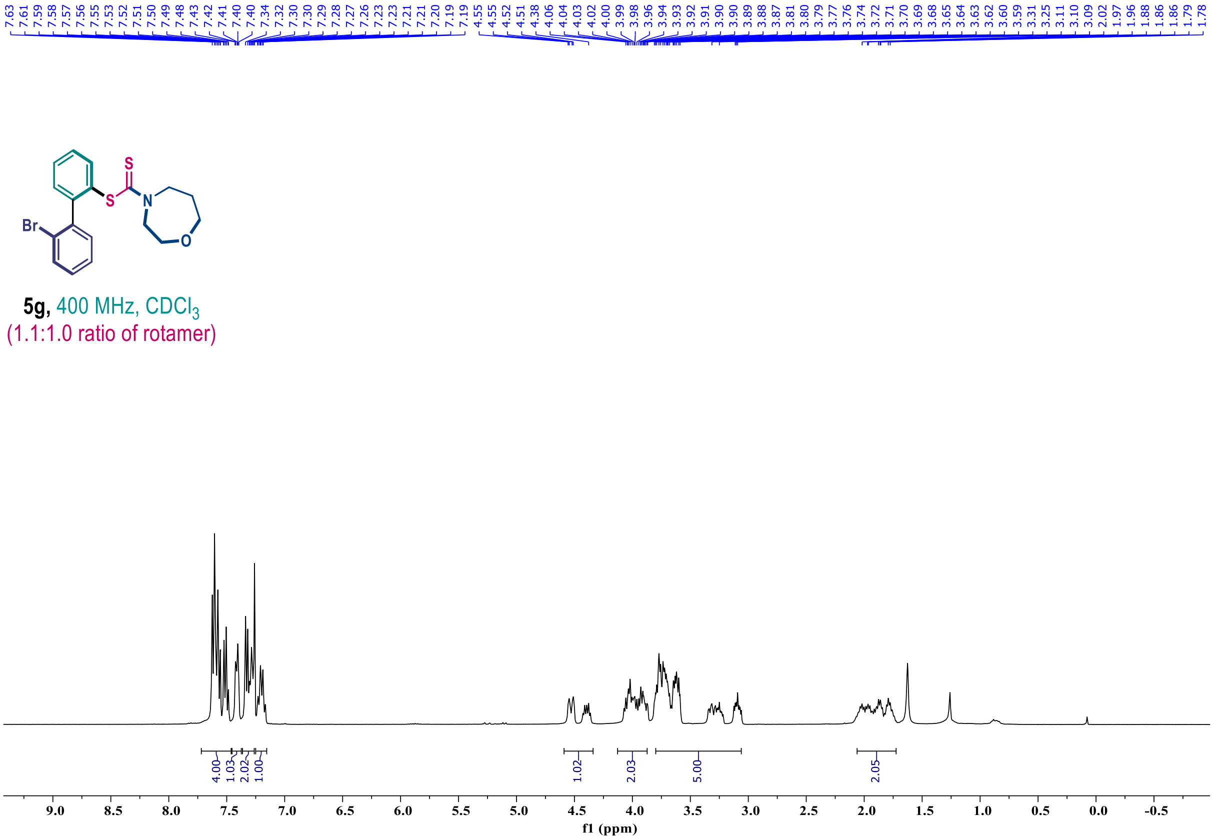

5g, 400 MHz, $CDCl_3$
(1.1:1.0 ratio of rotamer)

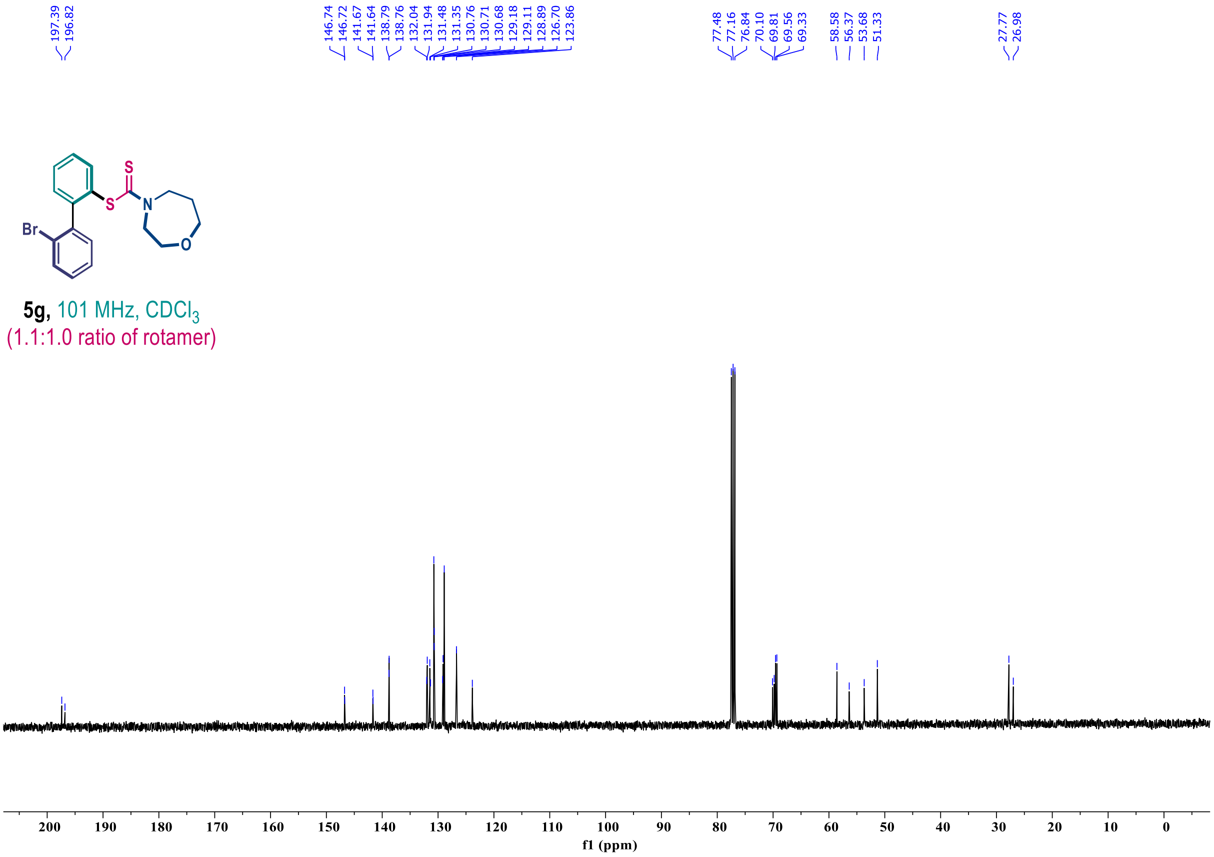

5g, 101 MHz, $CDCl_3$
(1.1:1.0 ratio of rotamer)

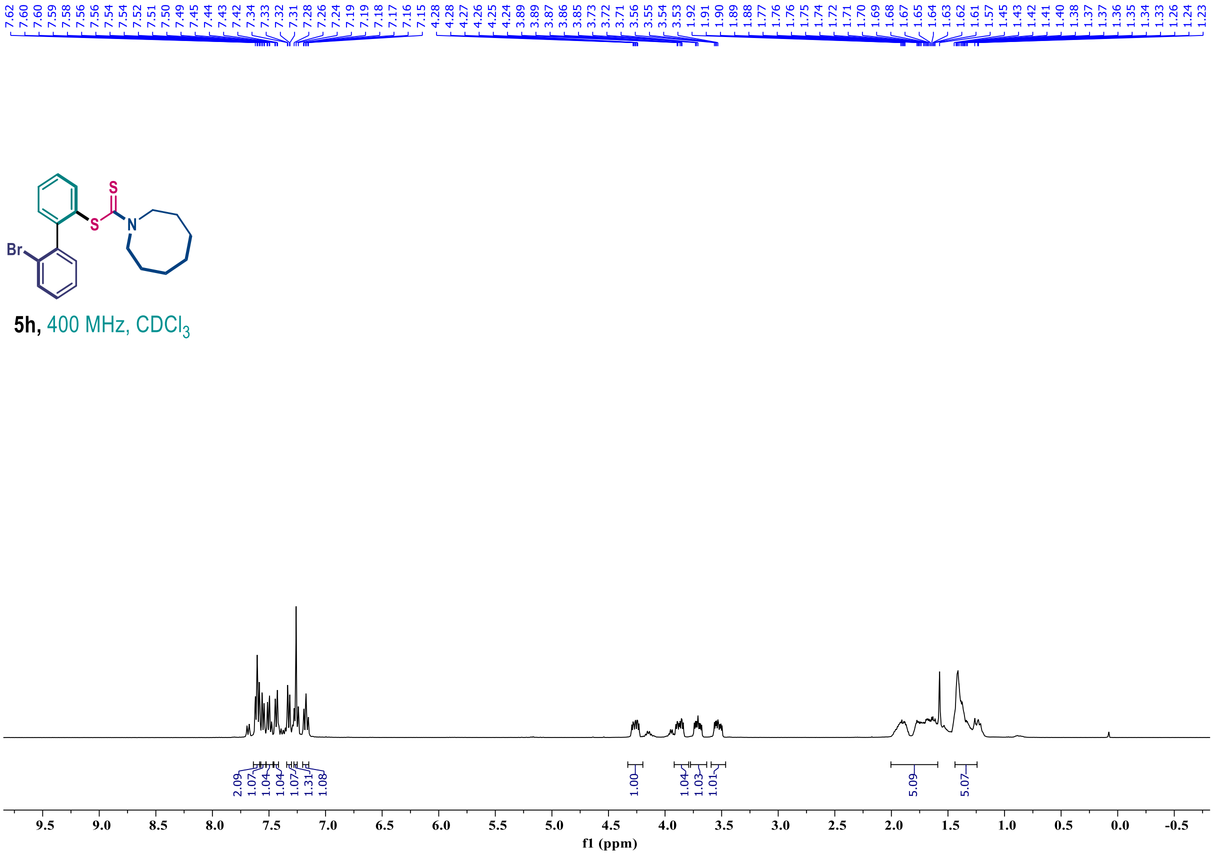

5h, 400 MHz, $CDCl_3$

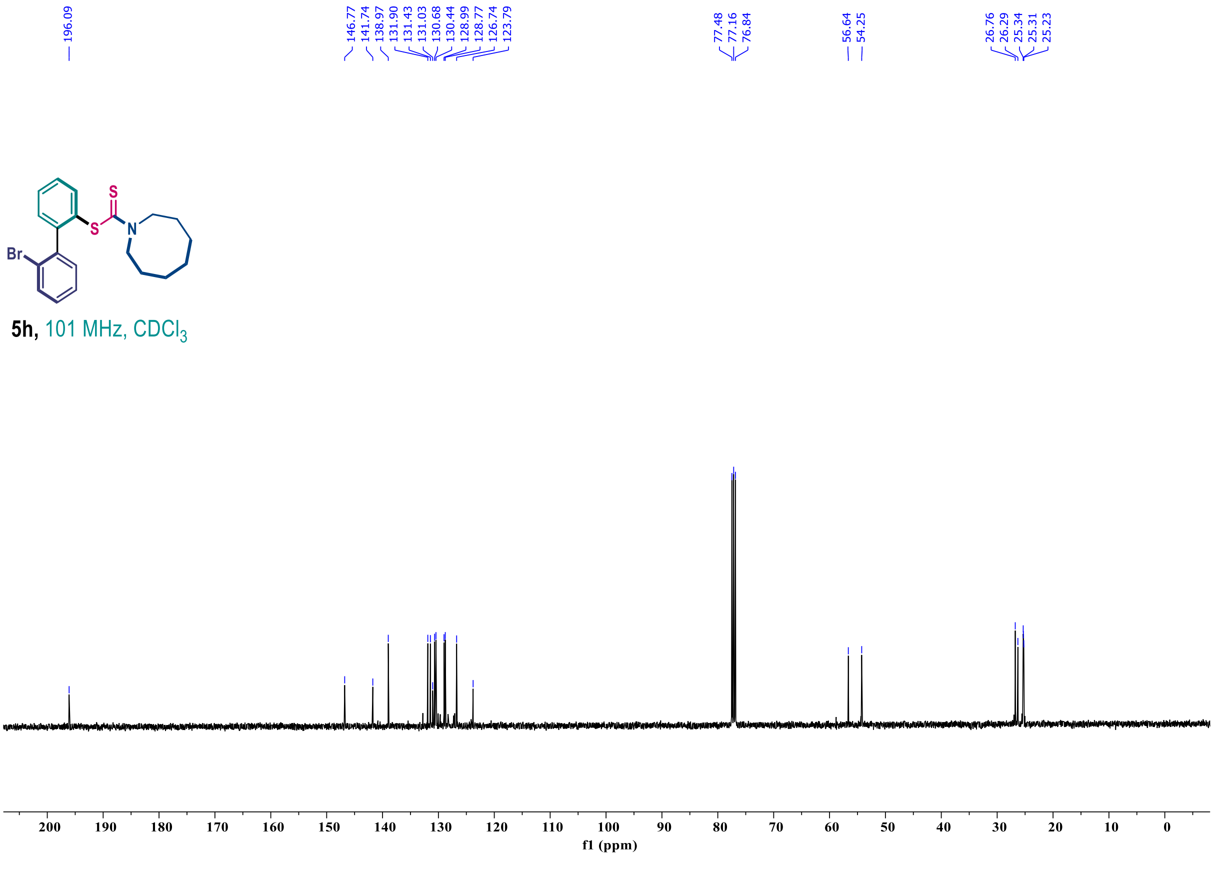

5h, 101 MHz, $CDCl_3$

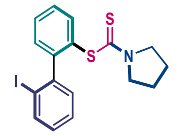

6a, 400 MHz, $CDCl_3$

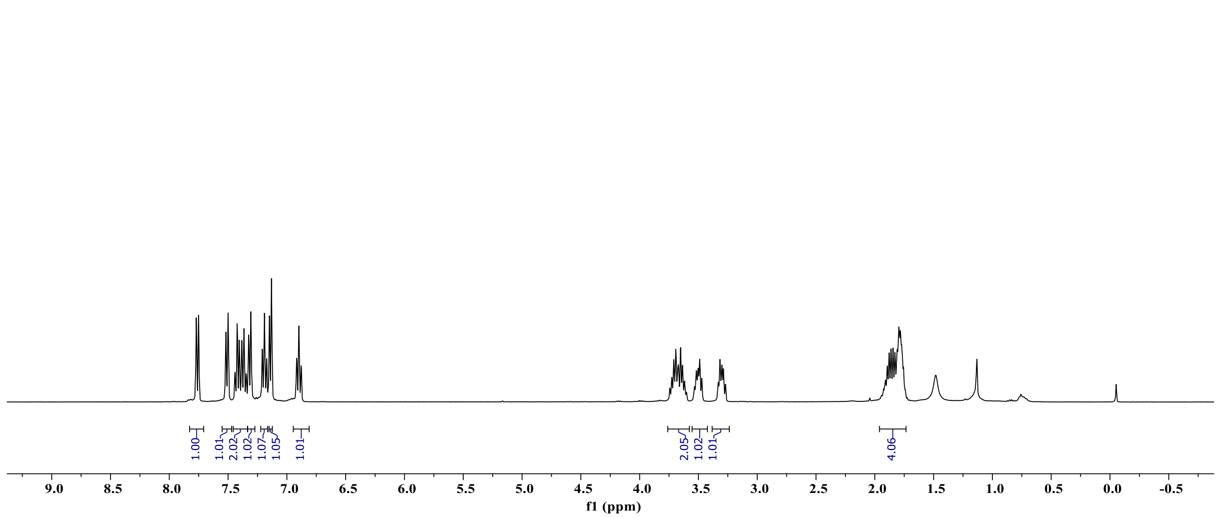

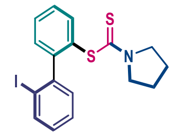

6a, 101 MHz, $CDCl_3$

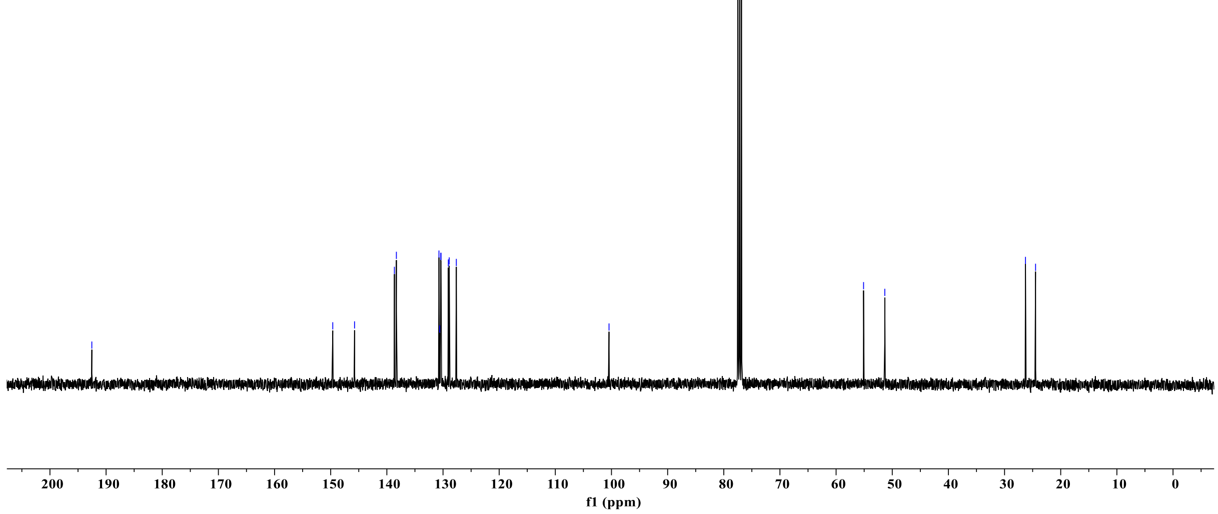

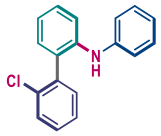

8a, 400 MHz, $CDCl_3$

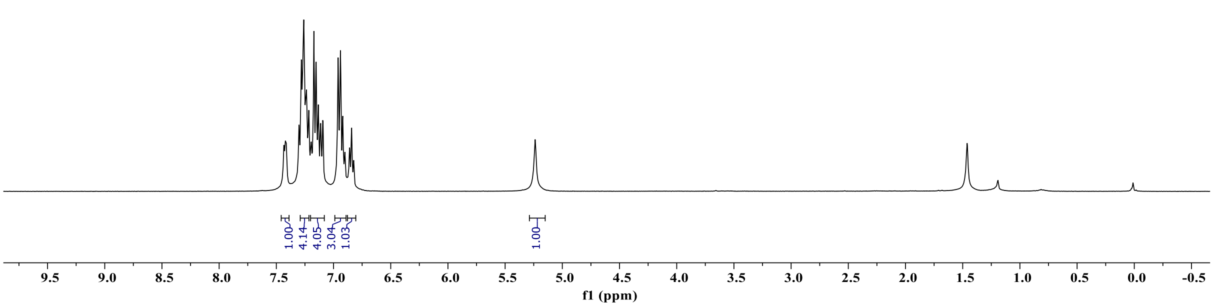

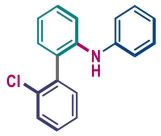

8a, 101 MHz, $CDCl_3$

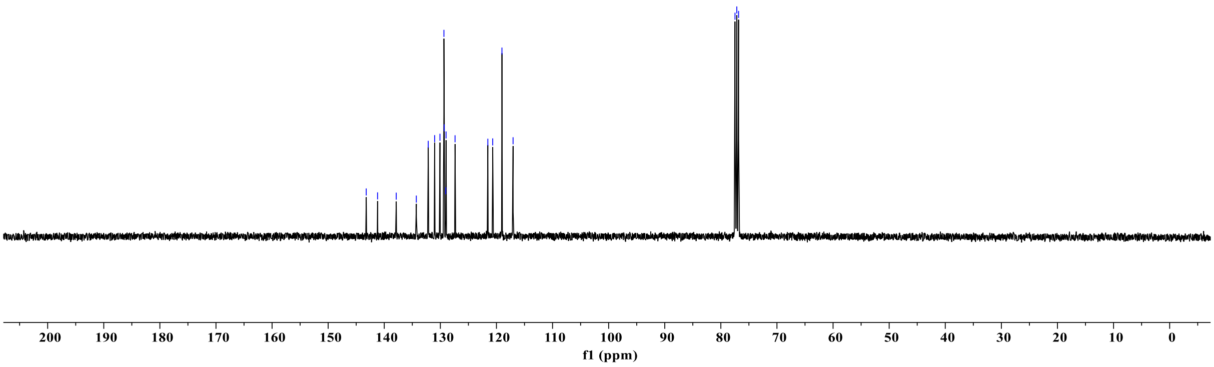

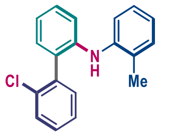

8b, 400 MHz, $CDCl_3$

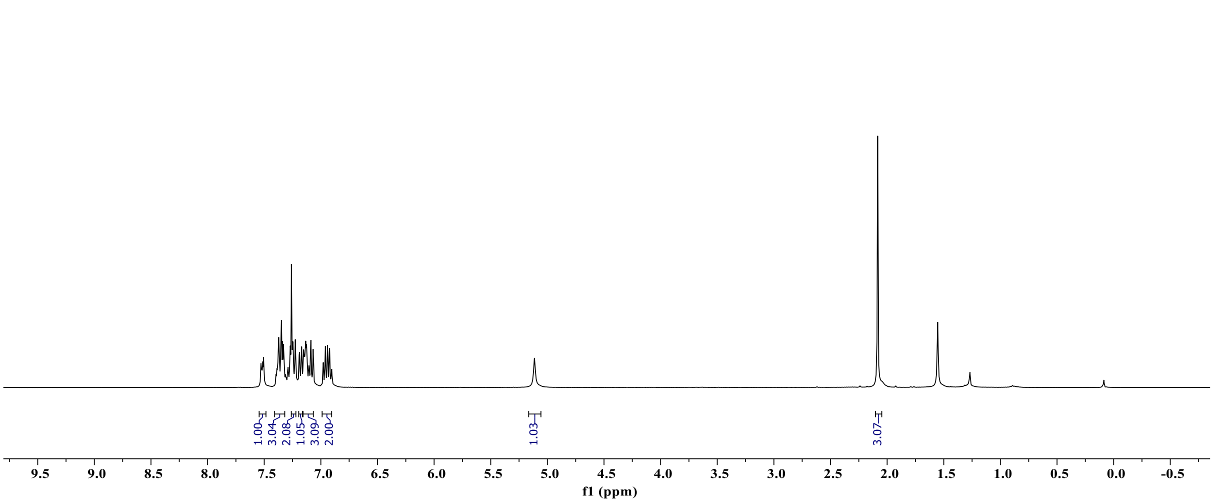

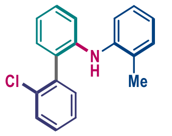

8b, 101 MHz, $CDCl_3$

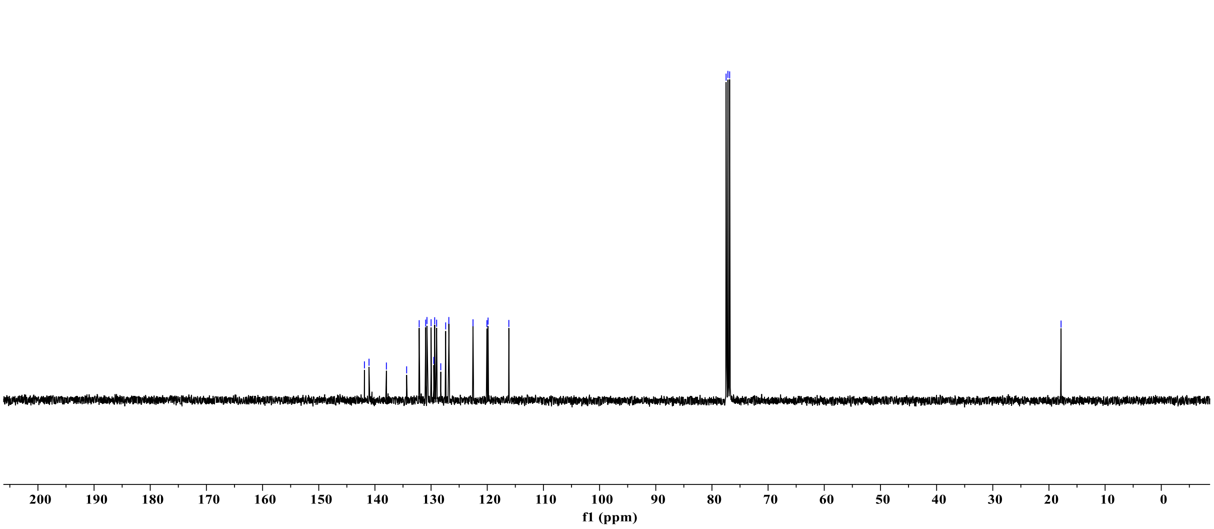

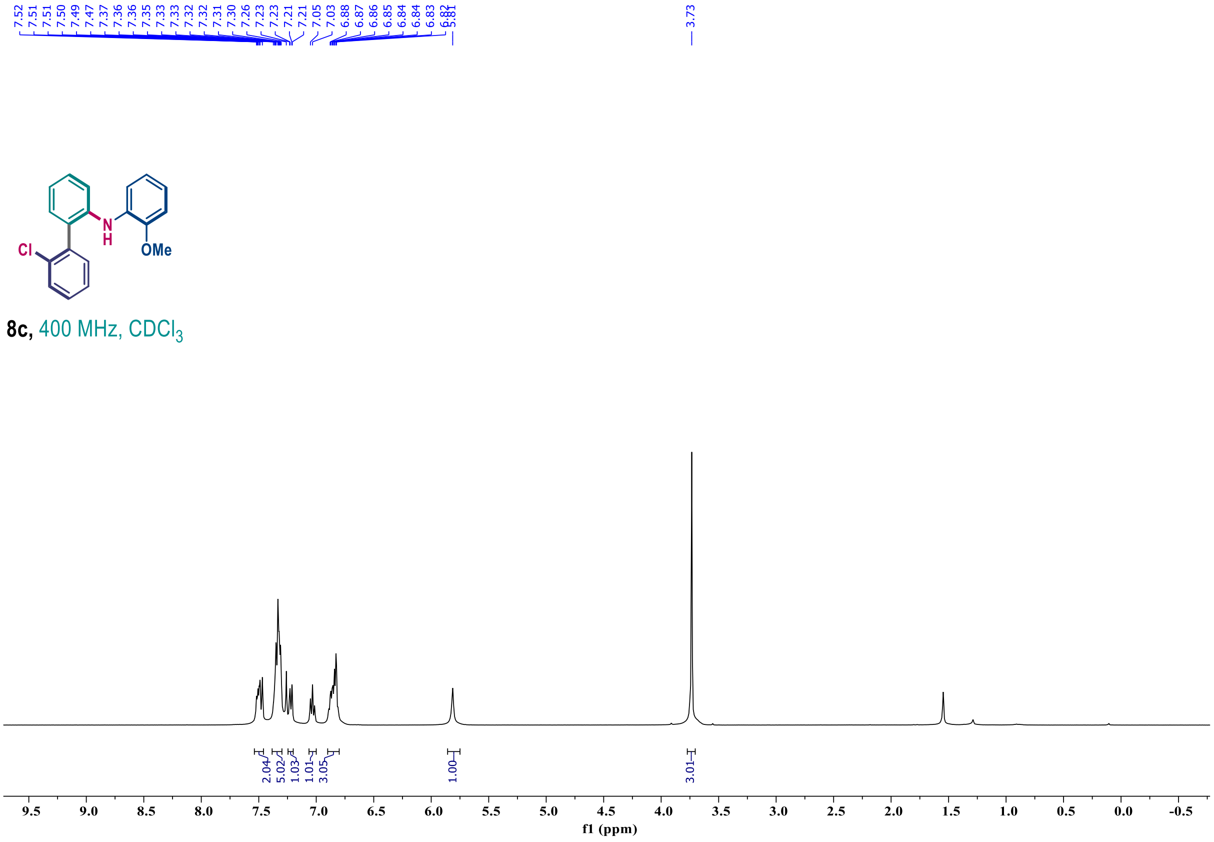
7.52 7.51 7.51 7.50 7.49 7.47 7.37 7.36 7.36 7.35 7.33 7.33 7.32 7.32 7.31 7.30 7.26 7.23 7.23 7.21 7.21 7.05 7.03 6.88 6.87 6.86 6.85 6.84 6.84 6.83 6.82 5.81
3.73
Cl
N
H
OMe
8c, 400 MHz, CDCl3
2.04 5.02 1.03 1.01 3.05 1.00 3.01
9.5 9.0 8.5 8.0 7.5 7.0 6.5 6.0 5.5 5.0 4.5 4.0 3.5 3.0 2.5 2.0 1.5 1.0 0.5 0.0 -0.5
f1 (ppm)

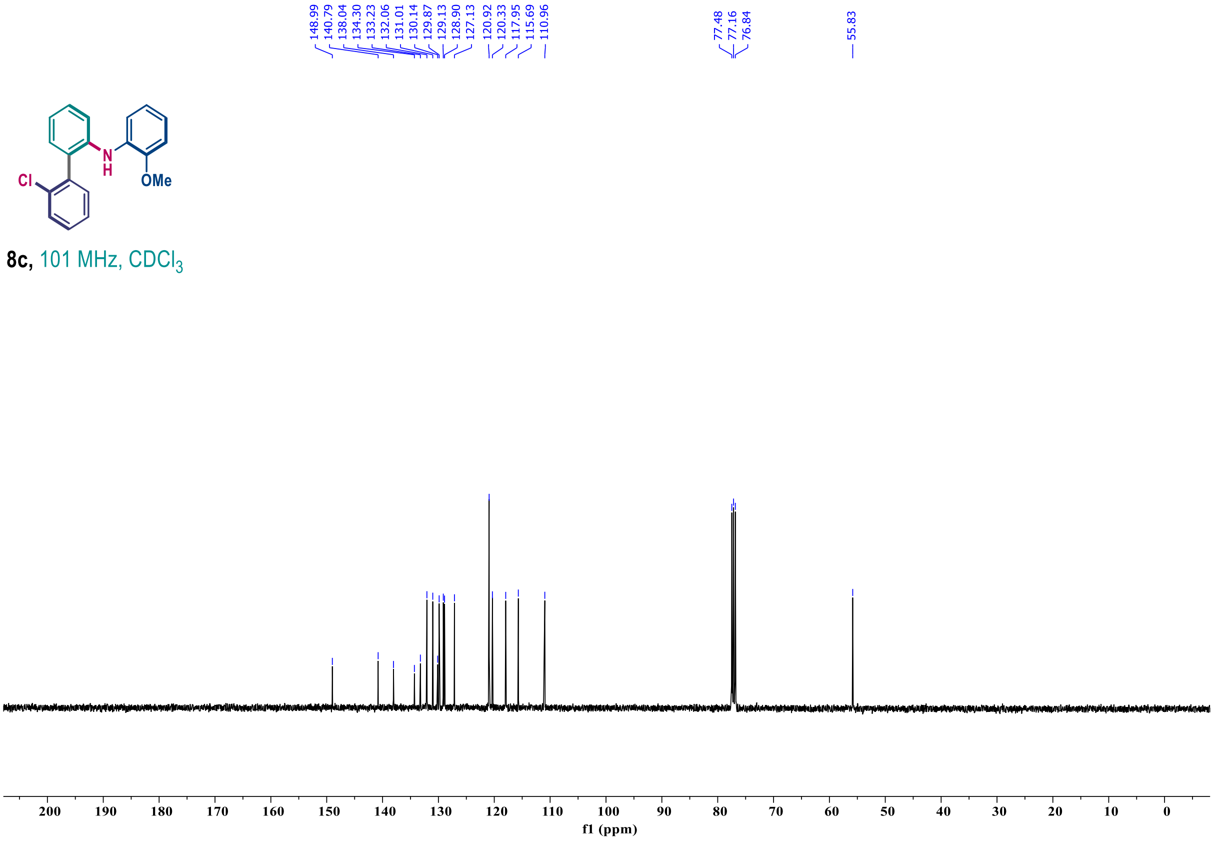
148.99 140.79 138.04 134.30 133.23 132.06 131.01 130.14 129.87 129.13 128.90 127.13 120.92 120.33 117.95 115.69 110.96
77.48 77.16 76.84
55.83
Cl
N
H
OMe
8c, 101 MHz, CDCl3
200 190 180 170 160 150 140 130 120 110 100 90 80 70 60 50 40 30 20 10 0
f1 (ppm)

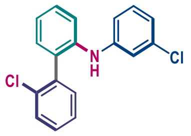

8d, 400 MHz, $CDCl_3$

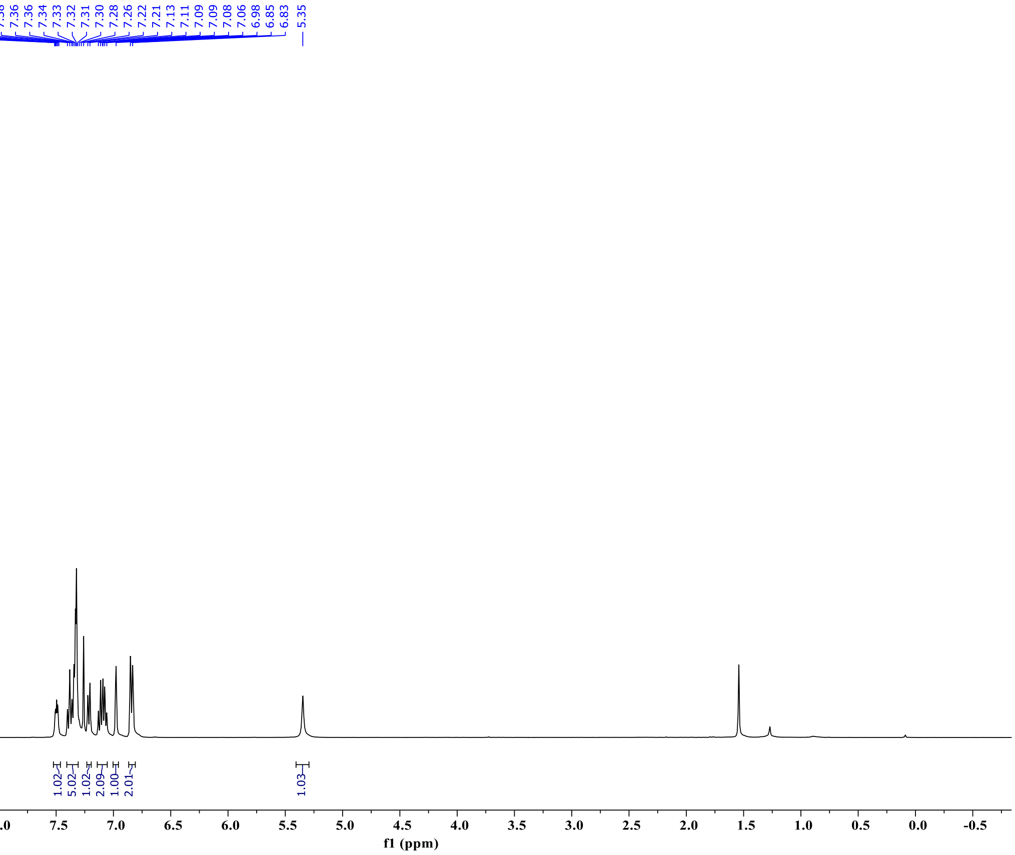

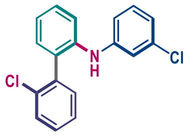

8d, 101 MHz, $CDCl_3$

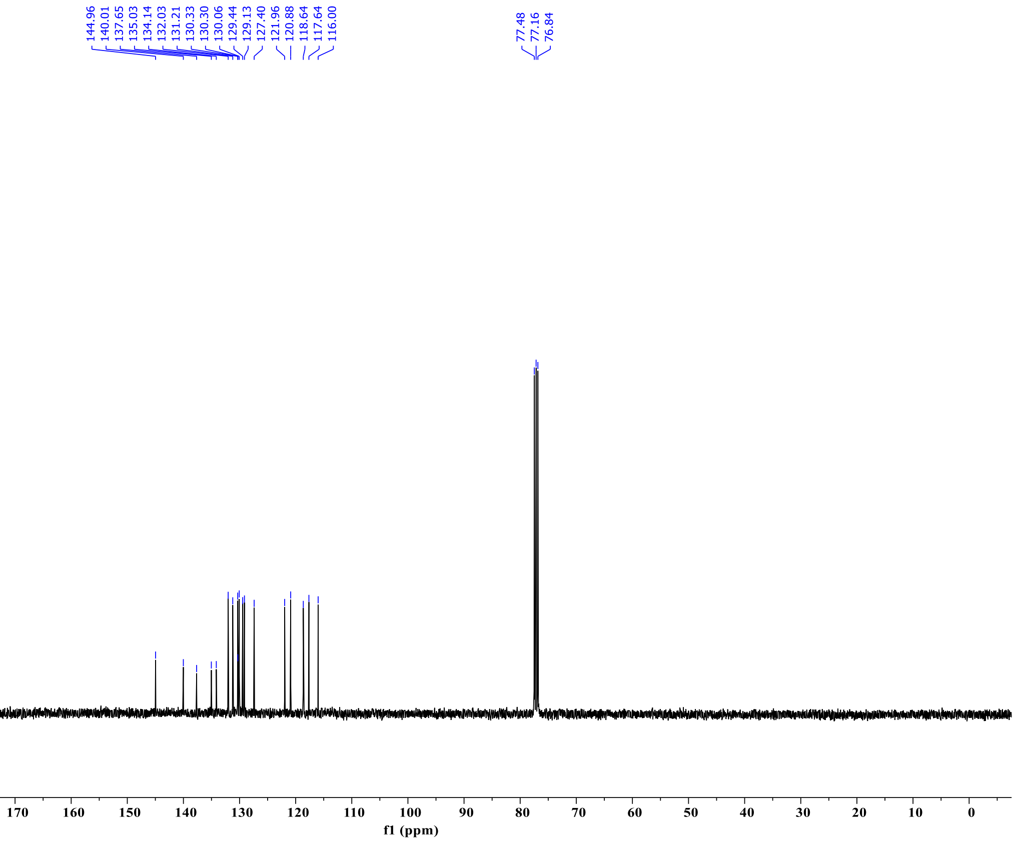

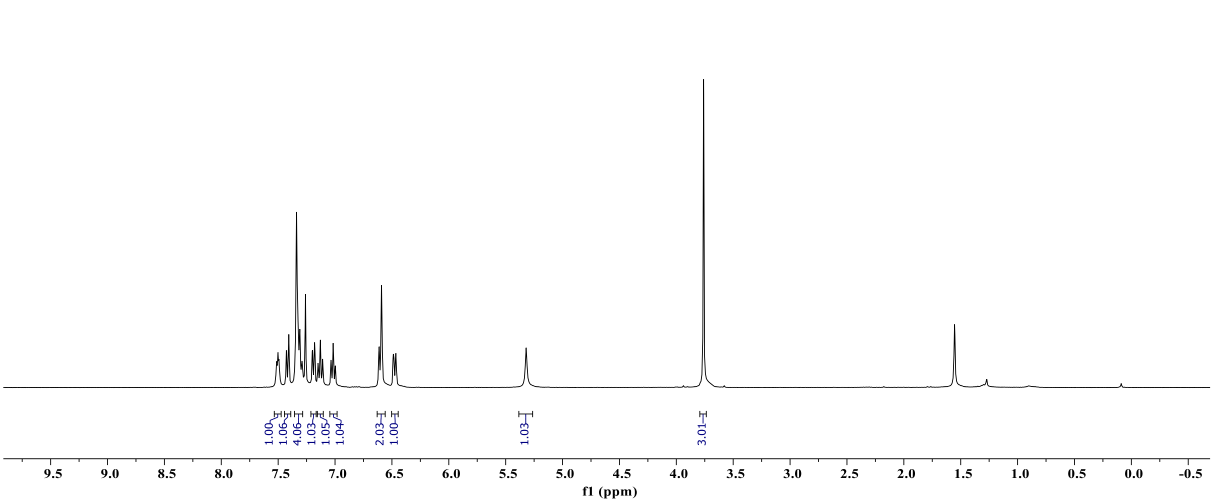

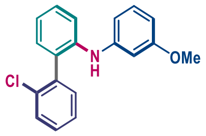

8e, 400 MHz, $CDCl_3$

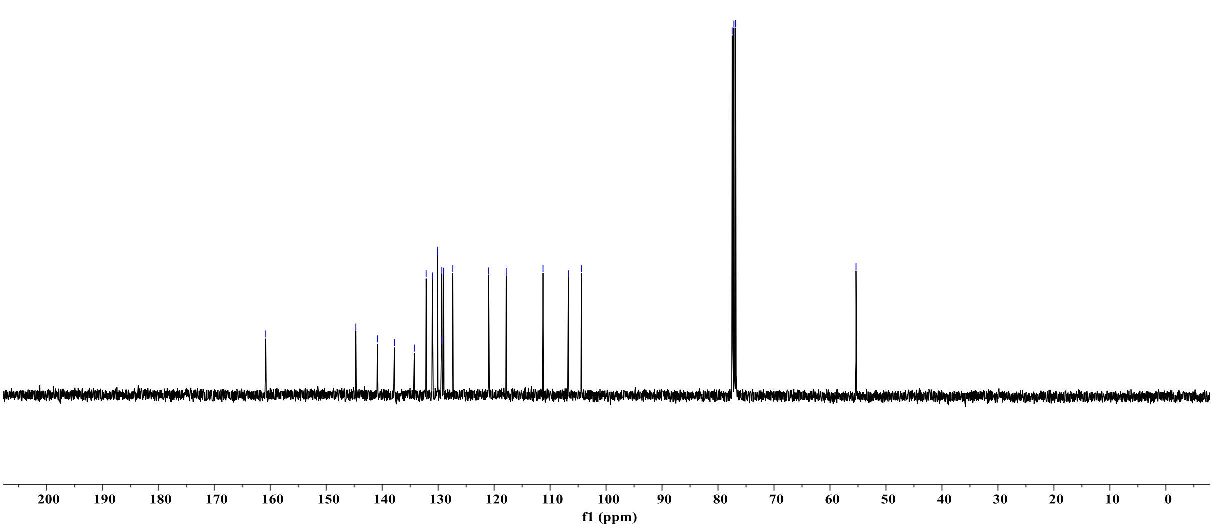

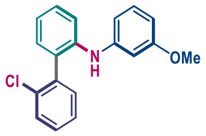

8e, 101 MHz, $CDCl_3$

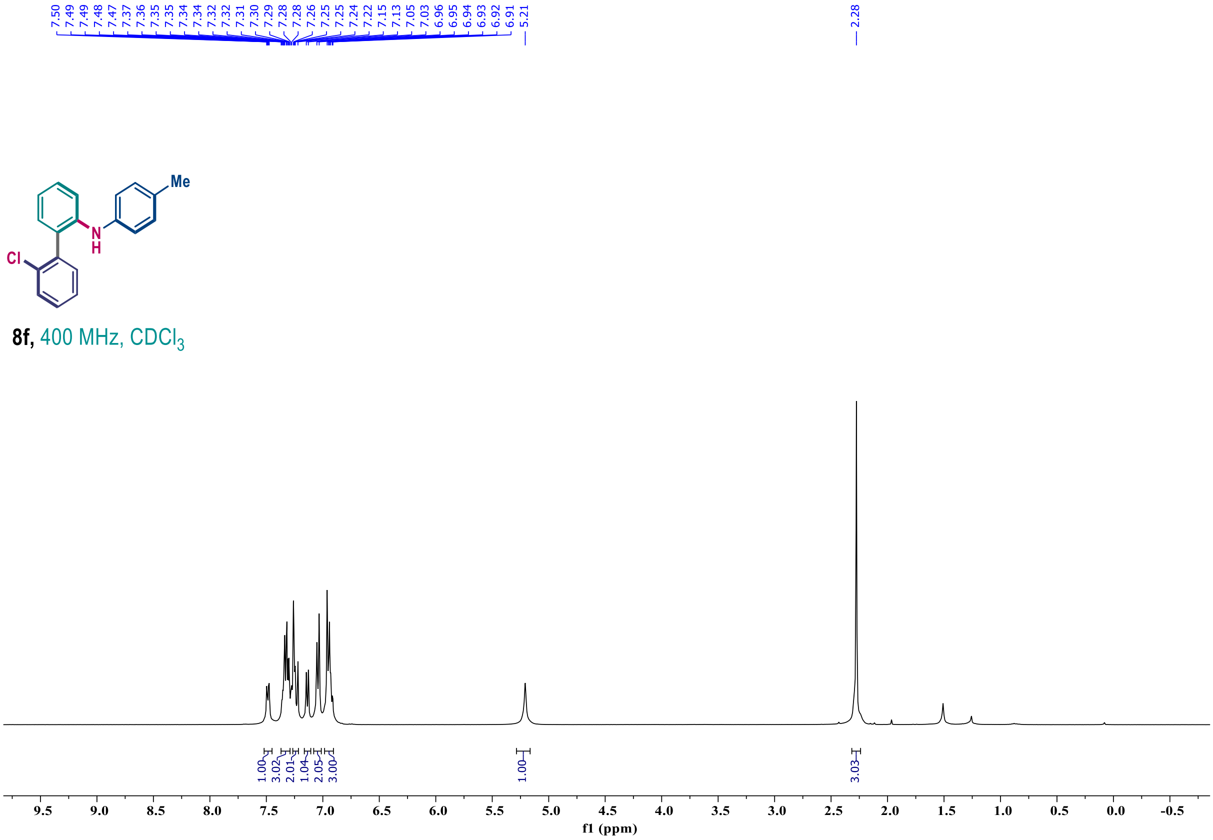
7.50
7.49
7.49
7.48
7.47
7.37
7.36
7.35
7.35
7.34
7.34
7.32
7.32
7.31
7.30
7.29
7.28
7.28
7.26
7.25
7.25
7.24
7.22
7.15
7.13
7.05
7.03
6.96
6.95
6.94
6.93
6.92
6.91
5.21
2.28
Me
N
H
Cl
8f, 400 MHz, CDCl3
1.00
3.02
2.01
1.04
2.05
3.00
1.00
3.03
f1 (ppm)

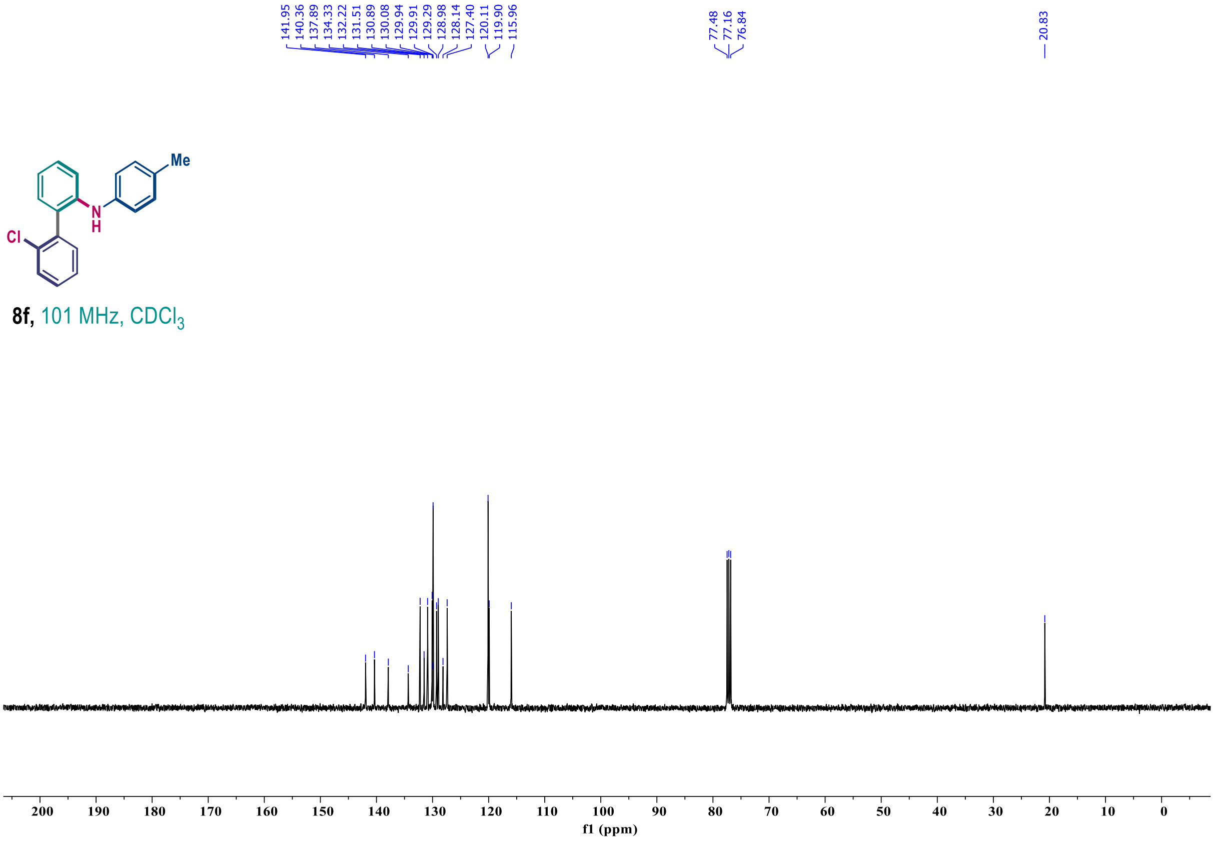
141.95
140.36
137.89
134.33
132.22
131.51
130.89
130.08
129.94
129.91
129.29
128.98
128.14
127.40
120.11
119.90
115.96
77.48
77.16
76.84
20.83
Me
N
H
Cl
8f, 101 MHz, CDCl3
f1 (ppm)

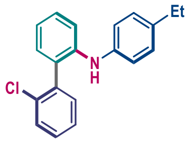

8g, 400 MHz, CDCl$_3$

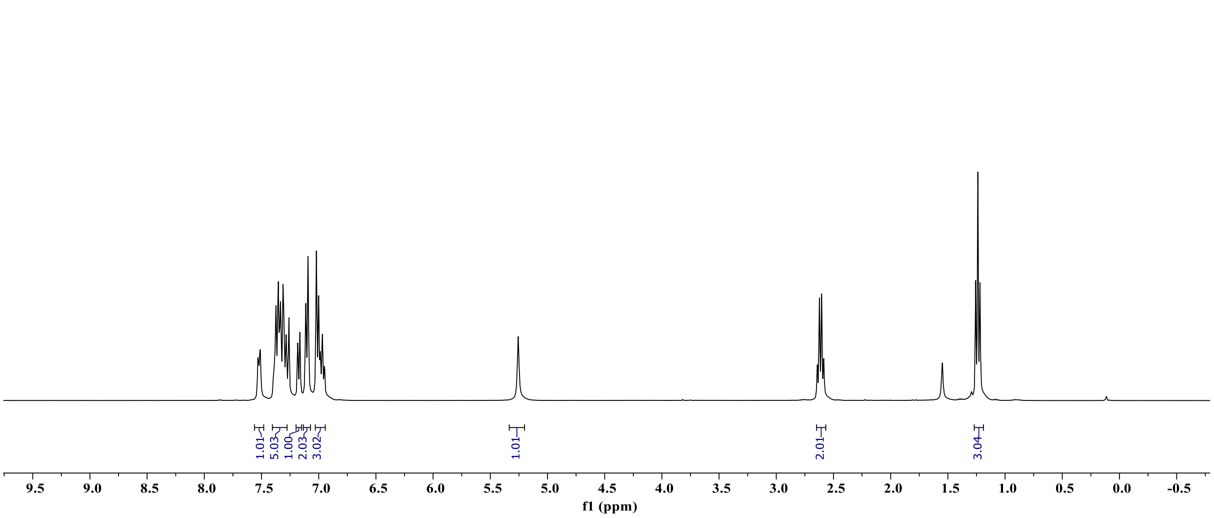

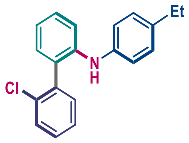

8g, 101 MHz, CDCl$_3$

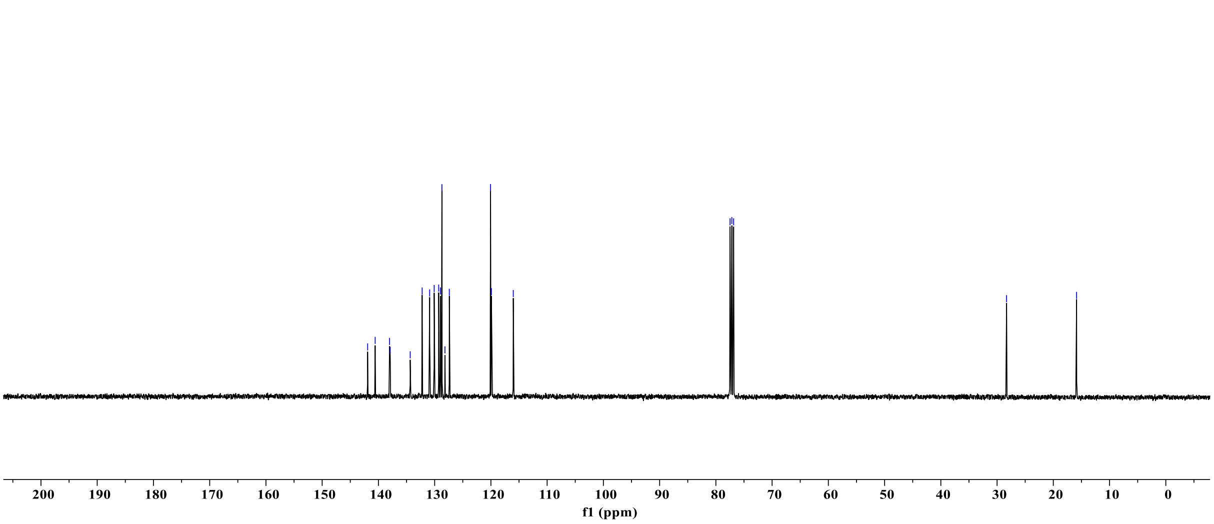

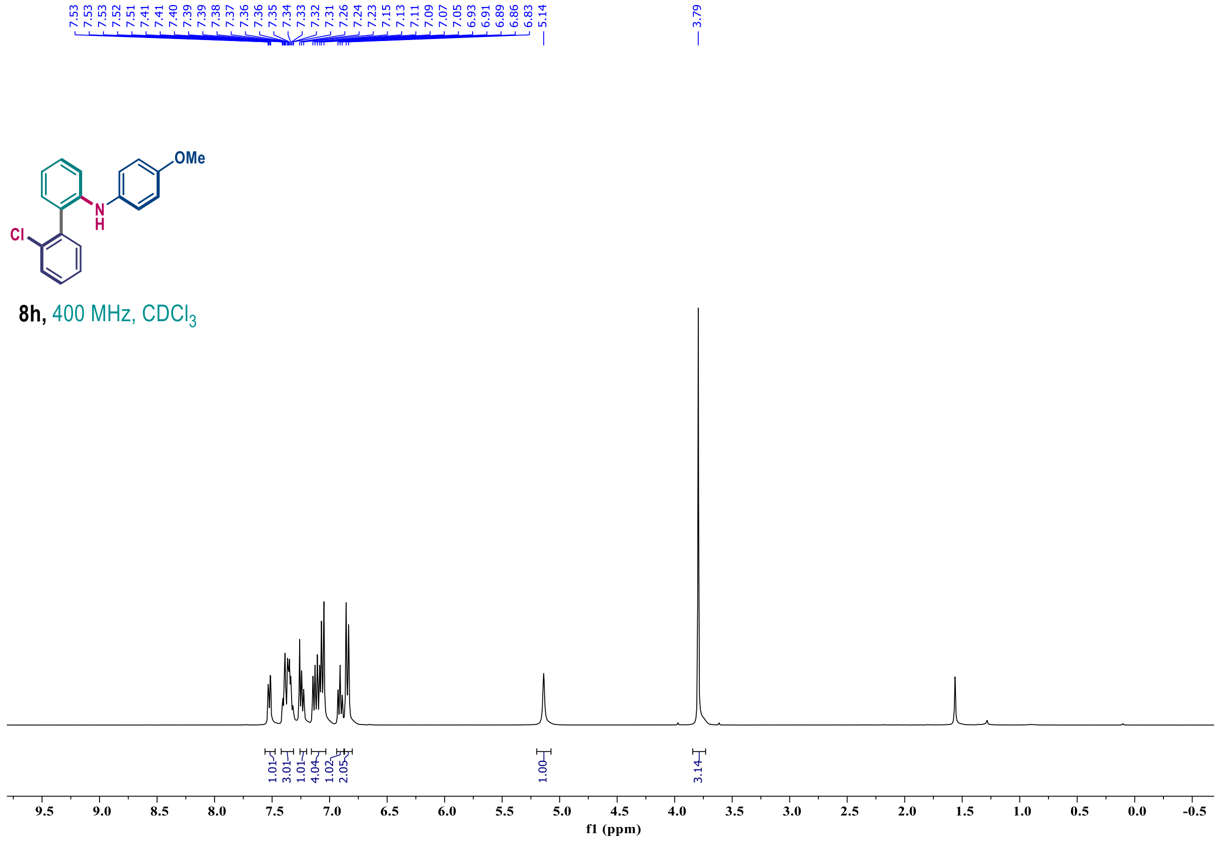
7.53
7.53
7.53
7.52
7.51
7.41
7.41
7.40
7.39
7.39
7.38
7.37
7.36
7.35
7.34
7.33
7.32
7.31
7.26
7.24
7.23
7.15
7.13
7.11
7.09
7.07
7.05
6.93
6.91
6.89
6.86
6.83
5.14
3.79
OMe
N
H
Cl
8h, 400 MHz, CDCl3
1.01
3.01
1.01
4.04
1.02
2.05
1.00
3.14
f1 (ppm)

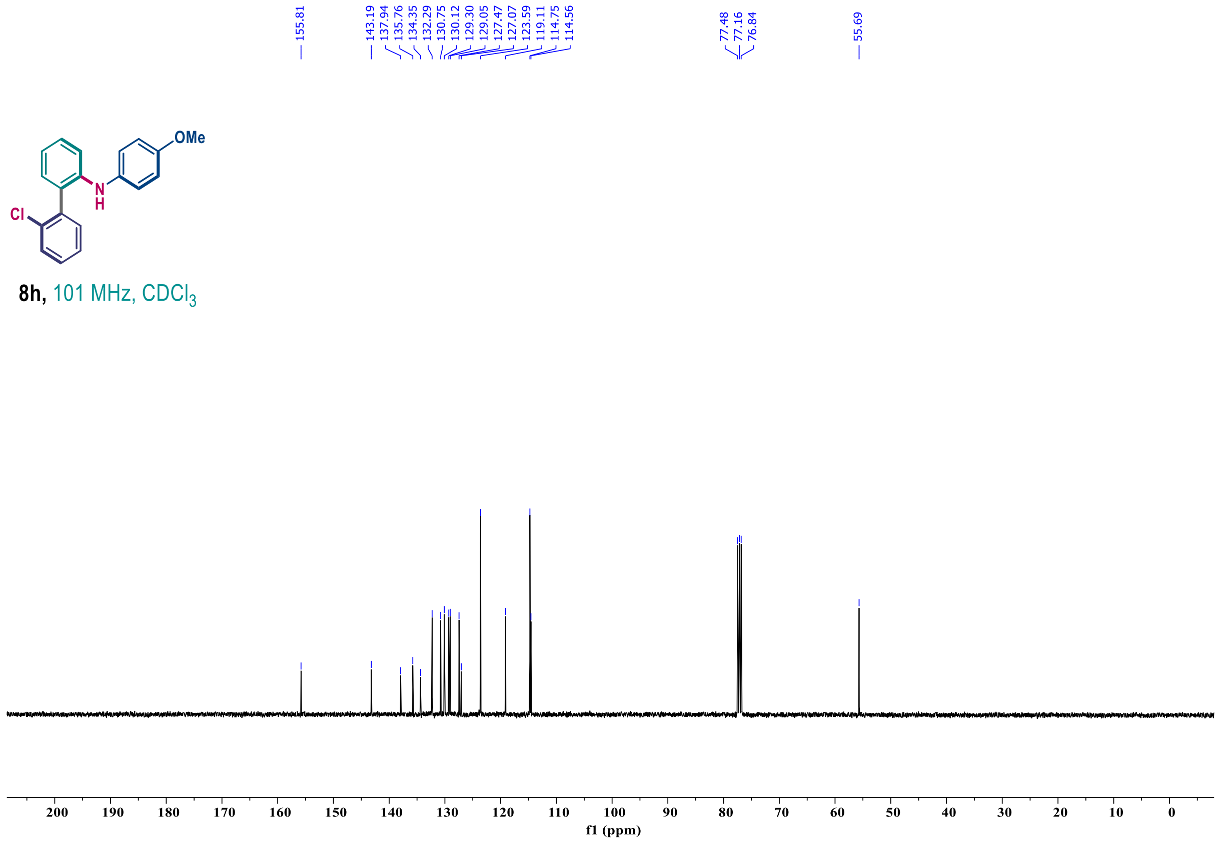
155.81
143.19
137.94
135.76
134.35
132.29
130.75
130.12
129.30
129.05
127.47
127.07
123.59
119.11
114.75
114.56
77.48
77.16
76.84
55.69
OMe
N
H
Cl
8h, 101 MHz, CDCl3
f1 (ppm)

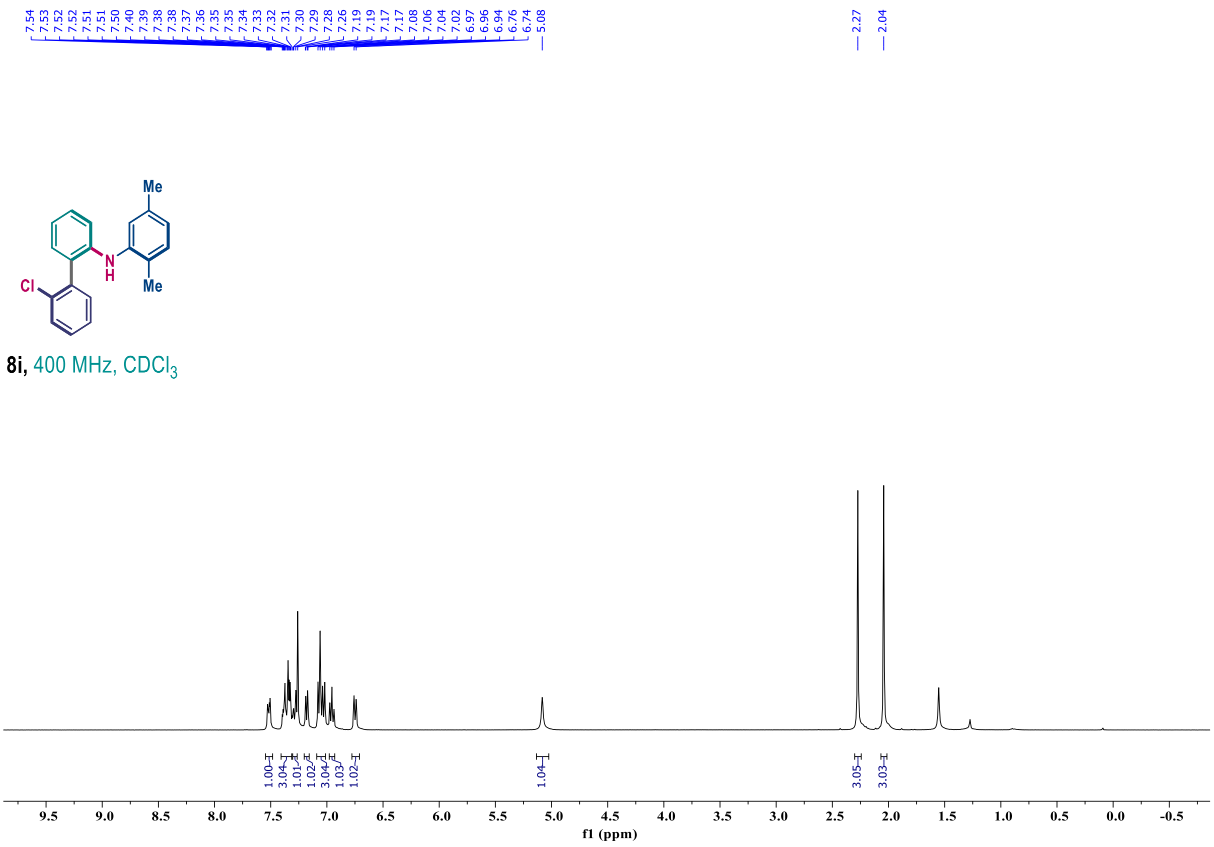
7.54 7.53 7.52 7.52 7.51 7.51 7.50 7.40 7.39 7.38 7.38 7.37 7.36 7.35 7.35 7.34 7.33 7.32 7.31 7.30 7.29 7.28 7.26 7.19 7.19 7.17 7.17 7.08 7.06 7.04 7.02 6.97 6.96 6.94 6.76 6.74 5.08
2.27 2.04
Me
N
H
Me
Cl
8i, 400 MHz, CDCl3
1.00 3.04 1.01 1.02 3.04 1.03 1.02 1.04 3.05 3.03
9.5 9.0 8.5 8.0 7.5 7.0 6.5 6.0 5.5 5.0 4.5 4.0 3.5 3.0 2.5 2.0 1.5 1.0 0.5 0.0 -0.5
f1 (ppm)

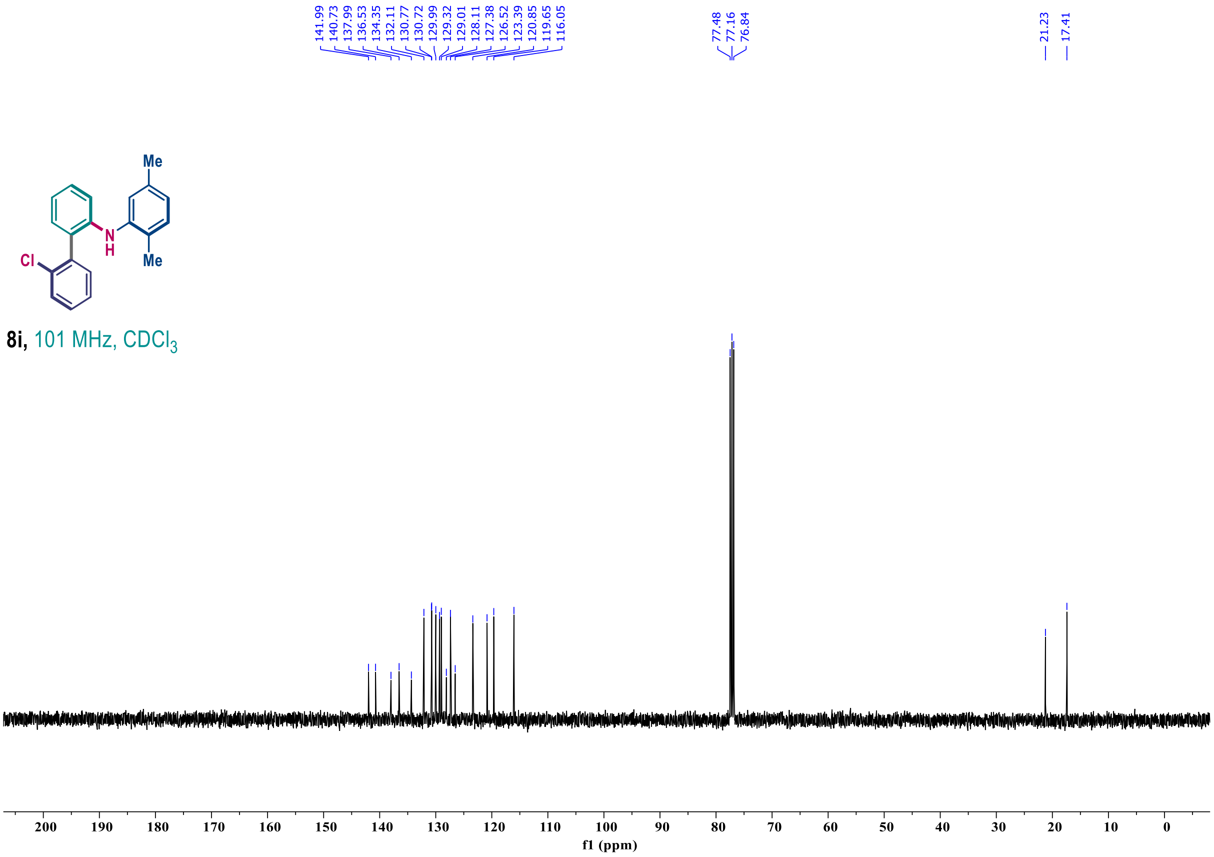
141.99 140.73 137.99 136.53 134.35 132.11 130.77 130.72 129.99 129.32 129.01 128.11 127.38 126.52 123.39 120.85 119.65 116.05
77.48 77.16 76.84
21.23 17.41
Me
N
H
Me
Cl
8i, 101 MHz, CDCl3
200 190 180 170 160 150 140 130 120 110 100 90 80 70 60 50 40 30 20 10 0
f1 (ppm)

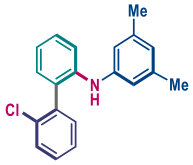

8j, 400 MHz, $CDCl_3$

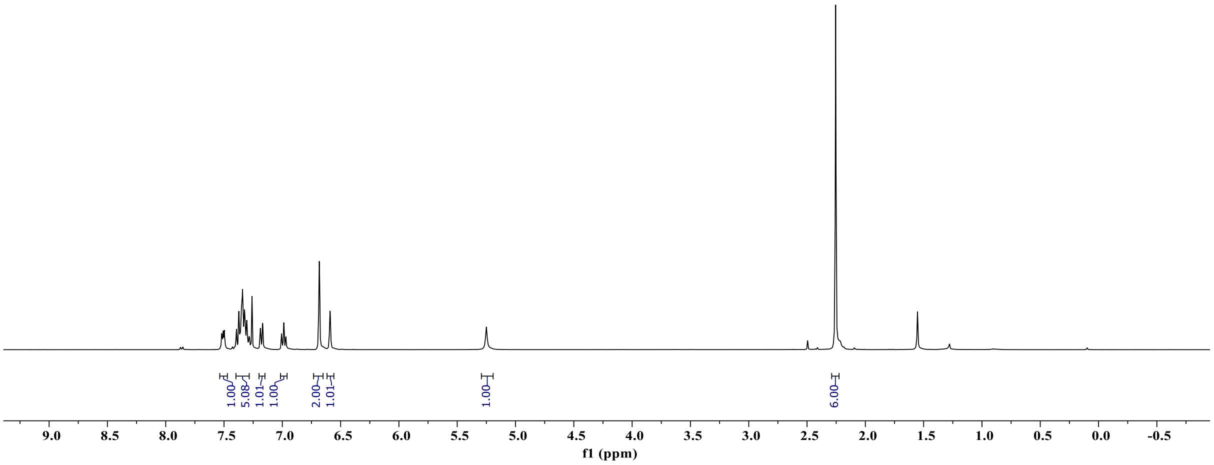

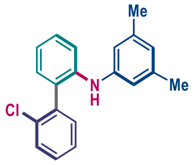

8j, 101 MHz, $CDCl_3$

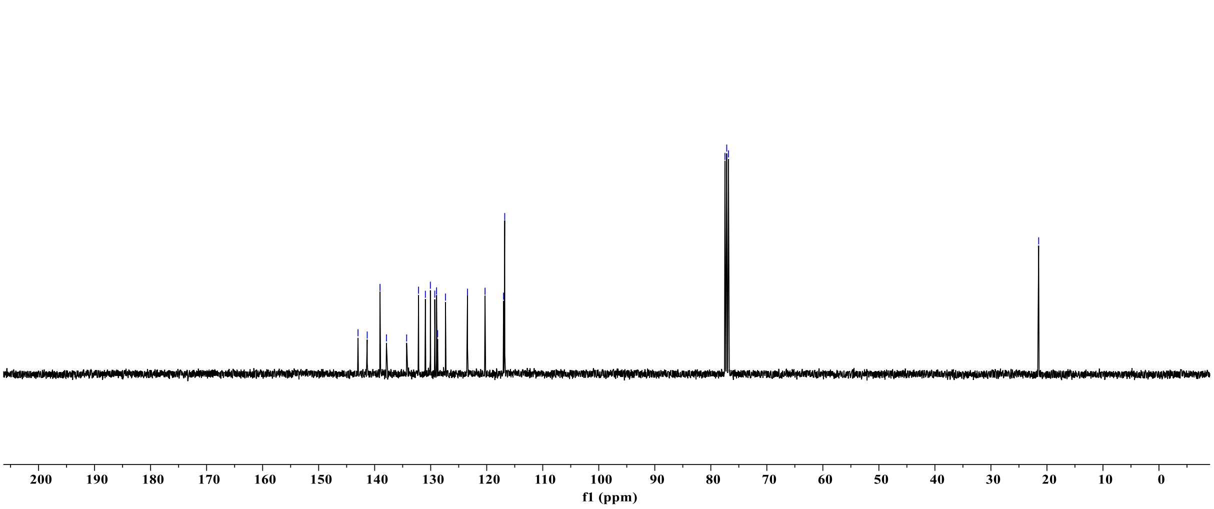

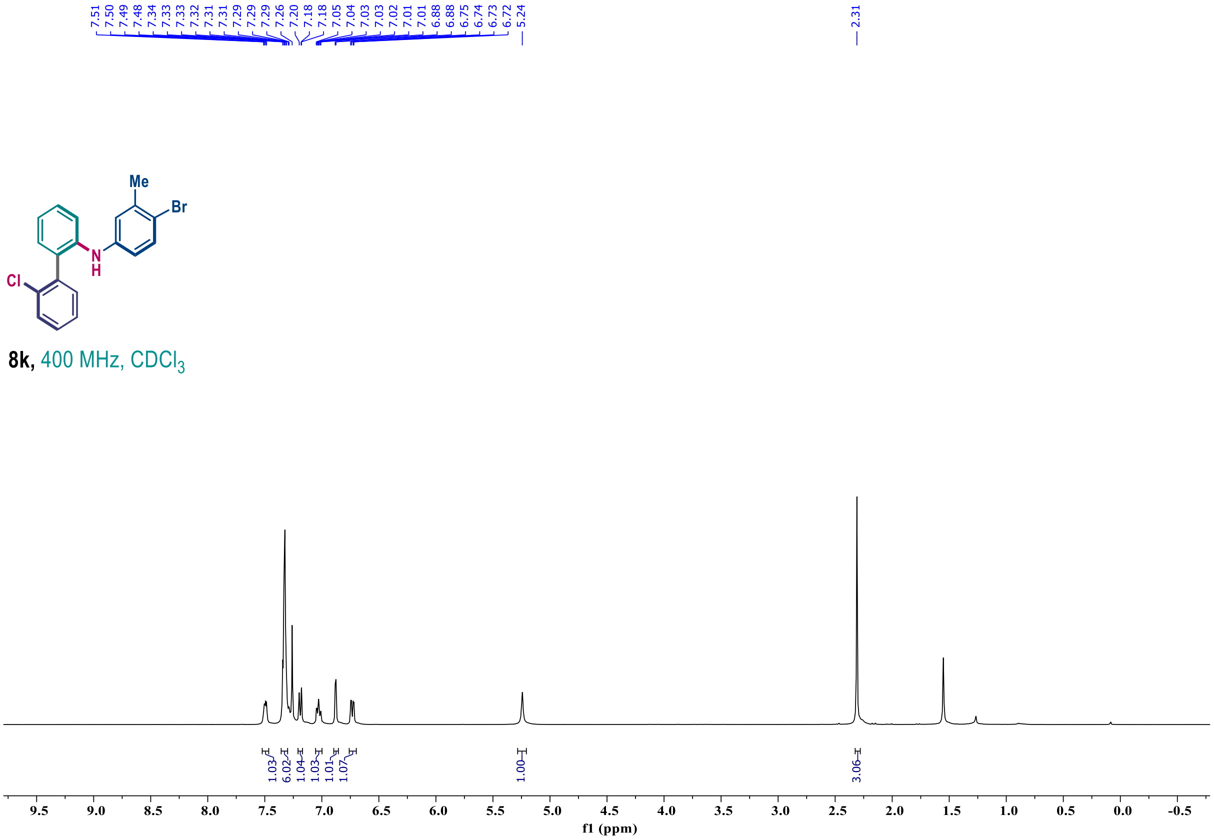

8k, 400 MHz, $CDCl_3$

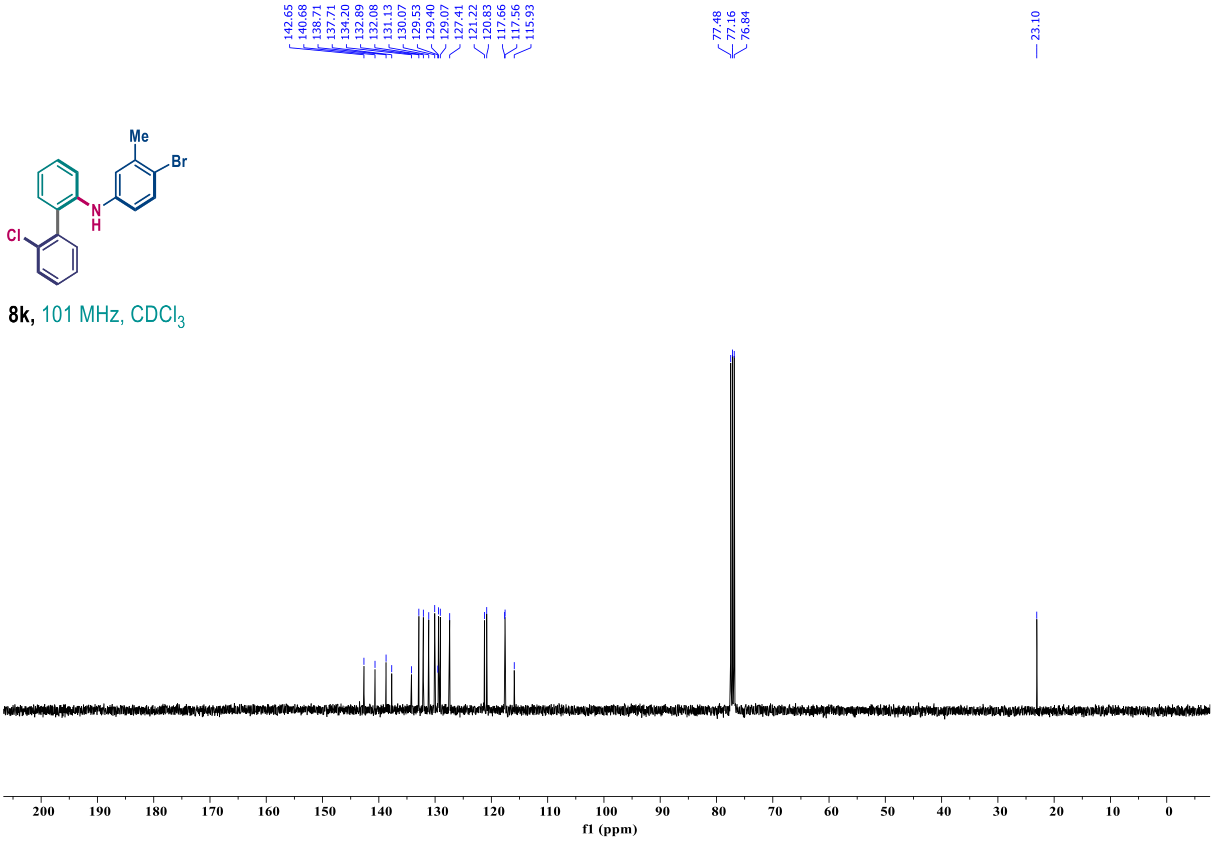

8k, 101 MHz, $CDCl_3$

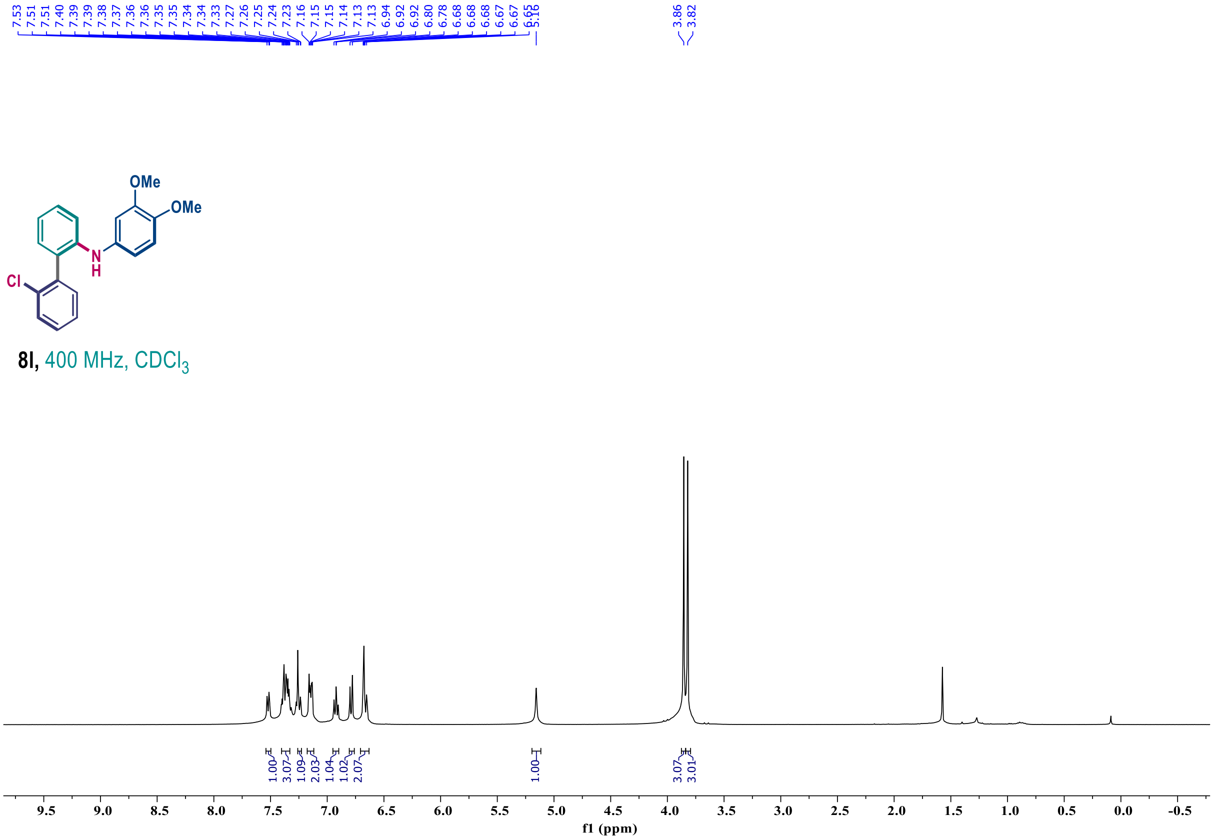
7.53 7.51 7.51 7.40 7.39 7.39 7.38 7.37 7.36 7.36 7.35 7.35 7.34 7.34 7.33 7.27 7.26 7.25 7.24 7.23 7.16 7.15 7.15 7.14 7.13 7.13 6.94 6.92 6.92 6.80 6.78 6.68 6.68 6.68 6.67 6.67 6.65 5.16
3.86 3.82
OMe
OMe
N
H
Cl
8l, 400 MHz, CDCl3
1.00 3.07 1.09 2.03 1.04 1.02 2.07
1.00
3.07 3.01
9.5 9.0 8.5 8.0 7.5 7.0 6.5 6.0 5.5 5.0 4.5 4.0 3.5 3.0 2.5 2.0 1.5 1.0 0.5 0.0 -0.5
f1 (ppm)

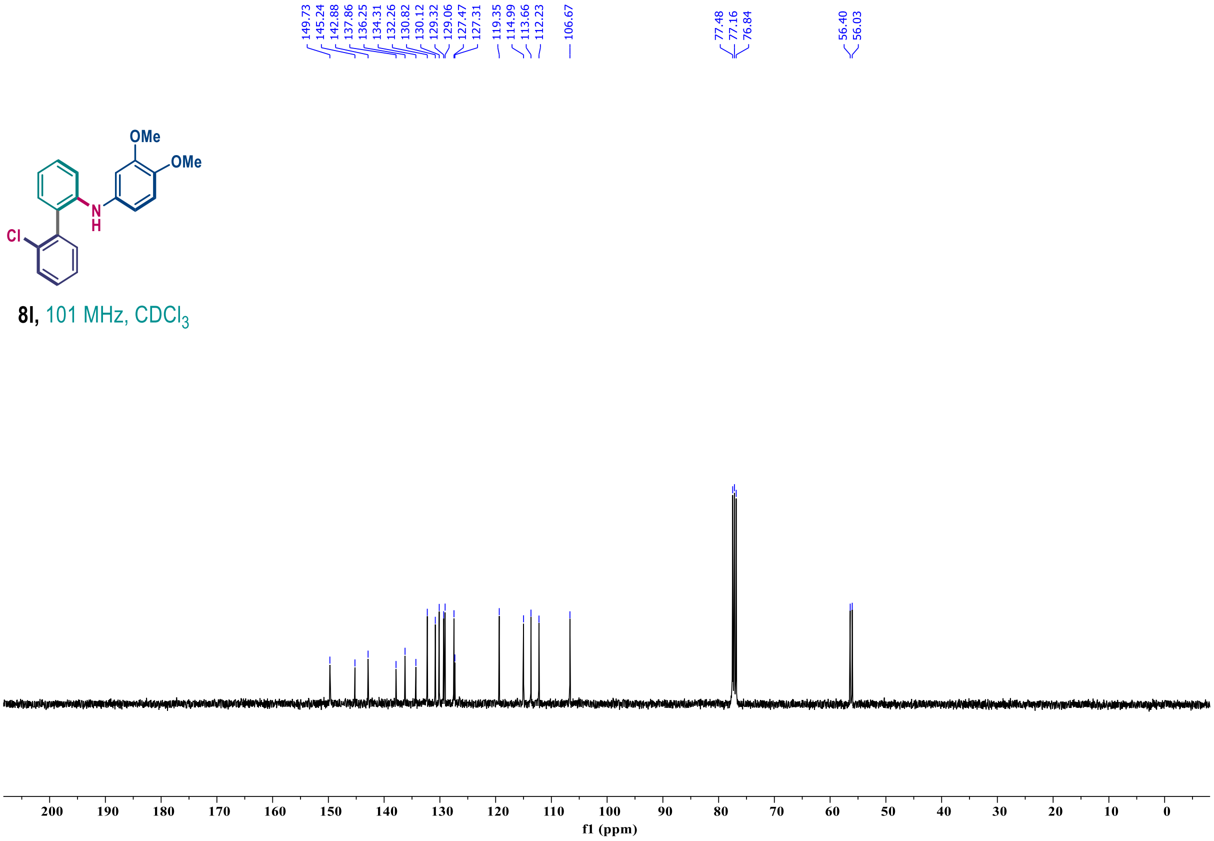
149.73 145.24 142.88 137.86 136.25 134.31 132.26 130.82 130.12 129.32 129.06 127.47 127.31 119.35 114.99 113.66 112.23 106.67
77.48 77.16 76.84
56.40 56.03
OMe
OMe
N
H
Cl
8l, 101 MHz, CDCl3
200 190 180 170 160 150 140 130 120 110 100 90 80 70 60 50 40 30 20 10 0
f1 (ppm)

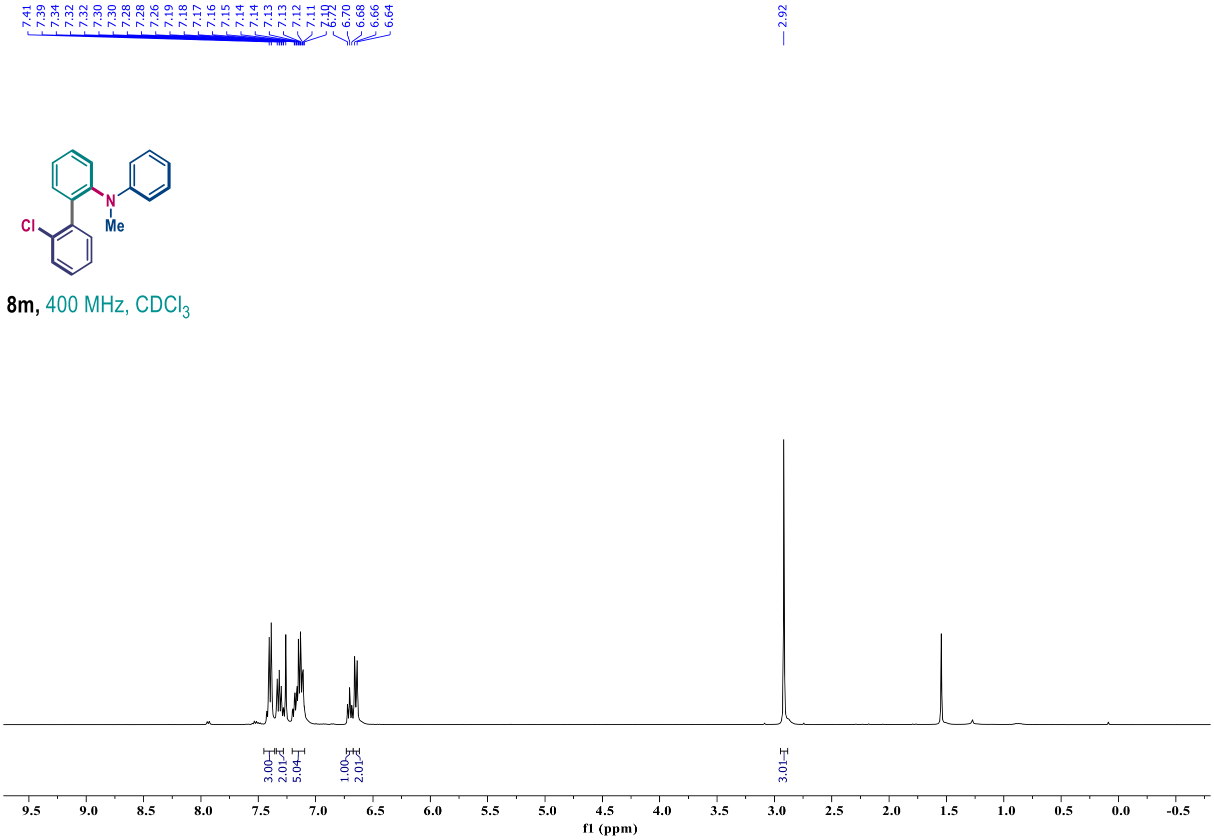

8m, 400 MHz, $CDCl_3$

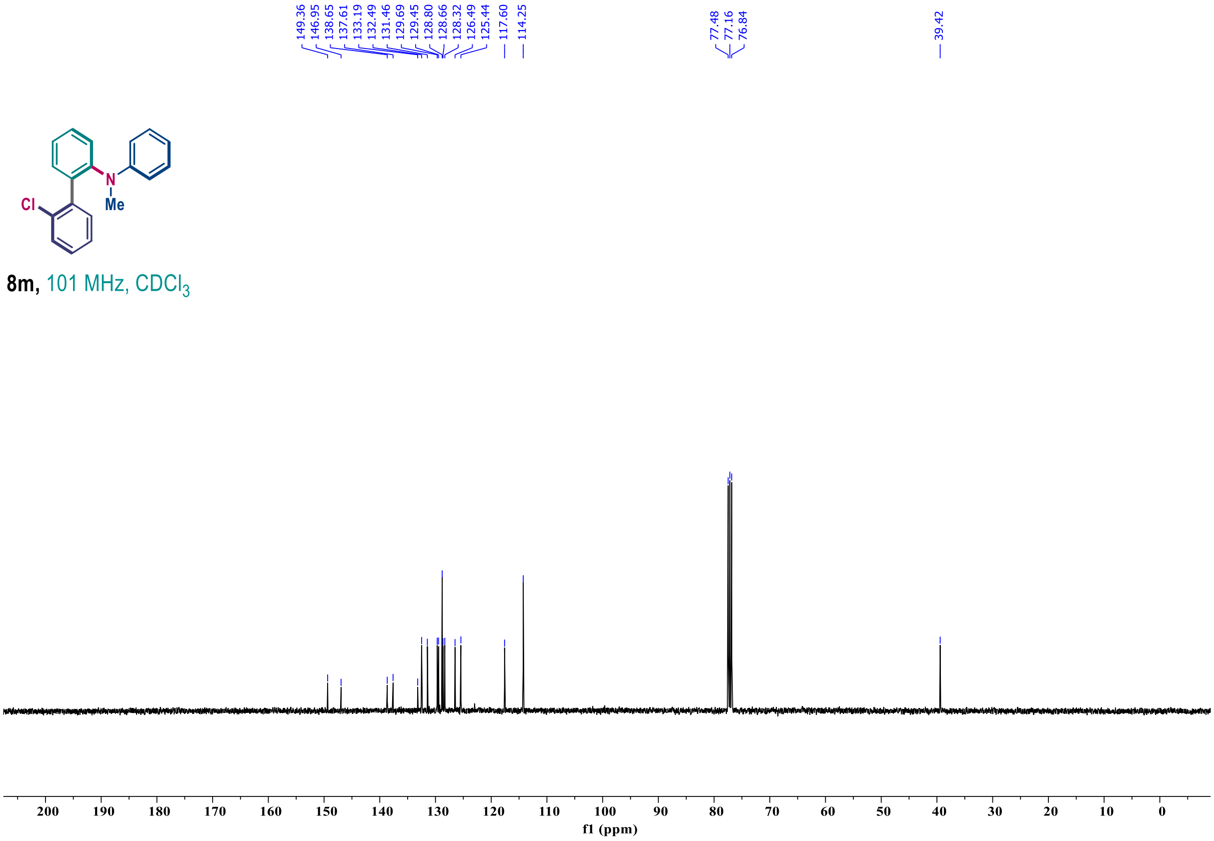

8m, 101 MHz, $CDCl_3$

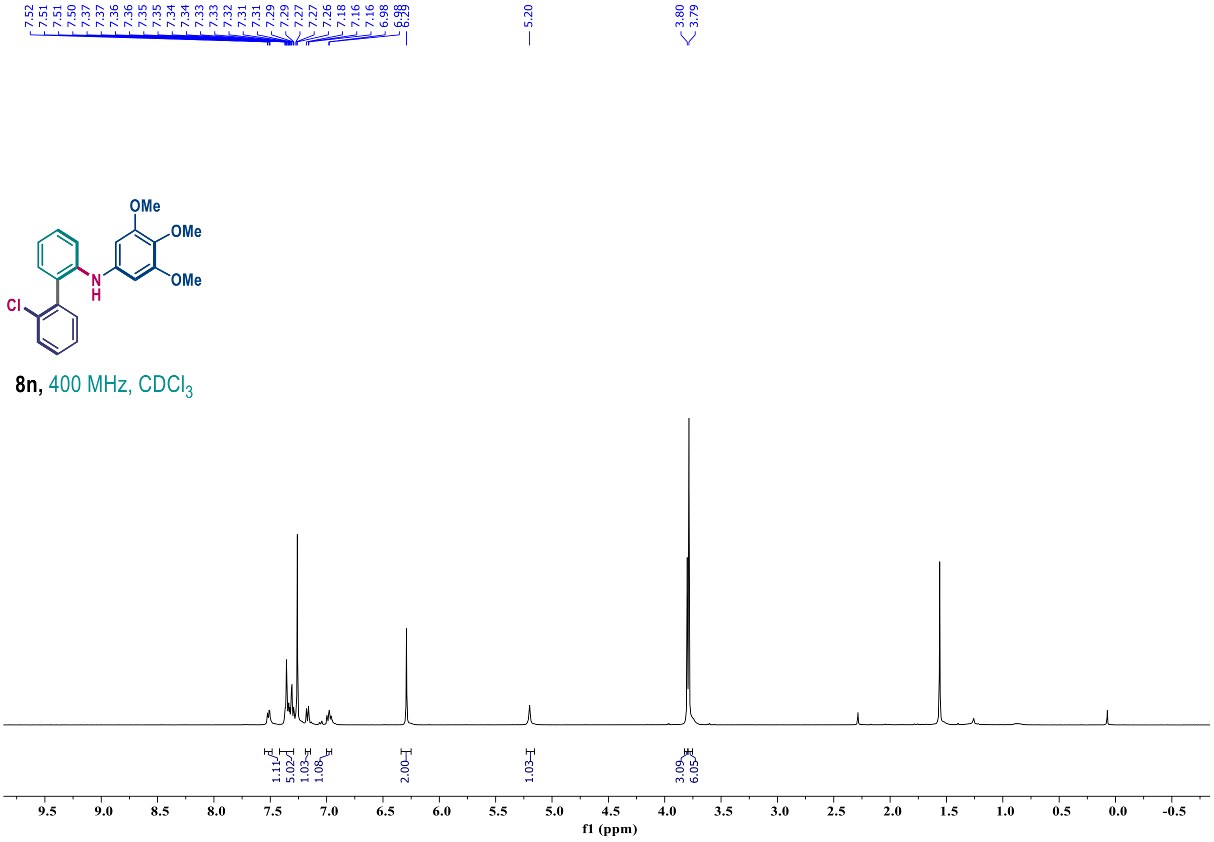

8n, 400 MHz, $CDCl_3$

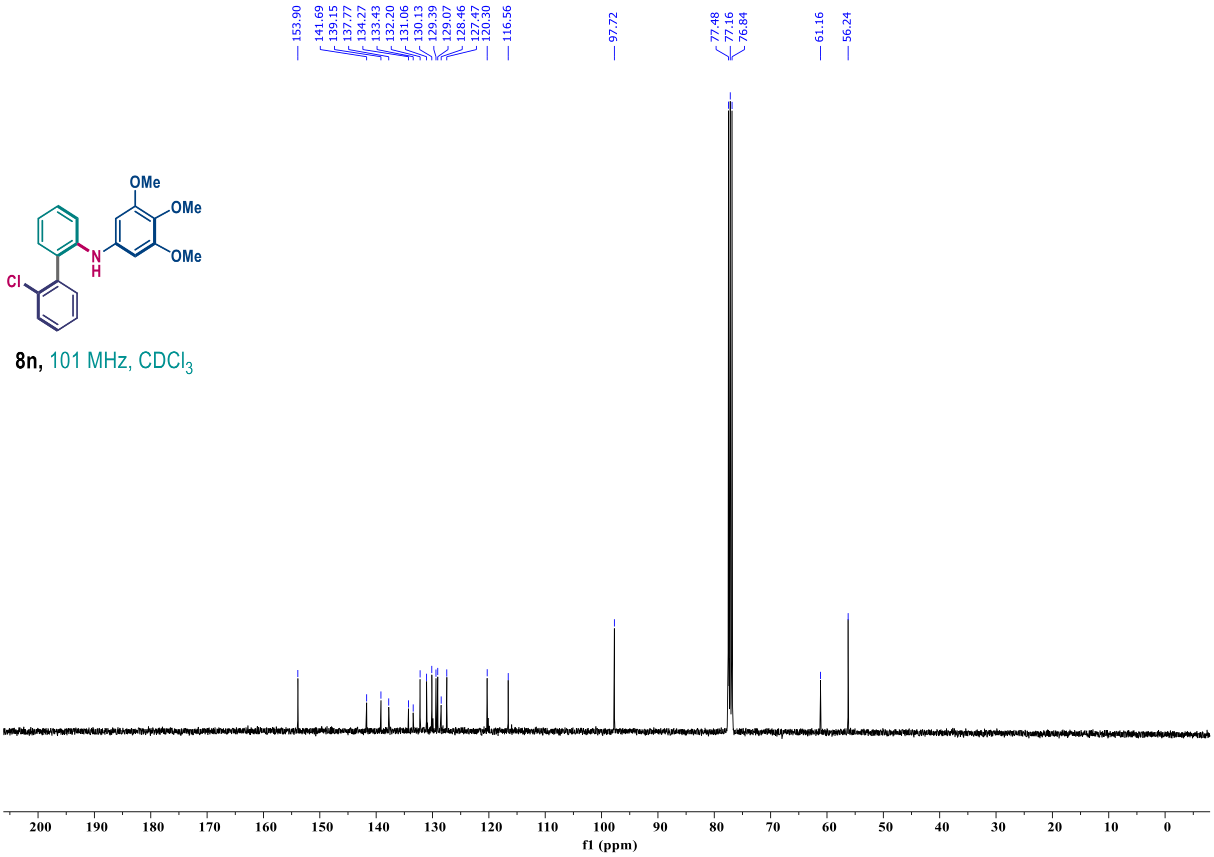

8n, 101 MHz, $CDCl_3$

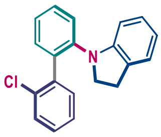

8o, 400 MHz, $CDCl_3$

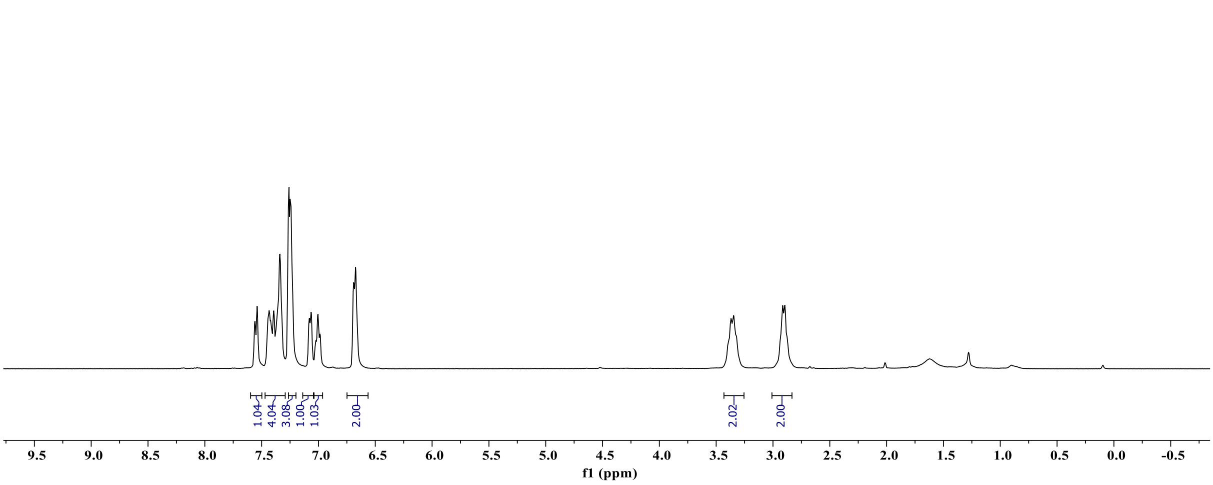

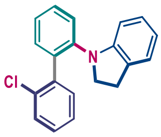

8o, 101 MHz, $CDCl_3$

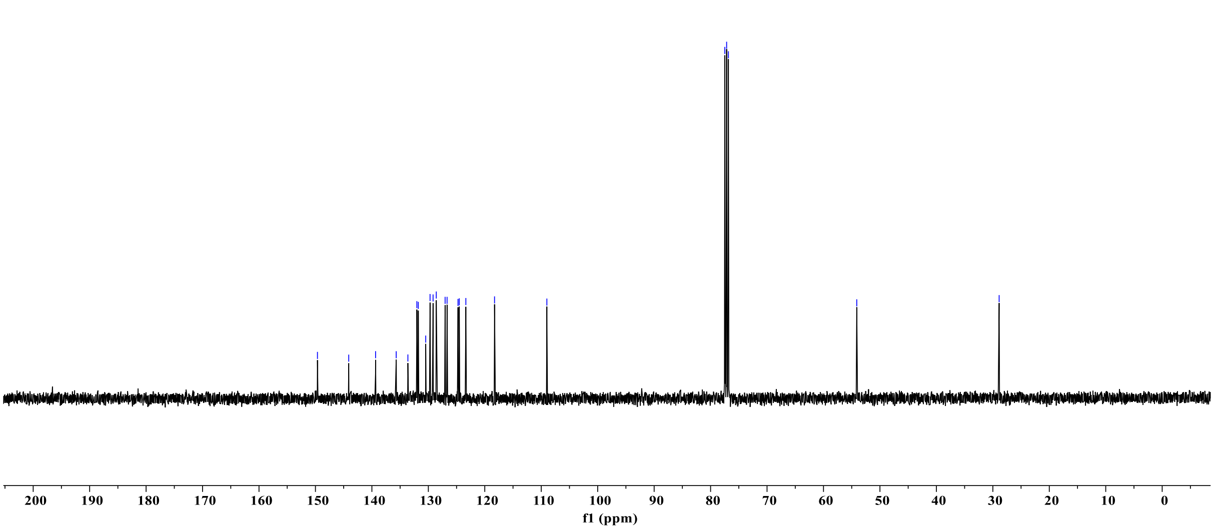

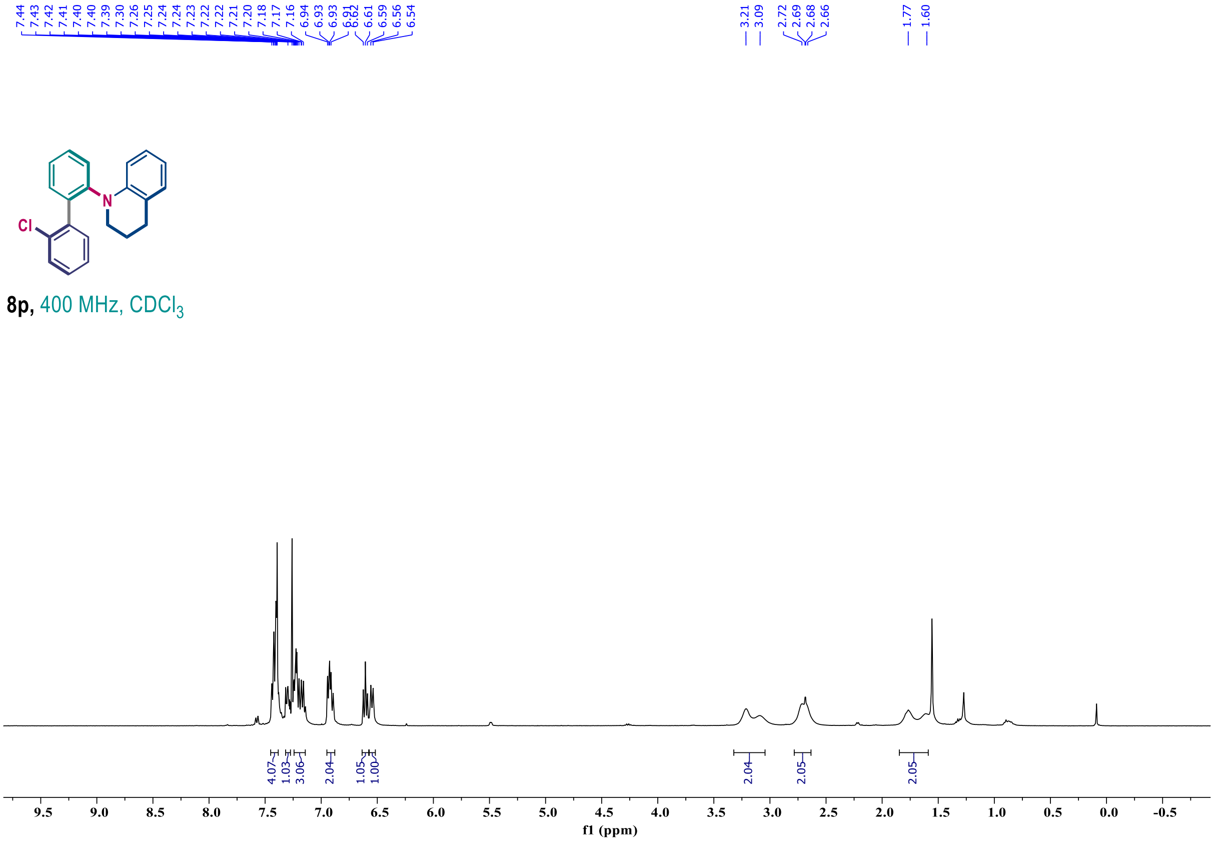
7.44
7.43
7.42
7.41
7.40
7.40
7.39
7.30
7.26
7.25
7.24
7.24
7.23
7.22
7.22
7.21
7.20
7.18
7.17
7.16
6.94
6.93
6.93
6.91
6.62
6.61
6.59
6.56
6.54
3.21
3.09
2.72
2.69
2.68
2.66
1.77
1.60
Cl
N
8p, 400 MHz, $CDCl_3$
4.07
1.03
3.06
2.04
1.05
1.00
2.04
2.05
2.05
f1 (ppm)

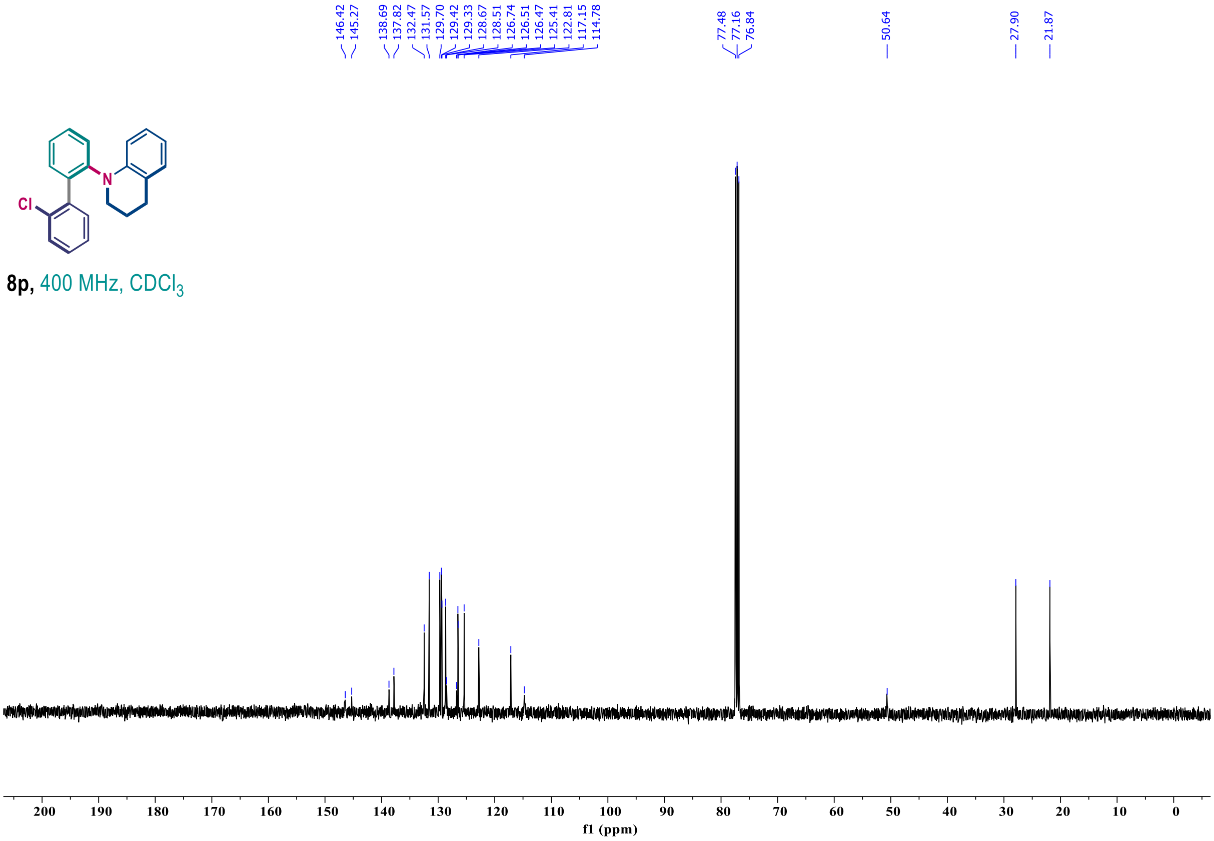
146.42
145.27
138.69
137.82
132.47
131.57
129.70
129.42
129.33
128.67
128.51
126.74
126.51
126.47
125.41
122.81
117.15
114.78
77.48
77.16
76.84
50.64
27.90
21.87
Cl
N
8p, 400 MHz, $CDCl_3$
f1 (ppm)

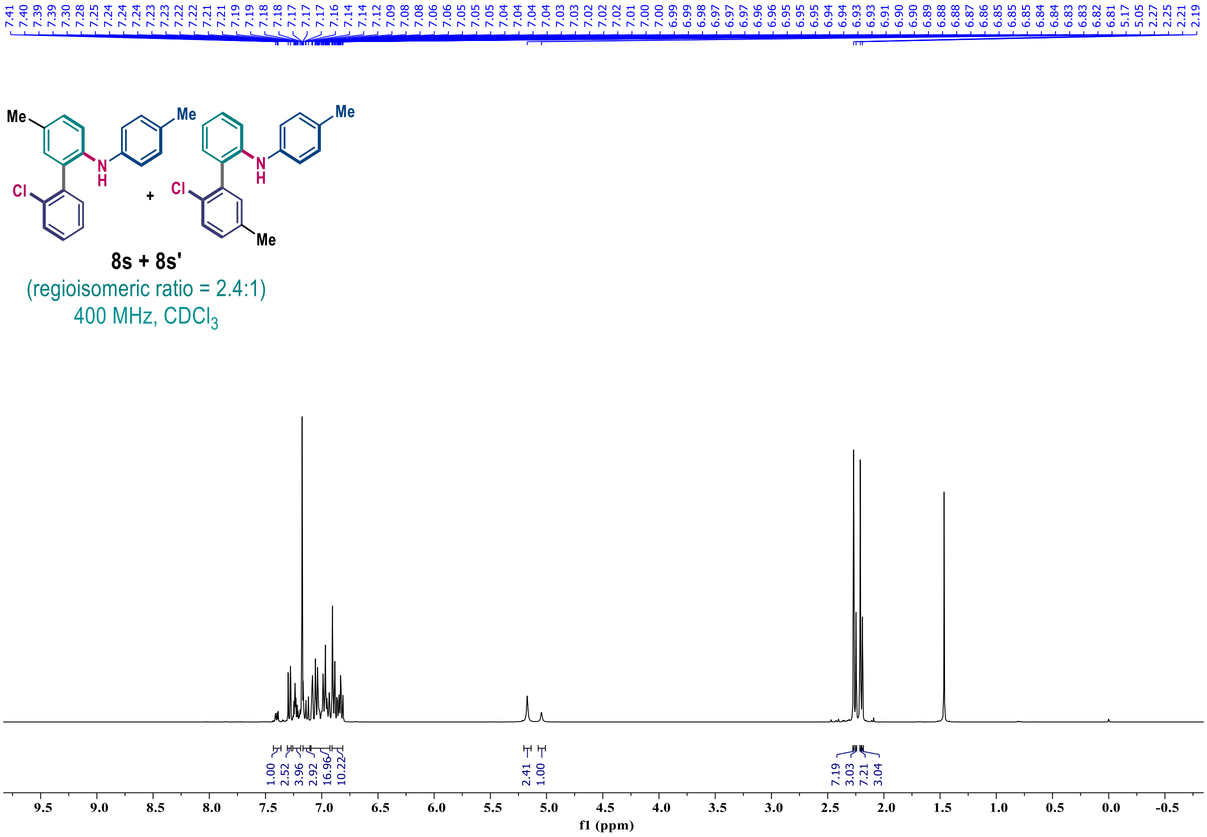

Me
Me
N
H
Cl
+
Me
N
H
Cl
Me
8s + 8s'
(regioisomeric ratio = 2.4:1)
400 MHz, $CDCl_3$
f1 (ppm)

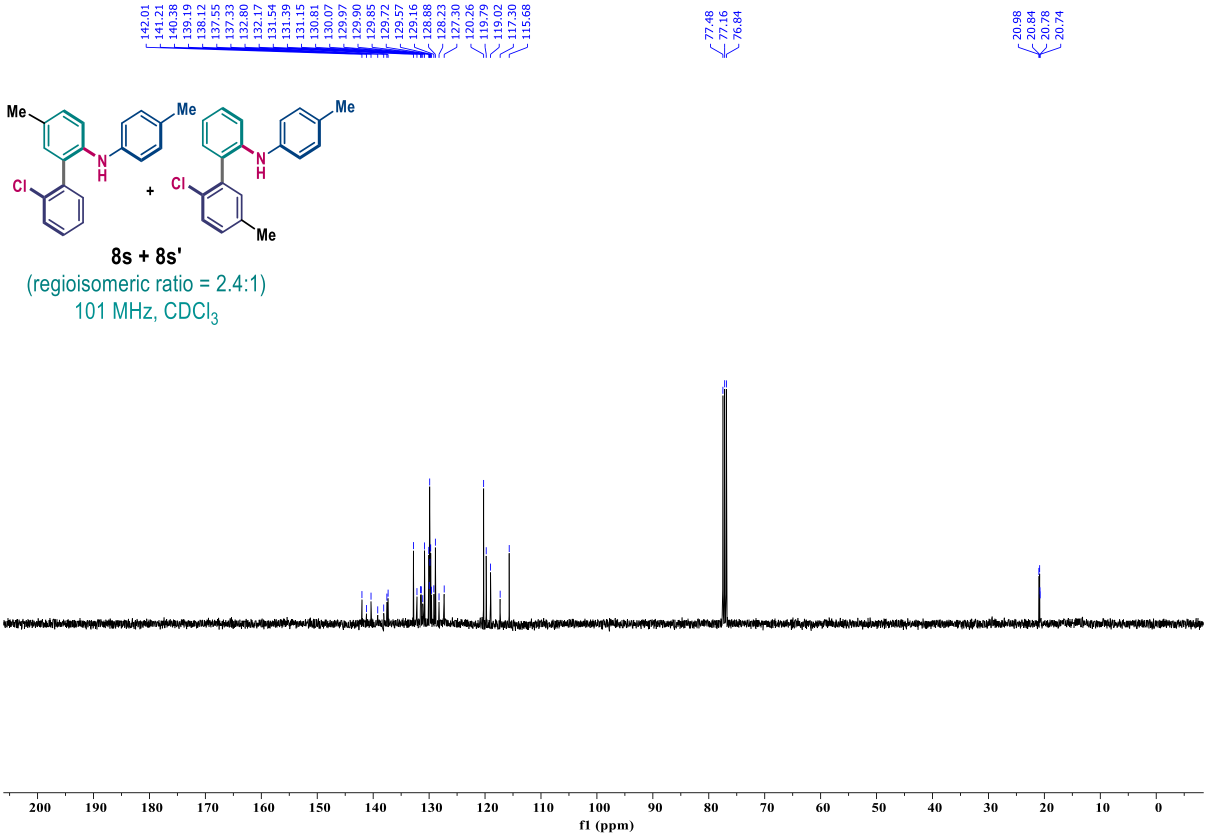

Me
Me
N
H
Cl
+
Me
N
H
Cl
Me
8s + 8s'
(regioisomeric ratio = 2.4:1)
101 MHz, $CDCl_3$
f1 (ppm)

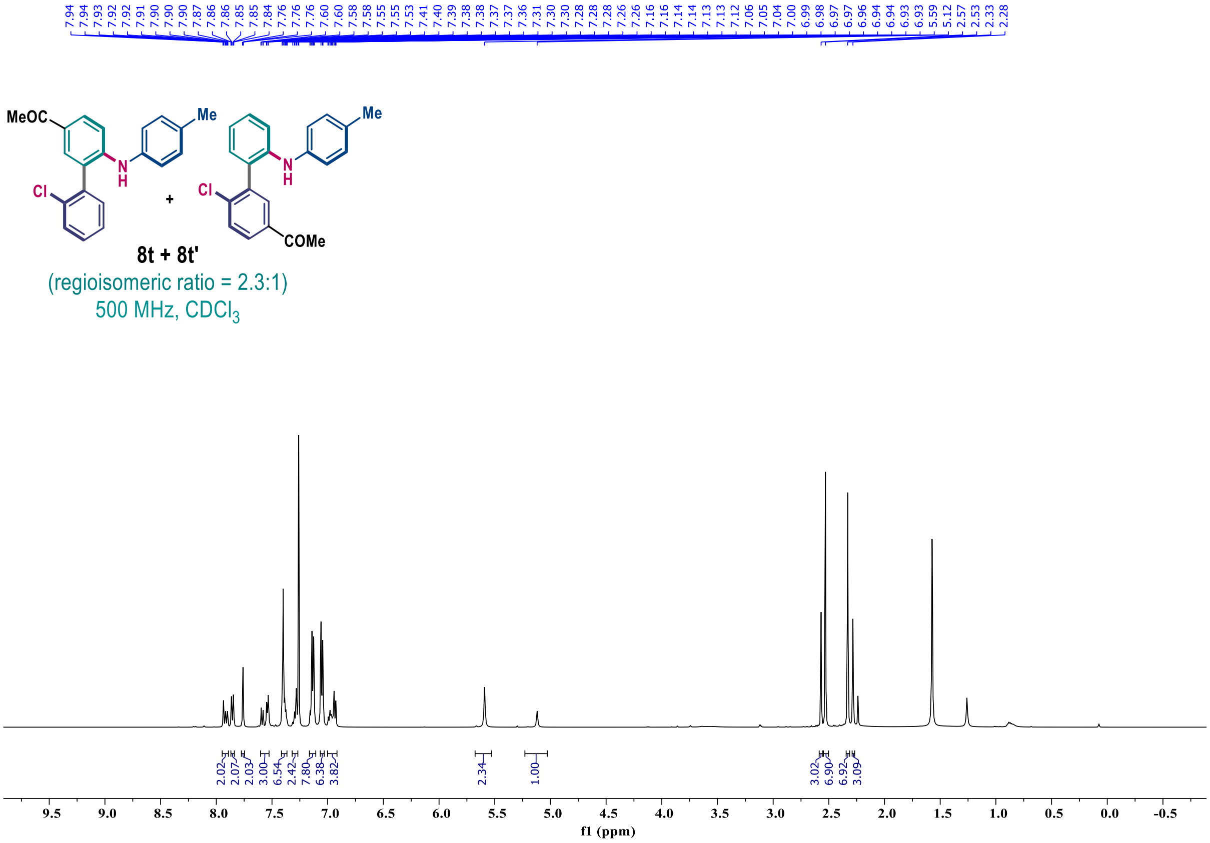
7.94 7.94 7.93 7.92 7.92 7.91 7.90 7.90 7.90 7.87 7.86 7.86 7.85 7.85 7.84 7.76 7.76 7.76 7.76 7.60 7.60 7.58 7.58 7.55 7.55 7.53 7.41 7.40 7.39 7.38 7.38 7.37 7.37 7.36 7.31 7.30 7.30 7.28 7.28 7.28 7.26 7.26 7.16 7.16 7.14 7.14 7.13 7.13 7.12 7.06 7.05 7.04 7.00 6.99 6.98 6.97 6.97 6.96 6.94 6.94 6.93 6.93 5.59 5.12 2.57 2.53 2.33 2.28
MeOC
Me
N
H
Cl
+
COMe
8t + 8t'
(regioisomeric ratio = 2.3:1)
500 MHz, CDCl$_3$
2.02 2.07 2.03 3.00 6.54 2.42 7.80 6.38 3.82 2.34 1.00 3.02 6.90 6.92 3.09
f1 (ppm)

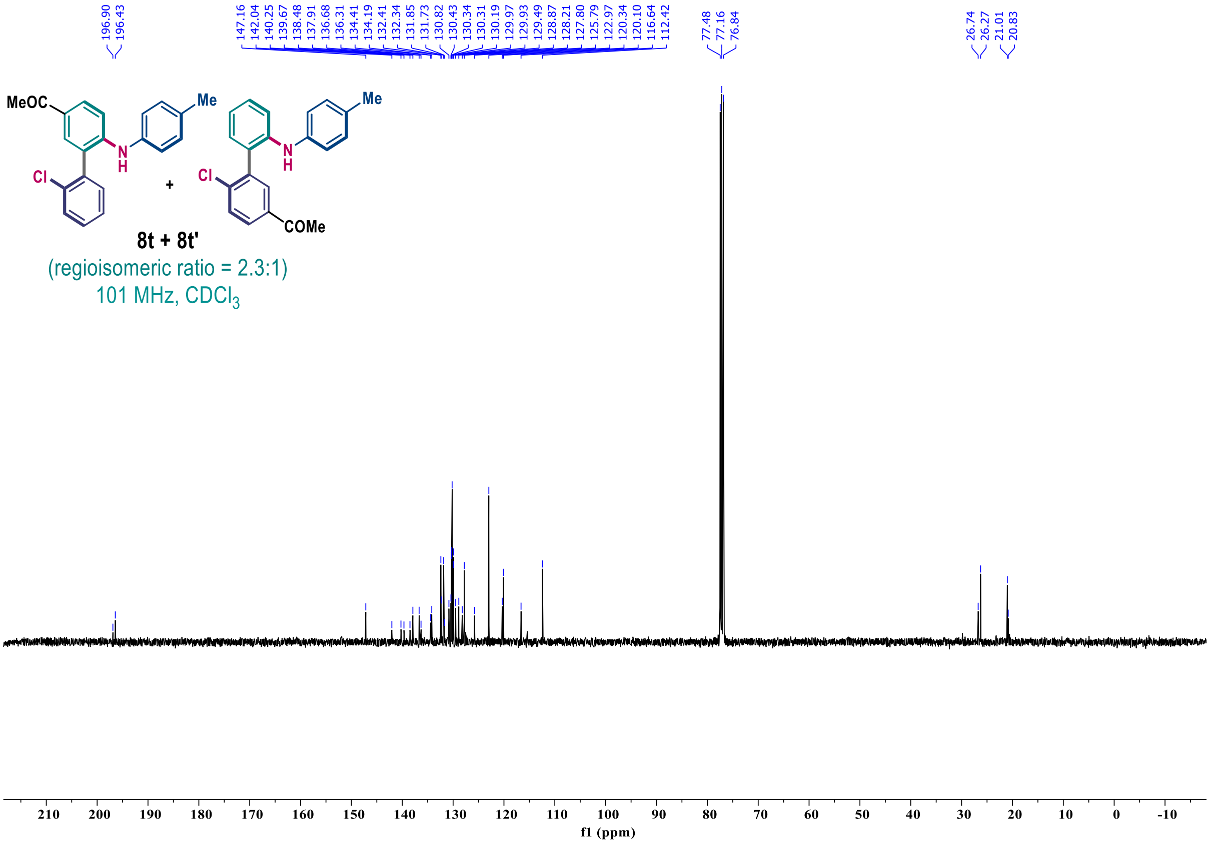
196.90 196.43
147.16 142.04 140.25 139.67 138.48 137.91 136.68 136.31 134.41 134.19 132.41 132.34 131.85 131.73 130.82 130.43 130.34 130.31 130.19 129.97 129.93 129.49 128.87 128.21 127.80 125.79 122.97 120.34 120.10 116.64 112.42
77.48 77.16 76.84
26.74 26.27 21.01 20.83
MeOC
Me
N
H
Cl
+
COMe
8t + 8t'
(regioisomeric ratio = 2.3:1)
101 MHz, CDCl$_3$
f1 (ppm)

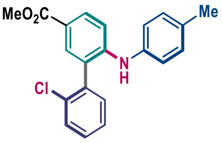

8u
(major isomer)
500 MHz, $CDCl_3$

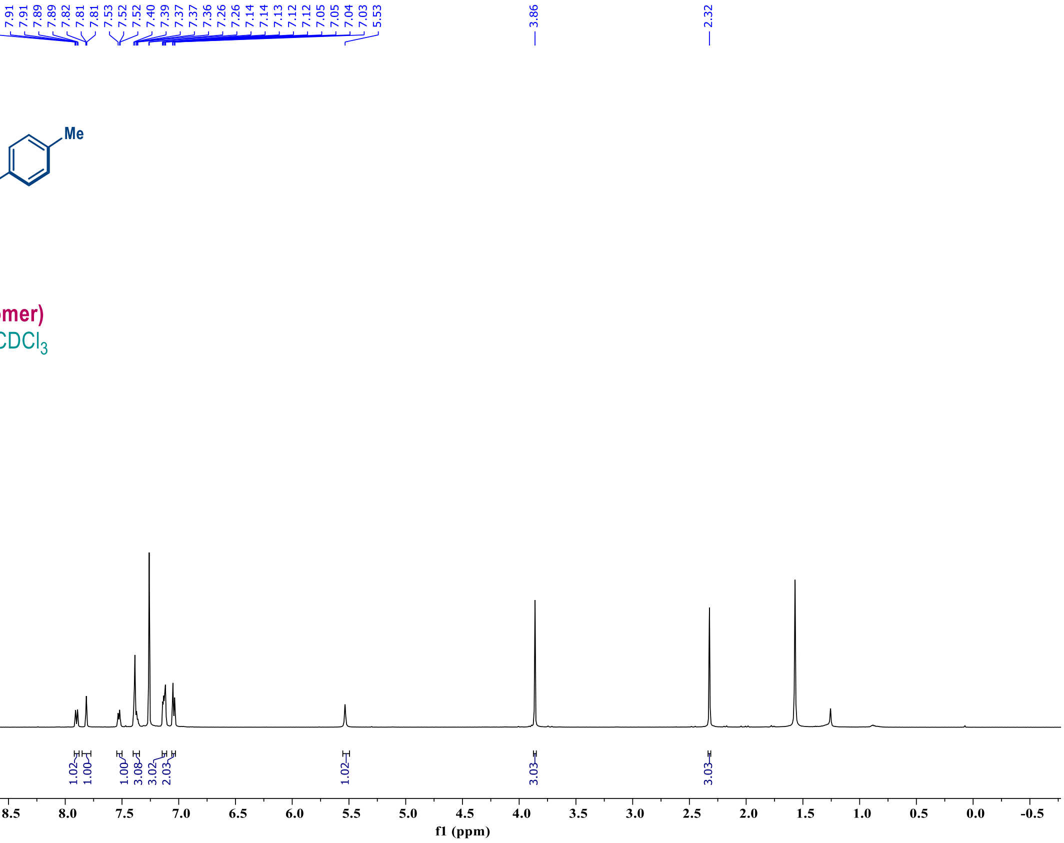

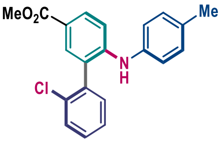

8u
(major isomer)
126 MHz, $CDCl_3$

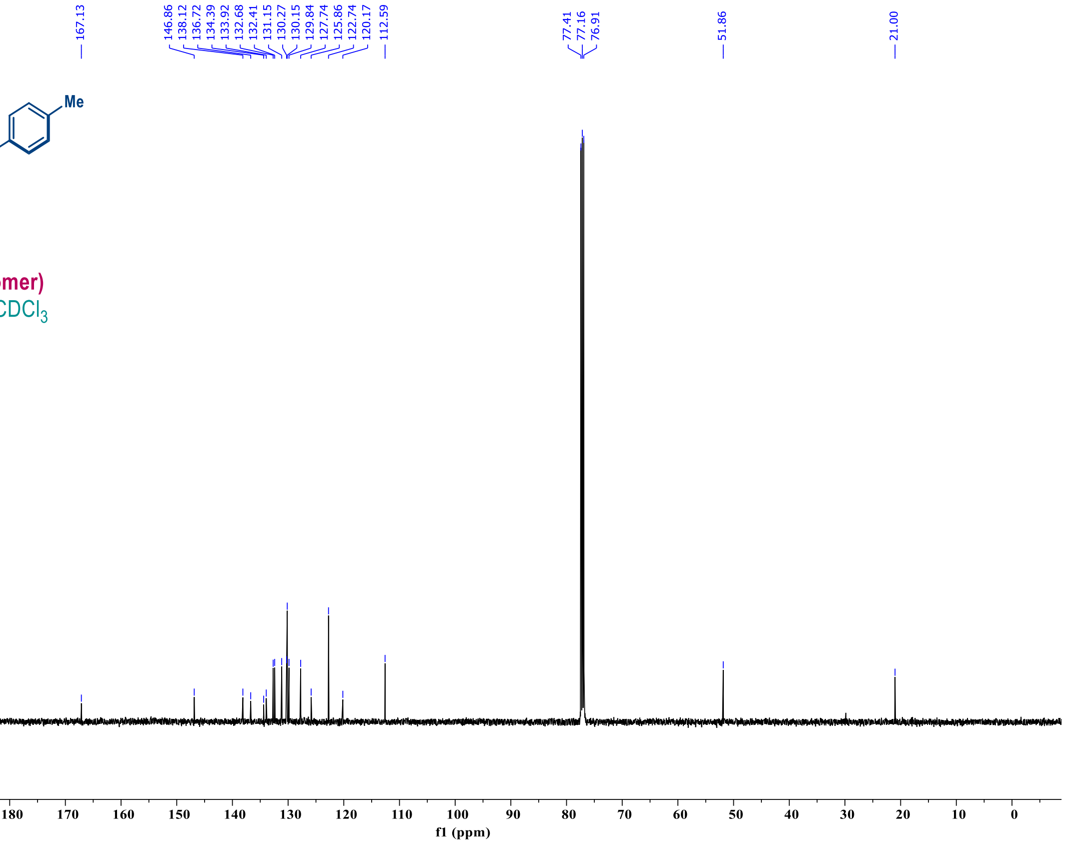

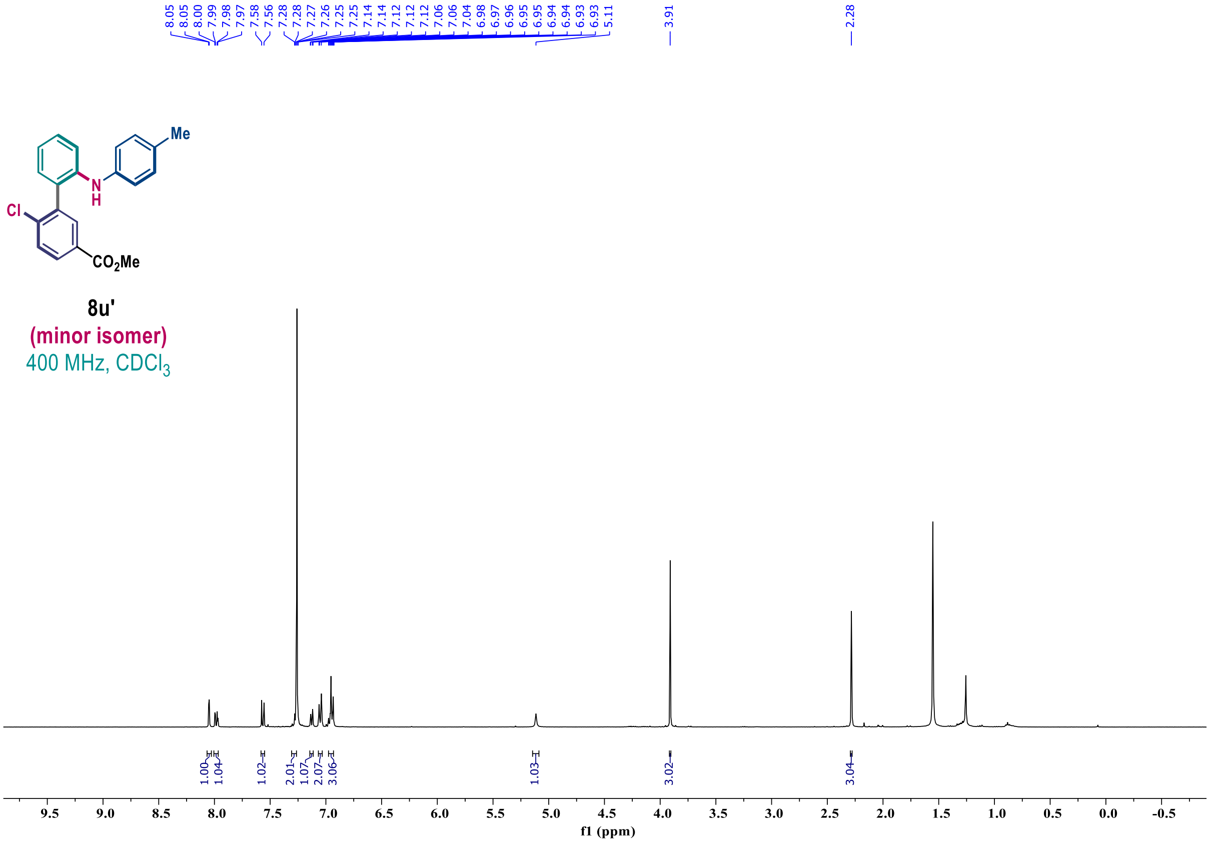

8u'

(minor isomer)

400 MHz, $CDCl_3$

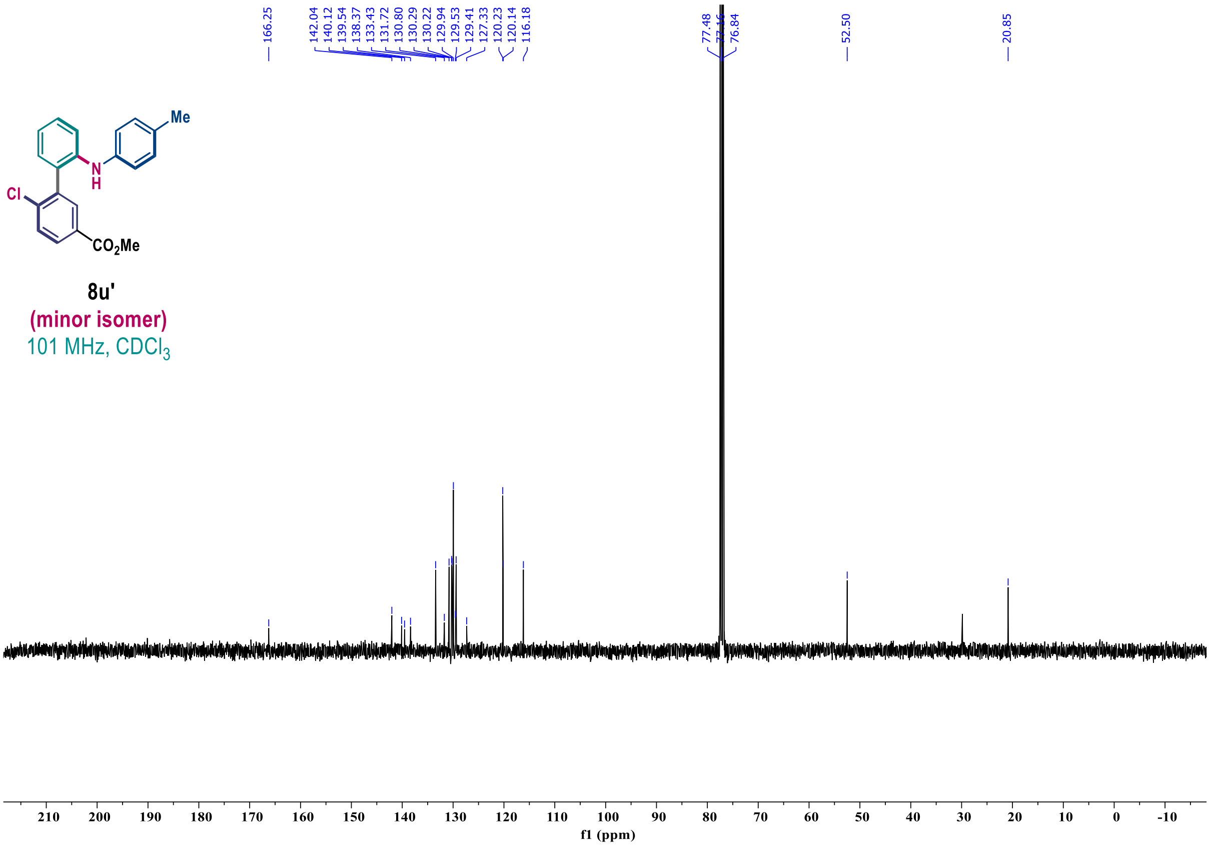

8u'

(minor isomer)

101 MHz, $CDCl_3$

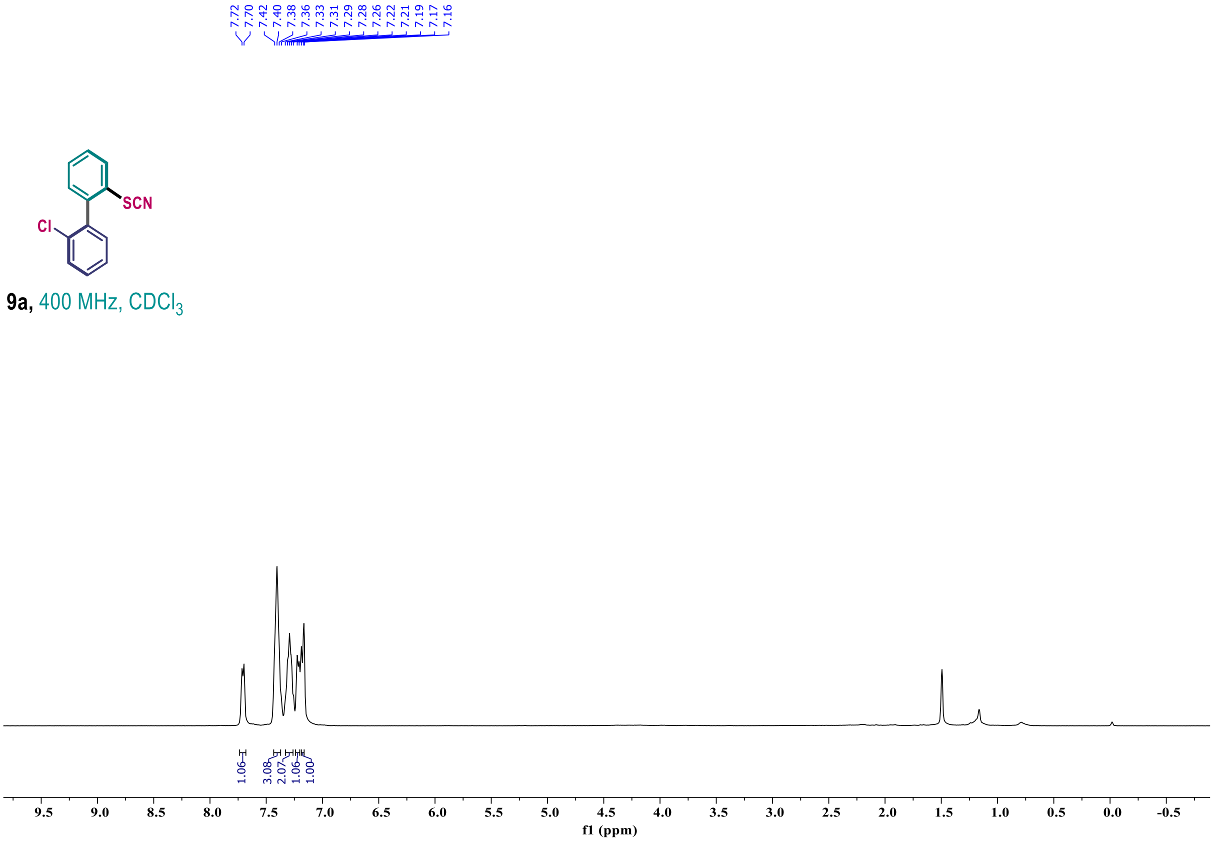

7.72
7.70
7.42
7.40
7.38
7.36
7.33
7.31
7.29
7.28
7.26
7.22
7.21
7.19
7.17
7.16
SCN
Cl
9a, 400 MHz, $CDCl_3$
1.06
3.08
2.07
1.06
1.00
f1 (ppm)

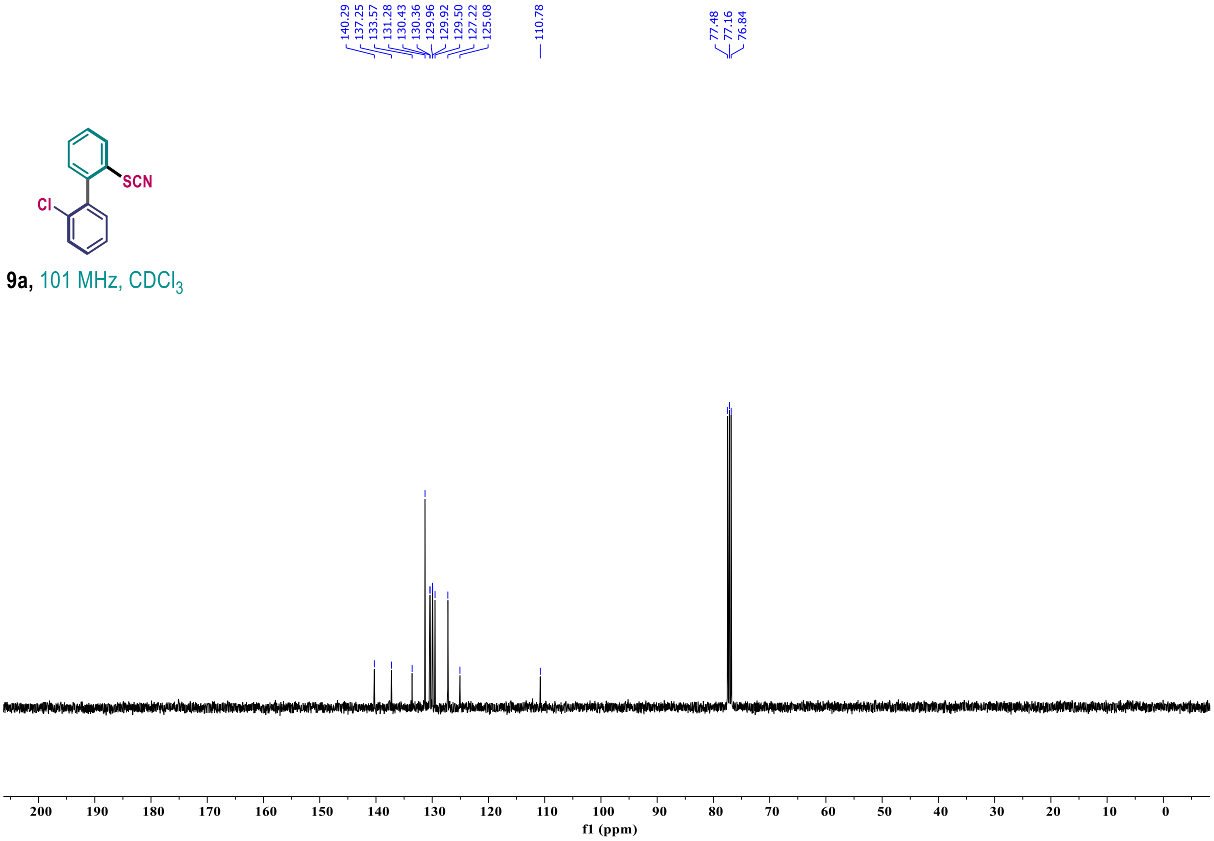

140.29
137.25
133.57
131.28
130.43
130.36
129.96
129.92
129.50
127.22
125.08
110.78
77.48
77.16
76.84
SCN
Cl
9a, 101 MHz, $CDCl_3$
f1 (ppm)

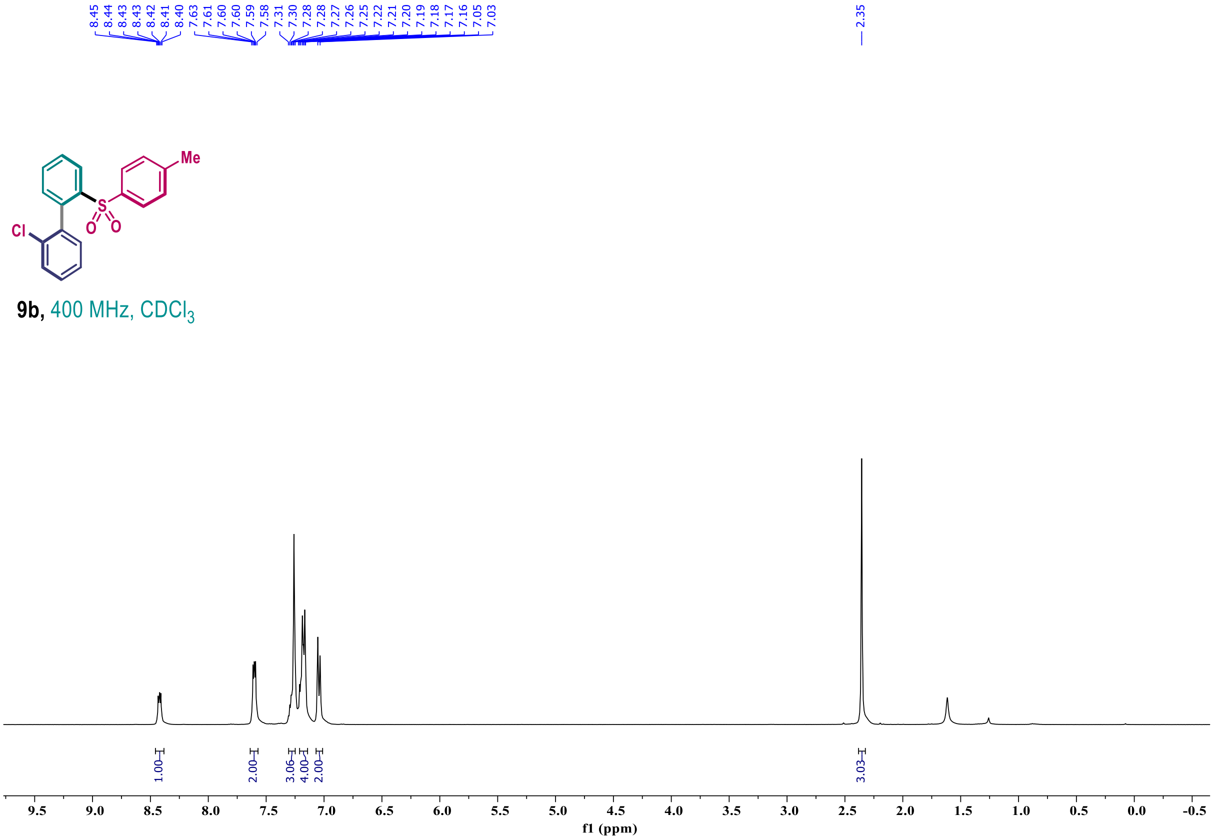
8.45
8.44
8.43
8.43
8.42
8.41
8.40
7.63
7.61
7.60
7.60
7.59
7.58
7.31
7.30
7.28
7.28
7.27
7.26
7.25
7.22
7.21
7.20
7.19
7.18
7.17
7.16
7.05
7.03
2.35
Me
S
O
O
Cl
9b, 400 MHz, $CDCl_3$
1.00
2.00
3.06
4.00
2.00
3.03
9.5 9.0 8.5 8.0 7.5 7.0 6.5 6.0 5.5 5.0 4.5 4.0 3.5 3.0 2.5 2.0 1.5 1.0 0.5 0.0 -0.5
f1 (ppm)

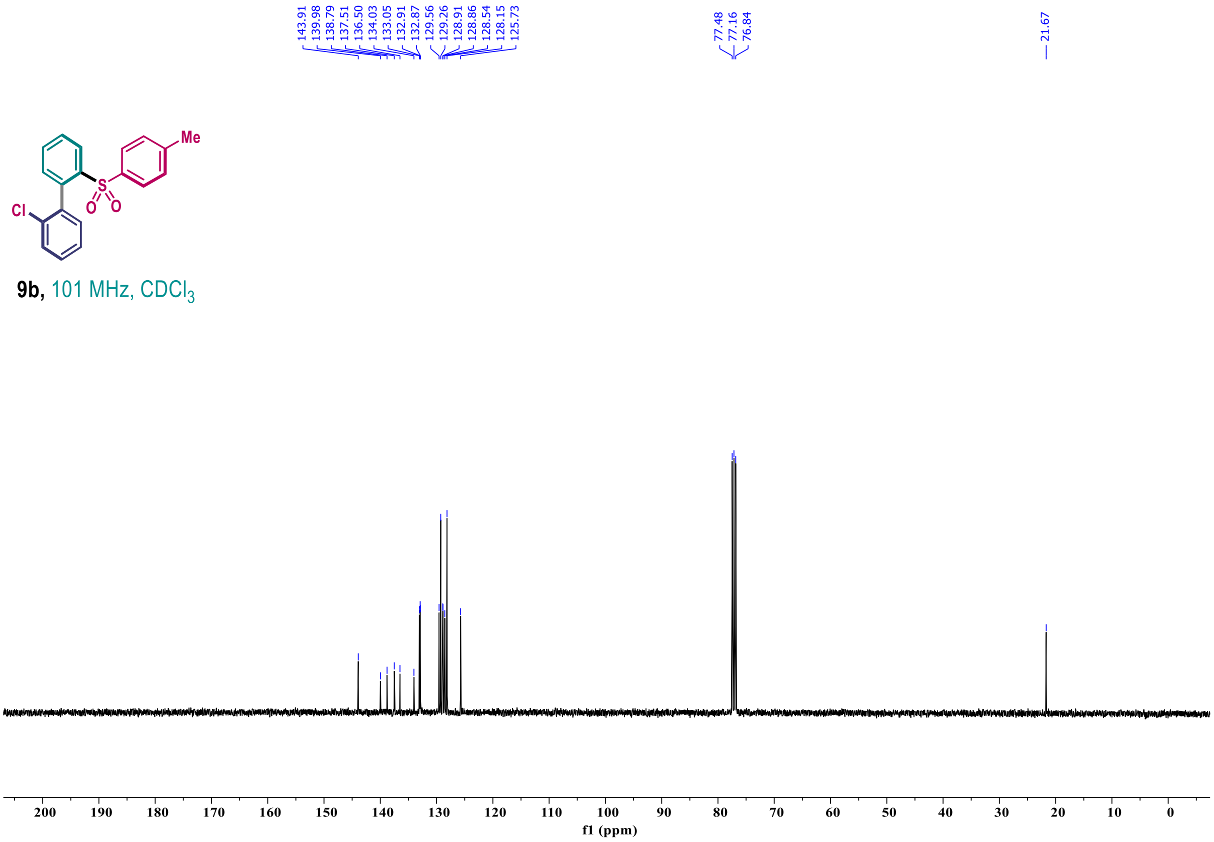
143.91
139.98
138.79
137.51
136.50
134.03
133.05
132.91
132.87
129.56
129.26
128.91
128.86
128.54
128.15
125.73
77.48
77.16
76.84
21.67
Me
S
O
O
Cl
9b, 101 MHz, $CDCl_3$
200 190 180 170 160 150 140 130 120 110 100 90 80 70 60 50 40 30 20 10 0
f1 (ppm)

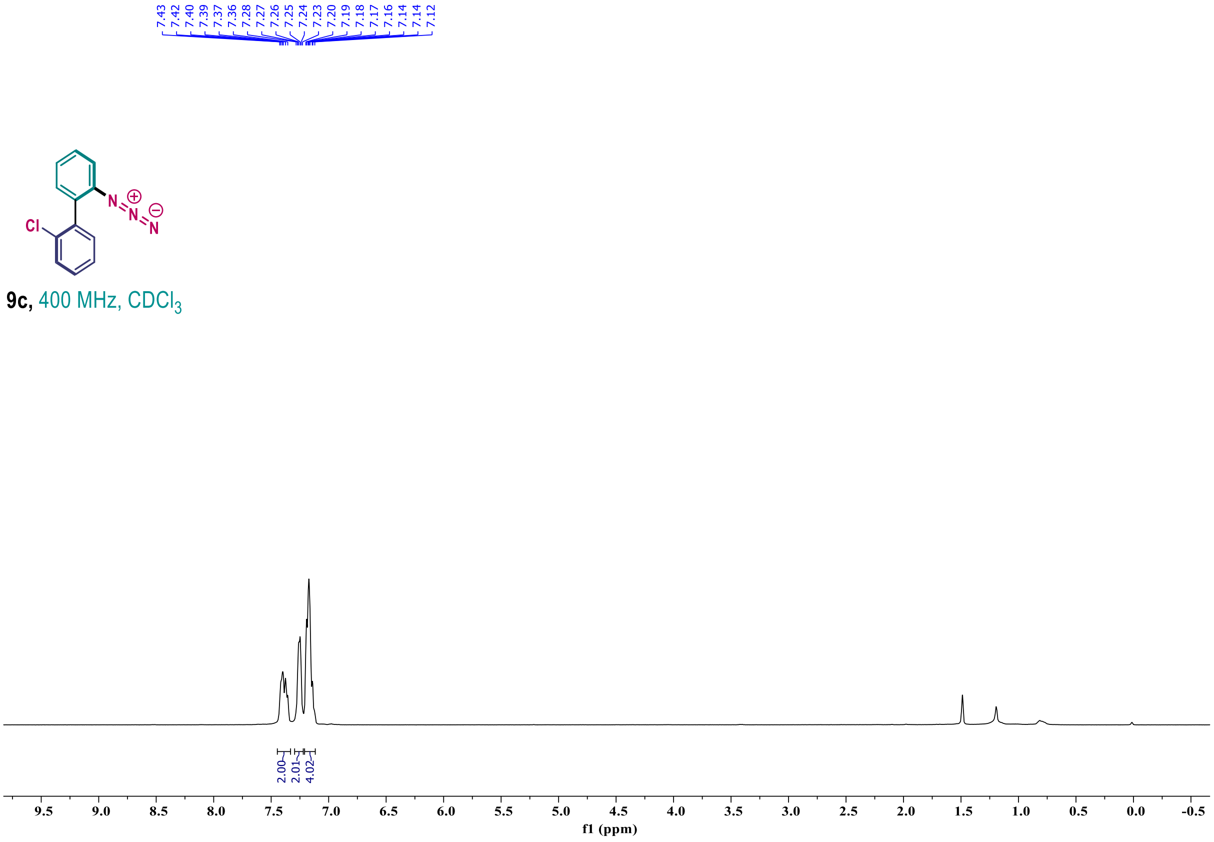
7.43
7.42
7.40
7.39
7.37
7.36
7.28
7.27
7.26
7.25
7.24
7.23
7.20
7.19
7.18
7.17
7.16
7.14
7.14
7.12
Cl
N
N
N
9c, 400 MHz, CDCl3
2.00
2.01
4.02
9.5
9.0
8.5
8.0
7.5
7.0
6.5
6.0
5.5
5.0
4.5
4.0
3.5
3.0
2.5
2.0
1.5
1.0
0.5
0.0
-0.5
f1 (ppm)

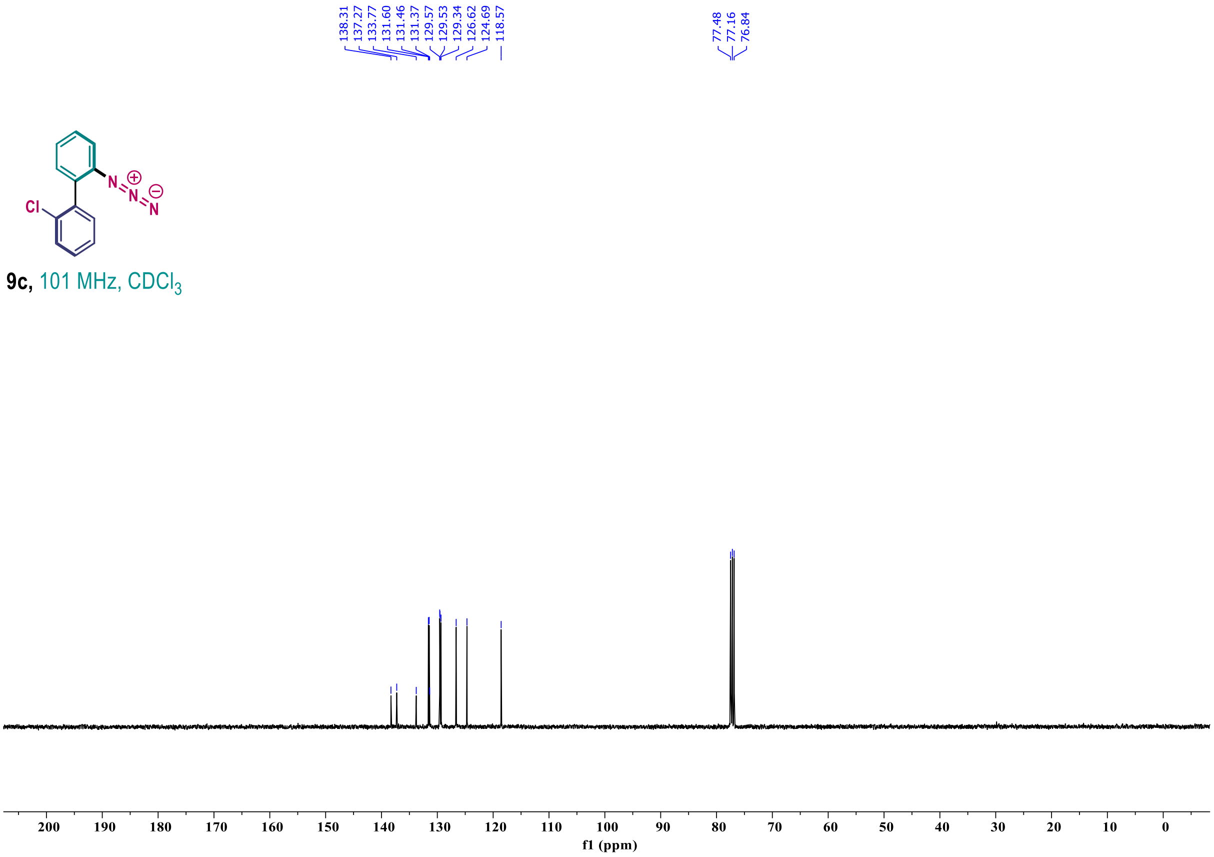
138.31
137.27
133.77
131.60
131.46
131.37
129.57
129.53
129.34
126.62
124.69
118.57
77.48
77.16
76.84
Cl
N
N
N
9c, 101 MHz, CDCl3
200
190
180
170
160
150
140
130
120
110
100
90
80
70
60
50
40
30
20
10
0
f1 (ppm)

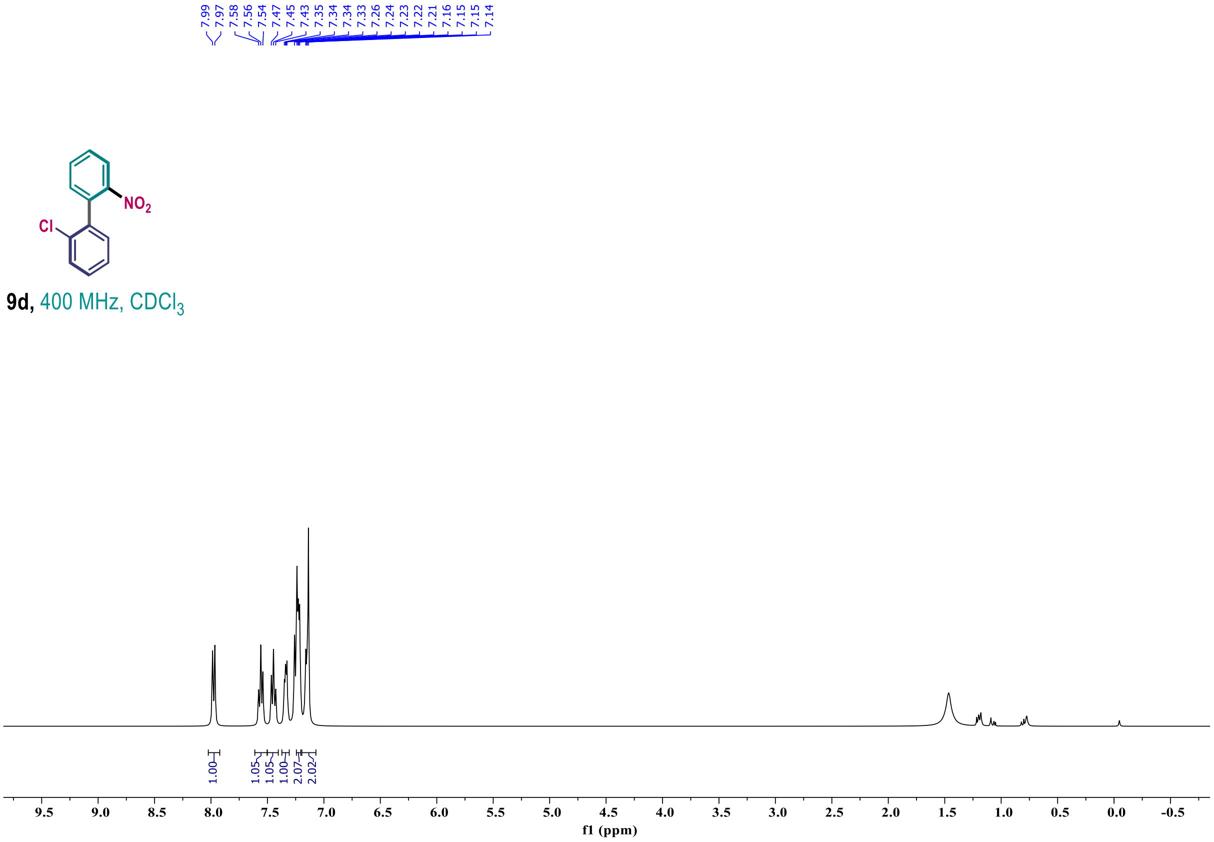
7.99
7.97
7.58
7.56
7.54
7.47
7.45
7.43
7.35
7.34
7.34
7.33
7.26
7.24
7.23
7.22
7.21
7.16
7.15
7.14
NO2
Cl
9d, 400 MHz, CDCl3
1.00
1.05
1.05
1.00
2.07
2.02
9.5 9.0 8.5 8.0 7.5 7.0 6.5 6.0 5.5 5.0 4.5 4.0 3.5 3.0 2.5 2.0 1.5 1.0 0.5 0.0 -0.5
f1 (ppm)

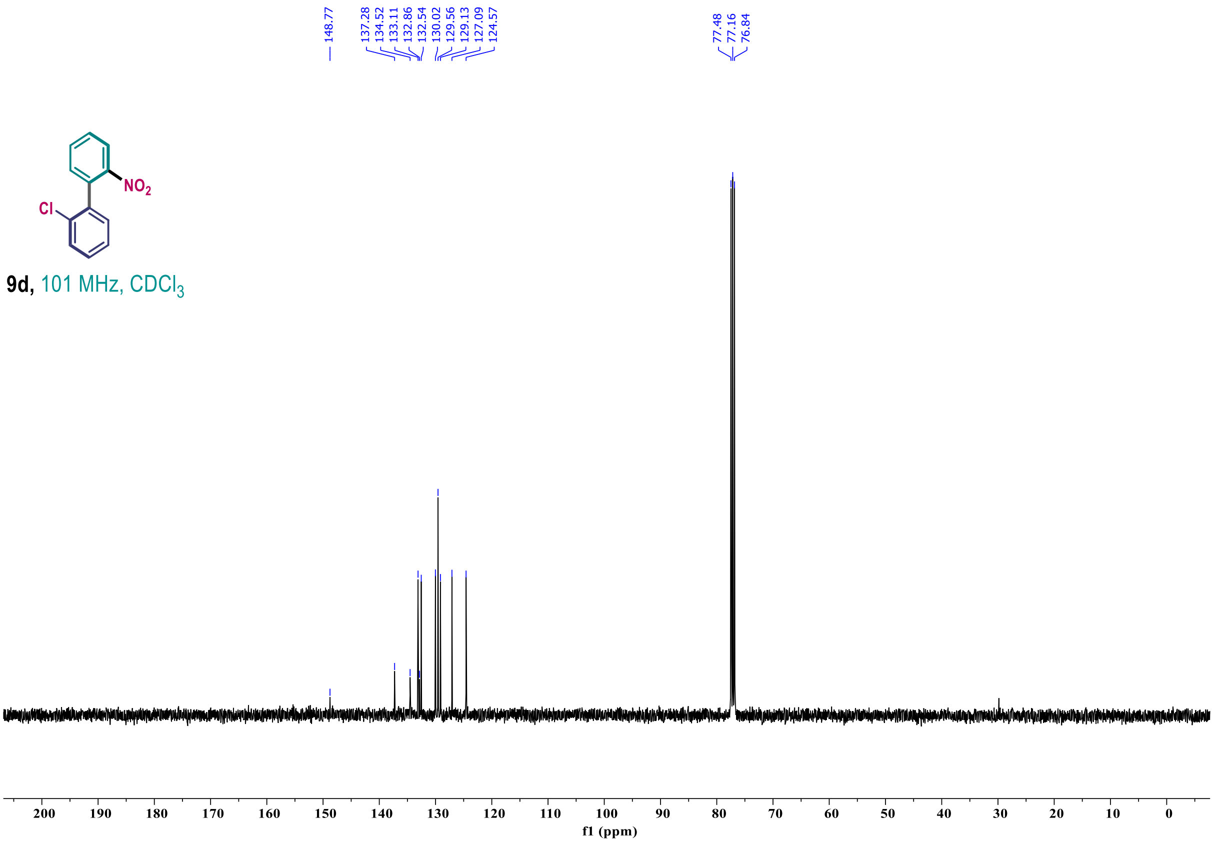
148.77
137.28
134.52
133.11
132.86
132.54
130.02
129.56
129.13
127.09
124.57
77.48
77.16
76.84
NO2
Cl
9d, 101 MHz, CDCl3
200 190 180 170 160 150 140 130 120 110 100 90 80 70 60 50 40 30 20 10 0
f1 (ppm)

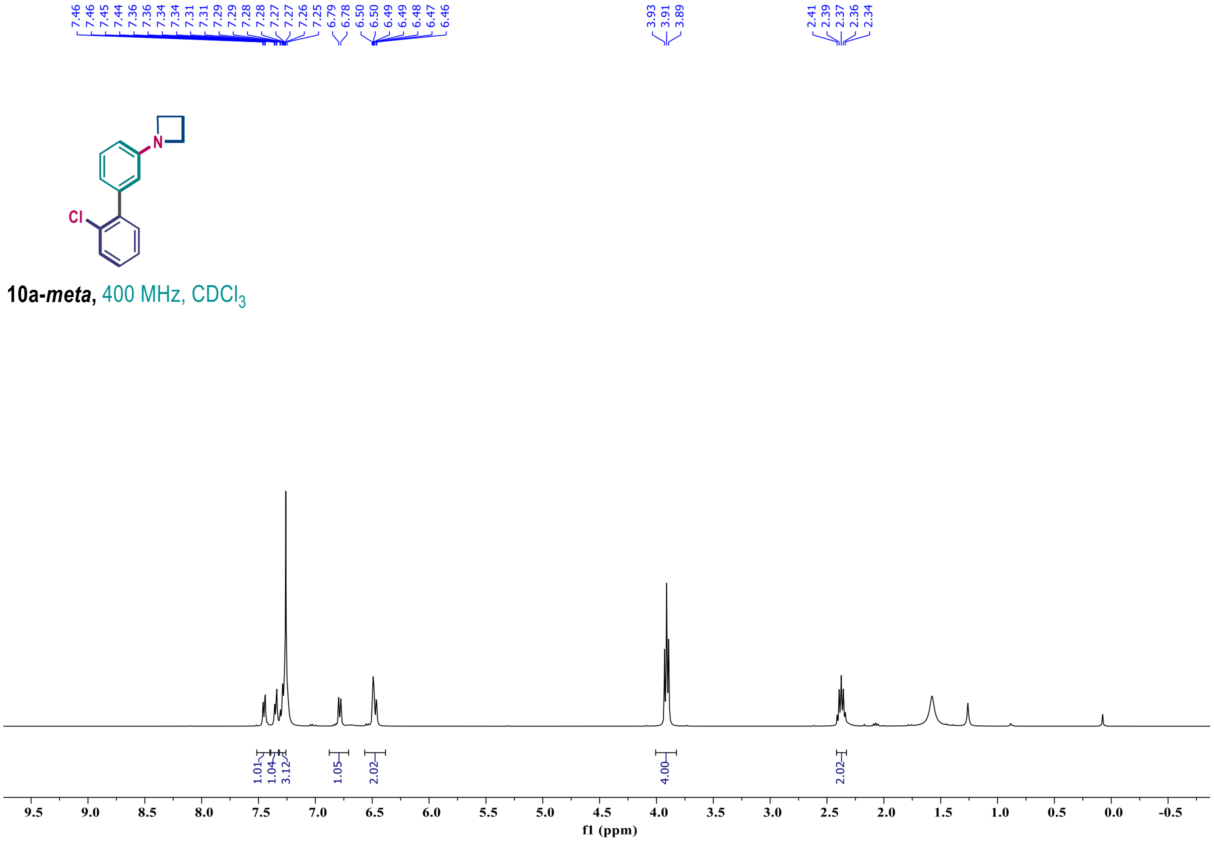

10a-*meta*, 400 MHz, $CDCl_3$

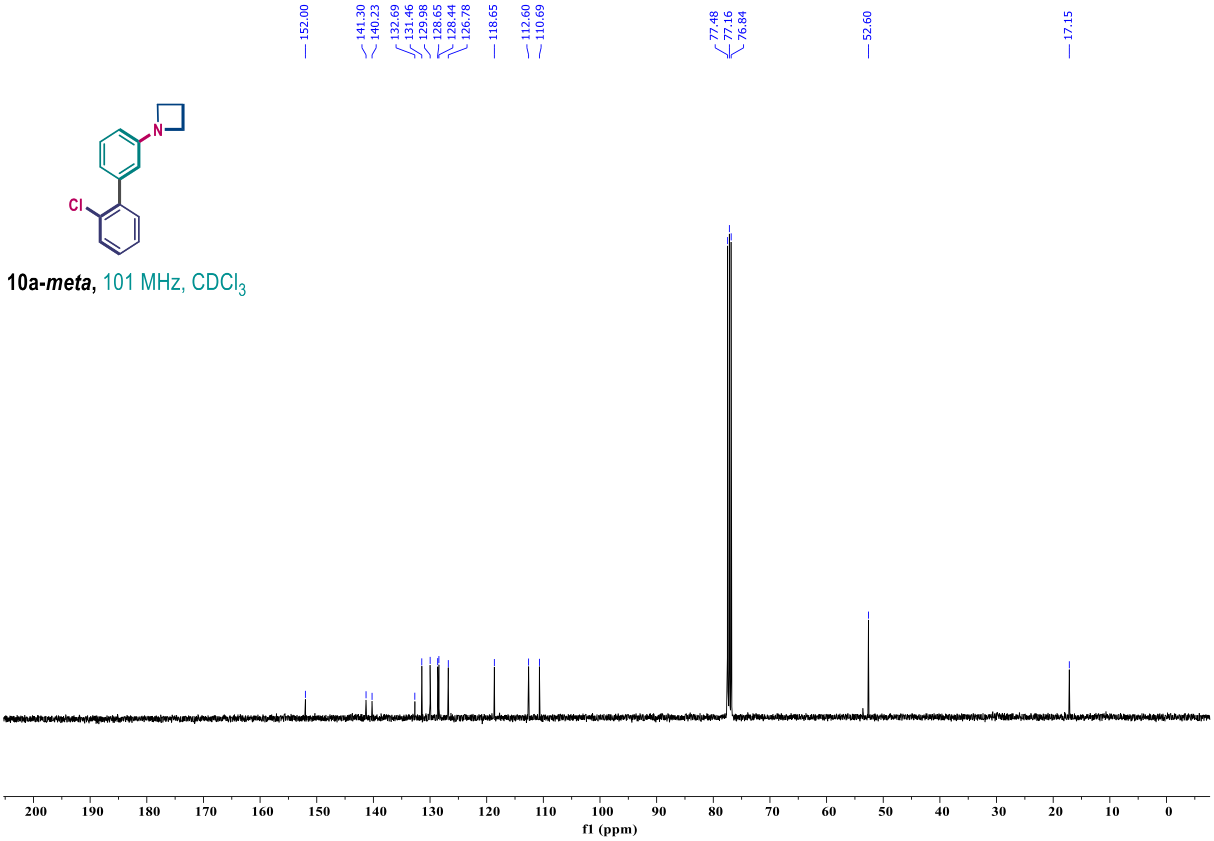

10a-*meta*, 101 MHz, $CDCl_3$

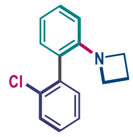

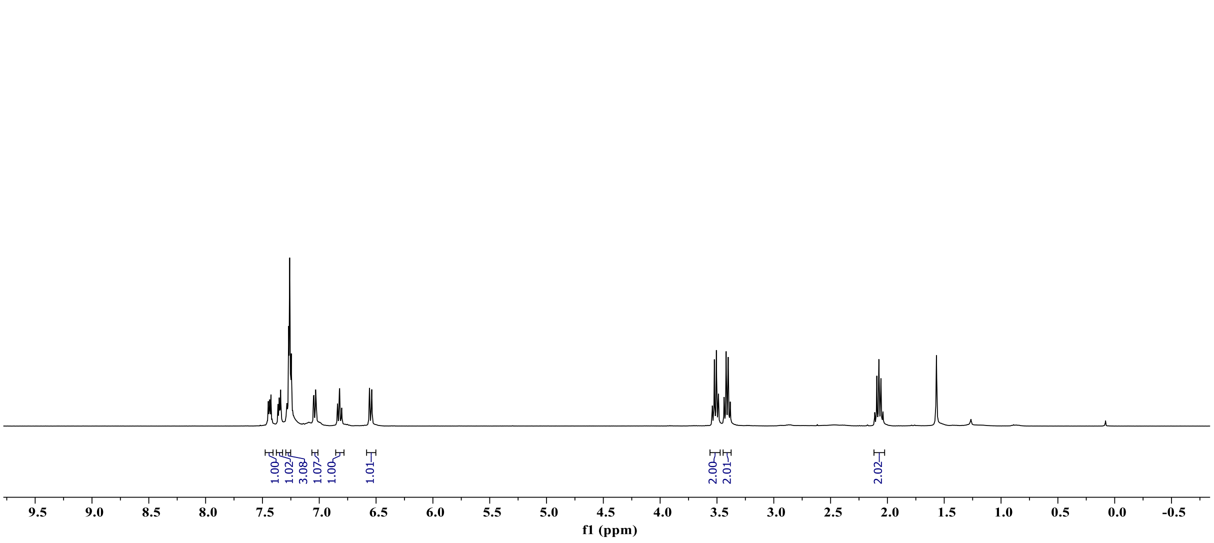

10a-*ortho*, 400 MHz, $CDCl_3$

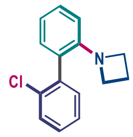

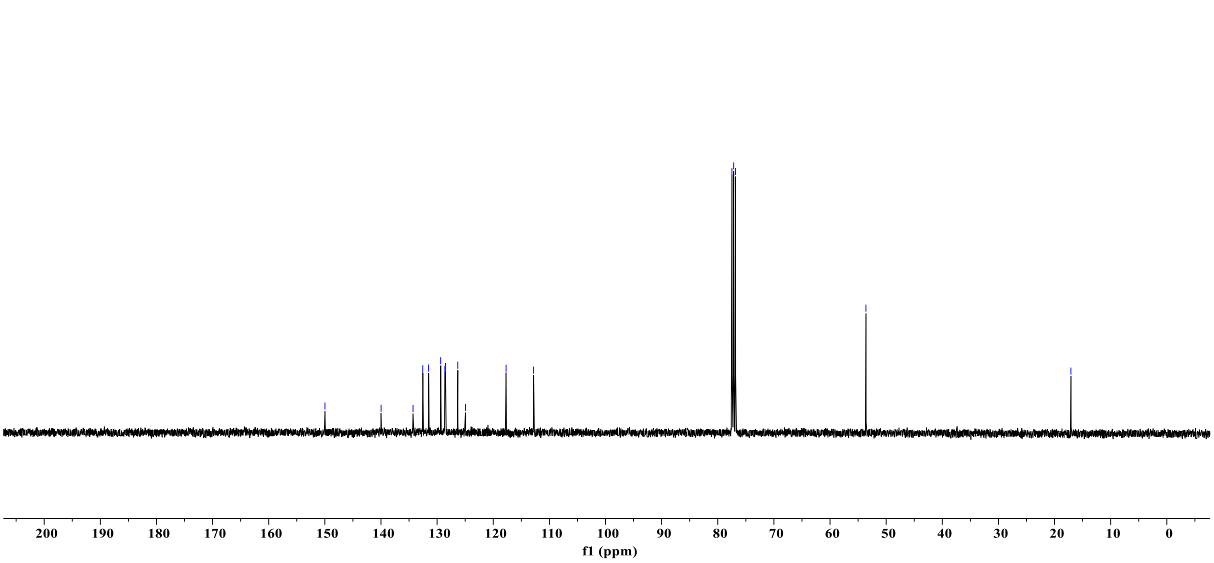

10a-*ortho*, 101 MHz, $CDCl_3$

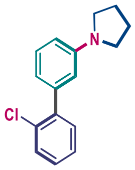

10b-*meta*, 400 MHz, $CDCl_3$

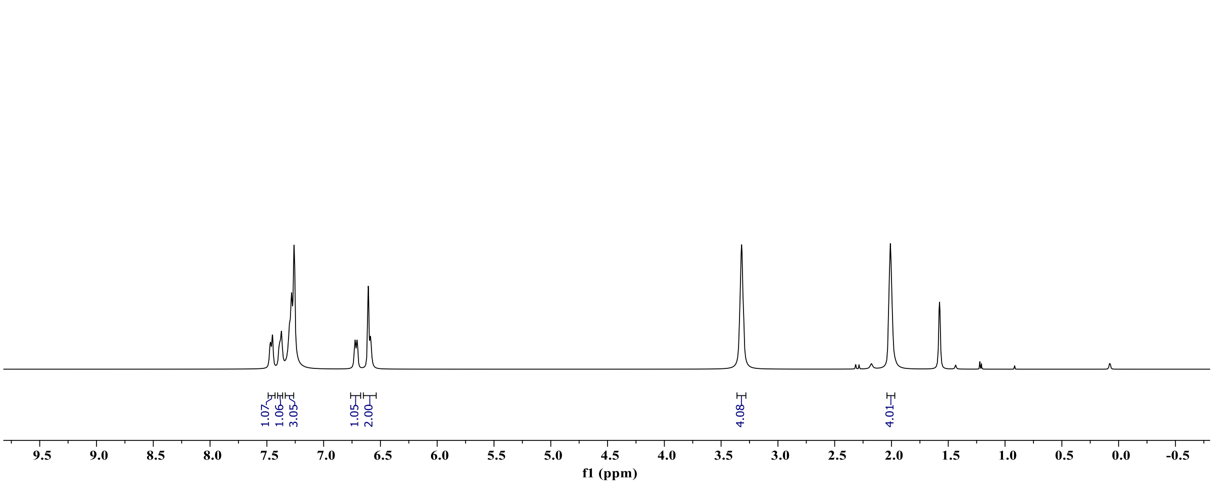

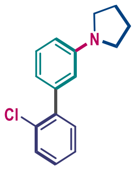

10b-*meta*, 101 MHz, $CDCl_3$

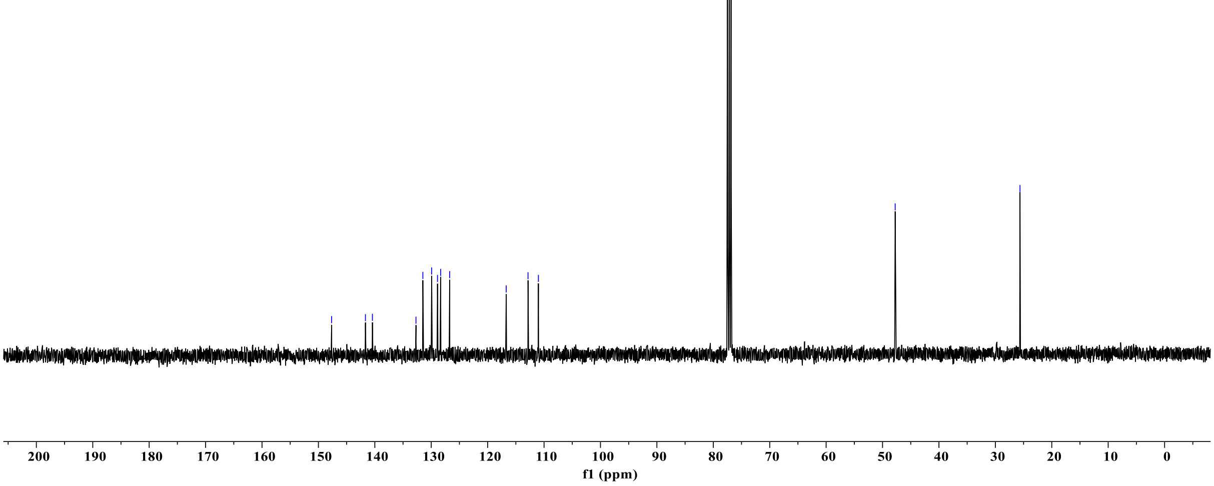

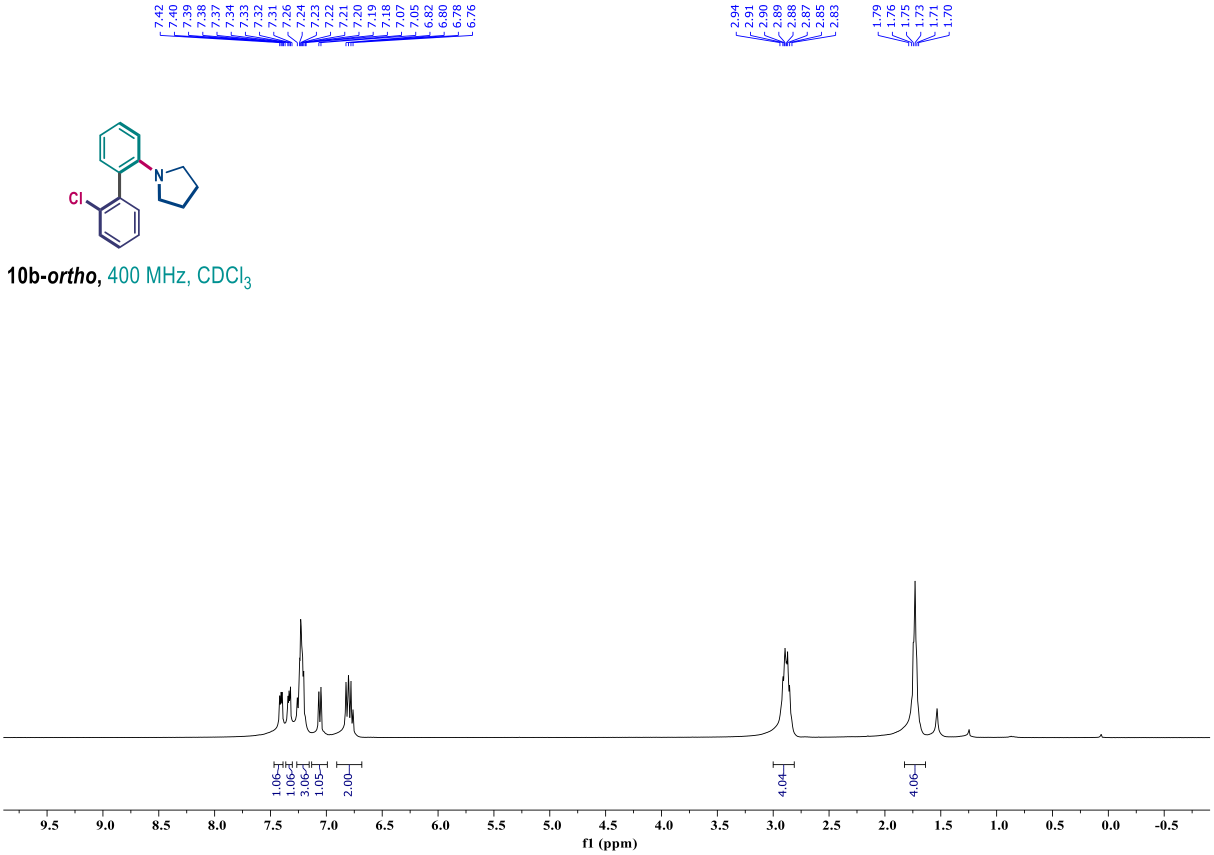

10b-*ortho*, 400 MHz, $CDCl_3$

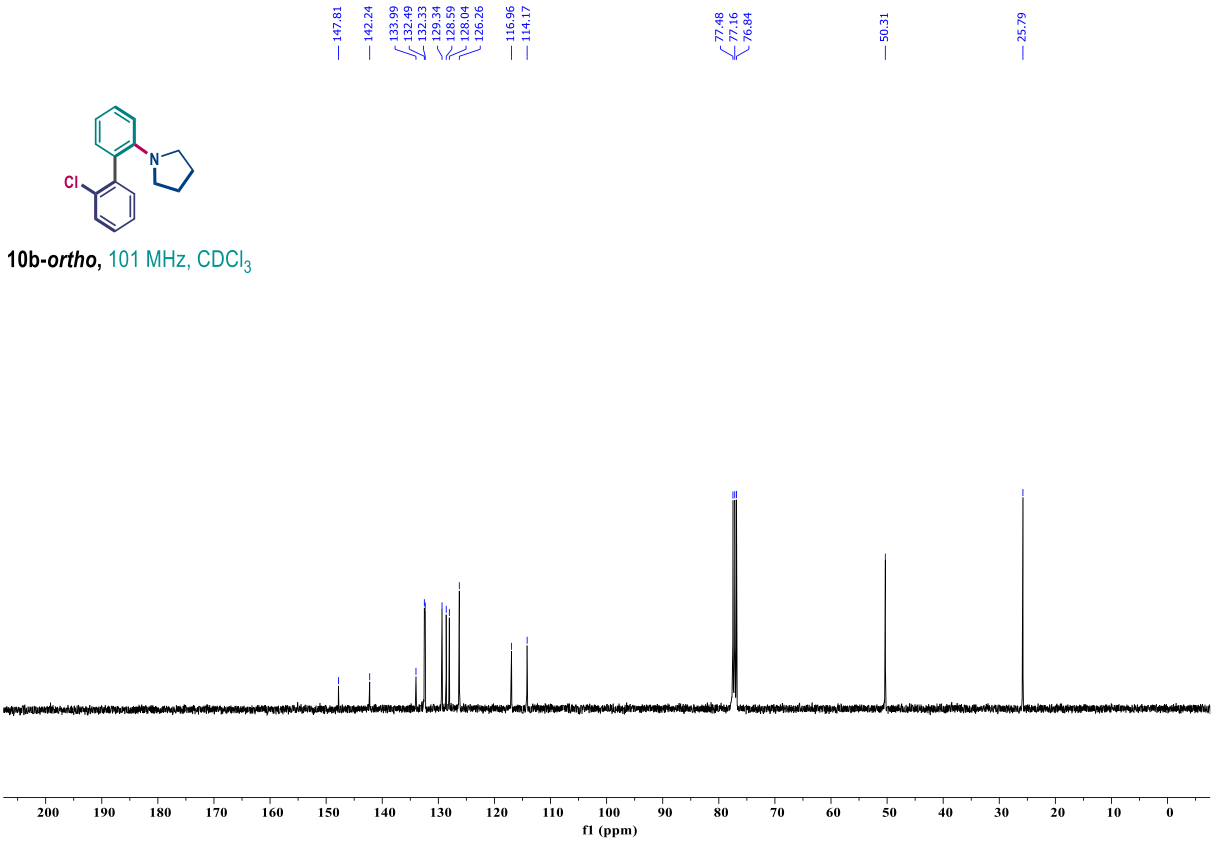

10b-*ortho*, 101 MHz, $CDCl_3$

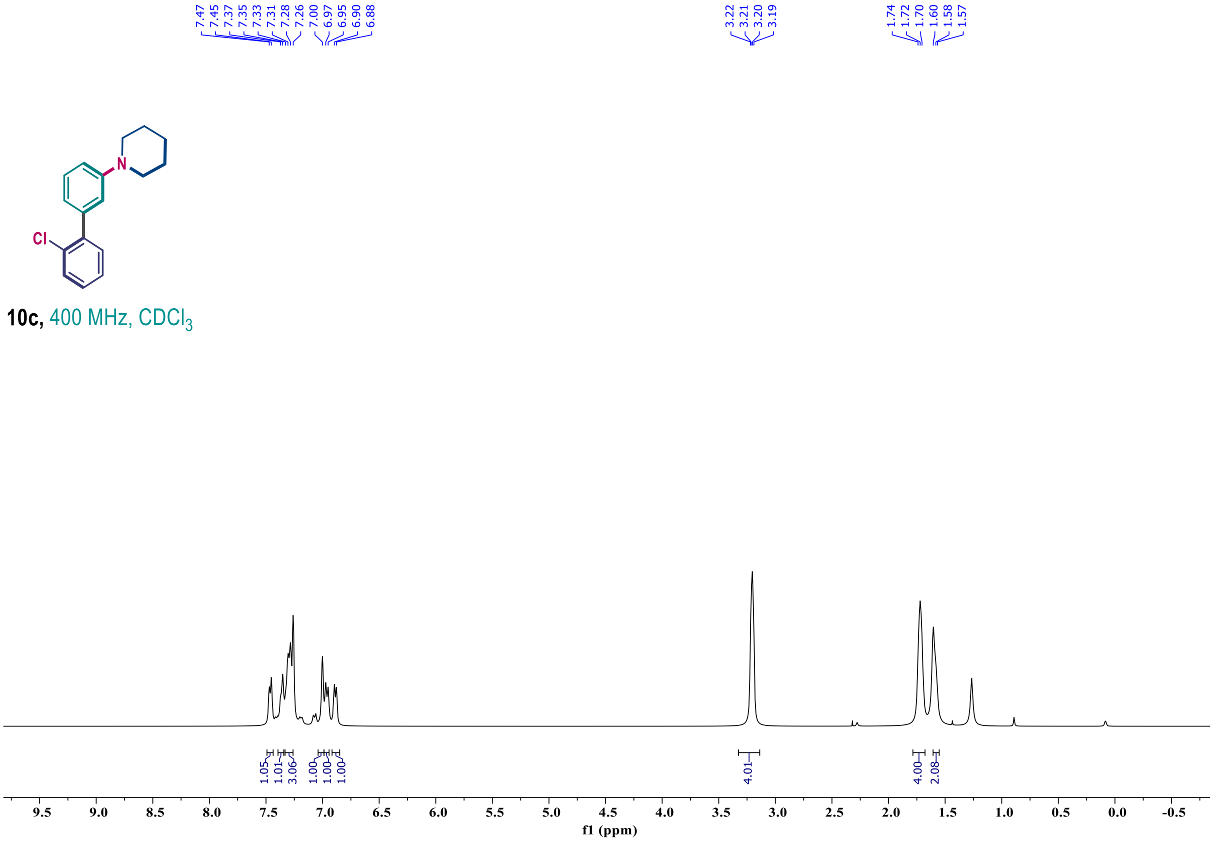

10c, 400 MHz, $CDCl_3$

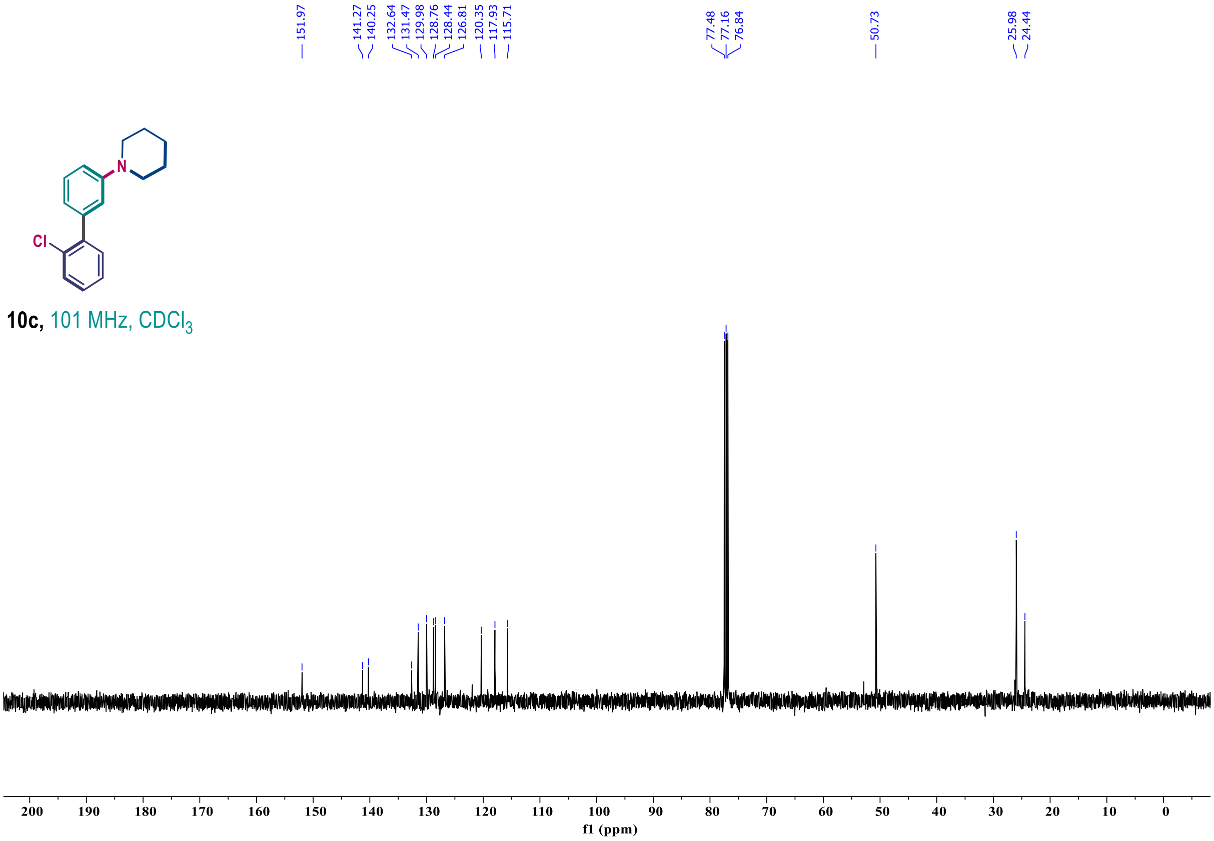

10c, 101 MHz, $CDCl_3$

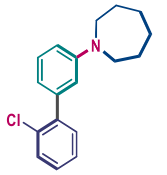

10d, 400 MHz, $CDCl_3$

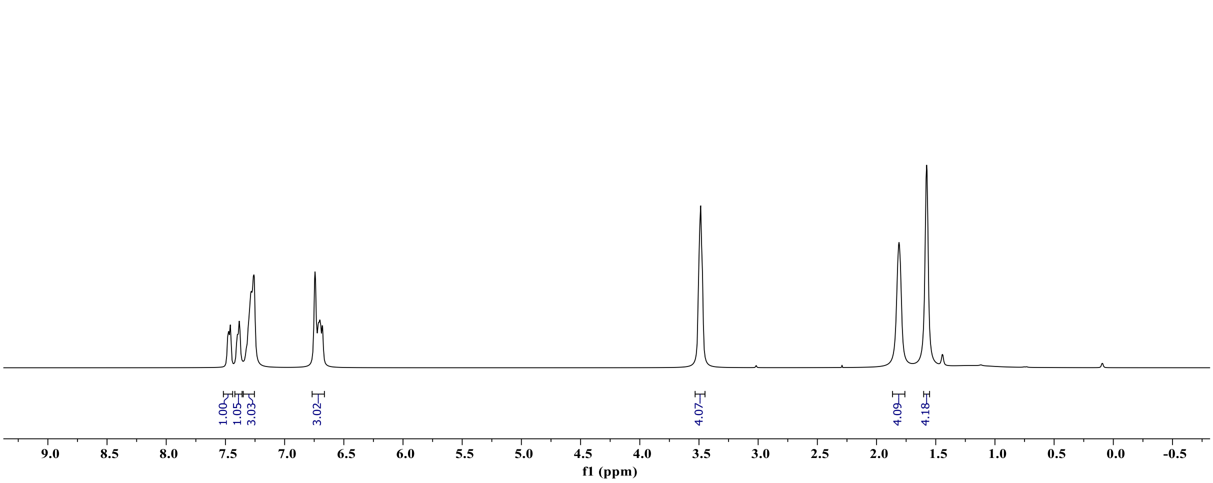

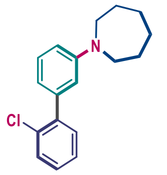

10d, 101 MHz, $CDCl_3$

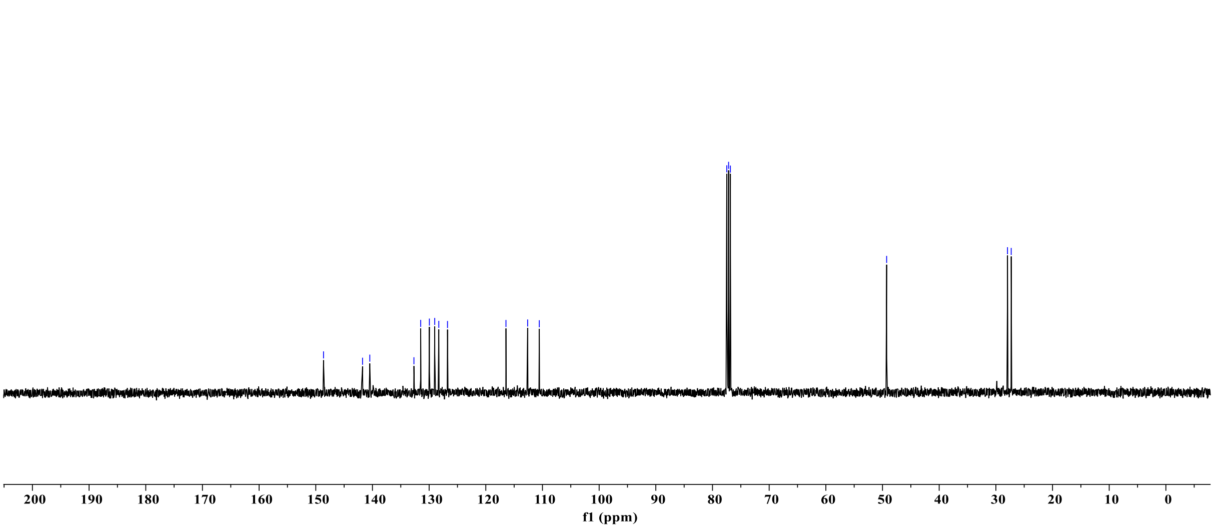

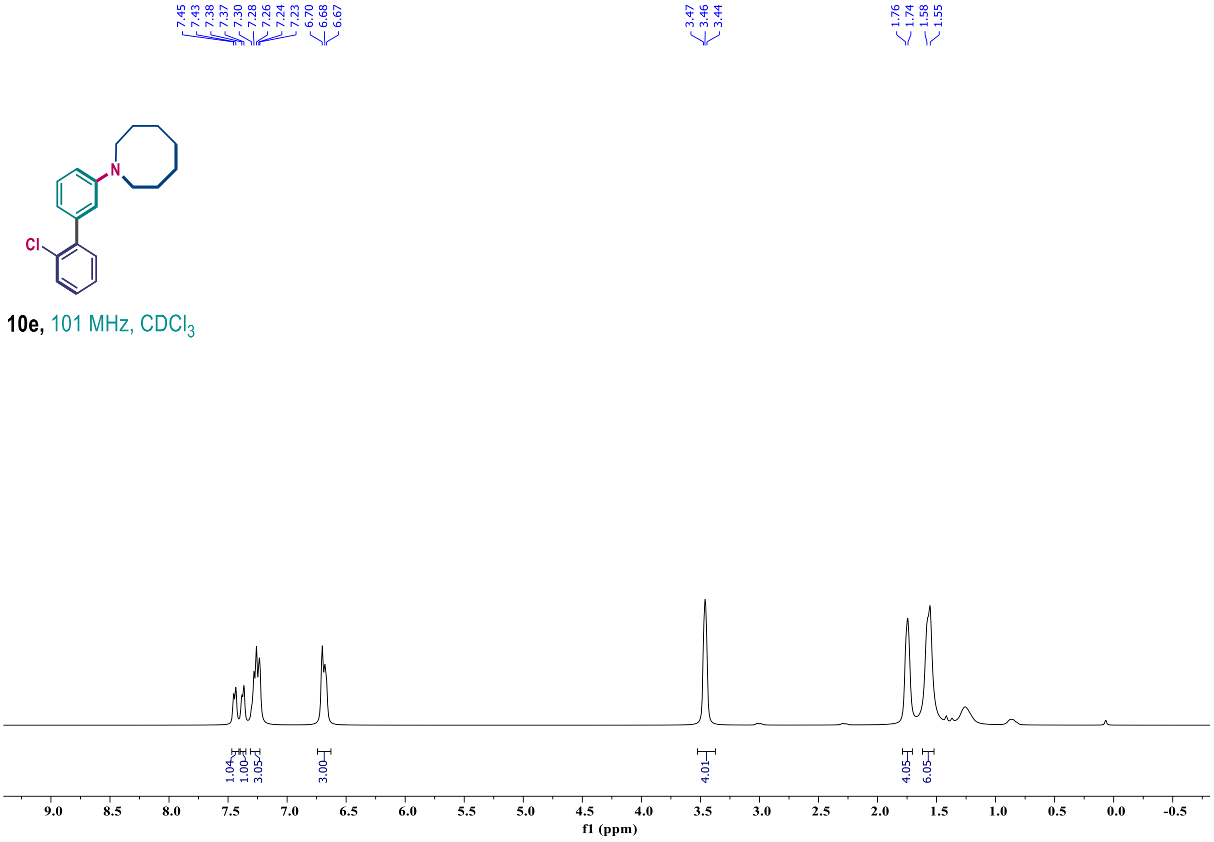
7.45
7.43
7.38
7.37
7.30
7.28
7.26
7.24
7.23
6.70
6.68
6.67
3.47
3.46
3.44
1.76
1.74
1.58
1.55
Cl
N
10e, 101 MHz, CDCl3
1.04
1.00
3.05
3.00
4.01
4.05
6.05
f1 (ppm)

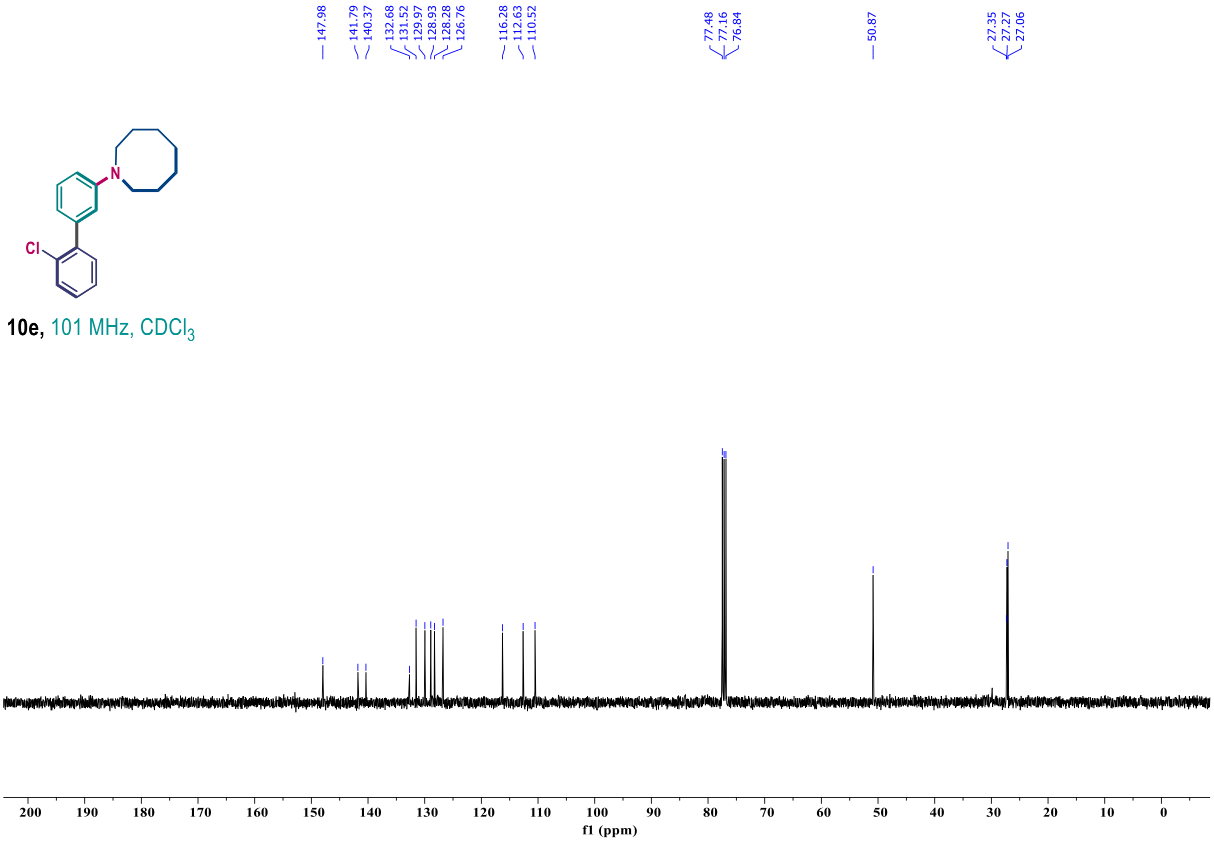
147.98
141.79
140.37
132.68
131.52
129.97
128.93
128.28
126.76
116.28
112.63
110.52
77.48
77.16
76.84
50.87
27.35
27.27
27.06
Cl
N
10e, 101 MHz, CDCl3
f1 (ppm)

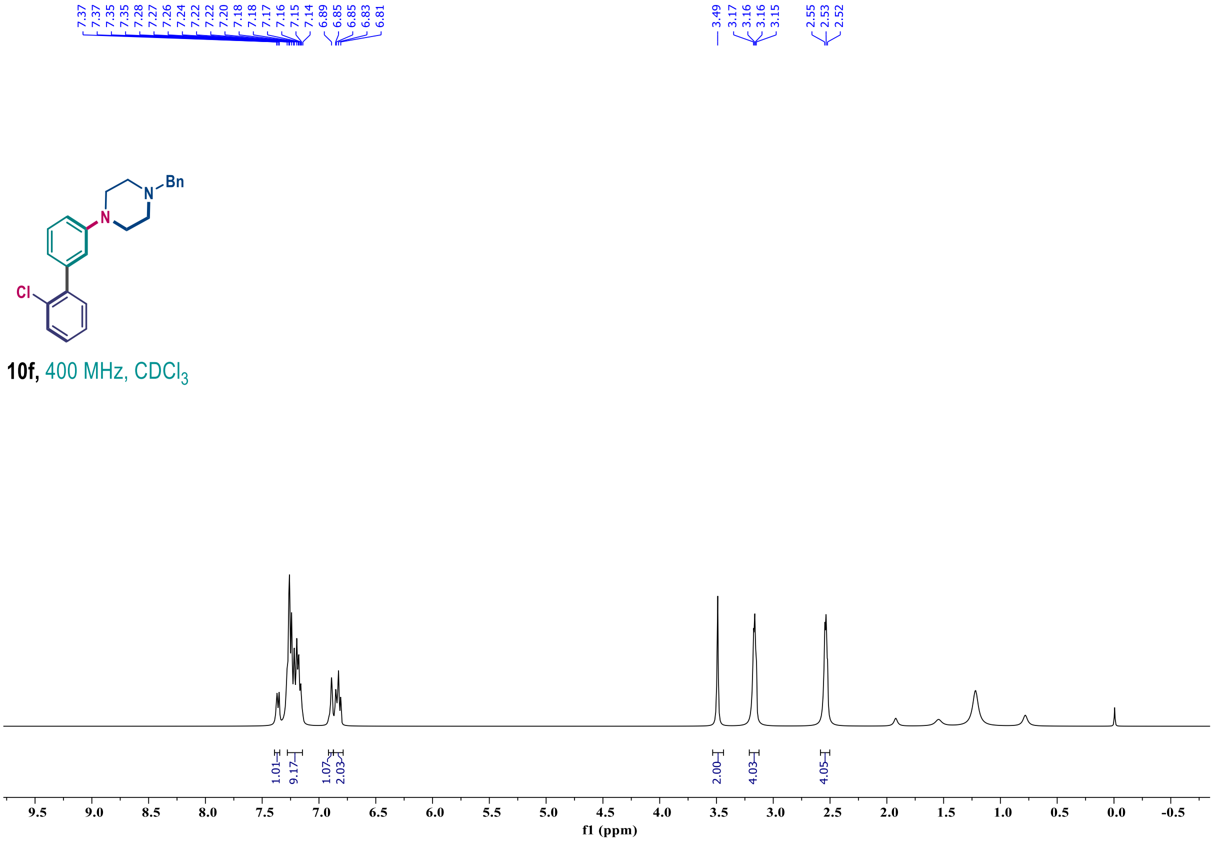

10f, 400 MHz, $CDCl_3$

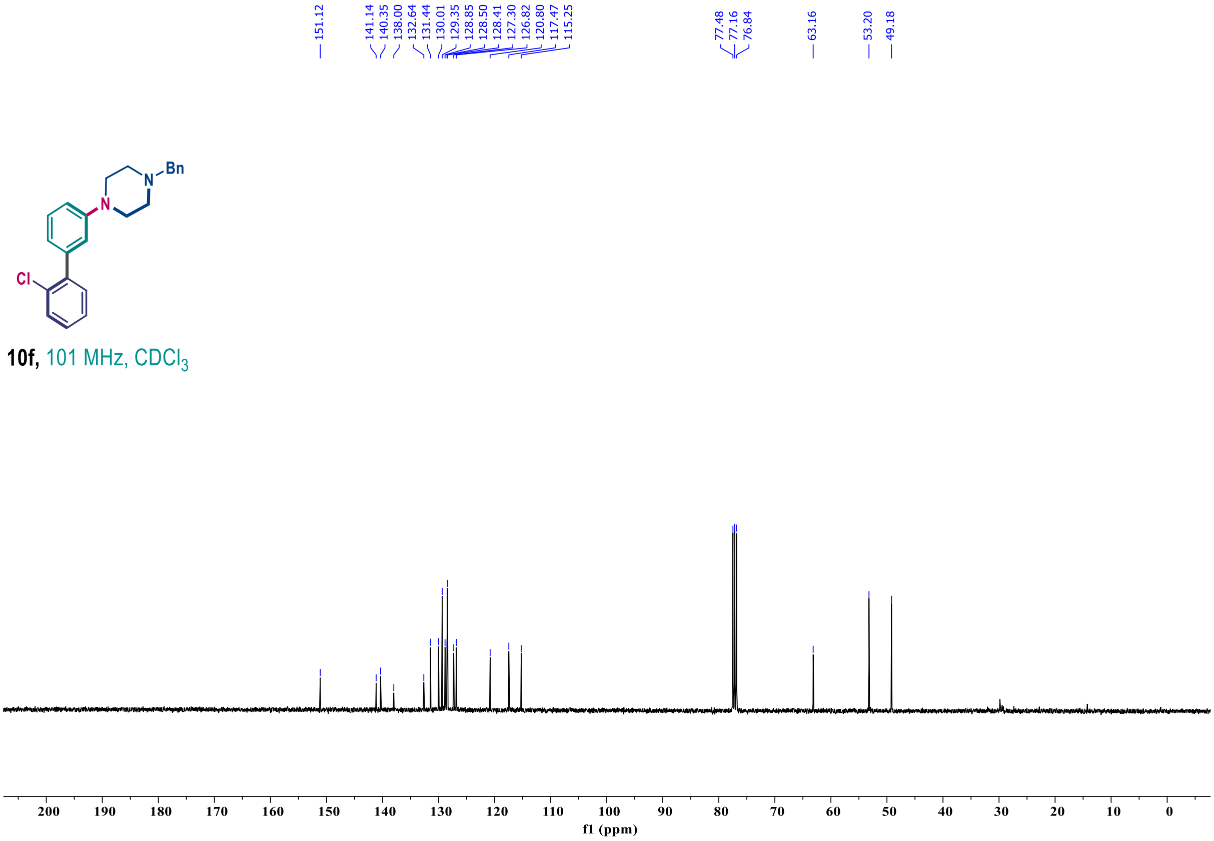

10f, 101 MHz, $CDCl_3$

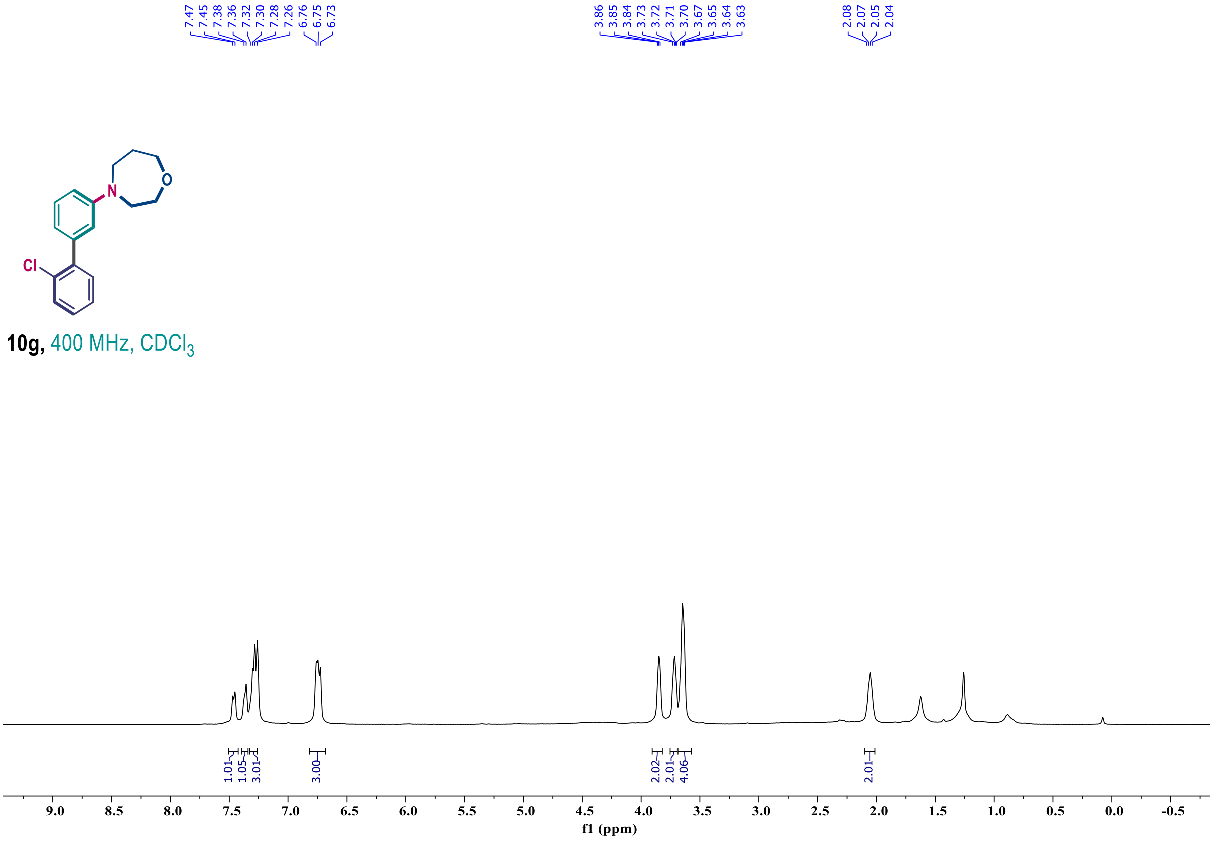

10g, 400 MHz, $CDCl_3$

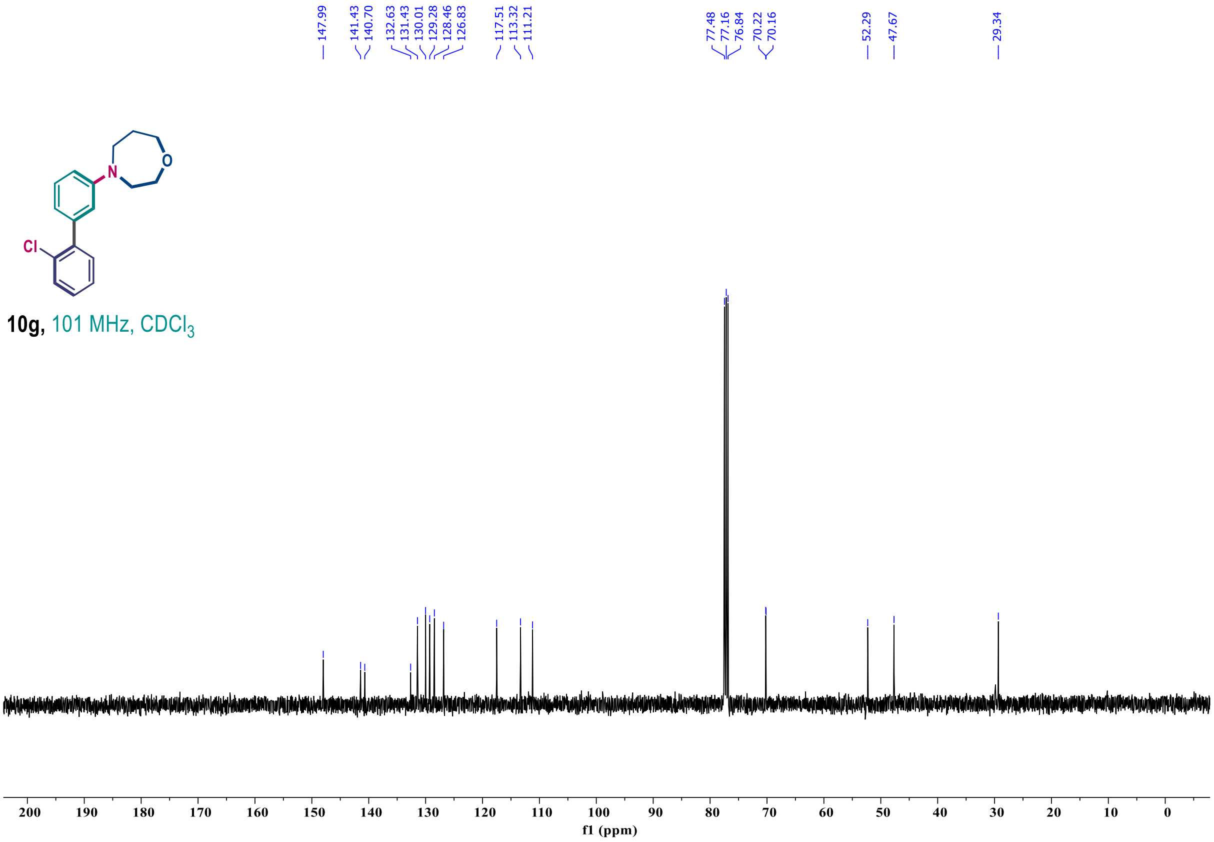

10g, 101 MHz, $CDCl_3$

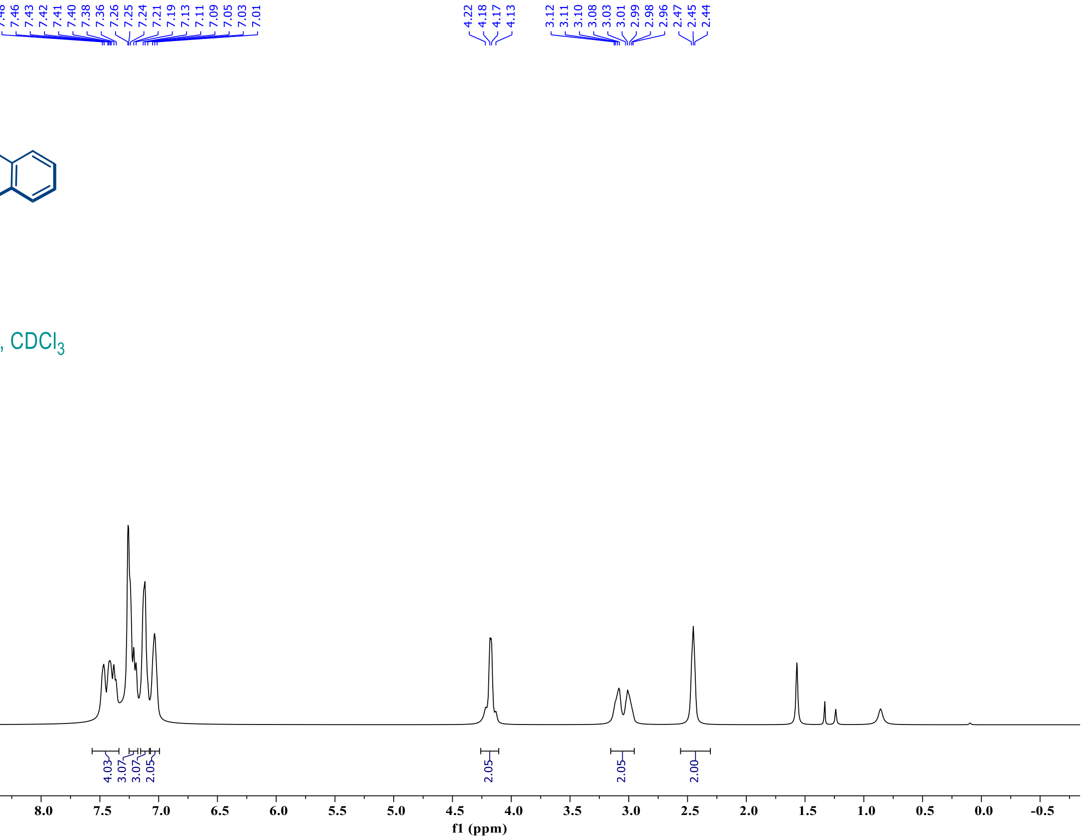

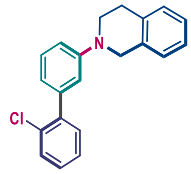

10h, 400 MHz, $CDCl_3$

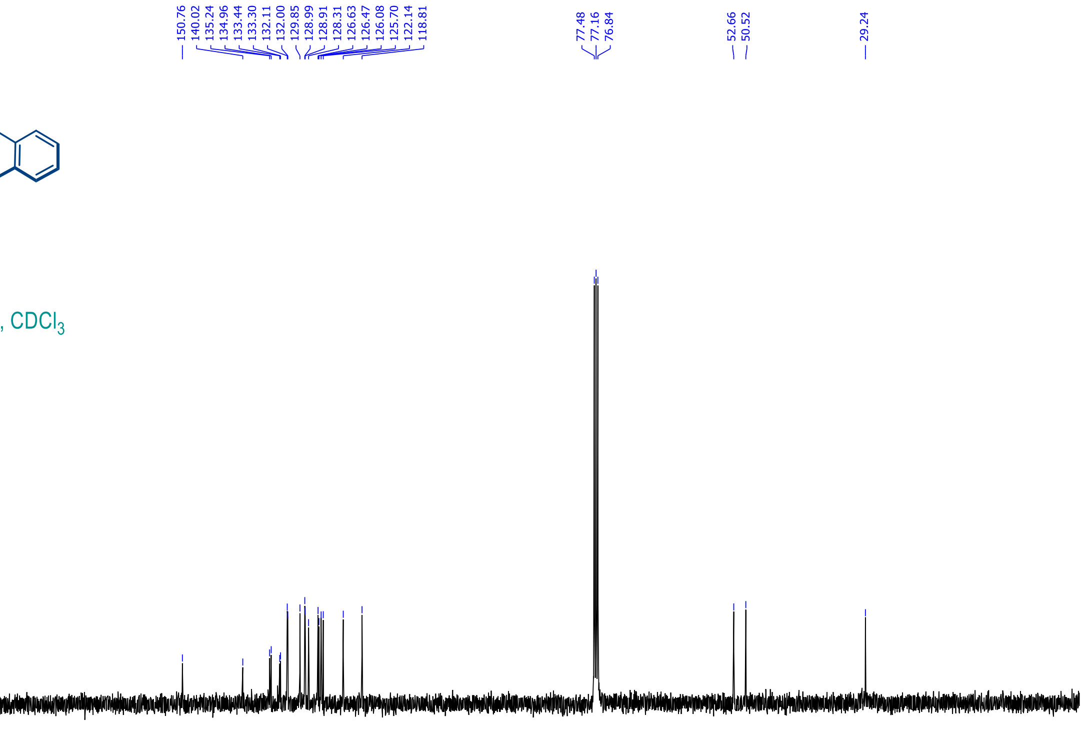

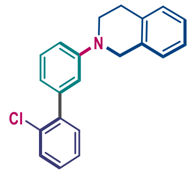

10h, 101 MHz, $CDCl_3$

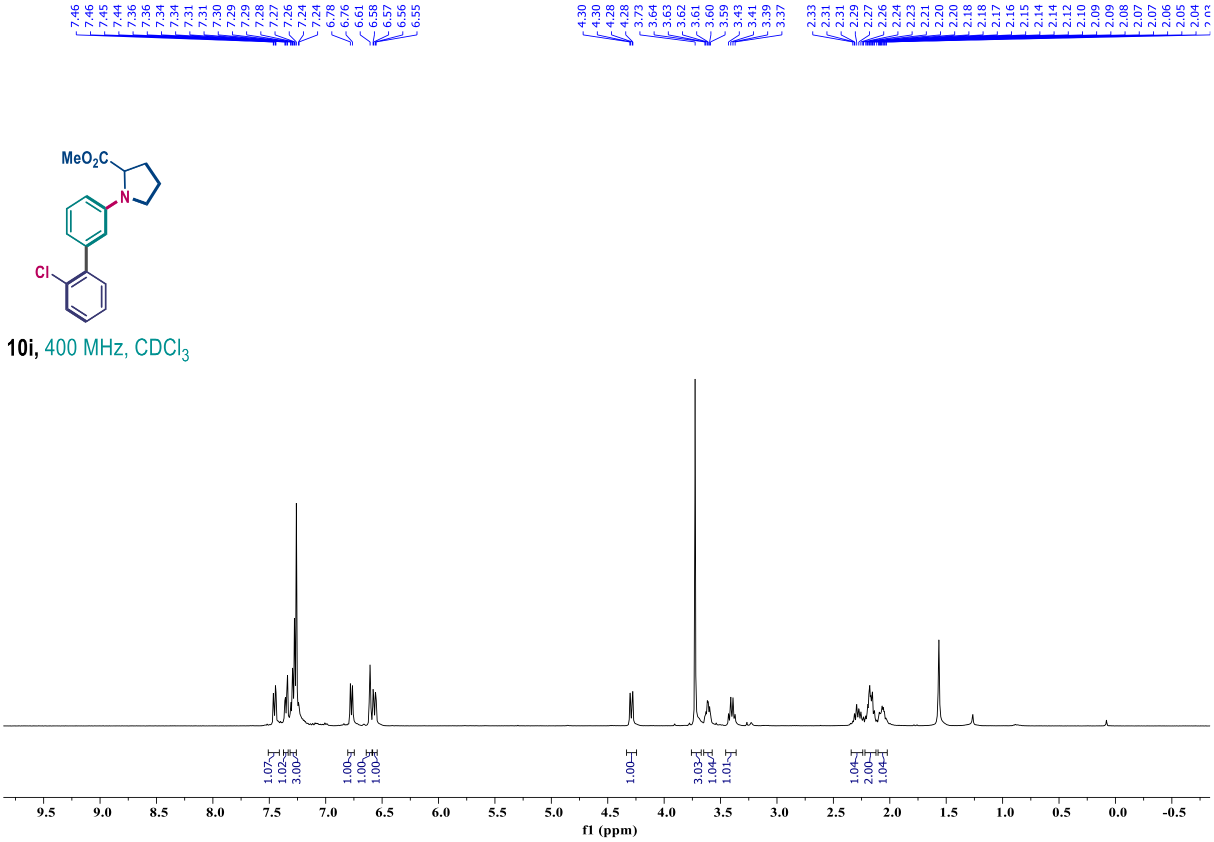

10i, 400 MHz, $CDCl_3$

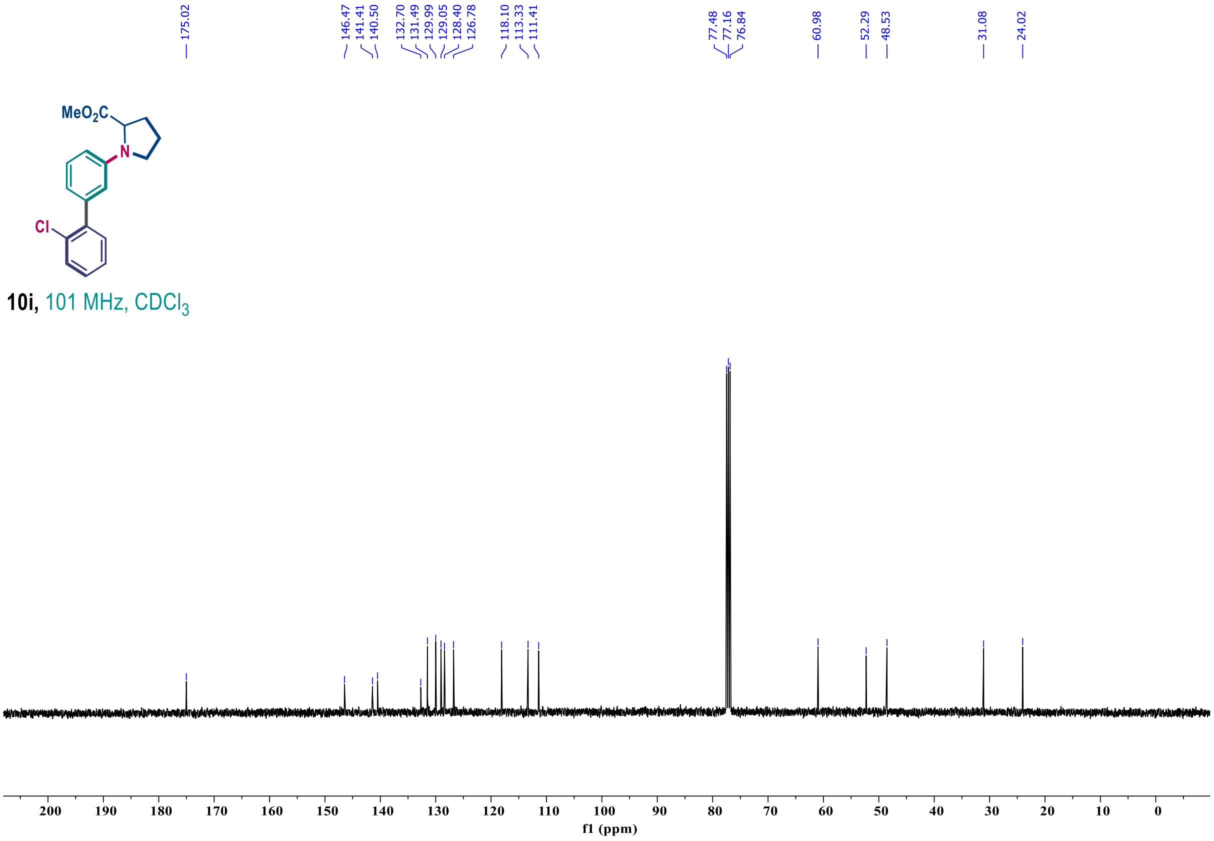

10i, 101 MHz, $CDCl_3$

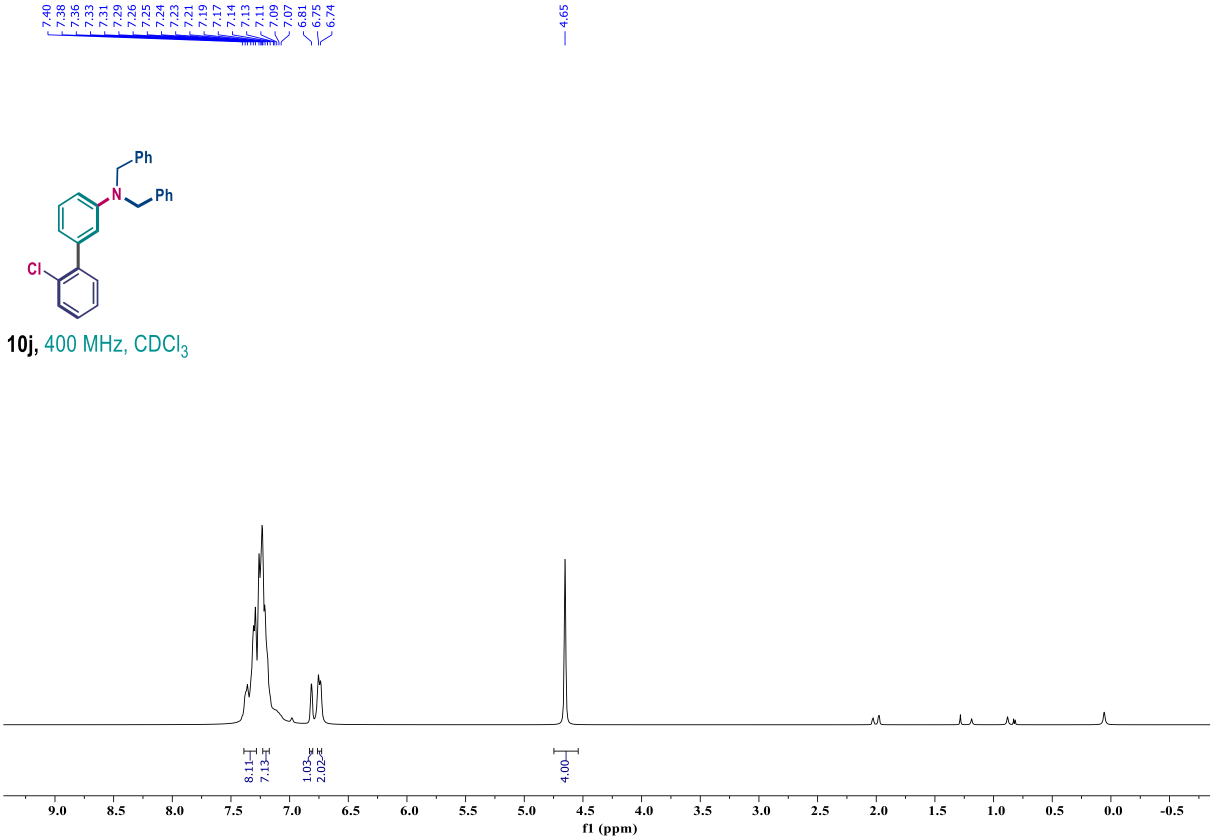

10j, 400 MHz, $CDCl_3$

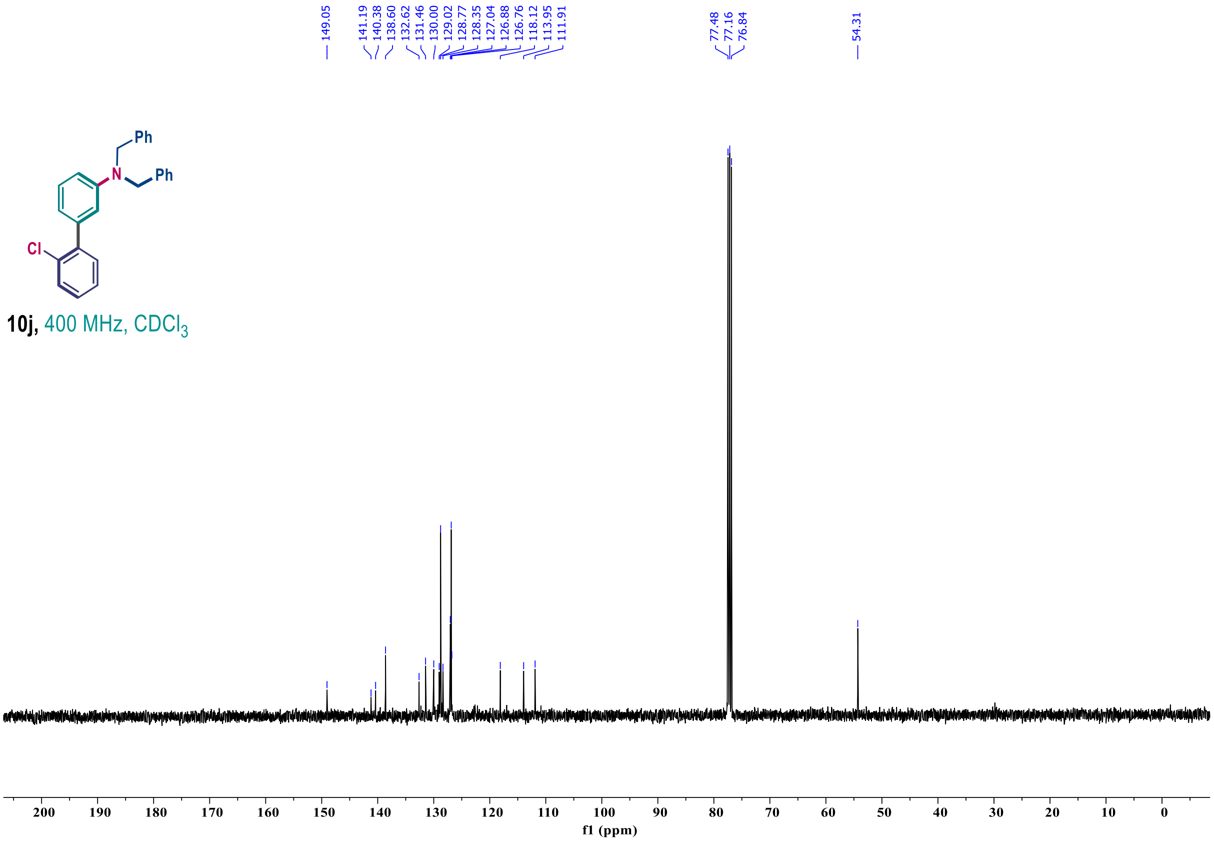

10j, 400 MHz, $CDCl_3$

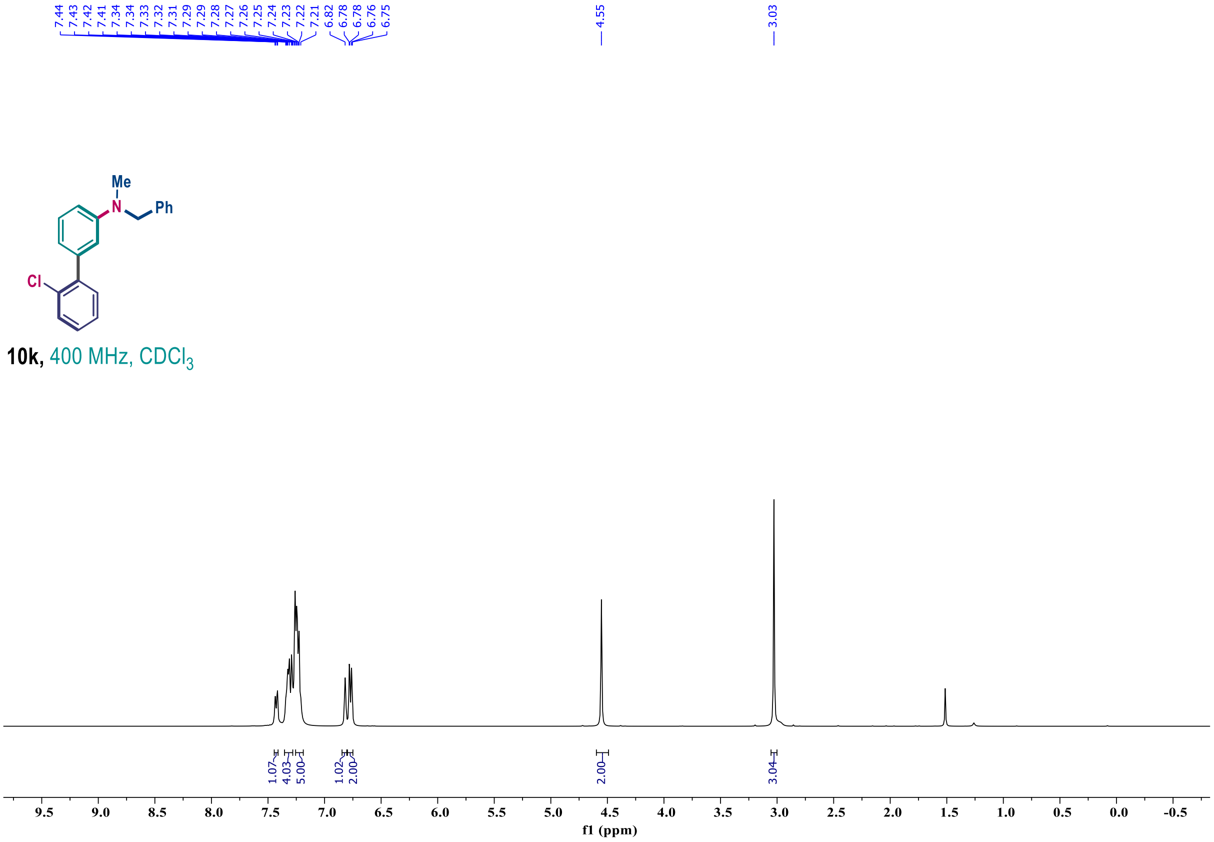

10k, 400 MHz, $CDCl_3$

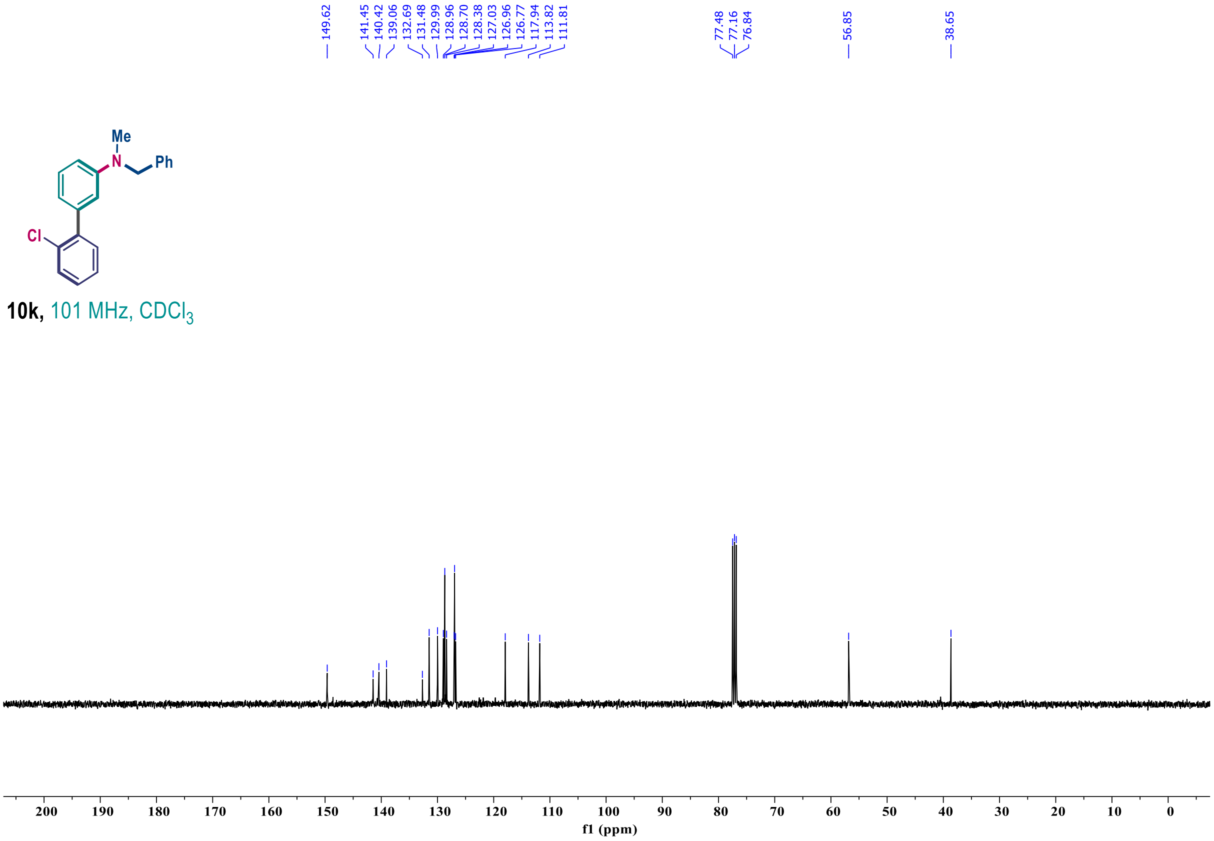

10k, 101 MHz, $CDCl_3$

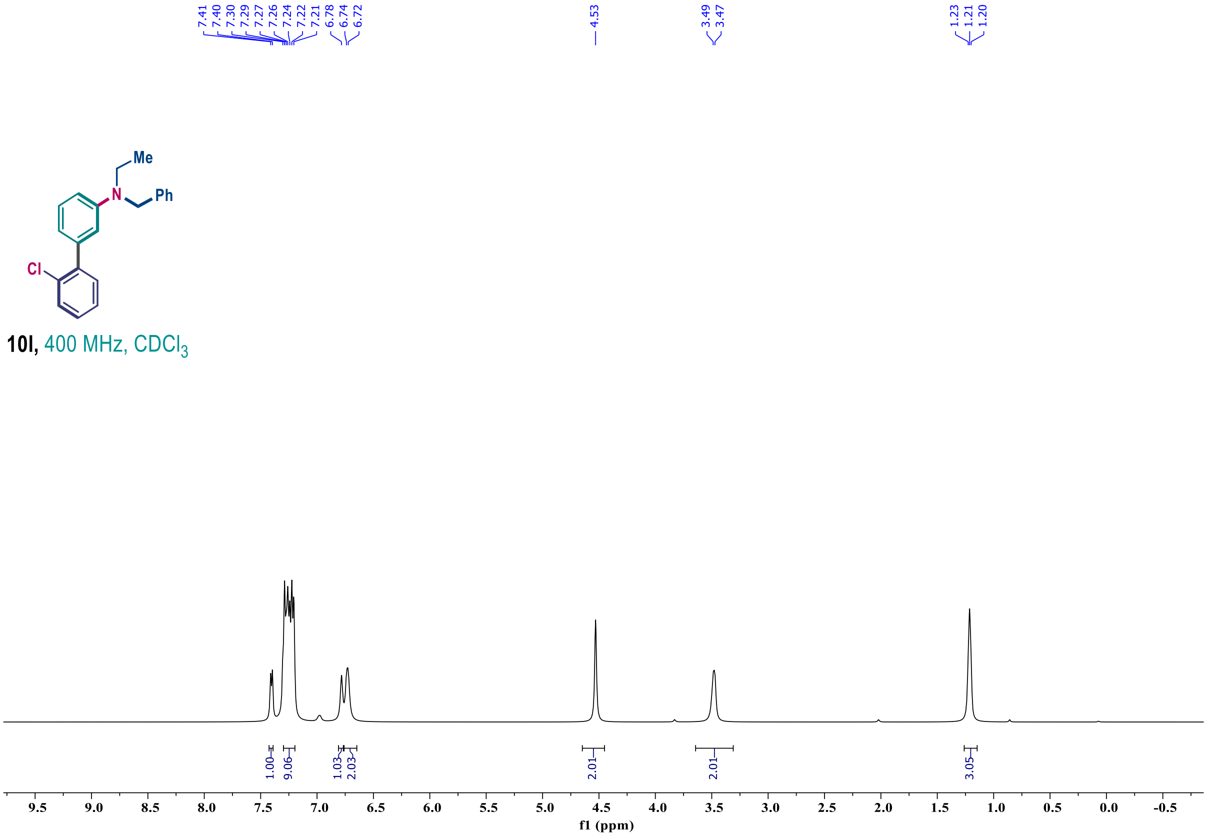
7.41 7.40 7.30 7.29 7.27 7.26 7.24 7.22 7.21 6.78 6.74 6.72
4.53
3.49 3.47
1.23 1.21 1.20
Me
N
Ph
Cl
10l, 400 MHz, CDCl3
1.00 9.06 1.03 2.03 2.01 2.01 3.05
9.5 9.0 8.5 8.0 7.5 7.0 6.5 6.0 5.5 5.0 4.5 4.0 3.5 3.0 2.5 2.0 1.5 1.0 0.5 0.0 -0.5
f1 (ppm)

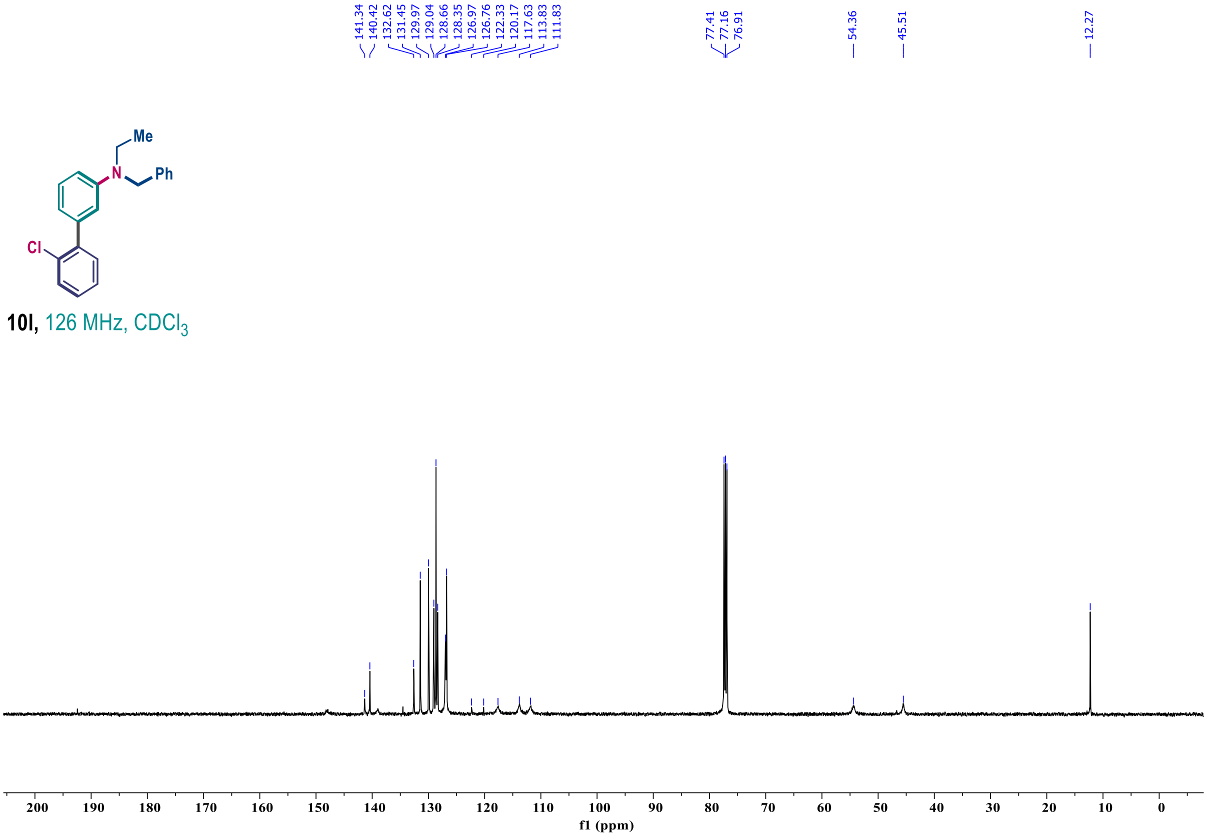
141.34 140.42 132.62 131.45 129.97 129.04 128.66 128.35 126.97 126.76 122.33 120.17 117.63 113.83 111.83
77.41 77.16 76.91
54.36
45.51
12.27
Me
N
Ph
Cl
10l, 126 MHz, CDCl3
200 190 180 170 160 150 140 130 120 110 100 90 80 70 60 50 40 30 20 10 0
f1 (ppm)

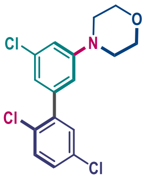

10m, 400 MHz, $CDCl_3$

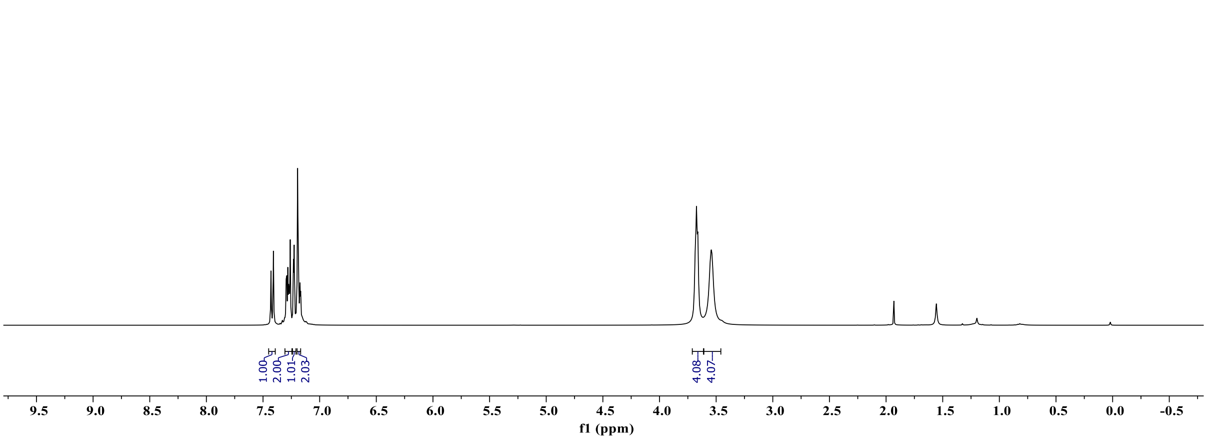

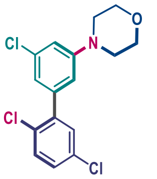

10m, 101 MHz, $CDCl_3$

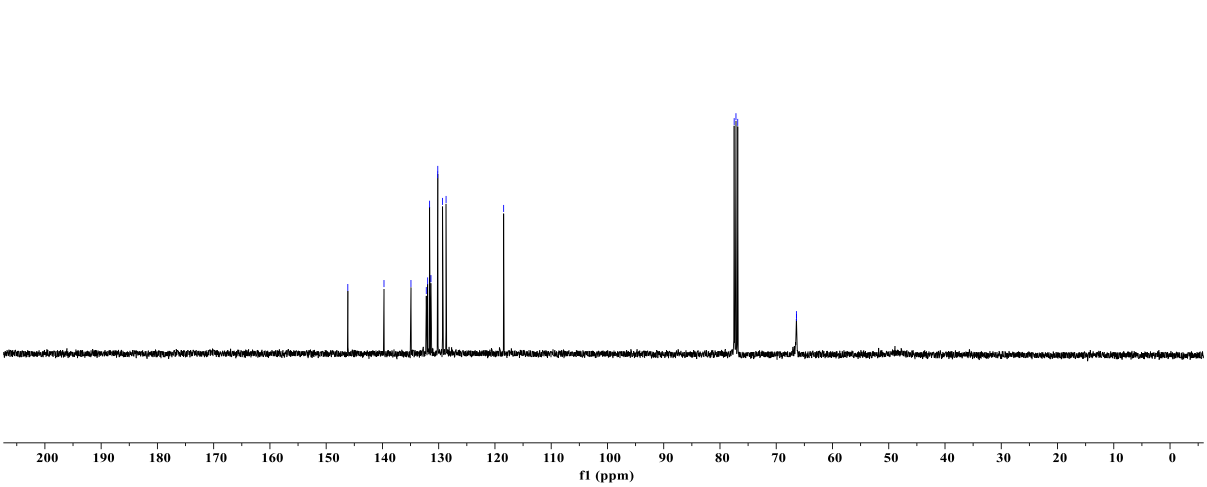

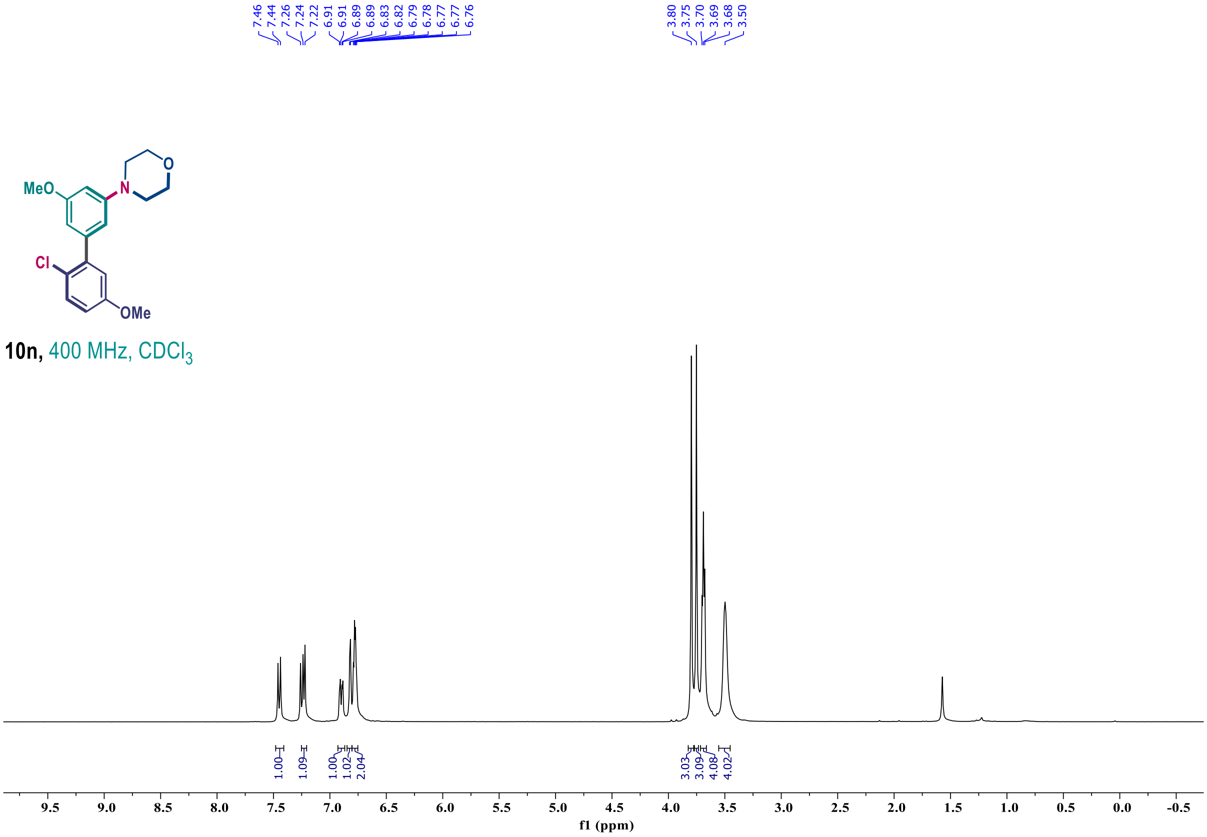

10n, 400 MHz, $CDCl_3$

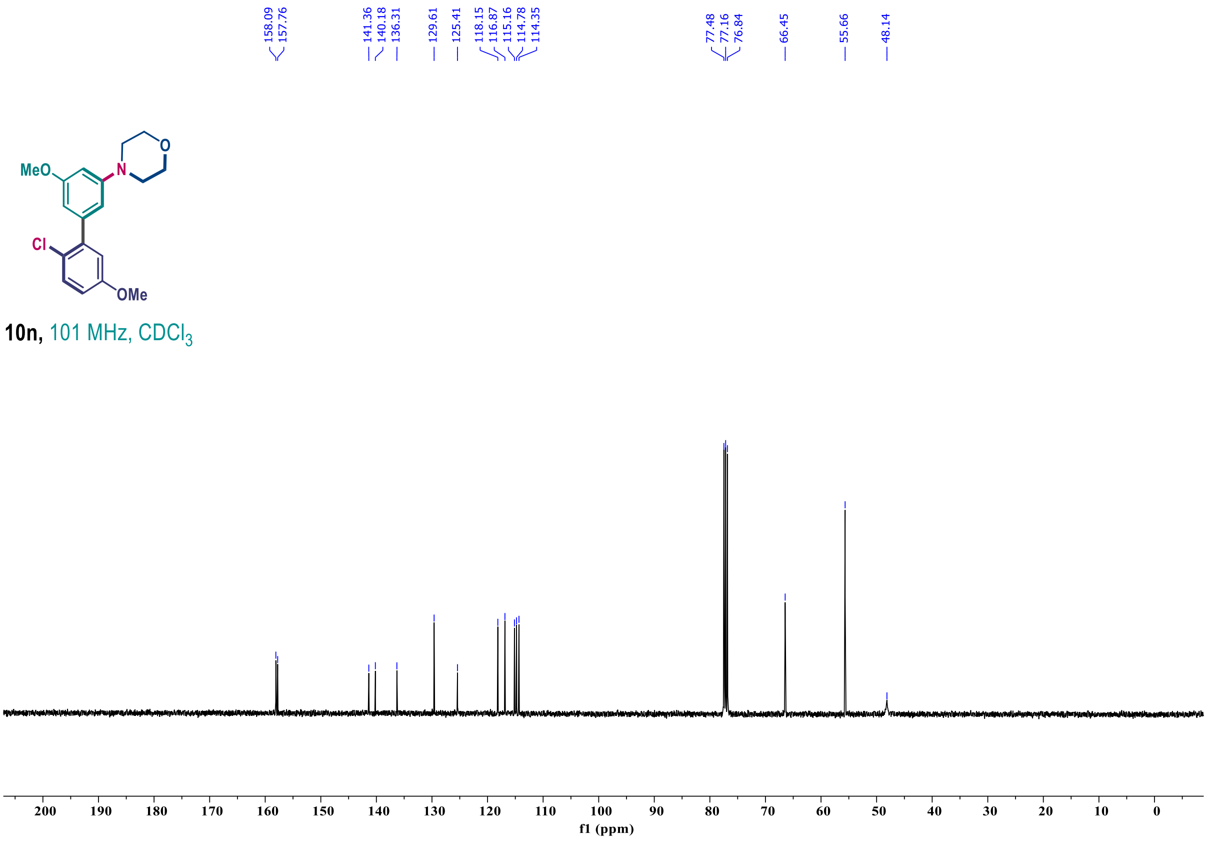

10n, 101 MHz, $CDCl_3$

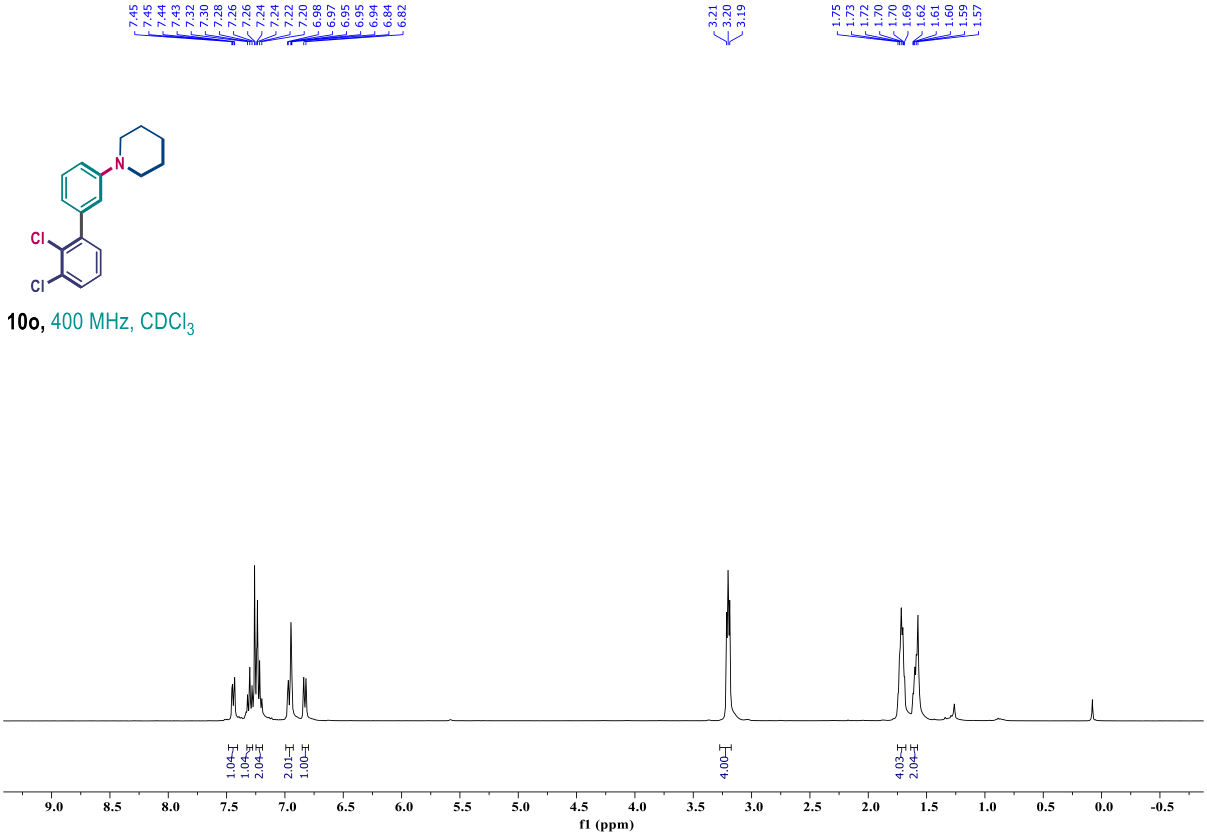

10o, 400 MHz, $CDCl_3$

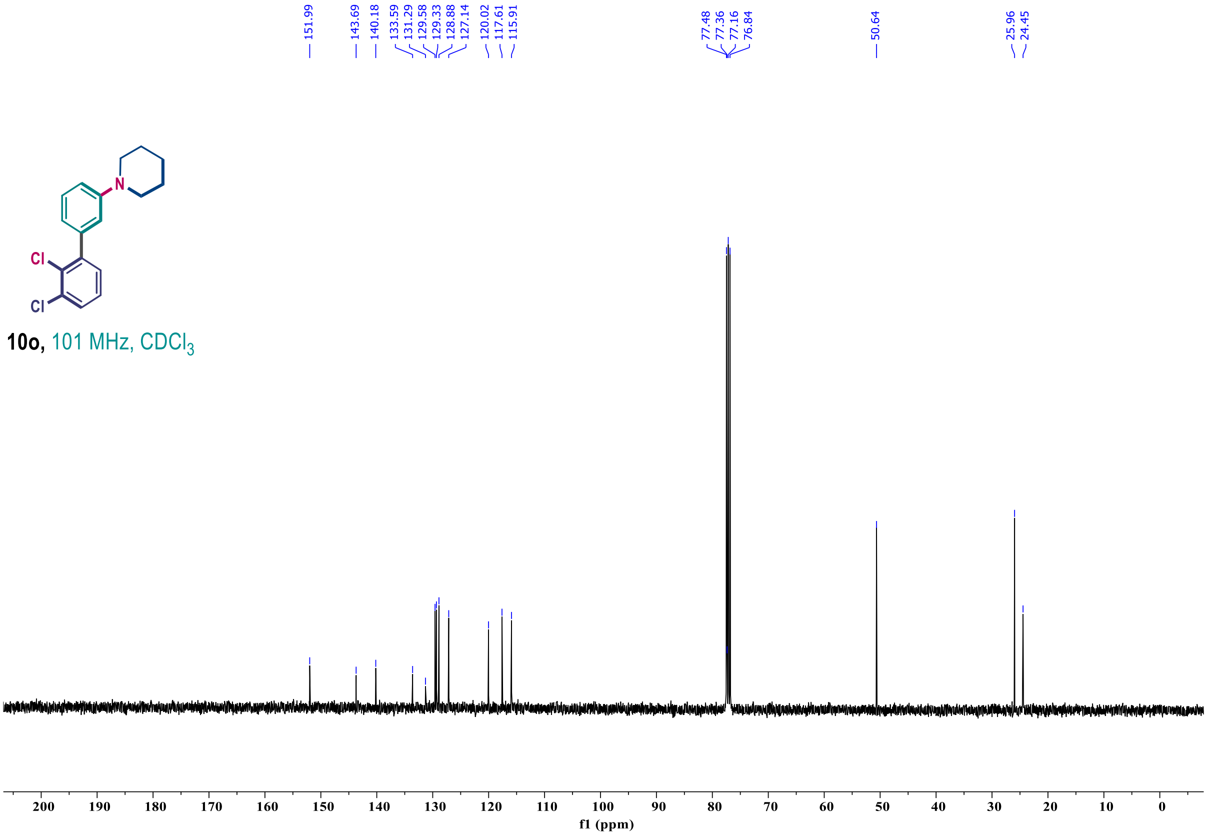

10o, 101 MHz, $CDCl_3$

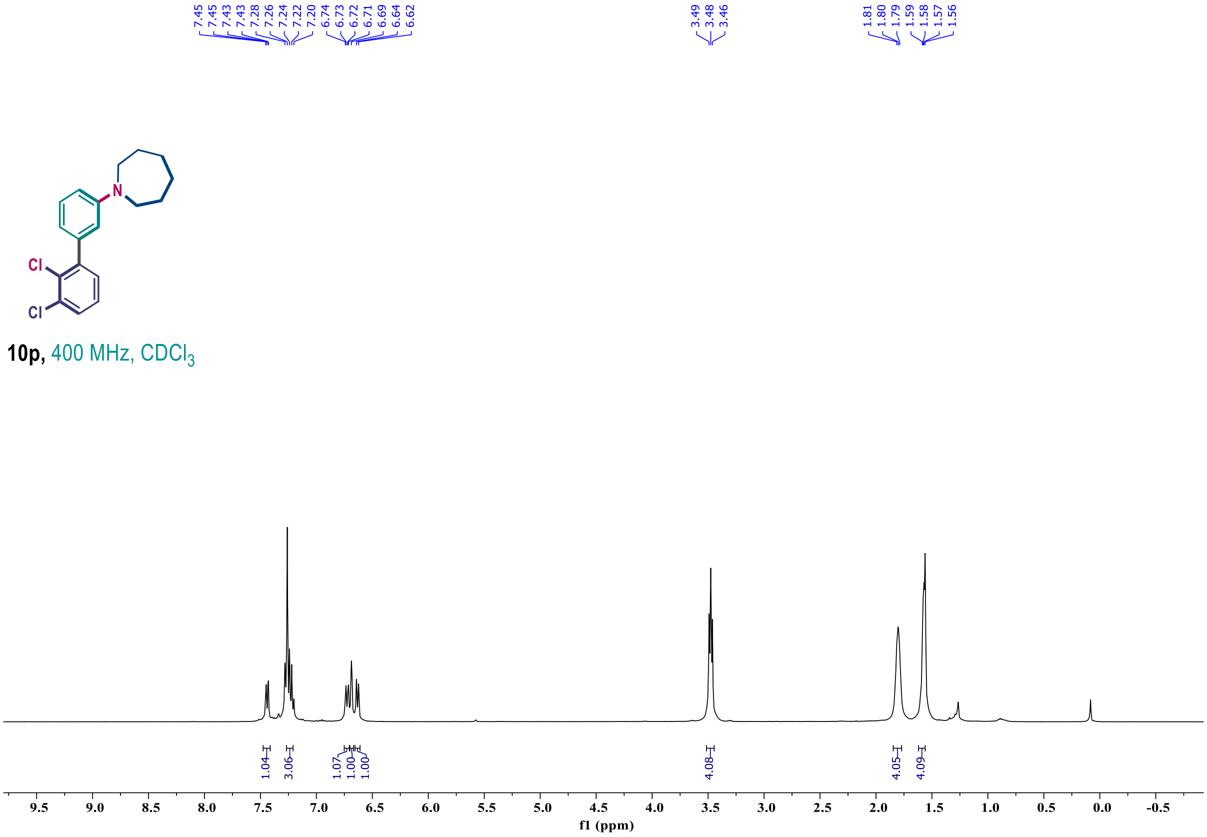
7.45 7.45 7.43 7.43 7.28 7.26 7.24 7.22 7.20 6.74 6.73 6.72 6.71 6.69 6.64 6.62
3.49 3.48 3.46
1.81 1.80 1.79 1.59 1.58 1.57 1.56
Cl
Cl
N
10p, 400 MHz, CDCl3
1.04 3.06 1.07 1.00 1.00 4.08 4.05 4.09
9.5 9.0 8.5 8.0 7.5 7.0 6.5 6.0 5.5 5.0 4.5 4.0 3.5 3.0 2.5 2.0 1.5 1.0 0.5 0.0 -0.5
f1 (ppm)

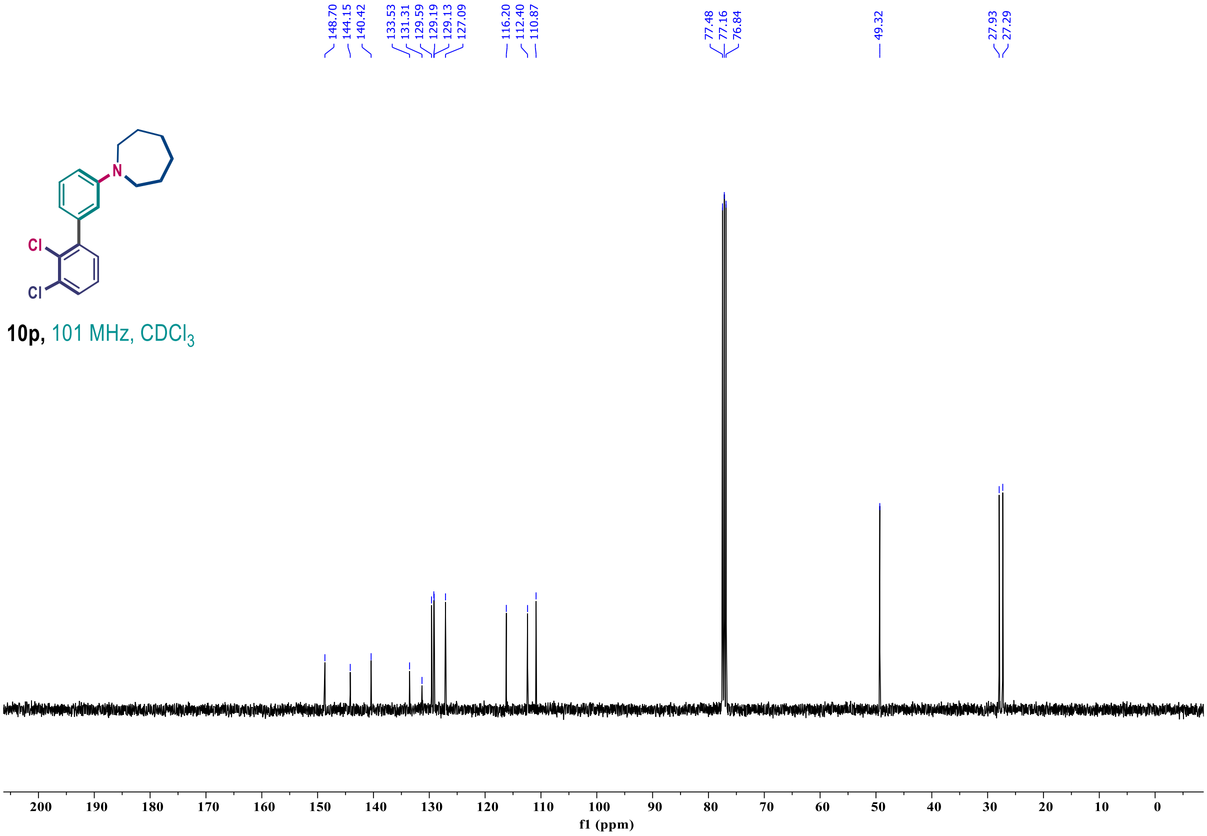
148.70 144.15 140.42 133.53 131.31 129.59 129.19 129.13 127.09 116.20 112.40 110.87
77.48 77.16 76.84
49.32
27.93 27.29
Cl
Cl
N
10p, 101 MHz, CDCl3
200 190 180 170 160 150 140 130 120 110 100 90 80 70 60 50 40 30 20 10 0
f1 (ppm)

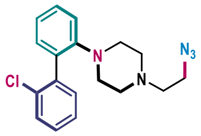

11a, 400 MHz, $CDCl_3$

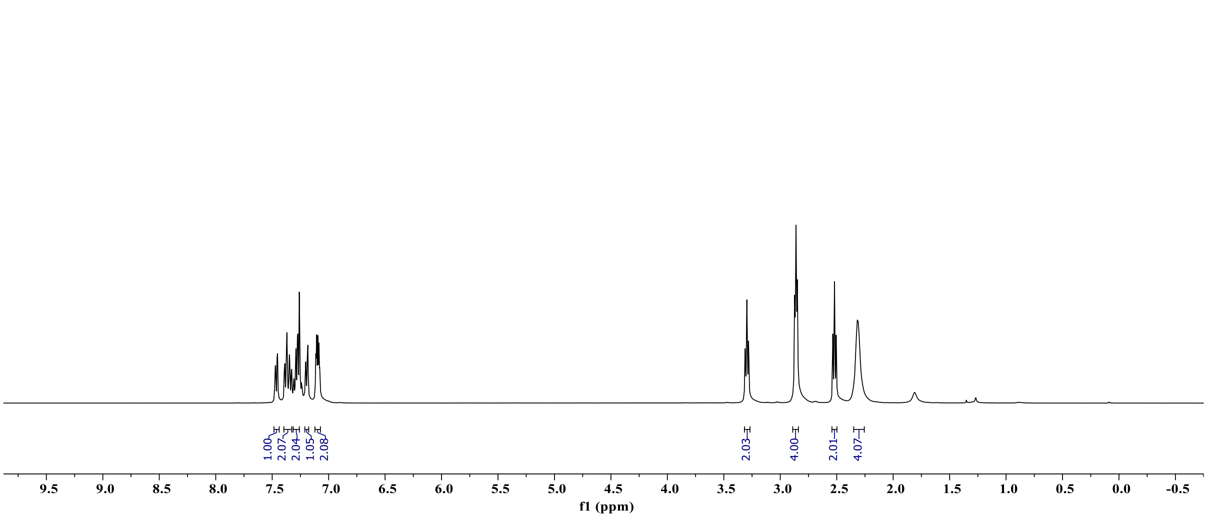

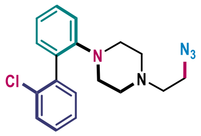

11a, 101 MHz, $CDCl_3$

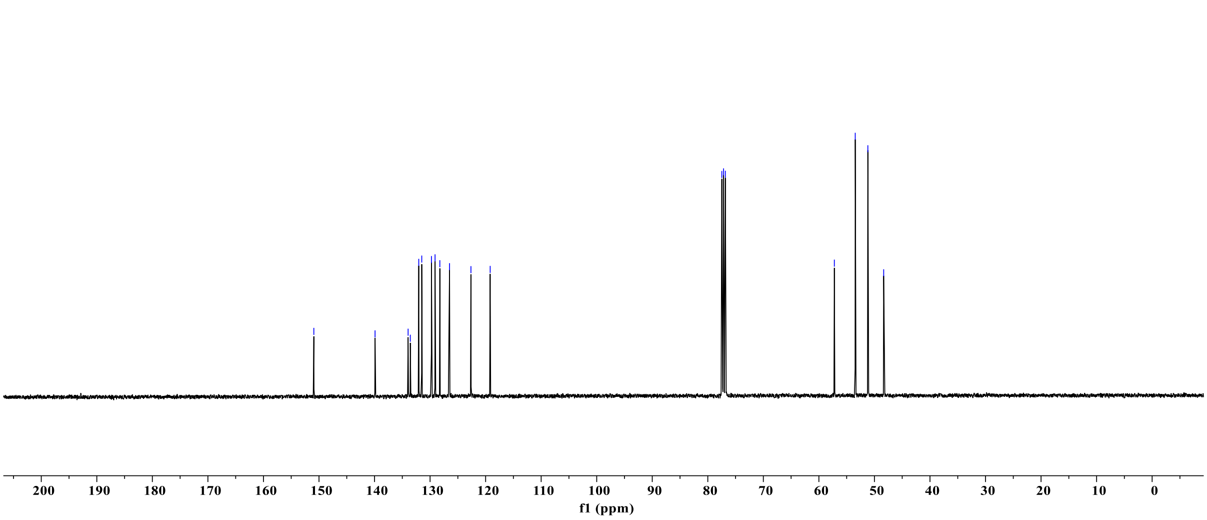

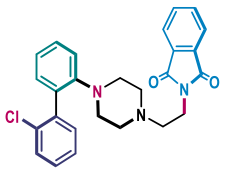

11b, 400 MHz, CDCl$_3$

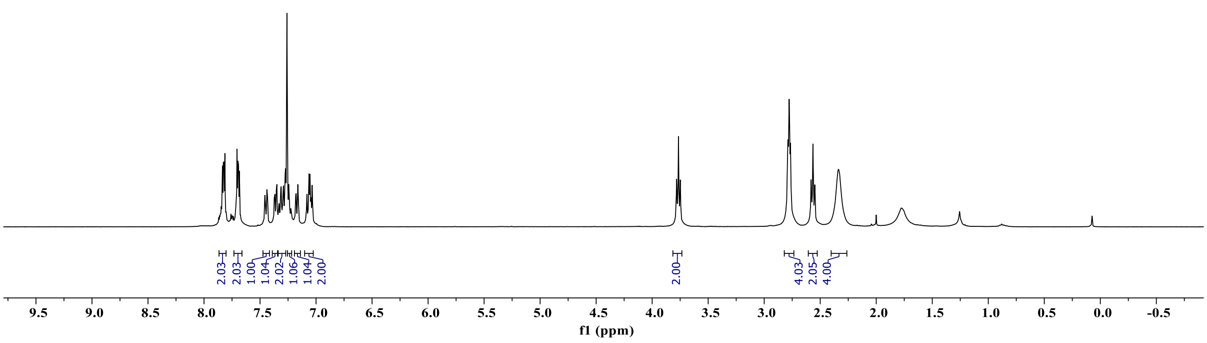

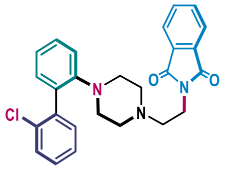

11b, 101 MHz, CDCl$_3$

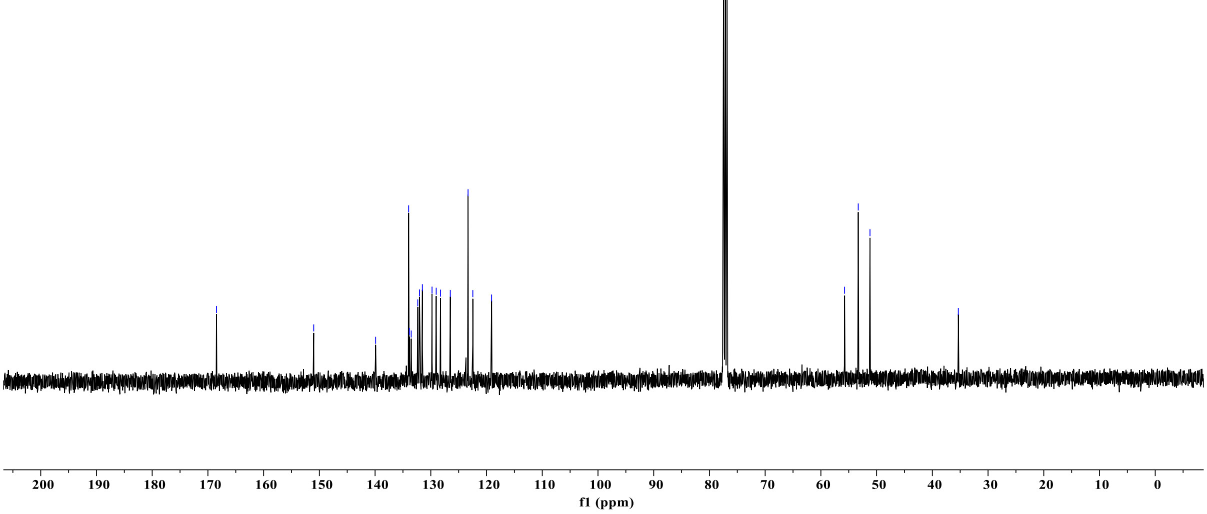

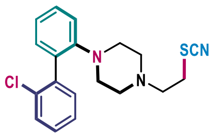

11c, 400 MHz, $CDCl_3$

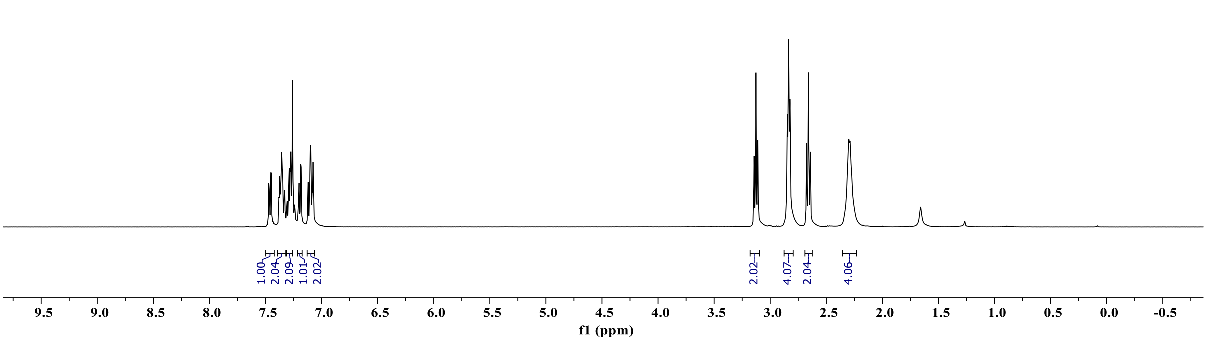

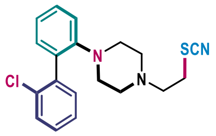

11c, 101 MHz, $CDCl_3$

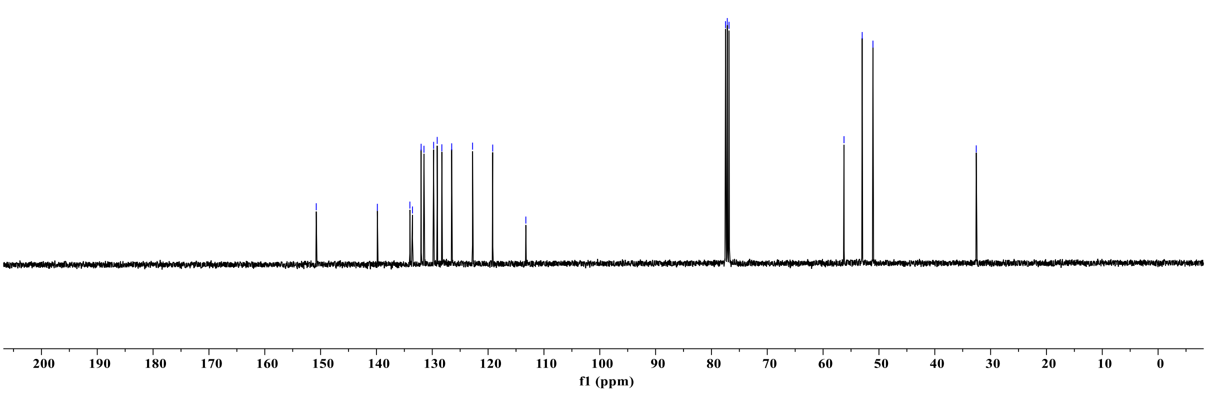

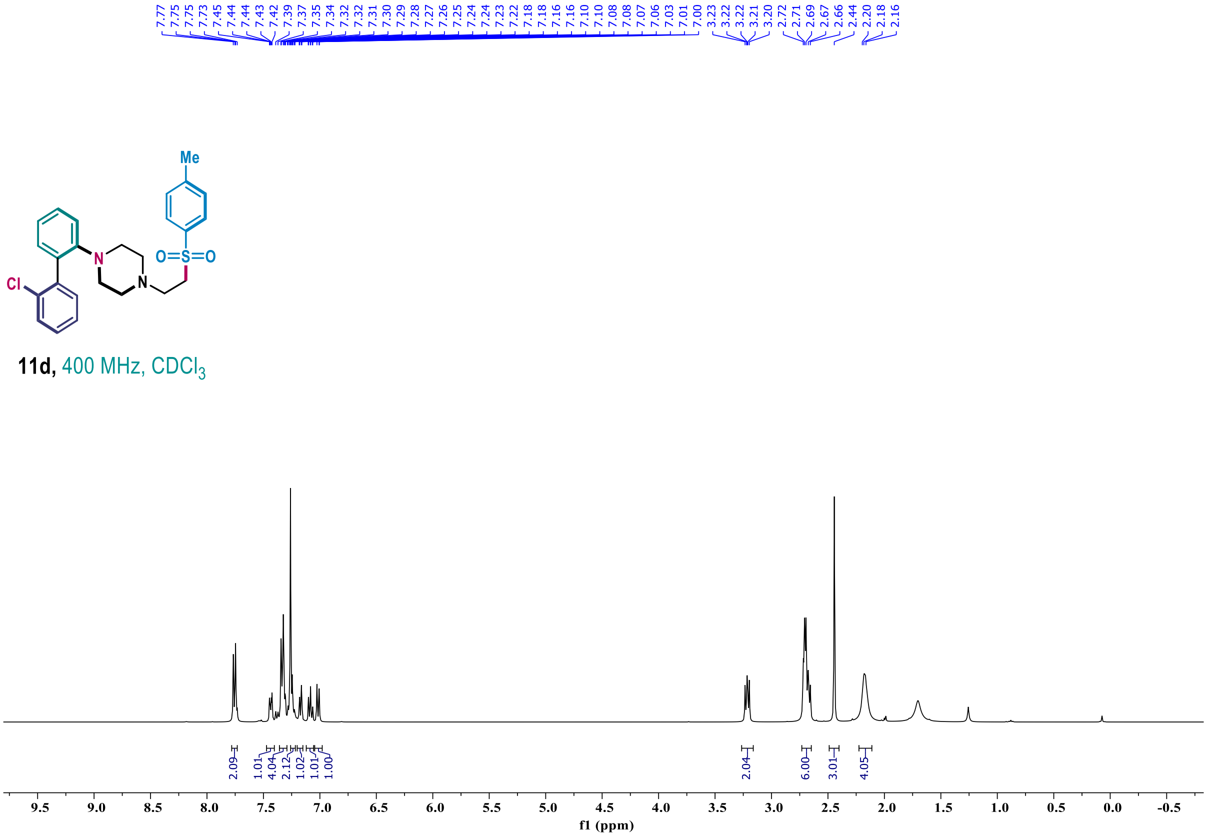

11d, 400 MHz, $CDCl_3$

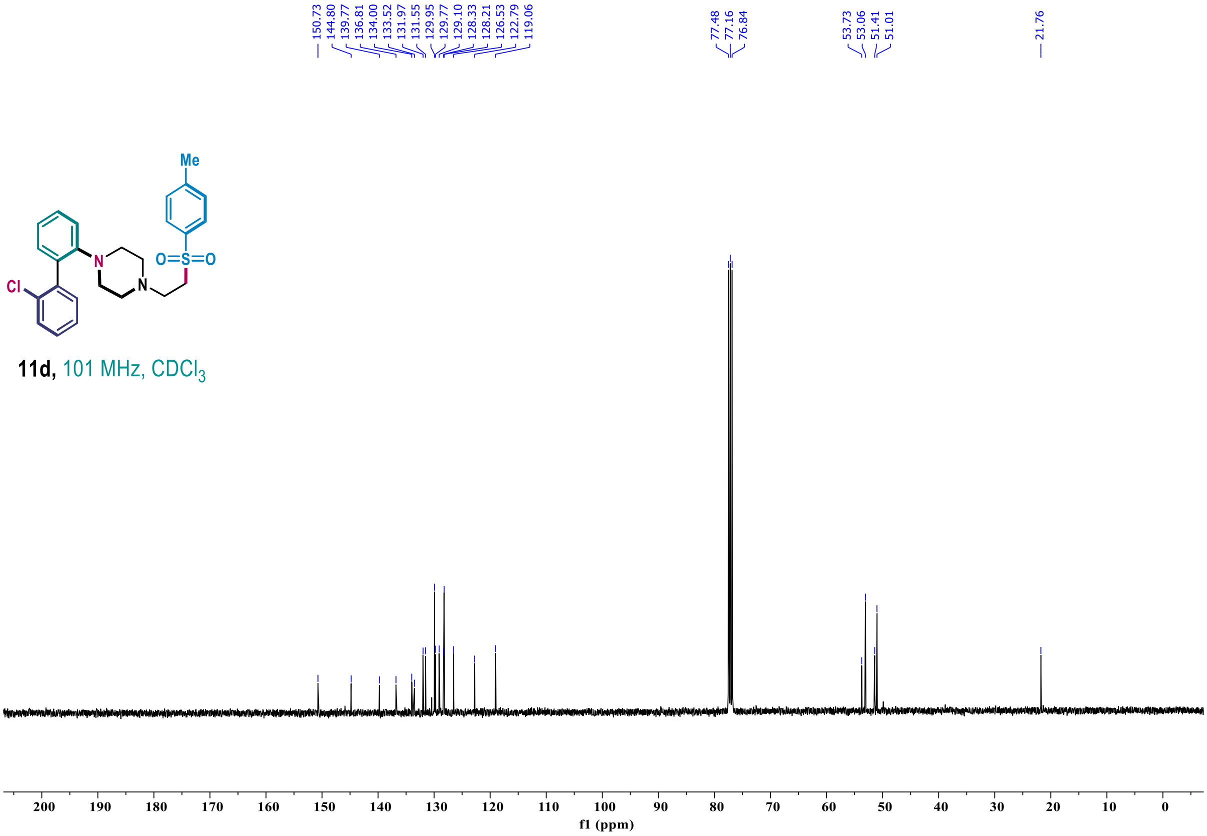

11d, 101 MHz, $CDCl_3$

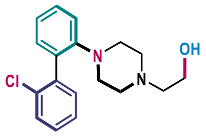

11e, 400 MHz, $CDCl_3$

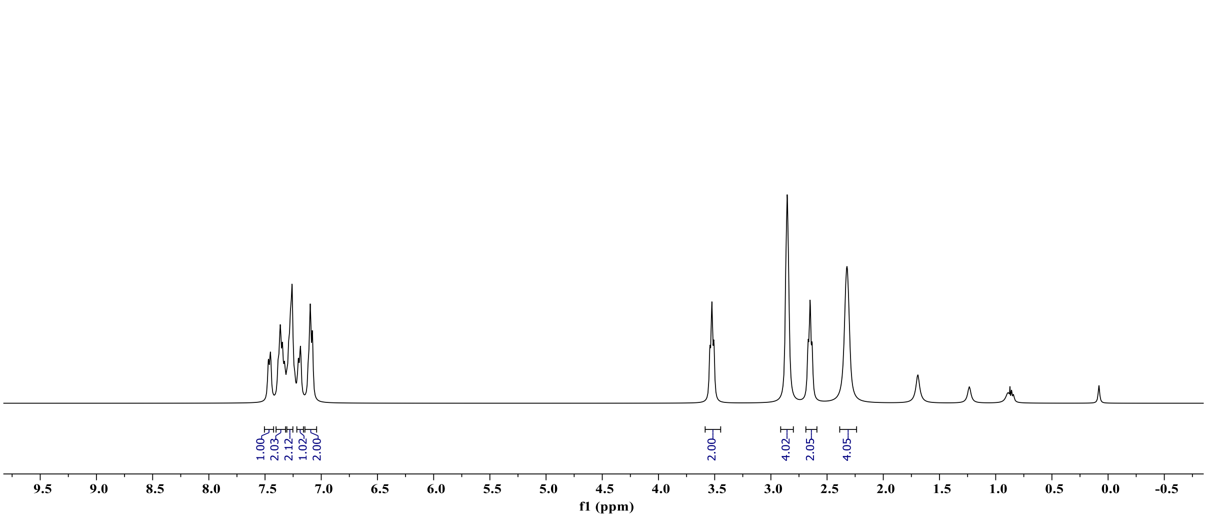

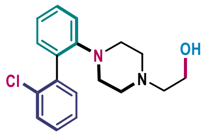

11e, 101 MHz, $CDCl_3$

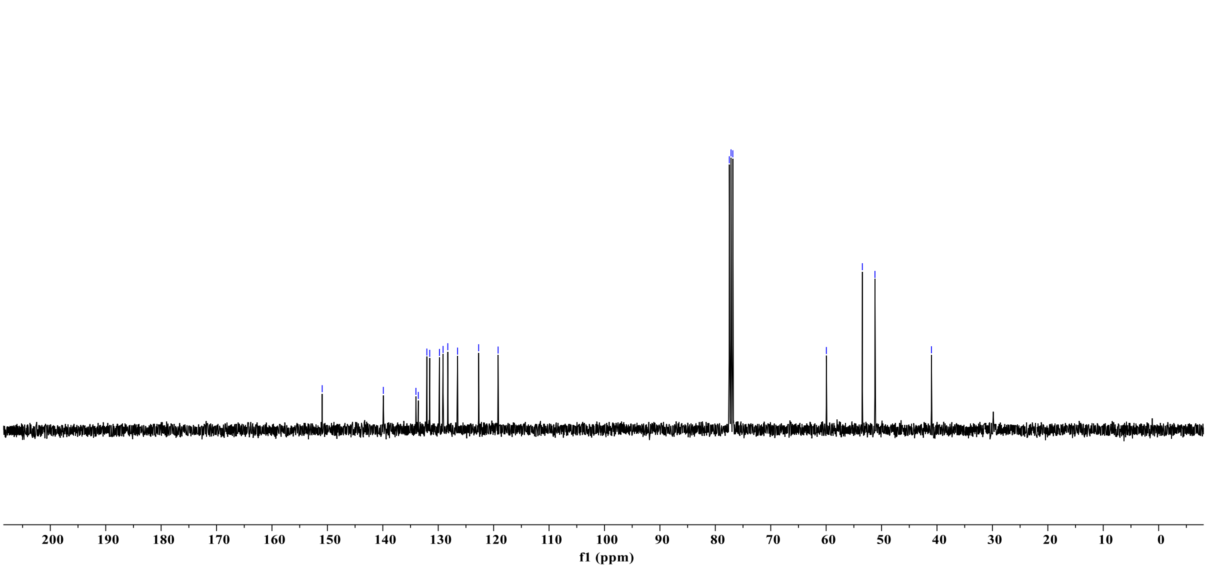

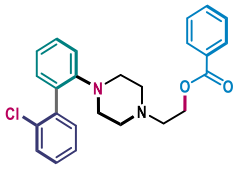

11f, 400 MHz, $CDCl_3$

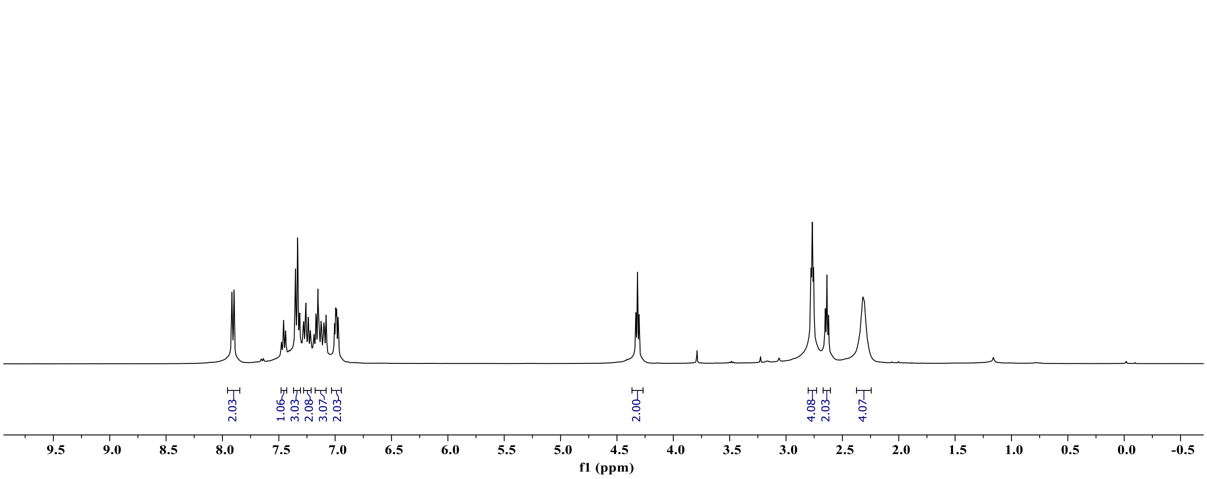

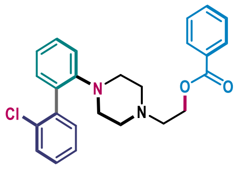

11f, 101 MHz, $CDCl_3$

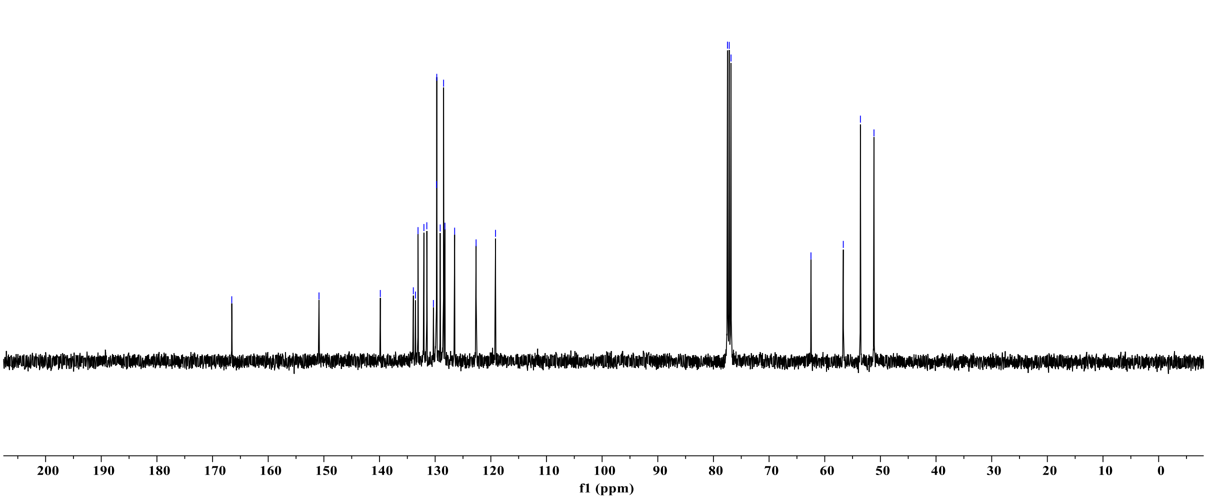

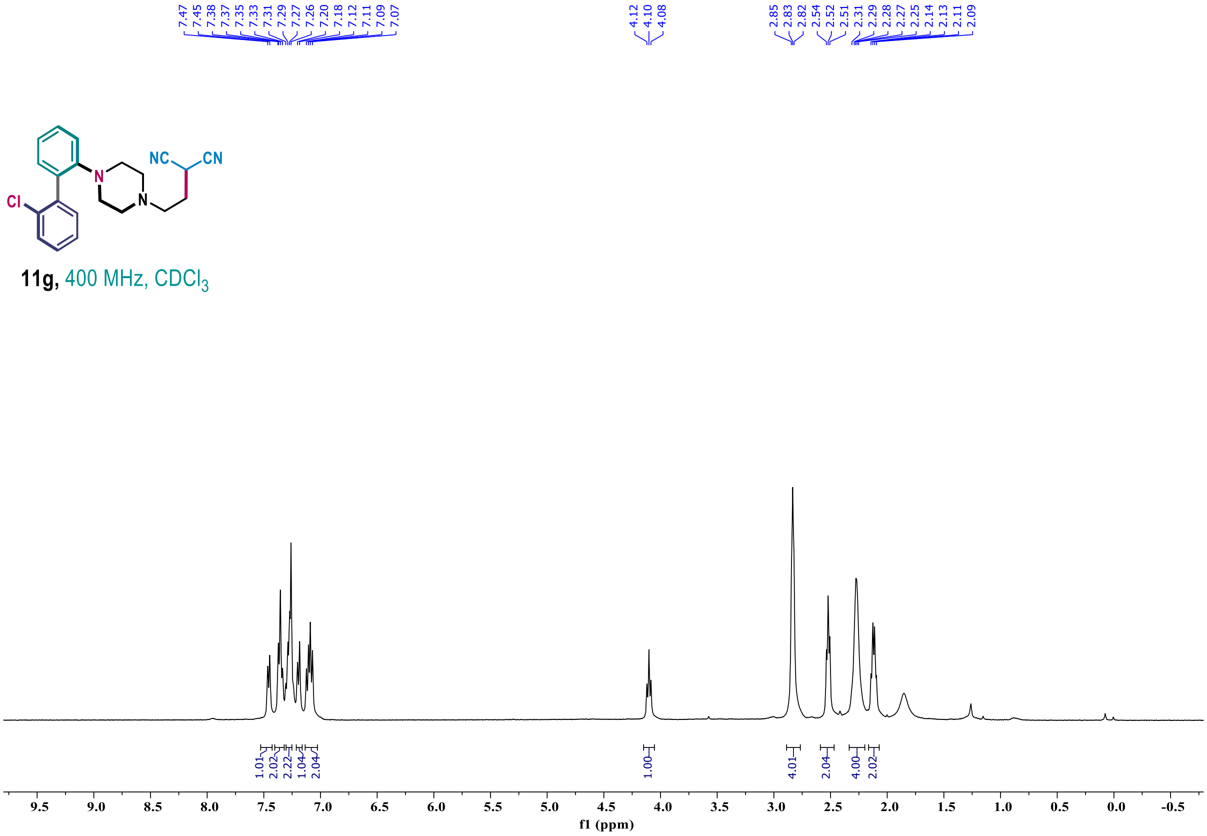

11g, 400 MHz, CDCl3
f1 (ppm)

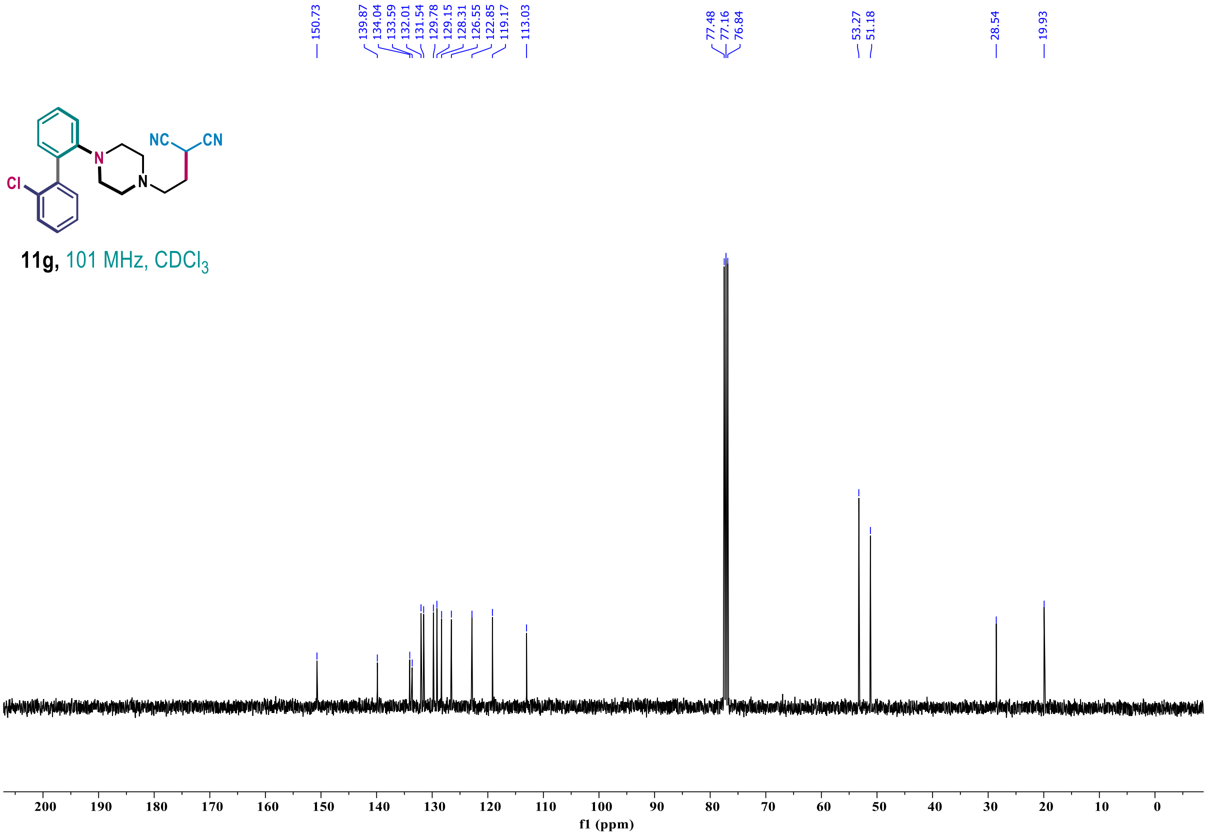

11g, 101 MHz, CDCl3
f1 (ppm)

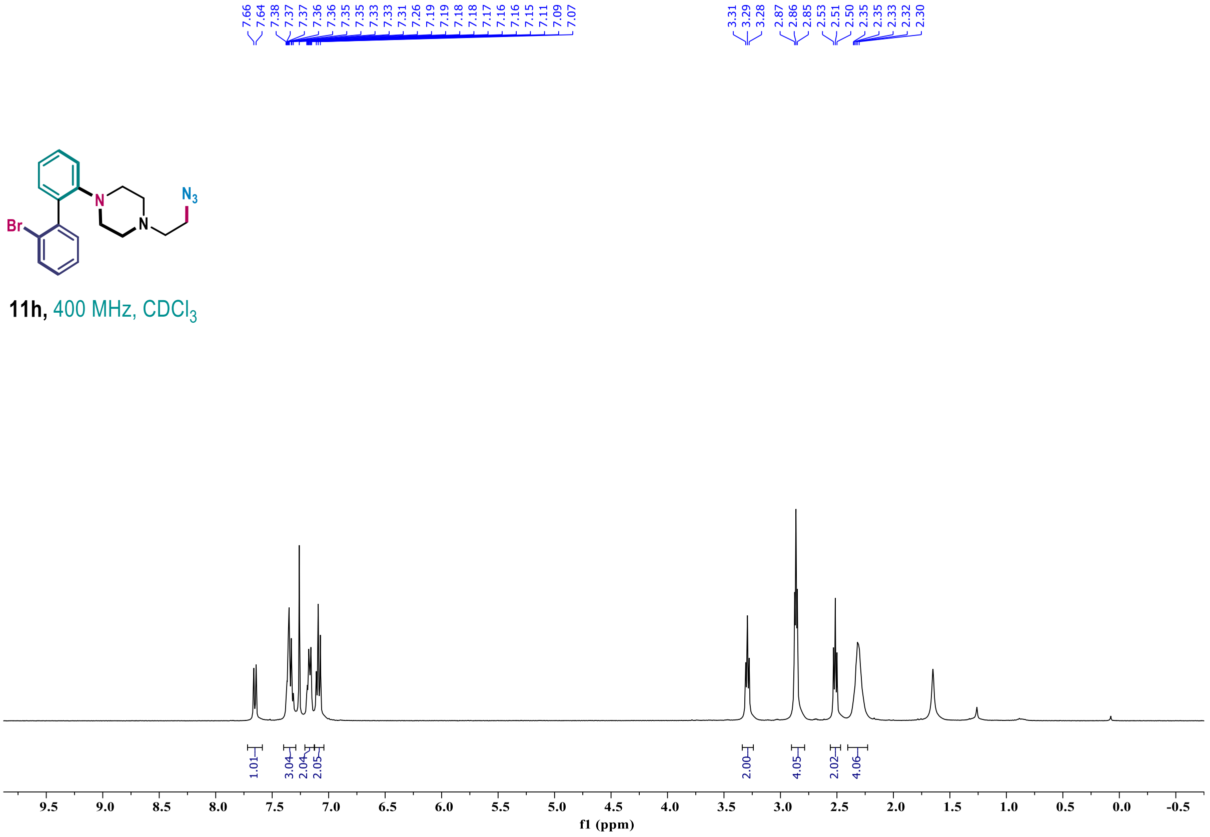

11h, 400 MHz, $CDCl_3$

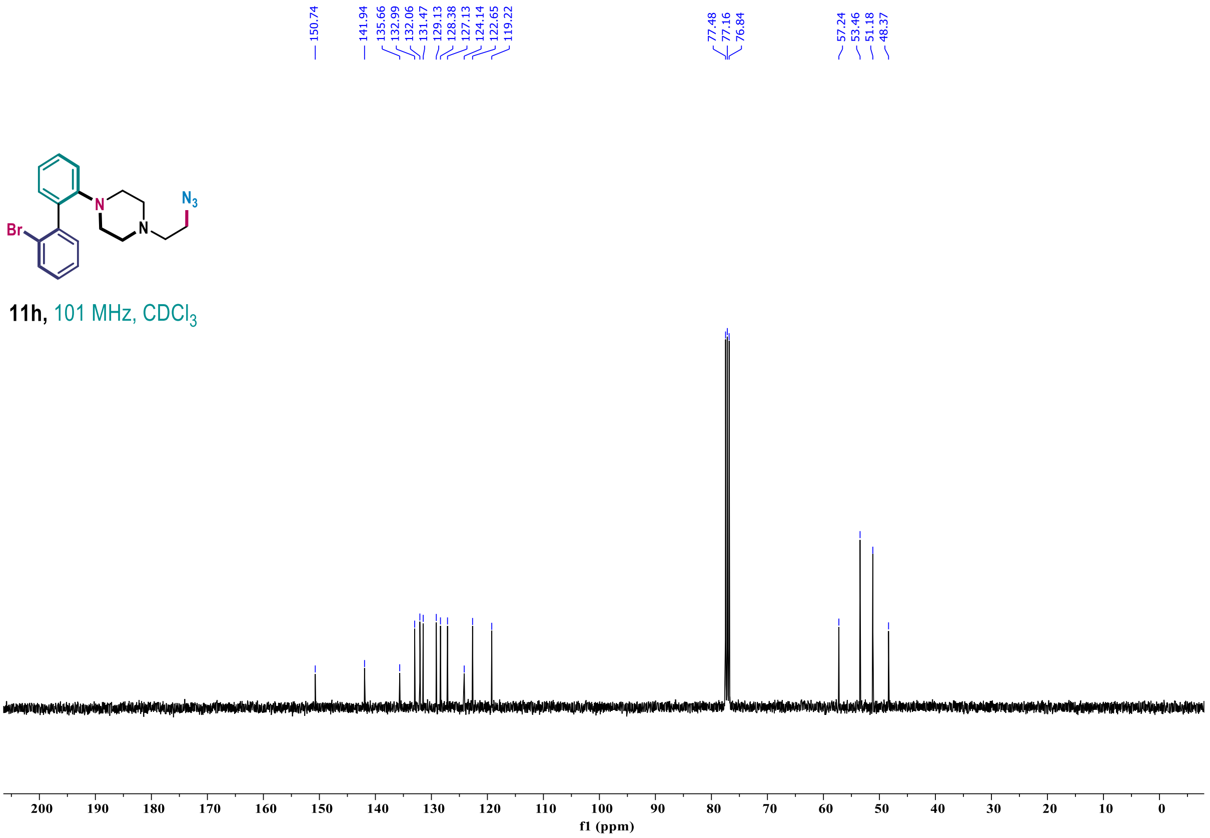

11h, 101 MHz, $CDCl_3$

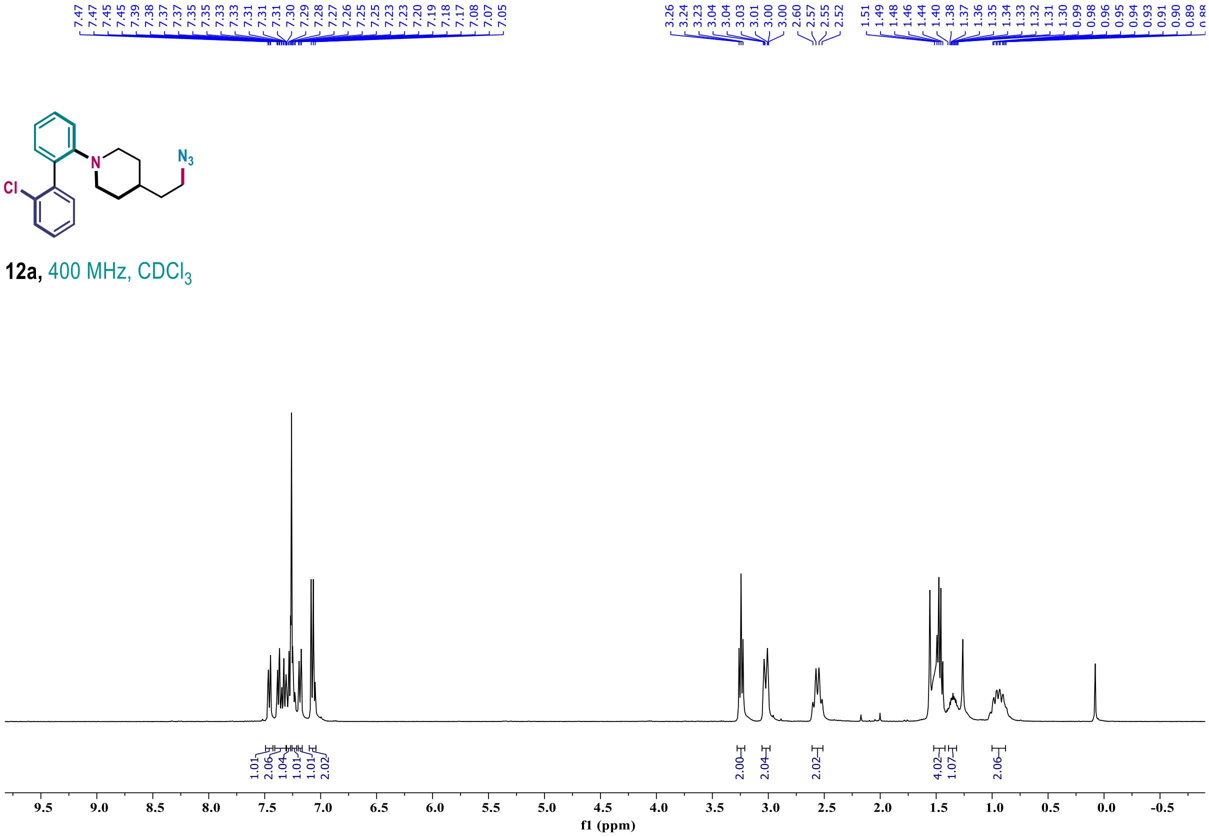
7.47 7.47 7.45 7.45 7.39 7.38 7.37 7.37 7.35 7.35 7.33 7.33 7.31 7.31 7.30 7.29 7.28 7.27 7.26 7.25 7.25 7.23 7.23 7.20 7.19 7.18 7.17 7.08 7.07 7.05
3.26 3.24 3.23 3.04 3.04 3.03 3.01 3.00 3.00 2.60 2.57 2.55 2.52 1.51 1.49 1.48 1.46 1.44 1.40 1.38 1.37 1.36 1.35 1.34 1.33 1.32 1.31 1.30 0.99 0.98 0.96 0.95 0.94 0.93 0.91 0.90 0.89 [illegible]
Cl
N
N_3
12a, 400 MHz, $CDCl_3$
1.01 2.06 1.04 1.01 1.01 2.02 2.00 2.04 2.02 4.02 1.07 2.06
9.5 9.0 8.5 8.0 7.5 7.0 6.5 6.0 5.5 5.0 4.5 4.0 3.5 3.0 2.5 2.0 1.5 1.0 0.5 0.0 -0.5
f1 (ppm)

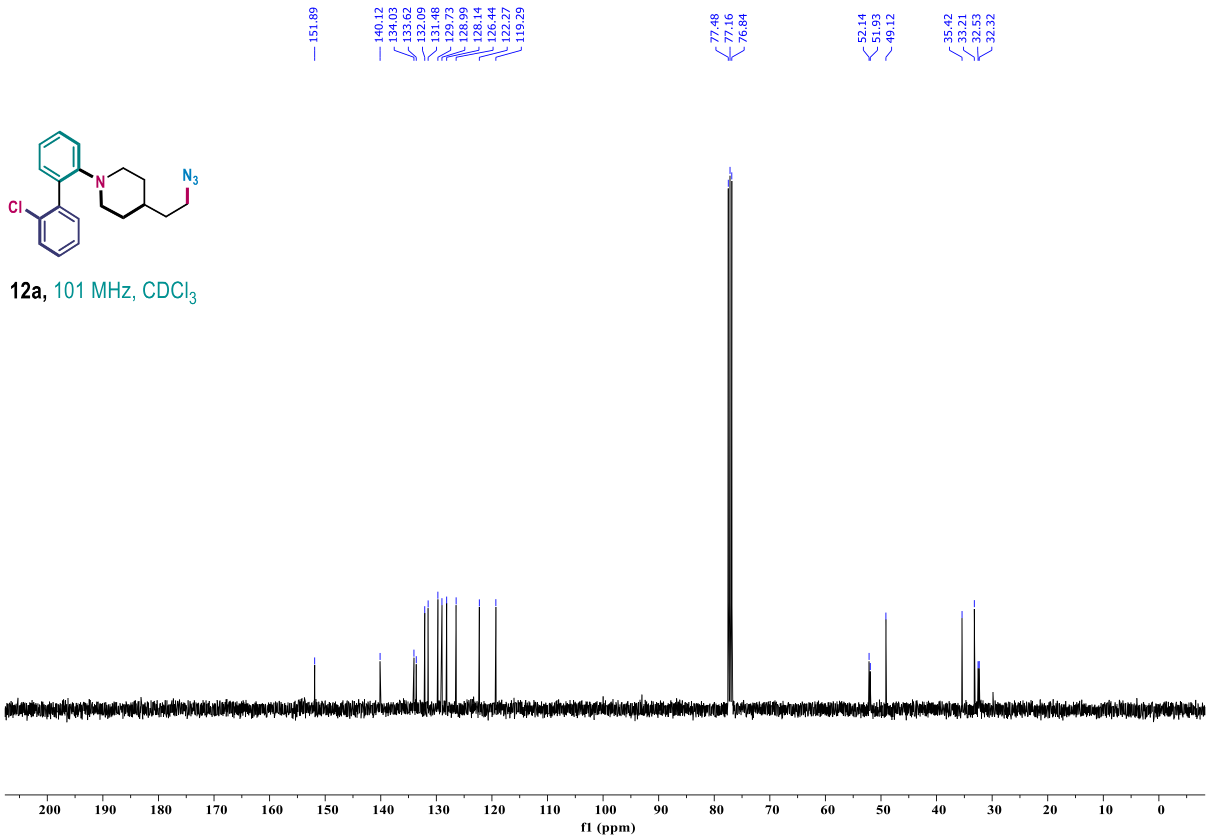
151.89
140.12 134.03 133.62 132.09 131.48 129.73 128.99 128.14 126.44 122.27 119.29
77.48 77.16 76.84
52.14 51.93 49.12
35.42 33.21 32.53 32.32
Cl
N
N_3
12a, 101 MHz, $CDCl_3$
200 190 180 170 160 150 140 130 120 110 100 90 80 70 60 50 40 30 20 10 0
f1 (ppm)

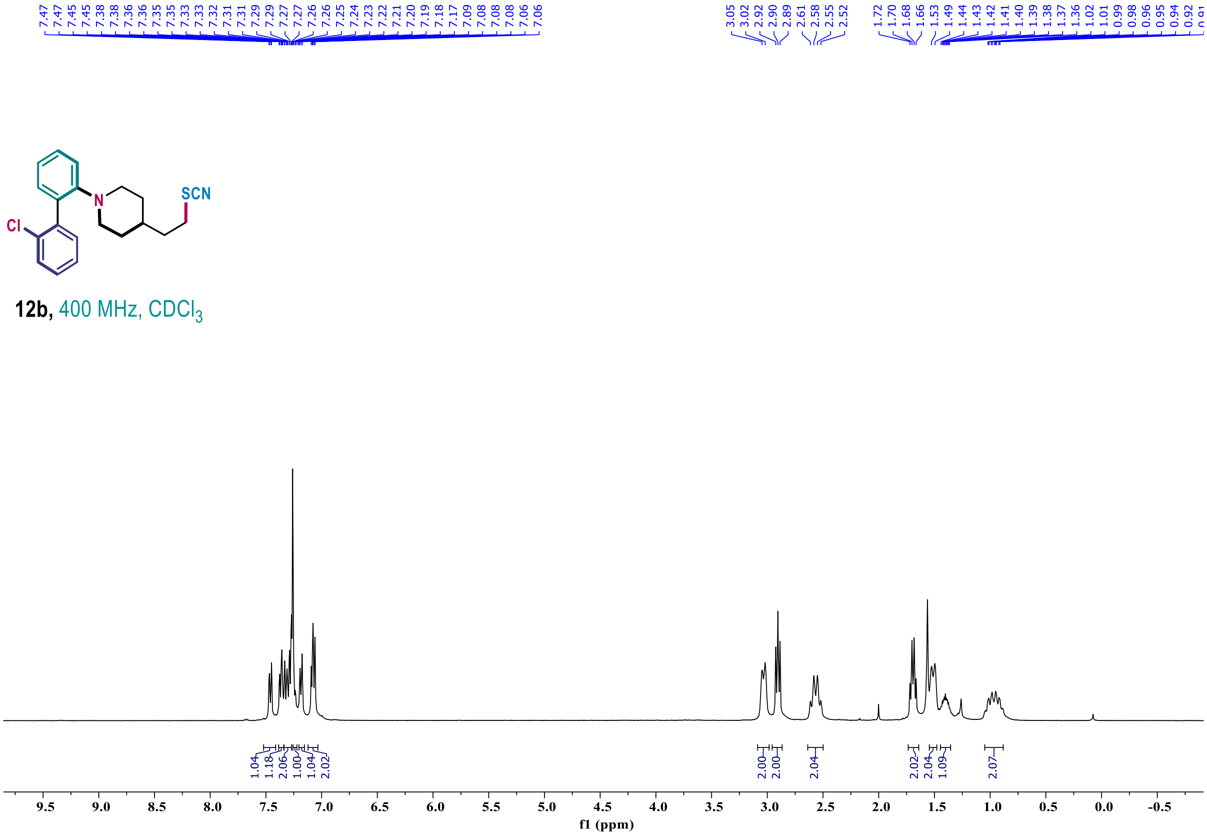

SCN
N
Cl
12b, 400 MHz, $CDCl_3$
f1 (ppm)

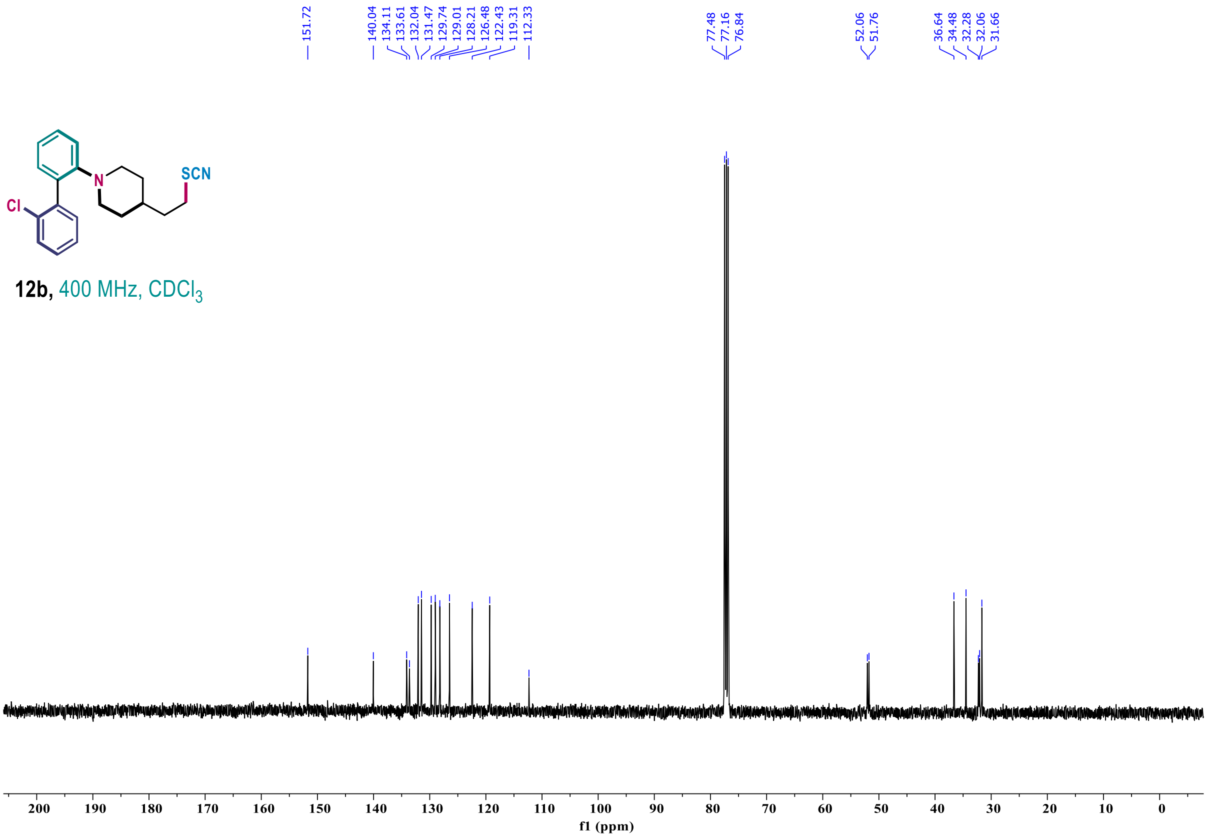

151.72
140.04
134.11
133.61
132.04
131.47
129.74
129.01
128.21
126.48
122.43
119.31
112.33
77.48
77.16
76.84
52.06
51.76
36.64
34.48
32.28
32.06
31.66
SCN
N
Cl
12b, 400 MHz, $CDCl_3$
f1 (ppm)

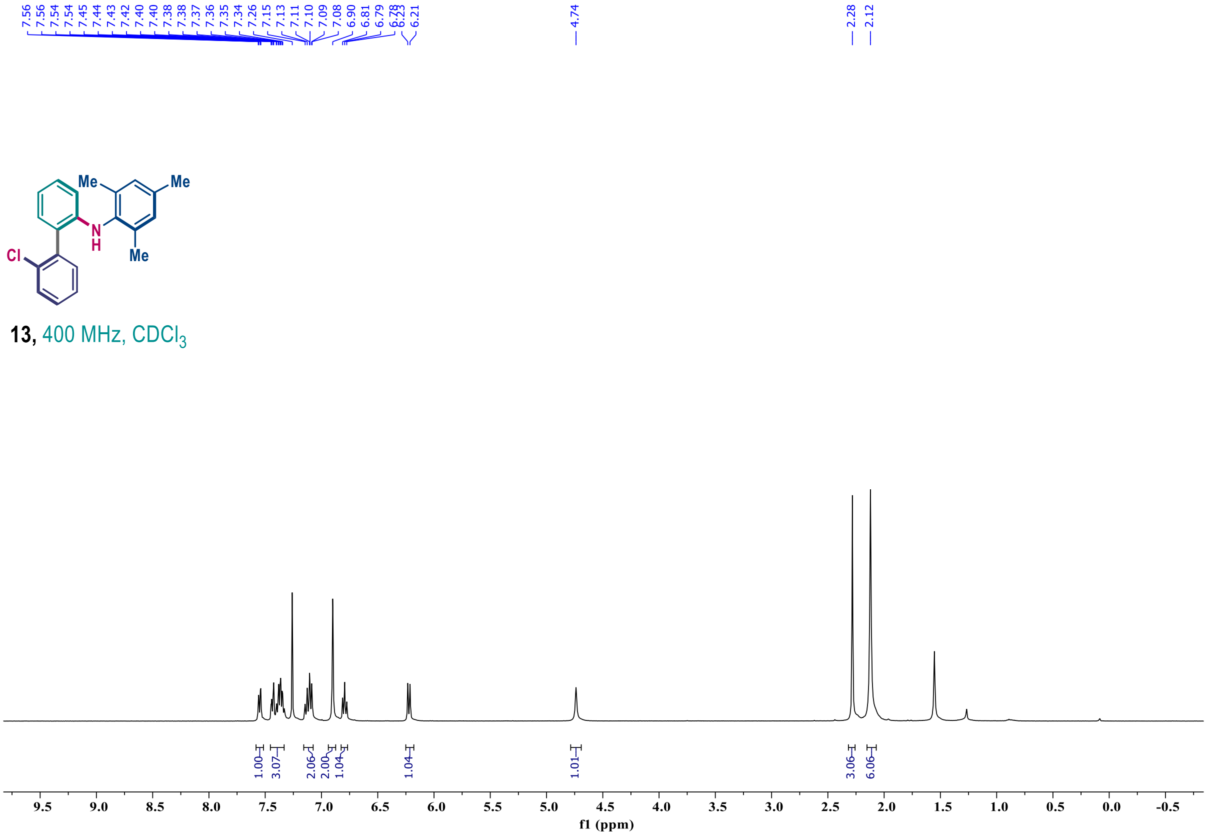

13, 400 MHz, $CDCl_3$

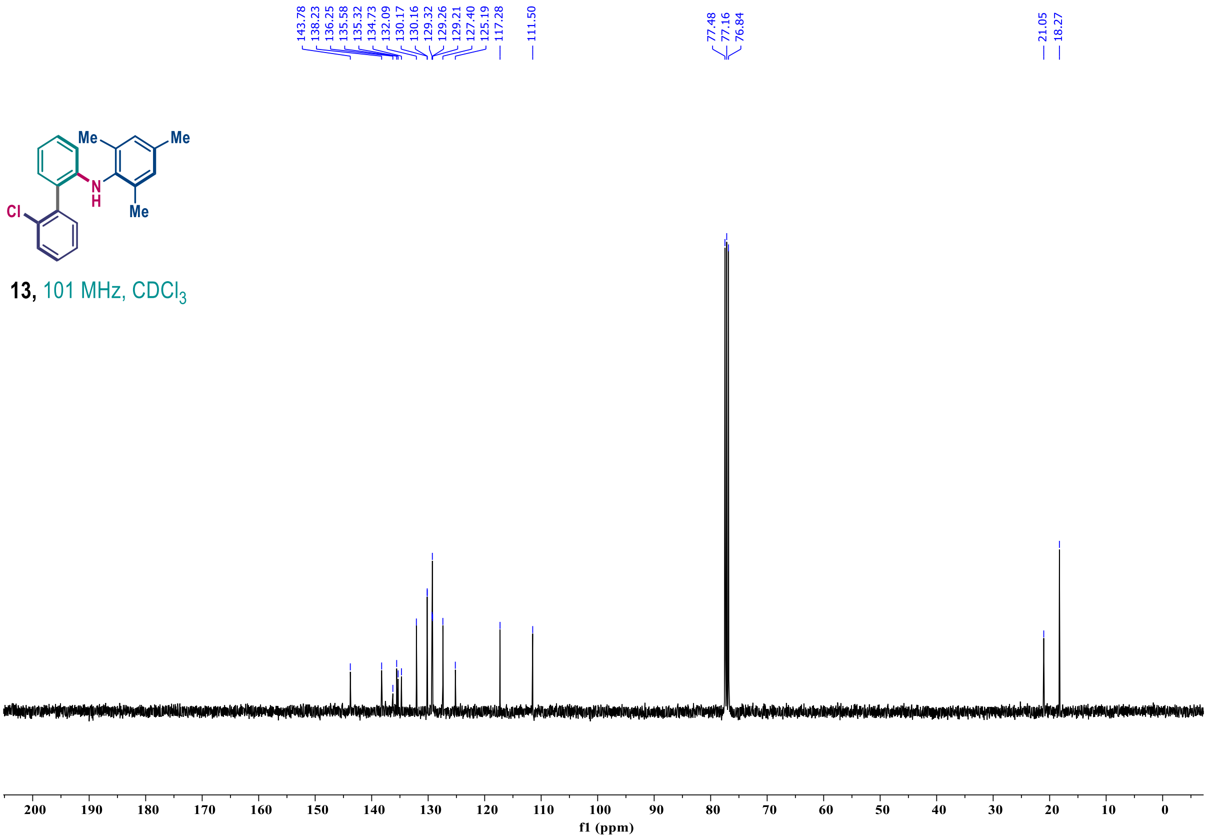

13, 101 MHz, $CDCl_3$

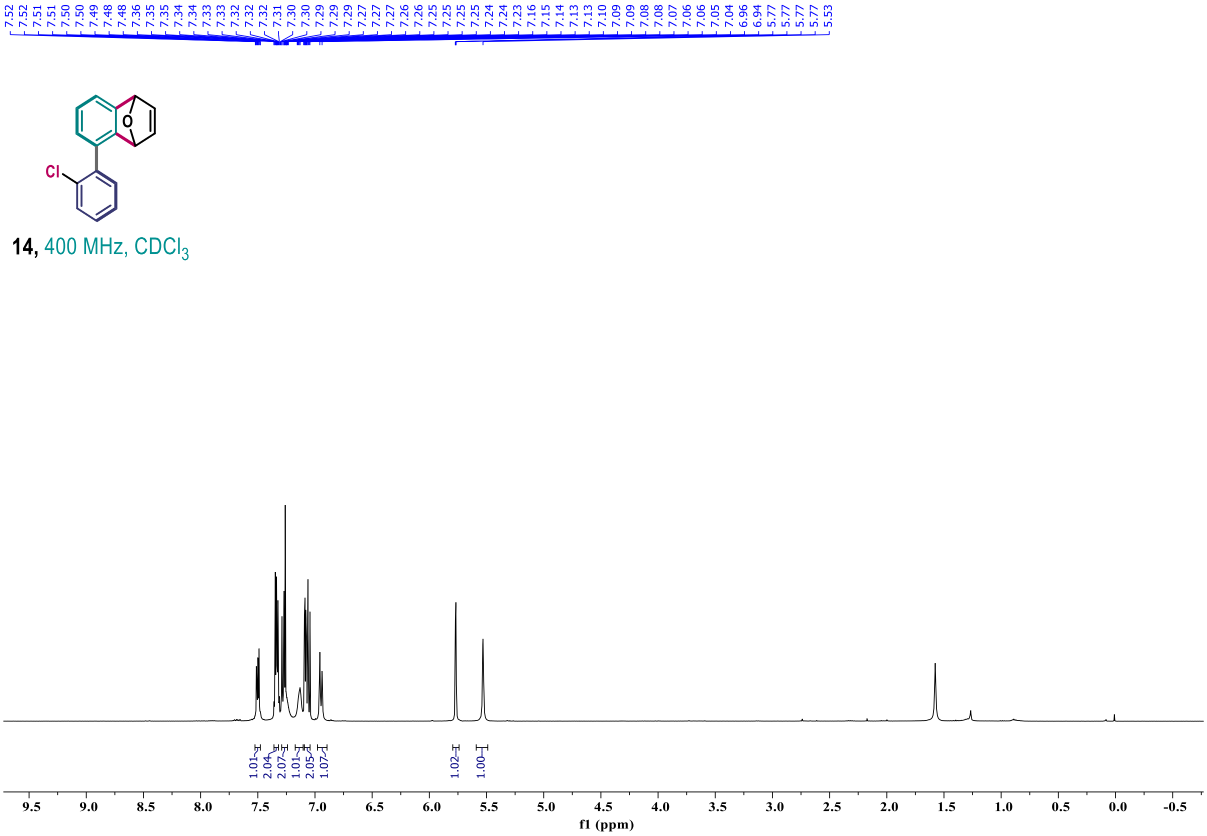
Cl
O
14, 400 MHz, $CDCl_3$
f1 (ppm)

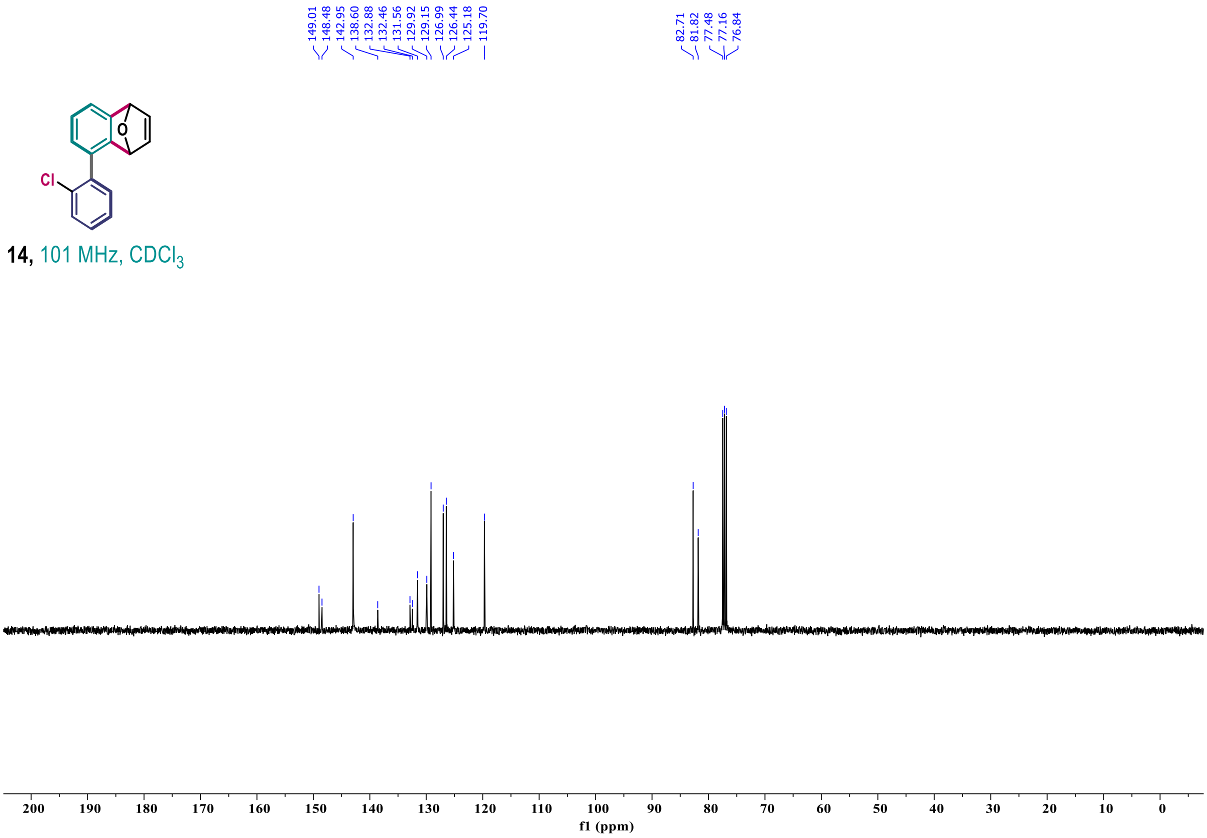
Cl
O
14, 101 MHz, $CDCl_3$
f1 (ppm)
